# Abstracts of the 15th Annual Meeting of the Israel Society for Neuroscience  Eilat, Israel, December 3–5, 2006

**DOI:** 10.1155/2007/73079

**Published:** 2006-12-11

## Abstract

The Israel Society for Neuroscience (ISFN) was founded in 1993 by a group of Israeli leading scientists conducting research in the area of neurobiology. The primary goal of the society was to promote and disseminate the knowledge and understanding acquired by its members, and to strengthen interactions between them. Since then, the society holds its annual meeting every year in Eilat during the month of December. At these annual meetings the senior Israeli neurobiologists, their teams, and their graduate students, as well as foreign scientists and students, present their recent research findings in platform and poster presentations. The meeting also offers the opportunity for the researchers to exchange information with each other, often leading to the initiation of collaborative studies. Both the number of members of the society and of those participating in the annual meeting is constantly increasing, and it is anticipated that this year about 600 scientists will convene at the Princess Hotel in Eilat, Israel.

Further information concerning the Israel Society for Neuroscience can be found at http://www.isfn.org.il.

*Committee:*

**Zvi Wollberg** (President) Tel Aviv University

**Edi Barkai** University of Haifa

**Etti Grauer** Israel Institute for Biological Research, Ness Ziona

**Yoram Rami Grossman** Ben Gurion University of the Negev

**Yoel Yaari** Hebrew University of Jerusalem

**Gal Yadid** Bar-Ilan University

**Shlomo Rotshenker** (President Elect) Hebrew University of Jerusalem

**Ettie Grauer** (Treasurer) Israel Institute for Biological Research, Ness Ziona

**Michal Gilady** (Administrator) Rishon Le Zion

Nitric oxide (NO) is produced in the brain by both neurons and astrocytes. It has been well accepted that neurons expressing the neuronal form of NOS (nNOS) are capable of rapid release of small amounts of NO serving as a neurotransmitter. On the other hand, astrocytic NO production has been demonstrated mainly as a slow reaction to various stress stimuli such as ischemia or inflammation, through the activity of an inducible NOS isoform (iNOS). Previous data from our laboratory described for the first time rapid astrocytic NO release in brains of healthy animals. To further explore the role of astrocytic-produced NO, we examined the iNOS knockout (KO) mouse. In this mutant, the fragment containing the calmodulin-binding domain of iNOS is replaced by the neomycin resistance gene. Homozygous KO mice are born with expected frequency and display no abnormalities except for increased susceptibility to systemic infections. NO-imaging and biochemical NOS enzymatic assay revealed compensatory NOS activity in mutants' neocortex. To test whether neuronal networks were modified by the mutation, we compared the performance of KO mice to their WT controls on basic behavioral tests. The motor activity and explorative behavior of the KO mice were identical to their controls on the hole-board and open-field tests. However, KO mice revealed highly significant differences in the parameters which indicate increased anxiety levels: thus, grooming during open-field and hole-board tests was almost completely suppressed in KO compared to WT mice. In the Elevated-Plus maze, KO mice fully avoided the open arms and exhibited lower number of stretch/attend postures. Furthermore, they showed increased freezing when placed in the center of the Open-Field and ventured less into the center of the field than the WT controls. The existence of a distinct behavioral phenotype in the iNOS KO mouse supports the idea that astrocytic-produced NO participates in Modulating neuronal function.
