# Supplementary material for: Abstracts of the 15th Annual Meeting of the Israel Society for Neuroscience : Eilat, Israel, December 3–5, 2006
Source: Neural Plast. 2006 Dec 11;2007:73079. doi: 10.1155/2007/73079 (PMC2377328; doi:10.1155/2007/73079)
Supplement: Related texts

# Abstract: TO WHAT EXTENT CAN PIGEONS LEARN NEW CATEGORIES?

**Authors:** R. Adam, M. Manns, O. Gunturkun

**Affiliations:** ^1^Department of Biopsychology, Institute of Cognitive Neuroscience, Ruhr University Bochum, 44780 Bochum, Germany; ^2^International Graduate School for Neuroscience (IGSN), Ruhr University Bochum, 44780 Bochum, Germany

Humans have concepts, which mean the ability to generalize within a class of stimuli and to discriminate between the classes. Pigeons are also capable of discriminating natural concepts such as “Human” and “Fish.” The learning was amazingly fast but it is unclear to what extent pigeons are really able to establish abstract concepts or even categories. To address this problem, we used a novel complex and artificial category, with no previously functional relevance for the birds to prevent embedded conceptual knowledge. We chose the category “Pikachu,” the main character in Pokmon, a kids fantasy world created by Nintendo. Go stimuli are characterized by the presence of the character “Pikachu.” Other Pokémon characters appeared in Go's and NoGo's. They appeared in various sizes and angles, and could be only partially shown. Four pigeons were trained in a standard Go-NoGo task. In each session a mean of 20 Go's and 20 NoGo's were drawn randomly from a larger set. The traditional rho value was used to compare performances. 48 ± 20 sessions were required till reaching the criterion of rho = 0.85 in three consecutive sessions. A transfer test examined the subjects' ability to generalize beyond the known examples. In each transfer session 12 new stimuli were embedded in 40 old training stimuli. Two transfer procedures were conducted. Two pigeons had 5 transfer sessions with 64 new nonreinforced, repeated transfer stimuli. The other birds were trained in six sessions with a total number of 68 reinforced and thus unrepeated transfer stimuli. The mean performances were rho = 0.694 ± 0.15, 0.581 ± 0.17 for the two groups, respectively. This indicated that categorization of “Pikachu,” as a novel artificial and complex 2D category was not achieved although the animals could memorize a larger number of highly complex stimuli. Past exposure could play a role in previous categorical learning by pigeons. An open question is how crucial the language component is to novel concept discrimination.

# Abstract: ACTIVITY-INDUCED LONG-TERM POST-BURST AHP REDUCTION IS OCCLUDED BY LEARNING

**Authors:** H. Adelstein, S. Cohen-Matsliah, E. Barkai

**Affiliations:** Departments of Biology and Neurobiology, Faculty of Sciences, University of Haifa, Haifa 31905, Israel

We have previously shown long-lasting olfactory-learning induced reduction in the post-burst afterhyperpolarization (AHP) in piriform cortex pyramidal layer II pyramidal neurons. Although much effort has been made to identify such learning-related long-term modifications in intrinsic neuronal properties, and explore their functional significance, the cellular mechanisms by which these modifications are induced are yet to be described. In brain slice preparation, it has been shown that intense activation of glutamatergic synapses receptors induces long-lasting reduction of the AHP and enhanced neuronal excitability in a kainite-receptor dependent process.Here we examine whether learning-induced reduction of the post burst AHP occludes subsequent reduction of the AHP by repetitive synaptic activation. Rats were trained in an olfactory discrimination task to distinguish between positive and negative odor cues until they demonstrated rule learning. Intracellular recordings from piriofm cortex layer II pyramidal neurons were performed three days after training completion. Twenty stimuli, at frequency of 50 Hz, were applied to the intrinsic fibers (layer Ib), after stimulus intensity was adjusted to evoke a 10 mV PSP in the recorded neuron. Within 60 minutes after application of repetitive stimulation, the averaged AHP value in four neurons from control rats was significantly reduced from 7.48 mV to 4.00 mV (*n* = 6, P < .01). The same treatment had no effect on four neurons for trained rats (from averaged values 4.83 mV before stimulation to 5.04 mV 60 minutes after stimulation, *n* = 5). Consequently, sixty minutes after stimuli, the averaged amplitudes of neurons from control and trained rats did not differ for each other. The data support the notion the learning-induced long lasting reduction of the post-burst AHP id triggered by intense glutamatergic release that occurs due intense synaptic activation during the learning process.

Supported by grant fromthe ISF.

# Abstract: INDIVIDUAL DIFFERENCES IN BRAIN REACTIVITY TO STRESS-RELATED CONTENT: PRELIMINARY RESULTS FROM A PROSPECTIVE fMRI STUDY

**Authors:** R. Admon, O. Stern, O. Rahamim, G. Lubin, T. Hendler

**Affiliations:** ^1^Functional Brain Imaging Unit, Wohl Institute for Advanced Imaging TASMC, Israel; ^2^Department of Psychology and Physiology, Tel Aviv University, Tel Aviv 69978, Israel; ^3^Medical Science, Faculty of Medicine, Tel Aviv University, Tel Aviv 69978, Israel; ^4^Mental Health, IDF, Israel

What determines our individual's manner to handle stress? A common consent is that certain personality traits may render individual's vulnerability to stress and to pathological reaction that may follow it. For example, elevated levels of neuroticism were found significantly associated with the occurrence of post traumatic stress disorder (PTSD). Prior imaging studies suggest that the amygdala, hippocampal gyrus, prefrontal cortex, and high-order visual areas are all brain regions involved in PTSD. However, it is not clear if those regions mediate the individual a priori vulnerability to stress or the post trauma abnormal processing of stress related content. The present study aimed to prospectively evaluate the individual brain reactivity to stress-related content in relation to a priori personality trait of neuroticism. For that we studied healthy subjects at two time points (before and after) a significant stressful life event which is mandatory recruitment to the Israeli Defense Forces (IDF) serving as combat paramedics. We hypotheses that the brain reactivity/sensitivity to visual content related to recently experienced stressful life events will correspond to the level of neuroticism. We report here on the results of the first time point from 50 soldiers (25F, age 18–20). Soldiers were scanned (3T. GE) while watching two relevant contents and two neutral contents: military, medical, neutral, and scrambled, respectively. In order to evaluate brain reactivity to those contents we used backward masked images presented at 33 ms or 83 ms. We found overall greater activation for high than low neurotics in the amygdala, enthorhinal cortex, and orbitofrontal cortex. These initial results suggest that certain brain areas in high neurotics are hyperactive during stress related visual detection task. Interestingly, those regions have been indicated before as mediating the expression of core personality traits.

# Abstract: CODING 3D BY 3V

**Authors:** E. Ahissar

**Affiliations:** Weizmann Institute of Science, Rehovat 76100, Israel

Rats use their whiskers (vibrissae) to localize and identify objects in their vicinity. We recently revealed the neural codes used by vibrissal receptors to encode the coordinates of object location in three dimensions. We found that the most efficient neural code for each of the three spatial dimensions is different: temporal for the horizontal axis (along whisker rows), spatial for the vertical (along whisker arcs), and rate for the radial axis (from the face out). What is the advantage of such triple coding scheme? In theory, suchmultiplexing of sensory information increases channel efficiency as less neurons and less axons are required; the same neurons can convey information on three dimensions at the same time without losing accuracy or reliability. By the fact that it fires, a neuron conveys information about the vertical coordinate of the object. The time of its firing, in relation to other “reference” neuronal signals, conveys information about the horizontal coordinate, and the number of spikes it generates during one whisking cycle conveys information about the radial coordinate. The output signal of this neuron can be conveyed in parallel to different readout circuits, each decoding one specific variable. In practice, however, the rat probably does not limit itself to one such framework. Our behavioral data indicate that (a) rats have enormous motor flexibility in controlling whisker movements, and (b) rats continuously adapt their motor strategies. How rats exploit motor flexibility and efficient coding to achieve their goals is a serious challenge for future research.

*Minerva, BSF, and HFSP EA hold the Helen Diller Family Professorial Chair in Neurobiology.*

# Abstract: THE POSSIBLE INVOLVEMENT OF THE DOPAMINE D2-RECEPTOR PATHWAY COMPONENTS IN SCHIZOPHRENIA

**Authors:** S. Amar, G. Shaltiel, A. Shamir, L. Mann, R. H. Belmaker, G. Agam

**Affiliations:** Stanley Research Center, Ben Gurion University of the Negev and Mental Health Center, Beer Sheva 84105, Israel

*Background*. Antipsychotics used in schizophrenia are dopamine-D2-receptor (DA2R) antagonists. Dopamine activation of DA2R causes downregulation of protein kinase A (PKA) activity and cAMP production. Prostate apoptosis response 4 (Par-4) mediates dopamine-induced PKA inhibition. Prolonged DA2R activation leads to G-protein-coupled receptor desensitization involving two protein families— GRKs and beta-arrestins. Beta-arrestins bind to phosphorylated receptors preventing further G protein stimulation and downstream signaling. The AKT1-GSK-3beta pathway has also been implicated in schizophrenia [1]. *Objective and methods*. To examine whether Par-4, beta-arrestin1, AKT1 and GSK-3beta are involved in the pathophysiology of schizophrenia, protein levels of Par-4 and beta-arrestin1 were measured in lymphocyte-derived cell-lines and AKT1 and Ser-9-phospho-GSK-3beta- in postmortem frontal cortex from schizophrenia patients versus healthy controls. *Results*. beta-arrestin1 levels were ∼2 fold increased and Par- 4—unaltered in schizophrenia. Ser-9-phospho-GSK-3beta levels were ∼50% decreased but, in contrast with Emamian et al [1], no difference in AKT1 levels was found. Conclusions. DA2R hyperactivation hypothesized to occur in schizophrenia may lead to a compensatory beta-arrestin1 upregulation and decreased in the GSK-3beta's inactive form (phosphorylated on Ser-9) due to PKA inactivation. Beta-arrestins are also signal transducers on their own [2]. As such, elevated beta-arrestin1 would result in elevated, rather than decreased, Ser-9-phospho-GSK-3beta, suggesting that betaarrestin1 is not a signal transducer of the PI3K-AKT1-GSK- 3beta cascade in the frontal cortex.

## References

[1] Emamian ES, Hall D, Birnbaum MJ, Karayiorgou M, Gogos JA (2004). Convergent evidence for impaired AKT1-GSK3*β* signaling in schizophrenia. *Nature Genetics*.

[2] Lefkowitz RJ, Shenoy SK (2005). Transduction of receptor signals by *β*-arrestins.. *Science*.

# Abstract: STRESS-INDUCED CHANGES OF S100ß AND GFAP-IMMUNOREACTIVE ASTROCYTES IN THE RODENT PREFRONTAL CORTEX

**Authors:** R. Antemano, C. Helmeke, K. Braun

**Affiliations:** Department of Zoology/Developmental Neurobiology, Otto von Guericke University Magdeburg, Germany

The fine tuning of synaptic networks in cortical and limbic brain areas is known to be dramatically affected by environMental factors, in particular by neonatal experiences and learning events. Since astrocytes play an important role in neuronal plasticity during brain development, we investigated whether they respond to environMental stimulation, which would indicate their possible involvement in experience-induced synaptic plasticity. To test this hypothesis, the impact of adverse juvenile emotional experience (6 hours of isolation stress) on glial plasticity was assessed in the medial prefrontal cortex (mPFC) and in the somatosensory cortex (SSC) of 19-day-old trumpet-tailed rats (Octodon degus) 1 hour (short interval = SI) or 48 hours (long interval = LI) after stress exposure. Densities of S100ßimmunoreactive astrocytes in each cortical layer were quantitatively compared between the two isolation groups and a nonstressed control group. In comparison to controls, SI as well as LI pups displayed significantly increased densities of S100ß-immunoreactive astrocytes in layer II-III and layer VVI but not in layer I, whereas only the SI pups showed significantly reduced densities of GFAP-immunoreactive astrocytes in the mPFC. Isolation stress had no effect on S100ßand GFAP-immunoreactivity in the somatosensory cortex. Furthermore, we observed higher numbers of astrocytic processes and astrocytic nodes in the mPFC of LI animals in comparison to controls, soma size of astrocytes did not differ between groups. Our results confirm our hypothesis that in the juvenile brain astrocytes respond to emotional stimulation in a region-, time-, and layer-specific manner. These alterations in astroglial reaction may have consequences in neuron-glia interactions and thereby affect the involvement of astrocytes in modulating synaptic activity.

*Supported by the German-Israeli Foundation (GIF).*

# Abstract: DISRUPTED LATENT INHIBITION IN CYCLIC AND OVARIECTOMIZED FEMALE RATS CAN BE REVERSED BY CLOZAPINE BUT NOT BY HALOPERIDOL

**Authors:** M. Arad, I. Weiner

**Affiliations:** Department of Psychology, Tel Aviv University, Tel Aviv 69978, Israel

Relapse rates in schizophrenic women are elevated when estrogen levels are low during the follicular phase, postpartum, and perimenopausal periods; whereas a remission in symptoms is observed when estrogen levels are high during the lutheal phase and pregnancy. Based on this line of findings, the estrogen hypothesis of schizophrenia postulated that estrogen has a neuroprotective effect delaying the onset of schizophrenia and reducing severity of symptoms in women. Latent inhibition (LI), the poorer conditioning to a stimulus that received nonreinforced preexposure prior to conditioning, is disrupted in rats and humans treated with the psychosis inducing drug amphetamine and in acute schizophrenia patients. Hence, we investigated the LI phenomenon and its modulation by gonadal hormones in cyclic and ovariectomized female rats. LI was measured in a thirst motivated conditioned emotional response procedure. Our results demonstrate that (1) females showed LI only if preexposure took place during estrus and conditioning took place during metestrus (estrus-metestrus), but not during the remaining sequential phases of the estrous cycle; (2) both haloperidol and clozapine restored LI in proestrousestrous rats but failed to restore LI in diestrous-proestrous rats, while LI in the metestrous-diestrous group was restored only by clozapine; (3) LI was disrupted in ovariectomized rats and restored only by clozapine. These results suggest that the expression of LI and its sensitivity to neuroleptics in female rats is correlated with hormonal fluctuation. Moreover, resistance of disrupted LI to haloperidol contrasts with its efficacy to restore amphetamine-induced LI disruption, suggesting that hormonal-induced LI disruption might reflect different underlying mechanisms. Considering the link between estrogen and psychotic outbreaks, clarifying the role of gonadal hormones in LI, may shed light on their role in schizophrenia as well as on gender differences in this psychopathology.

# Abstract: AGE- AND GENDER-DEPENDENT COGNITIVE DEFICITS IN A TRIPLE TRANSGENIC MOUSEMODEL OF ALZHEIMER'S DISEASE

**Authors:** A. D. Aran, Y. David, R. Duvdevani, J. E. Friedman, E. Yavin, A. Kozak

**Affiliations:** ^1^D-Pharm, Ltd, Rehovot, Israel; ^2^Department of Neurobiology, Weizmann Institute of Science, Rehovot 76100, Israel; ^3^Biotechnology Department, Ben Gurion University, Beer Sheva 84105, Israel

The relationship between amyloid-beta (A-b) plaques and tau hyper-phosphorylation on cognitive decline in Alzheimer's disease (AD) is still poorly understood. We have evaluated cortical, amygdala (AMY) and hippocampal (HIP) behavioral activity in a triple transgenic mouse (3xTg) model which consists of three mutant human genes (APPKM670/671NL, PS1M146V, and tauP301L) that develop A-b and tau pathologies in an age-dependent manner. Mice at three different ages (2, 10, and 16 months old) were assessed in a battery of behavioral tests: object recognition (OR), Morris water maze (MWM), and Pavlovian fear conditioning (PFC). Spatial reference memory was not affected by transgenic status, age (up to 16 months) or gender, but impairments related to age and gender were found in the retention test and in the reversal learning test, which may indicate dorso-HIP and putative frontal cortex dysfunction. Similar results were found in the object recognition test, a test that exploits the rodent's spontaneous preference for novel objects and strongly relies on visual memory, indicating also possible deficits in HIP and non-HIP structures like the perirhinal cortex. All 3xTg mice responded similarly when tested for PFC, indicating no detectable deficits or abnormalities in the brain areas that are responsible for such behavior. This includes the ventral hippocampus in the context test and the AMY in the tone test. Histological analysis of brain sections revealed a significant number of plaques in Hippocampus, cortex, as well as AMY and subiculum. We conclude that the impairments in cognitive flexibility and OR in older 3xTG mice are indicative of HIP, frontal and entorhinal cortex dysfunction and may be correlated with the appearance of A-b plaques and Tau phosphorylation in these brain areas. However, the presence of A-b plaques and phosphorylated Tau in the AMY and subiculum was not correlated with the PFC test results presented here.

*ADA, RD, JEF, and AK are employees of D-Pharm, Ltd. This work was supported, in part, by D-Pharm.*

# Abstract: ONLINE MOTOR ADJUSTMENTS IN SEQUENTIAL ADAPTATION TO ALTERED KINEMATICS AND DYNAMICS DEPEND ON PERTURBATION DIRECTION

**Authors:** F. Arce, I. Novick, E. Vaadia

**Affiliations:** ^1^Department of Physiology, Faculty of Medicine, Israel; ^2^Interdisciplinary Center for Neural Computation, Israel; ^3^The Hebrew University, Jerusalem 91010, Israel

A most common approach to study adaptations to novel sensorimotor associations is the interference paradigm (Krakauer et al, 1999; Boch et al, 2001; Shadmehr and Brashers-Krug, 1997). In this learning scheme, disruption in the stabilization of the motor memory trace, termed interference, may be retrograde, where the second task disrupts the retention of the newly acquired first task, or anterograde when the second task impairs performance of the first task on retest (Robertson et al, 2004). We sought to investigate the mutual interactions between visuomotor rotation and viscous force field to which subjects adapted in sequence. We previously showed results of facilitation and interference in the initial directional planning of reach movements (Arce et al, ISFN Abstract, 2005). Here we show the interactions involving late trajectory correction. Like the results found in direction planning, we found mutual facilitation in the direction-matched perturbations requiring matched motor adjustments. In contrast, opposite perturbations requiring opposing adjustments interfered. The presence of facilitation or interference suggests that the newly-acquired internal representations of kinematic and dynamic perturbations are not independent but they share common neuronal resources. Such overlap does not necessarily imply competition of resources.

*Bundesministerium fur Bildung und Forschung (BMBF-DIP), BSF, Jack Skirball Chair and Research Fund.*

# Abstract: NEURONAL CONDITIONING MEDIUM AND NERVE GROWTH FACTOR INDUCE NEURONAL DIFFERENTIATION OF COLLAGEN-ADHERENT PROGENITORS DERIVED FROM HUMAN UMBILICAL CORD BLOOD

**Authors:** H. Arien-Zakay, A. Nagler, H. Galski, P. Lazarovici

**Affiliations:** ^1^Department of Pharmacology and Experimental Therapeutics, The Hebrew University, Jerusalem 91010, Israel; ^2^Hematology Division and Cord Blood Bank, Israel; ^3^Laboratory of Molecular Immunobiology, Chaim Sheba Medical Center, Tel Hashomer, Israel

Research in the field of neuronal progenitors is rapidly advancing, driven by the potential use of these cells for cellular therapy of neurodegenerative disorders, stroke, trauma, and spinal cord injuries. The aim of the study was to isolate and characterize a population of neuronal progenitors in the human umbilical cord blood (HUCB) mononuclear cell (MNC) fraction, for in vitro manipulation towards neuronal differentiation. Selection of the HUCB neuronal progenitors (HUCBNPs) was based on the neuronal prerequisite of adherence to collagen. Populations of collagen-adherent, nestin-positive (94.8 ± 2.9%) progenitors expressing alpha 1/2 integrin receptors, as revealed byWestern blot and adhesion assay using selective antagonists, were isolated and survived for more than 14 days. In vitro differentiation of the HUCBNPs was achieved by treatment with neuronal conditioning media (CM) supplemented with 10 ng/mL nerve growth factor (NGF). Some 83 ± 8.2% of the surviving progenitors acquired a neuronal-like morphology, expressed by cellular outgrowths of different lengths. About 35 ± 6% of the HUCBNPs had long outgrowths with a length/cell diameter ratio greater than 2, typical of developing neurons. The majority of these progenitors, analyzed by immunocytochemistry and/or RT-PCR, expressed common neuronal markers such as microtubule-associated protein 2 (MAP-2; 98.5 ± 2%), neurotrophin receptor (TrkA; 98.5 ± 0.06%), neurofillament-160 (NF-160; 94.2 ± 1%), beta-tubulin III (89.8 ± 4.2%) and neuron specific enolase (NSE). Combined CM and NGF treatment induced constitutive activation of the mitogen-activated protein kinases ERK2, p38alpha and p38beta (36, 9, and 23-fold, resp, versus the control), most likely related to survival and/or differentiation. The results point to operationally defined conditions for activating HUCBNPs neuronal differentiation ex vivo and emphasize the crucial role of neuronal CM and NGF in this process.

# Abstract: MRI CHARACTERIZATION OF CNS NBS-1 KNOCKOUT MICE

**Authors:** Y. Assaf, I. Shapira, R. Galron, Y. Shiloh, A. Barzilai

**Affiliations:** ^1^Department of Neurobiochemistry, Faculty of Life Sciences, Tel Aviv University, Tel Aviv 69978, Israel; ^2^Department of Molecular Genetics and Biochemistry, Sackler Medical School, Tel Aviv University, Tel Aviv 69978, Israel

Nijmegen Breakage Syndrome (NBS) is a rare autosomal recessive disorder caused by a mutation in the gene NBS-1. NBS-1 produces nibrin, a protein believed to play a significant role in DNA damage response. NBS patients suffer from many of the symptoms of Ataxia-telangiectasia (variant V1) subjects including immunodeficiency, chromosomal instability, and lymphoreticular malignancies. The disorder itself causes brain microcephaly, that leads to severe developmental disorders and that frequently lead to pre-mature death of the subjects. In this work we have conducted an MRI study on conditional CNS Nbs1-del and wild-type (WT) mice in order to characterize, for the first time, the in vivo appearance and integrity of various brain structures in NBS. T2 weighted MRI revealed significant morphological and contrast changes between the CNS Nbs1-del and WT groups. First, the brains of the CNS Nbs1-del mice were significantly smaller than those of the WT with emphasized mal-development of the cerebellum. In addition, the typical hypo-intense white matter signal in the corpus callosum, internal-capsule, and cerebellar folia disappeared. Region of interest analysis revealed that white matter areas of CNS Nbs1-del mice have significantly higher T2 values than those of WT mice. The white matter areas that were studied were the corpus callosum, the cerebellar folia, internal capsule, and optic nerve. In contrast, any of the studied gray matter areas did not show differences between the two groups except for the hippocampus in which the T2 of the CNS Nbs1-del group was higher than the WT group. Higher T2 values in the white matter and hippocampus might indicate a progressive degenerative process occurring in these regions. These results suggest that white matter damage plays a significant role if not the leading role in the degeneration of CNS Nbs1-del mice brains. The observed damage might imply that damage to the oligodendrocytes is more severe than other types of cells in NBS.

# Abstract: NOVEL STRATEGY TERMED “REDOX CLUSTER BOMB” FOR THE TREATMENT OF NEURODEGENERATIVE DISORDERS

**Authors:** D. Atlas

**Affiliations:** Department of Biological Chemistry, Silberman Institute Life Sciences, The Hebrew University, Jerusalem 91010, Israel

Oxidative stress caused by various stimuli leads to oxidation of glutathione (GSH), the major redox power of the cell. Lower GSH levels correlated with the activation of mitogenactivated proteins kinases (MAPK) have been demonstrated in Alzheimer's disease, Parkinson's disease, and other neurodegenerative disorders and have been proposed to play a central role in the deterioration of the aging and neurodegenerative brain. As a potential use for neurodegeneration we have developed a novel strategy termed the “Redox Cluster Bomb.” It consists of a matrix of low molecular weight and nontoxic thiol compounds that are also inhibitors of MAPKs, p38, and ERK1/2. Upon penetration into the cell through the plasma membrane, the molecule is cleaved by the intracellular enzymatic machinery to generate a large number of smaller molecules, each one has the power to scavenge free radicals, generate GSH, and protect the cell from oxidation. This property gives an amplified action as well as a prolongation of the redox effects. Our results are consistent with the notion that the “cluster bomb” strategy may play an efficient protective role against neurotoxicity and thus would be suited for the treatment of neurodegenerative disorders.

# Abstract: GATING AND ASSEMBLY MODALITIES OF A HUMAN CARDIAC POTASSIUM CHANNEL: LESSONS FROM LONG QTMUTATIONS

**Authors:** B. Attali

**Affiliations:** Department of Physiology and Pharmacology, Sackler Faculty of Medicine, Tel Aviv University, Tel Aviv 69978, Israel

KCNQ1 potassium channels are members of the superfamily of voltage-gated K+ channels. The KCNQ1 pore-forming alpha subunit interacts with the KCNE1 auxiliary subunits to form the slow IKS K+ current which plays a major role in repolarizing the cardiac action potential. Mutations in either KCNQ1 or KCNE1 genes produce the long QT (LQT) syndrome, a life-threatening ventricular arrhythmia. We will discuss the biophysical and structural properties of two important gating modules, the pore and the C-terminus of KCNQ1. Removal of external Ca2+ produces a striking voltage-dependent macroscopic inactivation in WT KCNQ1 channels. Adding external Ca2+ suppresses the macroscopic inactivation with an EC50 of 1.5 micromolar. Mutagenesis studies and structural modeling indicate that external Ca2+ ions are tightly coordinated by two glutamate residues located at the outer pore in the turret region. Thus, external Ca2+ exquisitely controls KCNQ1 channel gating by preventing relaxation into slow macroscopic inactivation. We identified a similar slow inactivation in a KCNQ1 LQT pore mutant which hinders entry of external Ba2+ to its deep site in the pore and traps it by slowing its egress. Kinetic studies, structural modeling and dynamics simulations suggest that this slow inactivation involves conformational changes that converge to the selectivity filter and constrict the outer carbonyl ring of site s1, where the backbone becomes less flexible. This mechanism considerably differs from C-type inactivation where vacation of K+ from the filter was invoked. We suggest that trapping of K+ at s1 and consequent hindrance of the dehydration-resolvation transition underlie the slow inactivation mechanism of KCNQ1 channels. Biophysical and structural analysis of the KCNQ1 C-terminus and LQT mutants indicates that in healthy individuals, CaM binding to KCNQ1 is essential for correct channel folding, assembly, and gating.

*Supported by ISF (672/05) and the Keren Wolfson Family funds.*

# Abstract: SEROTONIN MODULATES THE FIRING PATTERNS OF CEREBELLAR PURKINJE CELLS IN GUINEAPIG, INVIVO

**Authors:** L. Atzmon, G. Jacobson, Y. Yarom

**Affiliations:** ^1^Department of Neurobiology, Institute for Life Science, The Hebrew University, Jerusalem 91010, Israel; ^2^Interdisciplinary Center for Neural Computation, Hebrew University, Jerusalem 91010, Israel

Purkinje cells are the sole output of the cerebellar cortex. As such, the patterns of action potentials in these cells are central to cerebellar function. Bi-stability of Purkinje cell membrane potential has been demonstrated both in vitro and in vivo. Currently there is an ongoing debate regarding the existence of Purkinje cell bi-stability in awake animals. Serotonin (5-HT) has been shown to emphasize bi-stability in slice preparation (Williams et al, 2002), and is known to be elevated in the cerebellum of awake animals during alertness and around significant events (Schonewille et al, 2006). To investigate the role of 5-HT in controlling bi-stability in intact animals, we performed extracellular recordings of Purkinje cell activity in anesthetized guinea pigs using sharp glass pipettes, and locally applied 5-HT using a custom-built injection pipette whose tip was 100–300 *μ*m away from the recording pipette. Purkinje cell was recorded from the superficial layer of the cerebellar cortex and identified by complex spikes existence. We compared the spontaneous activity of Purkinje cells before and after application of serotonin. We analyzed the modulation in simple spikes activity focusing on the statistics of the inter-spike-intervals of the simple spikes and their instantaneous firing rate. We found that 5-HT significantly modulated the firing patterns of cerebellar Purkinje cells. Two significant modulations were observed: (1) tonically firing Purkinje cells exhibit bi-stability after 5-HT application. Since there was a clear tendency toward longer quiescent period, we propose that 5-HT accentuated the bi-modal behavior of Purkinje cell. (2) The firing of the Purkinje cells becomes more regular, and the distribution of the higher firing frequencies is shifted towards lower values and fewer instances of low frequencies are apparent.

# Abstract: DOPAMINE NEURON DEGENERATION IN C. ELEGANS: IDENTIFICATION OF GENETIC AND CHEMICAL MODULATORS IN A NOVEL MODEL OF PARKINSON'S DISEASE AND MANGANISM

**Authors:** C. Au, M. Fullard, M. Aschner, R. Nass

**Affiliations:** Departments of Pediatrics and Pharmacology, Vanderbilt University Medical Center, Nashville, TN, USA

Parkinson's disease (PD) and manganism are characterized by motor deficits and dopamine neuron dysfunction, and dopamine or its metabolites are believed to contribute to the disorders. Furthermore, expression of the presynaptic protein a-synuclein, and the oxidative stress-induced protein parkin have been proposed to contribute to the pathogenesis of both disorders, and occupational exposure to Mn2+ has been invoked to predispose individuals to PD. Despite the initial characterization of these disorders well over a century ago, and intensive research within the past several decades, the origin of the pathogenesis and the molecular determinants involved in PD and manganism have yet to be fully elucidated. A significant hindrance in dissecting the molecular components is the high complexity of the vertebrate brain and lack of facile in vivo genetic models to determine and explore the mechanisms involved in the cell death. We have developed a novel pharmacogenetic model using the genetically tractable nematode *C elegans* to dissect and characterize the molecular components involved in DA neuron degeneration. We show that the DA neurons are sensitive to the PD-associated neurotoxin 6-OHDA and Mn, and cell death likely occurs through a novel pathway. We also show that a number of PD-associated genes contribute to the cell death. We have now instituted genetic and chemical screens for regulators of toxin-mediated cell death, and we have isolated several mutants and compounds that suppress the neurodegeneration. This system will allow us a facile test to examine the role xenobiotics and Mn2+ play in the degeneration of DA neurons.

*Supported in part by NIEHS 106563 and DoD W81XWH-05- 1-0239 to the third author, and R01 ES014459 to the fourth author.*

# Abstract: AGED SOD OVEREXPRESSING MICE EXHIBIT ENHANCED SPATIAL MEMORY AND A LACK OF NEUROGENESIS

**Authors:** A. Avital, A. Kamsler, V. Greenberger, M. Segal

**Affiliations:** ^1^Department of Neurobiology, The Weizmann Institute of Science, Rehovot 76100, Israel; ^2^Department of Behavioral Science, The Max Stern Academic College Emek Yezreel, Emek Yezreel 19300, Israel

Our recent finding that hippocampal slices from aged mice overexpressing the gene for superoxide dismutase (SOD) exhibit long-term potentiation (LTP) of reactivity to afferent stimulation that is significantly larger than that produced in aged wild-type (wt) mice has encouraged us to explore the effects of reactive oxygen species (ROS) on learning in aged mice. In addition, we used young-adult and aged wt and SOD transgenic mice in an attempt to correlate adult neurogenesis with spatial learning. Aged wt and SOD mice exhibited a 90% reduction in doublecortin-labeled new dentate gyrus neurons as compared to young mice with no significant difference between genotypes. In addition, aged SOD mice exhibited better performance than wt controls in both reference and working memory tasks in a water maze. These findings provide a behavioral measure to demonstrate that excessive production of H_2_O_2_ is beneficial in aged mice.

# Abstract: ANOREXIA NERVOSA: FROM ANIMAL MODELS TO CLINICAL TRIALS

**Authors:** Y. Avraham, O. Zolotarev, Y. Dagon, I. Magen, L. Vorobiav, Y. Latzer, M. Israeli, R. Mechoulam, E. Berry

**Affiliations:** ^1^Department of Human Nutrition and Metabolism, Hadassah Medical School, Jerusalem, Israel; ^2^Department of Eating Disorders, Rambam Hospital, Israel; ^3^Department of Medicinal Chemistry and Natural Products, Hadassah Medical School, Jerusalem, Israel

Anorexia nervosa (AN) is a potentially life threatening eating disorder of unknown origin for which no effective drug treatment is currently available. It occurs in women in adolescence and is characterized by severe low weight, cognitive distortions about body shape and weight and amenorrhea. It begins with “harmless” attempts at dieting which gets out of control. The patients think themselves to be too fat even when severely underweight. Understanding the interaction between severe reduction of body weight and cognitive function may lead to new strategies for the treatment of AN. Three kinds of animal models have been developed: diet restriction (DR), activity wheel, and separation stress, each of which mimics some aspects of the human disease. DR may benefit or impair animal cognition or motor performance depending on the degree of restriction. DR to 60% improved maze performance whereas 40% DR impaired it and was associated with high mortality. We have studied the effects of tyrosine as a catecholamine precursor, and endocannabinoids, derived from essential fatty acids, as neuromodulators. We have found that severe DR impaired the adrenergic, cholinergic, serotonergic, and opiate systems while tyrosine almost reversed the effects. Tyrosine or EC supplementation to the 40% DR mice improved cognitive function and brain neurotransmitters without increasing body weight. Such a strategy might break the vicious cycle in initiating treatment in patients with AN. Patients sometimes will not respond to supportive and psychological treatment before there is nutritional rehabilitation. Following these results we are currently undertaking clinical trials of THC and tyrosine in the treatment of AN. Treatment with tyrosine or THC caused improved cognitive function, treatment with THC caused differences in food consumption with regard to flexibility of food eaten. Both tyrosine and THC could be used as therapeutic agents for AN.

# Abstract: DYSREGULATED BDNF SECRETION FROM MONOCYTES OF MULTIPLE SCLEROSIS PATIENTS: REVISED BY IFN-BETA THERAPY IN A CD40-DEPENDENT MECHANISM

**Authors:** D. Azoulay, A. Karni

**Affiliations:** Department of Neurology, Sourasky Medical Center, Sackler School of Medicine, Tel Aviv University, Tel Aviv 69978, Israel

Immune cells can be regulated to express a neuroprotective effect trough their capacity to secrete neurotrophic factors.*Objective*. We studied the immune regulation of BDNF secretion from peripheral blood mononuclear cells (PBMCs) of relapsing remitting MS (RR-MS) patients and the effect of IFN-beta therapy on this regulation. *Methods*. PBMCs of 12 healthy controls (HC), 12 untreated (UMS), and 12 INF-beta treated (IMS) RR-MS patients were incubated in the absence or presence of a CD40-activation agonist: anti-CD40 mAb (10 *μ*g/mL). After 24 hours of incubation, the cell supernatants BDNF level was detected by ELISA. HC, UMS, and IMS cells were compared for the secreted BDNF levels obtained in the absence of stimulus (basal secretion) and for the individual ratio between CD40 activation and basal secretion level (BDNF secretion response to CD40 activation). CD40 expression on CD14+ momocytes was studied by flow cytometry.*Results*. The basal BDNF secretion from PBMCs of HC or IMS was found to be high as compared to that of UMS patients (HC 1020.67 ± 304.74 pg/mL, IMS 1336.20 ± 454.73, and UMS 675.04 ± 198.32, *P* < .0001). The PBMCs of UMS patients were found to be significantly less responsive to CD40 activation compared to that of HC or IMS (HC basal/CD40 ratio 1.32 ± 0.24, IMS ratio 1.53 ± 0.31, and UMS ratio 1.08 ± 0.12, *P* < .007). No such effect was shown when matched isotype control or anti-CD3 mAb was added. The surface expression of this CD40 on CD14+ monocytes of HC was significantly enhanced during incubation as compared to UMS (HC %CD14+/CD40+ 1.66 ± 0.55, UMS 0.75 ± 0.1, *P* < .0001). Furthermore, in vitro addition of INFbeta (10 ng/mL) to this UMS cells upregulated their CD40 expression (2.31 ± 0.34) and enhanced BDNF secretion response to CD40 activation.*Conclusions*. Our results show dysregulated BDNF secretion fromMSmonocytes via CD40. We also suggest an additional neuroprotective immunomodulatory effect for INF-beta as it showed to this revised dysregulation.

# Abstract: AGE DIFFERENCES IN BRAIN ACTIVATION DURING OBJECT NAMING PERFORMANCE

**Authors:** R. Babai, M. Lozin, T. Hendler, A. Gigi

**Affiliations:** ^1^Department of Science Education, The Constantiner School of Education, Tel Aviv, Israel; ^2^Department of Behavioral Sciences, College of Judea and Samaria, Ariel, Israel; ^3^Functional Brain Imaging Unit, Whol Institute for Advanced Imaging, Sourasky Medical Center, Israel; ^4^The Joseph Sagol Neuroscience Center, Sheba Medical Center, Tel Hashomer, Israel

Older people usually complain of naming difficulties. However, many studies could not demonstrate decline in naming performance and thus the cognitive and neural source of this problem remains controversial. We constructed a naming test in which common objects pictured from usual viewpoint were presented. Recently we reported that the behavioral naming performance of old participants was similar to young ones (Gigi et al [1]). In the current research, we applied fMRI to study brain activation patterns of young and old participants during naming performance. Seven young adults (mean age 26.2 ± 2.3) and eight older adults (61 ± 14.8) participated in the study. We found brain activations differences between the two age groups. The prominent difference included the right inferior prefrontal cortex (BA47) that appeared to be significantly activated in young participants but not in older participants. Hippocampus and medial-lateral prefrontal cortex (BA9, BA46) activations were found prominently in the older group. Taking into account the lack of age differences in the behavioral performance, and the known loss of frontal cortex volume with age (eg, Raz et al [2]), we suggest that the variation in brain activity between the two age groups relates to brain compensation and alternative networks that enable the successful naming performance of old adults.

*Supported by the Smith Grant (No 2004-41), The National Institute for Psychobiology, Israel.*

## References

[1] Gigi A, Babai R, Hendler T, Katzav E The role of frontal areas in naming of objects pictured from unusual viewpoints.

[2] Raz N, Gunning FM, Head D (1997). Selective aging of the human cerebral cortex observed in vivo: differential vulnerability of the prefrontal gray matter. *Cerebral Cortex*.

# Abstract: INDUCTION OF ADULT HUMAN BONE MARROW MESENCHYMAL STEM CELLS INTO FUNCTIONAL ASTROCYTE-LIKE CELLS: POTENTIAL FOR RESTORATIVE TREATMENT IN PARKINSON'S DISEASE

**Authors:** M. Bahat Stroomza, Y. Barhom, Y. Levy, O. Karpov, S. Bulvik, E. Melamed, D. Offen

**Affiliations:** Laboratory of Neurosciences, Felsenstein Medical Research Center, Israel

Parkinson's disease (PD) is a neurodegenerative disorder mainly caused due to loss resulting in the depletion of dopamine producing neurons in the substantia nigra. Pharmacological treatments aimed to increase the deficient dopaminergic neurotransmission being effective in ameliorating the symptoms of the disease, but none of these therapies are curative. It was suggested that treatment with neurotrophic factors might either protect the remaining healthy dopaminergic neurons or prevent death of damaged neurons and induce proliferation of axonal nerve terminals of surviving neurons. Glial cell line-derived neurotrophic factor (GDNF) is currently the most effective substance shown to increase dopaminergic neuronal survival in culture and promote its survival and axonal growth in animal models of PD. We therefore aimed to differentiate human bone marrow mesenchymal stem cells (MSCs) into astrocyte-like cells, capable of producing neurotrophic factors (NTFs). Indeed, MSCs treated with our novel astrocyte differentiation medium present astocyte-like morphology and express the astrocyte marker, S100â, glutamine synthetase (GS), and glial fibrillary acidic protein (GFAP). Moreover, these astrocyte-like cells produce and secrete significant amounts of GDNF, nerve growth factor (NGF), and brain-derived neurotrophic factor (BDNF) as indicated by mRNA, RT-PCR, ELISA, and Western blot analysis. When these GDNF-producing cells were transplanted into the striatum of 6-hydroxydopamine (6-OHDA) lesioned rats, a model of PD, they produced a progressive reduction in the rotational behavior induced by apomorphine administration as well as improvement in rotor-rod and the unflower- eating tests. Histological assessments revealed that the cells survived and expressed astrocyte and human markers. Our results indicate that GDNF-producing cells derived from human bone marrow stem cells might be used as a new strategy for autologous restorative transplantation in PD.

# Abstract: INTERFERENCE TO CONSOLIDATION PHASE GAINS IN LEARNING A NOVEL MOVEMENT SEQUENCE BY HANDWRITING IS DEPENDENT ON LATERALITY AND THE LEVEL OF EXPERIENCE WITH THE WRITTEN SEQUENCE

**Authors:** M. Balas, S. Netser, N. Giladi, A. Karni

**Affiliations:** ^1^The Brain Behavior Research Center, University of Haifa, Haifa 31905, Israel; ^2^Movement Disorder Unit, Department of Neurology, Tel Aviv Sourasky Medical Center, Tel Aviv, Israel; ^3^Sackler School of Medicine, Tel Aviv University, Tel Aviv 69978, Israel

*Background*. There are two expressions of procedural memory consolidation: (a) delayed performance gains evolving hours after training, and (b) a decrease in the susceptibility of the training-related gains to interference by subsequent experience. We showed previously (ISFN 2005) that the handwriting of words in a well-practiced script immediately after training on the finger opposition sequence (FOS) task interfered with the expected delayed gains. Here we compared the interference, on FOS learning in the left hand and in the righthand, exerted by handwriting using the right hand. We also tested whether the level of experience in the FOS and in the handwriting affected the degree of interference. *Method*. Right handed participants (*n* = 33) underwent a baseline (no interference) and an interference training condition, one week apart. The two study phases were identical except that in the interference phase, participants were given the interference task (handwriting, dominant hand, 16 repetitions of two words) immediately after FOS training. *Exp 1:* participants trained the FOS with either the right or the left hand. Interference consisted of common Hebrew words. *Exp 2:* the participants trained the FOS with their right hand. Interference consisted of common Hebrew words or nonwords written in Hebrew letters.*Results*. *Exp 1:* interference occurred only when practice in the FOS and the subsequent handwriting were performed by the same hand. *Exp 2:* unlike writing common words, there was no interference by handwriting of non-words, although both tasks were executed with the same hand. *Conclusions*. Interference occurs when there is a critical neuronal overlap between the representations of two tasks. The extent of overlap between the representations is related, at least in part, to (a) lateralization, that is, the execution of the two tasks with the same/different effector, and (b) the degree of training in both tasks.

# Abstract: THE DEVELOPMENT OF SENSITIVITY TO DYNAMIC AUDITORY STIMULI IN SCHOOL-AGE CHILDREN

**Authors:** K. Banai, A. T. Sabin, N. Kraus, B. A. Wright

**Affiliations:** ^1^Department of Communication Sciences and Disorders, Northwestern University, USA; ^2^Northwestern University Institute of Neuroscience, USA

Adequate sensitivity to dynamic auditory cues such as amplitude modulation (AM) and frequency modulation (FM) is important for normal speech perception and language skills. However, relatively little is known about the normal development of AM and FMsensitivity in school-age children and adolescents. In the current experiment we evaluated the developmental time course of sensitivity to sinusoidal AM(carrier: 1 s white-noise; modulation rates: 8, 64, and 125 Hz) and FM (carrier: 1.5 s 1-kHz; modulation rates: 2, 20, and 240 Hz) across children aged 8–10 and 11–12-year/old and adults. For FM, both average performance (mean) and performance consistency (within-listener standard deviation) were adult like in the 8–10-year/old for all three rates. On the other hand, for AM, average performance was still not adultlike in the 10–12-year/old for any rate. Poorer AM sensitivity in children could not, however, be attributed to poorer performance consistency in this group since standard deviation stayed stable across the three age groups. The different developmental time courses of average sensitivity and performance consistency for AM suggest that these two measures of performance may be governed by separate mechanisms. Furthermore, the presence of developmental improvement in AM but not FM sensitivity suggests that, contrary to theories based on data obtained from adults, at least during development, sensitivity to AM and FM may be limited by different neuronal bottlenecks thatmature at different rates.

*Supported by NIH/NIDCD.*

# Abstract: IMMUNOSUPPRESSIVE EFFECT OF SOME SSRI'S: THE EVIDENCE OF A PROAPOPTOTIC MECHANISM AND INVOLVEMENT OF THEMAPK PATHWAY

**Authors:** M. Bar, M. Taler, A. Zolkov, A. Weizman, I. Gil-Ad

**Affiliations:** Laboratory of Biological Psychiatry, FMRC Beilinson Medical Center, Rabin Campus, Tel Aviv University, Tel Aviv 69978, Israel

Recent evidence in our laboratory has shown that some antidepressants (sertraline, paroxetine, and clomipramine) affect the immune system and potently inhibit basal and mitogen induced lymphocyte proliferation of rodents and human. Few studies have demonstrated that the active antidepressants also reduced acute and chronic experimental inflammation. The aims of this study were to evaluate the effect of sertraline, paroxetine, and clomipramine on the proliferation of mitogen activated rat/human lymphocytes, and on the molecular and genetic mechanism underlying the inhibitory effect of the drugs. All drugs induced a dose-dependent decrease in the proliferation of ConA/PHA activated lymphocytes with IC50 levels (range from 1–5 *μ*md/L). The potency scale was sertraline > paroxetine > clomipramine. In parallel, we assessed the effect of the antidepressants on TH1/2 cytokine secretion and found a decrease in TNF*α* secretion and a small increase in IL10 secretion. All agents caused activation of the MAPK pathway with a rapid increase (2 hours) in C-Jun, p-c-Jun, and pERK. In addition, we found a decrease in Bcl2 and Cox2 expression after 24 hours. Sertraline and paroxetine induced an increase in caspase 3 specific activity. Flow cytometric analysis of propidium iodide stained rat splenocytes showed that sertraline and paroxetine induced a G1/G0 arrest accompanied by a decrease in the S phase. Moreover the antidepressants induced a dose-dependent increase in DNA fragmentation (16.7% in controls versus 45.5% in sertraline, 10 *μ*md/L), suggesting the activation of an apoptotic mechanism. These data support a potential immunosuppressive effect for some SSRIs, mainly sertraline and paroxetine, through an antiproliferative effect on activated lymphocytes.

# Abstract: AMINOINDAN, THE MAJOR METABOLITE OF RASAGILINE, EXERTS NEUROPROTECTIVE AND ANTIOXIDANT PROPERTIES IN VITRO

**Authors:** O. Bar Am, T. Amit, M. B. H. Youdim

**Affiliations:** Eve Topf Centre, Technion–Israel Institute of Technology, Haifa 32000, Israel

The anti-Parkinson's selective irreversible monoamine oxidase-B (MAO-B) inhibitor drug, rasagiline, has been shown to possess neuroprotective activities in cell culture and in vivo models. Preliminary studies have indicated that the major metabolite of rasagiline, 1-(R)-1, aminoindan may have neuroprotective activity. In this study, we assessed the neuroprotective properties of 1-(R)-aminoindan, using a cytotoxic model of human neuroblastoma SK-N-SH cells in high-density culture-induced neuronal death. In this model, aminoindan (0.1–1 *f* ý M) significantly reduced the levels of the early apoptosis-associated phosphorylated protein, H2A.X (ser 139), and decreased the cleavage of caspase 9 and caspase 3, while increasing the antiapoptotic proteins, Bcl-2 and Bcl-xl. The neuroprotective effect was prevented by the protein kinase C (PKC) inhibitor, GF109203X, indicating the involvement of PKC in aminoindan-induced cell survival. Indeed, aminoindan markedly elevated the levels of pPKC(pan) and specifically the prosurvival PKC isoform, PKC*f*Õ . Similar neuroprotective effects were observed with hydroxyaminoindan, a metabolite of the anti-Alzheimer's drug, Ladostigil (TV3326) [(N-propargyl-(3R) aminoindan- 5yl)-ethyl methyl carbamate]. In addition, aminoindan and hydroxyaminoindan were found to protect PC-12 cells against the parkinsonian neurotoxin, 6-hydroxydopamine (6-OHDA), suggesting that the neuroprotective properties may also result from its antioxidant properties. These findings suggest that both, the parent compounds and their metabolites may contribute to the overall neuroprotective activity.

*Supported by Teva Pharmaceutical Co, Israel, and Technion Research and Development.*

# Abstract: HEBBIAN SPIKE-TIMING-DEPENDENT PLASTICITY IN MULTIPLE DENDRITIC SITES

**Authors:** L. Bar Ilan, I. Segev

**Affiliations:** ^1^Department of Neurobiology, The Hebrew University, Jerusalem 91010, Israel; ^2^Interdisciplinary Center for Neural Computation, Jerusalem, Israel

Could multiple competing “Hebbian synaptic teachers” coexist at different dendritic sites while retaining the efficacy of their corresponding “synaptic pupils”? This question was explored using a model of layer 5 pyramidal neuron. In this model, synaptic plasticity in the apical tree is dominated by local dendritic Ca spikes via Hebbian spiketiming- dependent plasticity (STDP), whereas plasticity of peri-somatic and basal synapses is dominated by an identical Hebbian STDP rule with axo-somatic Na spikes. The somatic and the dendritic “synaptic teachers” interact with each other via back-propagating action potentials (BPAPs) from the soma to the dendrites that facilitate the generation of dendritic Ca spikes (BAC firing). In turn, the BAC firing encourages generation of Na spike bursts at the axosomatic region. We show that there exists a wide range of synaptic, excitability, and input parameters whereby the percentage of strong and weak synapses over the whole dendritic tree is rather equal. In other parameter regimes, either deneritic synapses survive and proximal synapses die-out, or vice versa. We conclude that several local dendritic “teachers” for synaptic plasticity may peacefully coexist in the same neuron, thus enabling a fine local control of synaptic efficacy using a Hebbian STDP rule.

# Abstract: SCOPOLAMINE INDUCES DISRUPTION OF LATENT INHIBITION WHICH IS PREVENTED BY NEUROLEPTICS AND AN ACETYLCHOLINESTERASE INHIBITOR: A CHOLINERGIC MODEL OF SCHIZOPHRENIA

**Authors:** S. Barak, I. Weiner

**Affiliations:** Department of Psychology, Tel Aviv University, Tel Aviv 69978, Israel

The fact that muscarinic antagonists may evoke a psychotic state and attentional deficits (“antimuscarinic psychosis”) along with findings of cholinergic abnormalities in schizophrenia have led to a growing interest in the involvement of the cholinergic system in this disorder. Latent inhibition (LI) is a cross-species phenomenon manifested as a poorer conditioning of a stimulus seen when the stage of conditioning is preceded by a stage of repeated nonreinforced preexposure to that stimulus, and is considered to index the capacity to in-attend irrelevant stimuli. Amphetamineinduced LI disruption and its reversal by antipsychotic drugs (APDs) is a well-established model of positive symptoms of schizophrenia. Here we tested whether the muscarinic antagonist scopolamine would disrupt LI and whether such disruption would be reversed by APDs and by the acetylcholinesterase inhibitor physostigmine. The results showed that scopolamine at doses of 0.15 and 0.5 mg/kg disrupted LI, and that this effect was due to the action of the drug in the preexposure stage, suggesting a role of muscarinic transmission in attentional processes underlying LI. Both the typical and the atypical APDs, haloperidol and clozapine, reversed scopolamine-induced LI disruption when given in conditioning or in both stages, but not in preexposure, indicating that the mechanism of antipsychotic action in this model is independent of the mechanism of action of the propsychotic drug. Scopolamine-induced LI disruption was reversed by physostigmine (0.05 and 0.15 mg/kg) which was ineffective in reversing amphetamine-induced LI disruption. This points to distinct mechanisms underlying LI disruption by scopolamine and amphetamine, and by corollary, scopolamine- and amphetamine-induced psychoses. We propose scopolamine-induced LI disruption as a model of cholinergic-related positive symptoms in schizophrenia.

# Abstract: PERSISTENT ACTIVITY IN NETWORKS WITH DYNAMIC SYNAPSES

**Authors:** O. Barak, M. Tsodyks

**Affiliations:** Department of Neurobiology, Weizmann Institute of Science, Rehovot 76100, Israel

Persistent activity states (attractors), observed in neocortex after the removal of a sensory stimulus, are believed to be the neuronal basis of working memory. One of the possible mechanisms that can sustain persistent activity is recurrent excitation mediated by intracortical synaptic connections. A recent experimental study revealed that connections between pyramidal cells in prefrontal cortex exhibit various degrees of synaptic depression and facilitation. Here we analyze the effect of synaptic dynamics on the emergence and persistence of attractor states of interconnected neural networks. We show that different combinations of synaptic depression and facilitation result in qualitatively different network dynamics with respect to the emergence of the attractor states. This analysis raises a possibility that attractors could represent time-dependent stimuli as well as static ones.

# Abstract: TYROSINE PHOSPHORYLATION OF THE NMDA RECEPTOR IN THE INSULAR CORTEX IS REQUIRED FOR NOVEL TASTE ACQUISITION

**Authors:** L. Barki-Harrington, A. Elkobi, K. Rosenblum

**Affiliations:** Department of Neurobiology and Ethology, Center for Brain and Behavior, University of Haifa, Haifa 31905, Israel

While tyrosine phosphorylation of post-synaptic density proteins is implicated in synaptic plasticity, its role in learning and memory is less clear. Previously we identified the NR2B subunit of the NMDAR as the major target of tyrosine phosphorylation in the taste cortex following taste learning. Here we hypothesize that the correlative increase in tyrosine phosphorylation modulates synaptic function and is essential for novel taste acquisition. Total lysates of insular cortices from rats exposed to either water (familiar taste) or saccharin (unfamiliar taste) were immunoprecipitated with anti-pY, and the resulting immune-complexes were probed for levels of NR2B, NR2A, NR1, and for additional proteins in the pY complex. Exposure of animals to saccharin significantly increased the phosphorylation of the fyn kinase target Y-1472 on NR2B. The precipitated pY complex also showed an elevation in levels of NR2A, NR2B, NR1, and in PSD 95. To test whether these changes are localized to the synapse, we analyzed the synatosomal fractions of the insular cortex from water and saccharin treated rats. Surprisingly, one hour after exposure to the novel taste, there was a dramatic decrease in the levels of NR2A, NR2B, and NR1 in the synaptic area. These changes were accompanied by a similar reduction in the levels of AMPA GluR1 subunit, suggesting weakening of synaptic strength. Finally, a bilateral, local infusion of the general tyrosine kinase inhibitor genistein to the insular cortex of rats exposed to saccharin caused a significant decrease in the levels of tyrosine phosphorylated NR2B. In accordance, a single microinjection of genistein to the insular cortex immediately prior to the preexposure period in the latent inhibition paradigm significantly attenuated taste memory. Together our data indicate a specific requirement for tyrosine phosphorylation in the insular cortex during the acquisition of novel taste.

# Abstract: NANOSCALE GEOGRAPHY OF SNARE COMPLEXES OFFERS A NOVEL MECHANISTIC INSIGHTS IN THE REGULATION OF EXOCYTOSIS

**Authors:** D. Bar-On, A. Mezer, G. Nudelman, U. Winter, T. Lang, D. Fasshauer, E. Nachliel, M. Gutman, U. Ashery

**Affiliations:** ^1^Department of Neurobiochemistry, Tel Aviv University, Tel Aviv 69978, Israel; ^2^Laser Laboratory for Fast Reactions in Biology, Department of Biochemistry, Tel Aviv University, Tel Aviv 69978, Israel; ^3^Faculty of Life Sciences, Bar-Ilan University, Israel; ^4^Department of Neurobiology, Max Planck Institute for Biophysical Chemistry, Goettingen, Germany

Syntaxin-1A, SNAP-25, and synaptobrevin/VAMP-II form the SNARE complex, which is crucial for vesicle fusion. Syntaxin-1A is concentrated in cholesterol-rich clusters at the plasma membrane (PM) that might function as docking and fusion sites for exocytosis. The formation of the nonproductive cis-SNARE complexes between PM-SNAREs counteracts the formation of trans-SNARE complexes between the PM syntaxin, SNAP-25, and vesicular SNARE, synaptobrevin. The formation of SNARE complexes depends on the local SNARE-availability, which might be determined by the cluster dynamics and the disassembly of cis-complexes by NSF and á-SNAP. The goal of this research is to examine the SNARE nonhomogenous distribution and kinetics on the PM at the nonoscale resolution in various advanced molecular, microscopical, and computational methods. Assembly and disassembly of PM SNARE cis complexes were investigated, using the PM sheets preparation. The rate of SNARE complexes assembly was found to be only slightly affected by cholesterol depletion, which should lead to disintegration of the syntaxin clusters. To learn more about the dynamics of the SANRE proteins on the PM, we built a Monte Carlo simulation and examined assembly and disassembly of cis complexes, with or without syntaxin domains. We developed a graphic interface that enables to monitor the proteins' movement and complex formation. Consistent with our experimental findings, clustering of syntaxin only slightly slows down the spontaneous formation of cis-SNARE complexes. The dissociation of the cis complex on the PM sheets by the NSF/á-SNAP was found to be much faster, exhibiting a linear dependency on the concentration of NSF and á-SNAP. In summary, the combination of the cell surface simulation and the PM sheets experiments allows to examine the influence of the syntaxin clusters on assembly and disassembly of SNARE complexes with the final goal to understand the biological role of the clusters.

# Abstract: THE IMPACT OF NETWORK ACTIVITY ON LAYER 5 NEOCORTICAL PYRAMIDAL NEURONS FROM THE RAT

**Authors:** D. Bar-Yehuda, A. Korngreen

**Affiliations:** ^1^The Leslie & Susan Gonda Multidisciplinary Brain Research Center, Bar-Ilan University, Israel; ^2^The Mina & Everard Goodman Faculty of Life Sciences, Bar-Ilan University, Israel

In recent years there has been a growing number of researches investigating the dynamics of single neurons. At the same time there has also been a growing interest in the computational aspects of neural networks. There still is, however, a great gap between these two realms. Our research aims to combine the biological and mathematical methodologies by analyzing measurements taken from pyramidal neurons of layer 5 of the somatosensory cortex. These neurons are known to interact with their surrounding network activity. This surrounding network activity, though being measured in vitro, simulates key parameters of actual in vivo activity (ie, spontaneous flow of synaptic input). This activity is generated using innovative techniques alongside the use of wellknown methods (eg, whole-cell patch-clamp). We explore intrinsic mechanisms of information processing in the form of synaptic input, and the influence of background activity on it. Therefore, the focus is on measuring backpropagation within each neuron in various locations along the apical dendrite under different levels of network activity. According to preliminary results the backpropagating action potential has not been changed due to increased background synaptic activity, yet, the calcium spike, generated when backpropagating action potential and distal synaptic input couple, is modulated by that activity.

# Abstract: LENTIVIRAL TRANSDUCTION OF HUMAN BONE MARROW MESENCHYMAL STEM CELLS AS A NOVEL STRATEGY FOR INDUCTION OF DOPAMINERGIC DIFFERENTIATION

**Authors:** R. Barzilay, T. Ben-Zur, S. Bulvik, E. Melamed, D. Offen

**Affiliations:** ^1^Laboratory of Neuroscience, Felsenstein Medical Research Center, Rabin Medical Center, Tel Aviv University, Tel Aviv 69978, Israel; ^2^Departement of Neurology, Rabin Medical Center, Tel Aviv University, Tel Aviv 69978, Israel; ^3^Laniado Medical Center, Natanya, Israel

Adult bone marrow mesenchymal stem cells are known for their capacity to differentiate to bone, fat, and cartilage. Previously, we have shown the efficient induction of these cells to dopaminergic-like cells following exposure to extrinsic signaling molecules. In the current work we study the genes, which might be involved in adult stem cells differentiation to dopaminergic neurons. Our primary objective was to introduce genes that have been reported to be effective in human embryonic stem cells. Using lentiviral expression system in bone marrow stem cells we obtained efficient transduction, as indicated by high expression of green and red fluorescence proteins. Transduction of Lmx1a gene, one of the embryonic transcription factors, demonstrates that its high expression levels was associated with modified expression of other dopaminergic genes, such as Nurr-1. Our results reveal that introduction of genes known to play a role during embryonic development of the dopaminergic system may also facilitate differentiation in adult bone derived stem cells.

# Abstract: EFFECTS OF PREPUBERTAL STRESS EXPOSURE ON ADULT STRESS RESPONSE ARE CORRELATEDWITH CHANGES IN CIRCULATING CORTICOSTERONE AND BRAIN-DERIVED NEUROTROPHIC FACTOR

**Authors:** N. Bazak, N. Kozlovsky, Z. Kaplan, M. A. Matar, G. Richter-Levin, H. Cohen

**Affiliations:** ^1^Ministry of Health Mental Health Center, Anxiety and Stress Research Unit, Ben Gurion University, Beer Sheva 84105, Israel; ^2^Department of Psychology, University of Haifa, Haifa 31905, Israel

Early-life stress produces a cascade of neurobiological events that cause enduring changes in neural plasticity and synaptic efficacy which appear to play pivotal roles in the pathophysiology of posttraumatic stress disorder (PTSD). Brain-derived neurotrophic factor (BDNF) has been implicated in the neurobiological mechanisms of these changes, in interaction with components of the stress-response, such as corticosterone. This study examined the consequences of juvenile stress on behavior at later stages of life in correlation to circulating corticosterone levels, BDNF, and TrkB mRNA expression. The experiments looked at single exposure to predator scent stress as opposed to repeated exposure, early in life, and later on. Behavioral responses were assessed in the elevated plus-maze and the acoustic startle response paradigms. Animals were subsequently sacrificed, and brain areas dissected and analyzed for mRNA BDNF and TrkB levels. The results show that juvenile trauma increases the vulnerability for developing long-term behavioral disruptions, taken to represent posttraumatic stress responses, subsequent to reexposure to the same stressor in adulthood. Juvenile exposure significantly lowered corticosterone levels compared to controls, whereas adulthood exposure caused significant increases. Exposure to both early and later life trauma elicited reduced levels of corticosterone following the initial exposure, which were not raised by reexposure and elicited significant downregulation of mRNA for BDNF in the CA1 subregion of the hippocampus, compared to the other groups. The results suggest that juvenile stress has resounding effects in adulthood reflected in behavioral responses. The concomitant changes in BDNF and corticosterone levels may mediate the changes in neural plasticity and synaptic functioning underlying clinical manifestations of PTSD.

# Abstract: THE INVOLVEMENT OF CORTICAL mTOR IN TASTE MEMORY

**Authors:** K. Belelovsky, K. Rosenblum

**Affiliations:** Department of Neurobiology and Ethology, University of Haifa, Haifa 31905, Israel

The evolutionarily conserved protein kinase mTOR (mammalian target of rapamycin) is a major control device of cell growth and proliferation via the regulation of the protein synthesis machinery. Some in vitro studies suggest a role of the mTOR in learning and synaptic plasticity. However, its role in cortical-dependent learning is not clear. In order to explore the possible involvement of mTOR in corticaldependent learning we study in detail its role in the insular cortex (IC) of rats, subserving novel taste memory formation and consolidation. Microinjection of 10 *f* ýM rapamycin 25 minutes prior to exposure to novel taste attenuates long-term taste memory as defined in the latent inhibition paradigm. Additional biochemical analysis of several downstream targets of mTOR signaling was performed following similar local microinjection to the insular cortex. We observed decreased phosphorylation levels of ribosomal protein kinase S6K1 (Thr 389) and initiation factor 4E (eIF4E) (Ser 209), elevated phosphorylation levels of elongation factor 2 (eEF2) (Thr 56), and a decrease in the total levels of elongation factor 1A (eEF1A). MAPK/ERK2 phosphorylation was not affected by inhibition of mTOR signaling, suggesting that MAPK and mTOR cascades are working in parallel in the taste cortex. These results suggest that mTOR pathway in the insular cortex is involved in the process of novel taste memory formation. Currently, we are further exploring the role of mTOR in the insular cortex during the formation of longterm taste memories.

# Abstract: ApoE4 INDUCES ABETA-MEDIATED APOPTOSIS IN VIVO

**Authors:** H. Belinson, D. M. Michaelson

**Affiliations:** Tel Aviv University, Tel Aviv 69978, Israel

The apoE4 isoform of apolipoprotein E, the major genetic risk factor of Alzheimer's disease (AD), is associated with increased Abeta deposition. The initial stages of the amyloid cascade play a pivotal role in AD. The objective of this work was to investigate the pathological effects of the initial aggregation stages of Abeta and the effects thereon of apoE4. This was investigated by studying the effects of the apoE genotype on Abeta-mediated cellular pathology in apoE transgenic mice following prolonged inhibition of neprilysin. Under basal conditions the density of hippocampal neurons of transgenic mice which express either apoE4 or, the AD benign allele, apoE3 was similar. In contrast, elevation of brain Abeta levels by inhibition of neprilysin resulted in a significant decrease in the density of CA1 neurons of the apoE4 mice (∼30%) which reached a plateau at two weeks. Whereas the CA1 neurons of the apoE3 mice were not affected during the first 2 weeks and were only partly reduced by 4 weeks following initiation of the treatment. This effect was specific to CA1. Furthermore, the CA1 neurons of the apoE4 mice contained significant levels of activated caspase 3 at 2 weeks and 4 weeks; whereas this activity was detected at the apoE3 mice only at 4 weeks. Additionalmeasurements of inflammatory activation revealed progressive microgliosis, which was markedly more pronounced in the apoE4 than in the apoE3 mice, and similar but low levels of astrogliosis in both mice. These results show that apoE4 stimulates Abeta-mediated apoptosis of CA1 neurons in vivo and suggest that this effect may be mediated via microglia. This effect is apparent within a week, during which insoluble Abeta is scarce, and does not colocalize with the insoluble Abeta deposits. This suggests that the proapoptotic effects of apoE4 are mediated by soluble Abeta oligomers.

# Abstract: G-PROTEIN-COUPLED RECEPTORS ARE VOLTAGE SENSORS: MOVEMENT OF “GATING CHARGE” IS COUPLED TO LIGAND BINDING

**Authors:** Y. Ben-Chaim, B. Chanda, N. Dascal, F. Bezanilla, I. Parnas, H. Parnas

**Affiliations:** ^1^Department of Neurobiology, The Hebrew University, Jerusalem 91010, Israel; ^2^Department of Physiology, University of Wisconson, Madison, WI, USA; ^3^Department of Physiology and Pharmacology, Tel Aviv University, Tel Aviv 69978, Israel; ^4^Institute for Molecular Pediatric Sciences, University of Chicago, Chicago, IL 60637, USA

G-protein-coupled receptors (GPCRs) play a crucial role in most signal transduction processes. Binding of agonist is considered the only mechanism for activation of a GPCR. Yet, in a number of experimental systems, changes in membrane potential affected the agonist efficacy in activating the GPCR. In all these cases the process and protein which is voltage sensitive had not been identified. Even though GPCRs span the cell membrane they are not considered to be voltage sensitive. Recently it was found in our laboratory that the affinity for the agonist of two muscarinic GPCRs is affected by membrane potential. It was unclear, however, whether the GPCRs themselves are voltage sensitive. We now show that the m2 and m1 muscarinic receptors display charge movement, similar to gating currents of voltagegated channels. The “gating current”-voltage relationship of the m2R correlates well with the change in affinity of the receptor for acetylcholine. The 3rd intracellular loop of m2R and m1R seems to link between the region in the molecule that serves as a voltage sensor and the region that determines the affinity of the receptor. These findings show that both agonist concentration and membrane potential modulate the activity of GPCRs.

# Abstract: A POSSIBLE ROLE FOR ALPHA-SYNUCLEIN IN MEMBRANE TRAFFICKING MEDIATED BYMEMBRANE ENRICHMENT IN PUFA

**Authors:** T. Ben-Gedalya, Y. Altschuler, D. J. Selkoe, R. Sharon

**Affiliations:** ^1^Department of Cellular Biochemistry and Human Genetics, The Hebrew University, Ein Kerem, Jerusalem 91010, Israel; ^2^Department of Pharmacology, The Hebrew University, Ein Kerem, Jerusalem 91010, Israel; ^3^Center for Neurologic Diseases, Harvard Institutes of Medicine, Boston, MA 02115, USA

The neuronal cytoplasmic protein, alpha-Synuclein (aS), has been implicated in the pathogenesis of Parkinson's disease (PD) at both the genetic and cytopathological levels. aS, a highly conserved presynaptic protein is a major component of Lewy bodies—interneuronal inclusions that are the pathological hallmark of Parkinson's disease. Despite extensive study, little is known about the normal function or pathobiology of aS. Previously we reported that aS interacts with polyunsaturated fatty acids (PUFA) as part of its physiological function. We have also shown that aS increases membrane fluidity by enrichment of membranes specifically with PUFA. Our working hypothesis is that aS affects membrane trafficking through its effect on membrane fluidity. Using incorporation of FM 1–43 dye by living MES neurons, we detect enhanced accumulation of newly formed endosomes by either supplementing the conditioned medium with certain PUFAs or by overexpressing aS in the presence of standard medium. A synergistic enhancement of membrane trafficking is observed when PUFA treatment is combined with aS overexpression. The longer and more unsaturated the FA is, the more potent the enhancement of membrane trafficking is. Moreover, the PD-causing A53T mutant form of aS has a stronger effect on membrane trafficking than wt aS. We now identified an endocytic mechanism that is activated by aS-mediated enrichment of membranes with PUFA. aS and PUFA synergistically induced transferrin endocytosis. Transferrin endocytosis by its receptor is known to be mediated by receptor-mediated endocytosis (RME). This result indicates that clathrin-mediated endocytosis (CME) and (RME) are both induced by PUFAs and aS. To further verify this observation we used a dominant negative form of dynamin (K44A) and a shRNA against clathrin heavy chain. We conclude that aS activates endocytosis through its effect on membrane PUFA composition.

# Abstract: LYSOPHOSPHOLIPIDS MODULATE L-TYPE AND T-TYPE CALCIUM CHANNEL CURRENTS IN PITUITARY CELLS

**Authors:** G. Ben-Zeev, Nussinovitch

**Affiliations:** Department of Anatomy and Cell Biology, The Hebrew University Medical School, Jerusalem 91120, Israel

Lysophospholipids (LPLs) are lipophilic molecules consisting of a hydrophilic head and a hydrophobic tail. It was suggested that partition of these molecules into the phospholipid bilayer alters membrane tension and thereby affects the gating of mechanosensitive ion channels. LPLs were defined by their shapes as cones, inverted-cones, or cylinders. It was suggested that partitioning of inverted-cones into the outer leaflet of the phospholipid bilayer forms convex membrane structures mimicking membrane compressionor cell shrinkage. In this study we examined whether an inverted-cone-shaped molecule, Lysophosphatidylchloline (LPC), modulates L-type, and T-type calcium channel currents (IL and IT) in pituitary cells. Our main findings may be summarized as follows. (1) LPC (3–30 micromolar) suppressed both IL and IT in a dose-dependent manner. (2) This suppression of IL and IT was irreversible. Full reversibility was observed only after washout of LPC with BSA (0.5mg/ml). (3) The effects of LPC on IL and IT were differential. The suppression of IT was more prominent than the suppression of IL. In addition, the suppression of IT started after a short delay of several seconds whereas the suppression of IL started after a long delay of 50–100 seconds. (4) The suppression of IL was voltage dependent with a stronger suppression at more negative potentials. (5) The negatively charged inverted-cone-shaped lysophosphatidylinositol (LPI), but not the cylinder-shaped phosphatidylcholine (PC), had similar effects as those produced by LPC on calcium currents. In summary, our results show that lipophilic molecules in the shape of inverted cones suppress calcium currents in pituitary cells. It is possible that partition of these molecules into the plasma membrane alters membrane curvature and tension, thereby modulating calcium influx in pituitary cells.

*Supported by the Israel Science Foundation Grant 826/04.*

# Abstract: POLYMORPHISMS IN THE DOPAMINE D4 RECEPTOR GENE (DRD4) CONTRIBUTE TO INDIVIDUAL DIFFERENCES IN HUMAN SEXUAL BEHAVIOR: DESIRE, AROUSAL, AND SEXUAL FUNCTION

**Authors:** T. Ben-Zion, Y. Raz, R. Tessler, L. Cohen, E. Lerer, R. Bachner-Melman, I. Gritsenko, L. Nemanov, A. Zohar, J. Benjamin, R. P. Ebstein

**Affiliations:** ^1^Faculty of Health Sciences, Ben Gurion University of the Negev, Beer Sheva 84105, Israel; ^2^Department of Psychology, The Hebrew University, Mount Scopus, Jerusalem 91010, Israel; ^3^Department of Cellular Biochemistry and Human Genetics, The Hebrew University, Jerusalem 91010, Israel; ^4^S. Herzog Memorial Hospital, Jerusalem, Israel; ^5^Department of Psychology, Behavioral Sciences, Ruppin Academic Center, Emek Hefer, Israel

Although there is some evidence from twin studies that individual differences in sexual behavior are heritable, little is known about the specific molecular genetic design of human sexuality. Recently, a specific dopamine D4 receptor (DRD4) agonist was shown in rats to induce penile erection through a central mechanism. These findings prompted us to examine possible association between the well-characterized DRD4 gene and core phenotypes of human sexual behavior that included desire, arousal, and function in a group of 148 nonclinical university students. Association between five DRD4 polymorphisms (3 promoter SNPs, a 120 bp promoter region tandem duplication, and the exon3 repeat region) and self-report questions regarding desire, arousal, and function were tested using robust family-based methods, FBAT, and UNPHASED. The DRD4 exon3most common D4.4 repeat is negatively associated with desire and function scores. Carriers of the D4.4 repeat display less desire (*Z* = −2.02, *P* = .04) and report more sexual dysfunction (*Z* = −2.02, *P* = .04). The single most frequent (18%) five locus haplotype of the five DRD4 polymorphisms (C-521T&C-616G & A-809G & EXON3 & 120 bp tandem duplication) genotyped was CG- G-4-2 and was negatively associated with desire (*Z* = −3.19, *P* = .001), arousal (*Z* = −2.95, *P* = .003), and function (*Z* = −3.19, *p* = 0.001). The current results are consistent with animal studies that show a role for dopamine and specifically the DRD4 receptor in sexual behavior and suggest that one pathway by which individual variation in human desire, arousal, and function is mediated is based on allelic variants coding for differences in DRD4 receptor gene expression and protein concentrations in key brain areas.

# Abstract: OBJECT RECOGNITION MODEL INSPIRED BY THE VISUAL CORTEX

**Authors:** E. Berkovich, M. Gur, H. Pratt

**Affiliations:** ^1^Department of Biomedical Engineering, Technion–Israel Institute of Technology, Technion City, Haifa 32000, Israel; ^2^Evoked Potentials Laboratory, Technion–Israel Institute of Technology, Technion City, Haifa 32000, Israel

Although there has been a remarkable progress in pattern recognition algorithms in recent years, these algorithms still do not compare with the human ability for object recognition. This recognition ability, performed intuitively and spontaneously, is one of the main functions of the human visual system. Using various perceptual cues and multiple sensory inputs we can identify a variety of objects instantly and effortlessly. Unlike computer vision systems, the human visual system is indifferent to changes in illumination, pose, and scale. The neurophysiological procedures underlying the recognition process are complex and use features such as shape, color, and orientation. The primary visual cortex (V1) generates a unique representation of these features that lead, eventually, to the perceived object. Hence, in this research, a biologically motivated recognition model is developed. Lowlevel features of the image are extracted by appropriate filters, in a manner similar to the human visual system, in which visual information is sampled by the retina, passes through the lateral geniculate nucleus (LGN) to the primary visual cortex where low-level image features are represented. Additionally, image intensities are converted to neuronal spike rates in accordance with the visual system. The resulting spike trains are fed into a recognition module which is also based on biological concepts: its first stage is a biological neural network which implements a neural microcircuit model, and its output is fed into a standard artificial neural network for final classification. The current experimental setup produces good categorization results (eg, boats, cars, faces), and the use of a biological neural network improves the recognition performance. When compared to previous biological models, the proposed recognition model yields improved recognition results while being more consistent with physiological mechanisms.

# Abstract: THE INFLUENCE OF PHONOLOGICAL TRANSPARENCY ON READING

**Authors:** A. S. Bick, G. Goelman, R. Frost

**Affiliations:** ^1^ICNC, The Hebrew University, Jerusalem 91010, Israel; ^2^Medical Biophysics, Hadassah Hebrew University Hospital, Jerusalem, Israel; ^3^Department of Psychology, The Hebrew University, Jerusalem 91010, Israel

Two possible routes could be used when reading—a direct grapheme-to-lexicon route or a route in which the phonology of words is first assembled via grapheme-to-phoneme translation rules. Cognitive studies show that the choice of route is influenced by proficiency, task, and word's phonological transparency. Different languages differ in their extent of phonological transparency. Hebrew has different levels of transparency; pointed words are fully transparent, unpointed word's transparency varies according to their degree of freedom (DF)—words with more “vowel letters” are more transparent (low DF) and words with less are phonologically ambiguous (high DF). In this fMRI study, subjects were requested to covertly name words presented in a block design. The first experiments included pointed and unpointed words, and in the second experiment two levels of DF were implemented. In both experiments two levels of word frequency were used. Areas activated differentially by the word's pointedness were found in the occipital cortex, in the occipotemporal region, and in the parietal cortex. Response to words with different DF was tested in these areas. Volumes in the parietal cortex were found to be sensitive to the phonological transparency of words with similar activation patterns in both experiments. Activation in this volume was significantly higher when the phonological information was present regardless of the frequency of the words. Furthermore this volume was activated significantly higher by nonwords rather than words. Further analysis showed that this difference was significant only for high DF words. These results show that the parietal region is involved in graphemeto- phoneme conversion. This process is used for nonword and when the word's phonology is transparent. The reading system recruits the different routes according to the stimuli in order to obtain efficient reading. Knowledge of these processes may assist our understanding of reading disabilities.

*Supported by the Center for Complexity Science. Scans were performed in Hadassah Medical Center.*

# Abstract: NEURAL CORRELATES OF MORPHOLOGICAL PROCESSES IN HEBREW

**Authors:** A. S. Bick, R. Frost, G. Goelman

**Affiliations:** ^1^ICNC, The Hebrew University, Jerusalem 91010, Israel; ^2^Medical Biophysics, Hadassah Hebrew University Hospital, Jerusalem, Israel; ^3^Department of Psychology, The Hebrew University, Jerusalem 91010, Israel

A dispute exists as to whether morphology is a discrete and independent element of lexical structure or whether it simply reflects a fine-tuning of the system to the statistical correlation that exists among the orthographic and semantic properties of words. Imaging studies in English failed to show unequivocal morphological activation that is independent of semantic or orthographic activation [1]. Cognitive research in Hebrew has revealed that morphological decomposition is an important component of print processing [2]. In Hebrew, morphological relatedness does not necessarily induce a clear semantic relatedness, thus, Hebrew provides a unique opportunity to investigate the neural substrates of morphological processing. In this fMRI study participants were required to perform judgment tasks of morphological relatedness, semantic relatedness, rhyming, and orthographic similarity. Half of the morphologically related words were semantically related and half were semantically unrelated. This design was chosen to induce explicitmorphological processing. A line pattern judgment task was used as a control. Words were presented in block design. We identified two locations involved in morphological processing: the lMFG and lIPS. Comparing locations of morphologically related activation to the locations of semantic and orthographic related activation, we found that the areas neighbored but only partially overlapped. Both morphological conditions displayed a similar pattern of activation. These results coincide with the behavioral data previously obtained in Hebrew, demonstrating the important role of morphological processing in reading, and suggest that morphological analysis is an independent process of visual word recognition. Since our results show activation that is independent of the semantic overlap between words, the possibility that this activation results from semantic factors seems unlikely.

*Supported by the Center for Complexity Science. Scans were performed at Wohl Institute, Sourasky Medical Center.*

## References

[1] Devlin JT, Jamison HL, Matthews PM, Gonnerman LM (2004). Morphology and the internal structure of words. *Proceedings of the National Academy of Sciences of the United States of America*.

[2] Frost (2000). *Journal für Entwicklungspolitik*.

# Abstract: TIME- AND REGION-DEPENDENT REDUCTION OF BRAIN NMDA RECEPTOR AVAILABILITY FOLLOWING A FLUROTHYL-INDUCED SEIZURE IN RATS

**Authors:** A. Biegon, D. Anschel, L. Ephraty, M. Nawrocky, R. Tzabari

**Affiliations:** ^1^Brookhaven National Laboratory, PO Box 5000, Upton, NY 11973-5000, USA; ^2^J. Sagol Neuroscience Center, Sheba, Israel

Acute seizures and chronic epilepsy are known to be associated with deficits in cognitive function. NMDA receptor activation in the hippocampus is considered to be a key step in memory formation. A loss of function of these receptors may mediate such deficits. The goal of the present study was to evaluate the effects of a single seizure on NMDA receptor function in the rat hippocampus, using quantitative in vitro autoradiography. Rats were induced to have a single generalized tonic-clonic seizure by Flurothyl (2,2,2-Trifluoroethyl ether) inhalation. Groups of 4-5 rats were sacrificed at various time points (15 min to 7 days) after the seizure and their brains processed for autoradiography. An intact control group was included for comparison. Coronal cryostat sections at the level of the hippocampus were incubated with 5 nM of the use dependent NMDA antagonist 3H-MK801; without rinsing or addition of glutamate or glycine. Nonspecific binding was assessed on consecutive sections incubated with a large excess (100 microM) unlabeled MK801. Washed and dried sections were scanned using a beta imager. Regional analysis demonstrated a statistically significant, 20%– 25% transient reduction in [3H]MK801 specific binding in hippocampus (dorsal and ventral), entorhinal cortex and temporal cortex 1 hour after the seizure. No reductions were observed in the striatum and substantia nigra. Specific dorsal hippocampal subfields (CA1 and dentate gyrus) showed a more sustained reduction, which was still significant 4 hours after the seizure. MK801 binding in all brain regions investigated was similar to control levels by 8 hours after the seizure and remained unchanged up to a week later. These results show that a single seizure can cause a significant though transient reduction in hippocampal, entorhinal, and temporal cortical NMDA receptor function; which can explain the well documented amnesic effects observed acutely in the aftermath of seizures in humans.

*Supported by DOE-OBER.*

# Abstract: MUSIC AND MUSCLE RELAXATION THERAPIES AS TREATMENT FOR INSOMNIA IN PTSD PATIENTS

**Authors:** M. Blanaru, L. Ben-Oriel, E. Yehezkeli, R. Shornik, T. Rotem, N. Ziv, Z. Arnon, I. Kremer, I. Haimov

**Affiliations:** ^1^Psychiatric Department, Emek Medical Center, Afula, Israel; ^2^Department of Behavioral Sciences, The Max Stern Academic College of Emek Yezreel, Emek Yezreel 19300, Israel

Post-traumatic stress disorder (PTSD) is a psychiatric disorder characterized by pathological anxiety that usually occurs after an individual experiences or witnesses severe trauma that constitutes a threat to the physical integrity or life of the individual or of another person. Disturbed sleep is a common complaint among PTSD patients and has even been referred to as the “hallmark” of PTSD. Both the safety and the effectiveness of sleeping pills for treatment of insomnia in PTSD patients are questionable. Daytime carryover effects observed with longer-acting sleep medications are likely to produce additional, potentially serious decrements in daytime function among PTSD patients. Thus, emphasis should be placed on nonpharmacological interventions to improve sleep efficiency among these patients. Thus, in the present study we examined the effects of music and muscle relaxation therapies as treatment for insomnia in PTSD patients. Thirteen PTSD patients participated in this randomized controlled trail: 8 males and 5 females, mean age 45.7 years, SD = 11.4. All of the participants suffered from insomnia and exhibited actigraphically confirmed decreases in sleep efficiency. Analysis revealed a significant increase in sleep efficiency following music therapy, compared with baseline. The results imply the beneficial effect of Music Therapy compared to muscle relaxation therapy as treatment for insomnia in PTSD patients.

# Abstract: FRONTAL ABNORMALITIES IN FIRST-EPISODE SCHIZOPHRENIA: EVIDENCE FROM fMRI AND HIGH B VALUE

**Authors:** M. Bleich-Cohen, R. Strous, A. Mendehlson, Y. Assaf, T. Hendler

**Affiliations:** ^1^Functional Brain Imaging Unit, Wohl Institute for Advanced Imaging, Tel Aviv Sourasky Medical Center, Tel Aviv 64239, Israel; ^2^Levi-Edersheim-Gitter Institute for Human Brain Mapping, Tel Aviv University, PO Box 39040, Tel Aviv 69978, Israel; ^3^Beer Yaakov Mental Health Center, Tel Aviv University, Beer Yaakov 70350, Israel

*Introduction*. Accumulating evidence is pointing to structural and functional impairments in the frontal lobe in schizophrenia. Though, it is not clear what the relation between the two levels of disturbance is. Advanced MRI allows the combination of structural and functional measurements in the same subject. *Objective*. The current study focused on first-episode schizophrenia patients in order to identify possible early onset brain pathology related to the frontal lobe. We used DWI to evaluate WM integrity and organization. In addition, fMRI was applied to measure brain response to language task which allowed us to examine functional asymmetry—a principle neurodevelopmental organization in the healthy brain.*Method*. Twelve first-episode schizophrenia patients (ages 21–45; 7M, 5F) and 12 matched healthy controls (ages 23–50; 7M, 5F) were scanned in a 1.5T GE scanner. fMRI was acquired during auditory verb generation and passive music listening tasks. DWI was based on high b-value approach. *Results*. fMRI revealed less lateralization in frontal language-related regions such as Broca's area in schizophrenia patients compared to controls (P < .005). Interestingly, this change in lateralization was due to relative increase in activation in the right homologue region. High b-value DWI demonstrated decrease white matter integrity in the left frontal lobe region in schizophrenia patients compared to healthy controls (based on ROI histogram analysis (P < .05)). *Conclusion*. This study suggests that already in the early stage of schizophrenia, MRI detects structural and functional abnormalities in the frontal lobe. The fact that such findings are observed at the first episode supports a possible neurodevelopmental mechanism in schizophrenia. Supported by Institute of Psychobiology (TH, RDS).

*Supported by Institute of Psychobiology (TH, RDS).*

# Abstract: SACRAL PROJECTION INTERNEURONS ARE INVOLVED IN SENSORY ACTIVATION OF LOCOMOTOR PATTERN GENERATORS IN THE NEONATAL MOUSE SPINAL CORD

**Authors:** D. Blivis, G. Z. Mentis, M. J. ó Donovan, A. Lev-Tov

**Affiliations:** ^1^Department of Anatomy and Cell Biology, Medical School, The Hebrew University, Jerusalem 91120, Israel; ^2^Section of Developmental Neurobiology, NINDS, NIH, Bethesda, MD, USA

We have previously shown that stimulation of sacrocaudal afferents (SCA) induced a locomotor-like and tail-moving rhythm in the isolated spinal cord of the neonatal rat and mouse and that the SCA-induced locomotor rhythm depended on synaptic activation of sacral interneurons whose axons cross the cord and project rostrally mainly through the contralateral ventral funiculus (VF). In the present study we identified sacral interneurons with rostral projections through the contralateral VF and examined their association with SCA and nearby motoneurons in the neonatal mouse spinal cord, using double and triple fluorescent labeling techniques and two-photon confocal microscopy. The activity pattern of these VF interneurons was then characterized during SCA-induced rhythmic activity. In vitro retrograde fluorescent labeling of the cut VF at the caudal end of the lumbar region (fluorescein dextran) revealed several groups of S1–S4 interneurons contralateral to the fill. Labeled interneurons were mostly located in the intermediate gray, a few in the dorsal horn, and some in the central canal region and the ventral horn. Some of these interneurons were contacted by anterogradely labeled (Texas red dextran) SCA terminals, suggesting a direct monosynaptic afferent innervation. Optical imaging studies of sacral interneurons loaded with calcium green-dextran through the contralateral VF revealed either tonic or rhythmic firing pattern of these VF interneurons during SCA stimulation. The discharge of VF interneurons was abolished by mu-opioid receptor agonists and restored in the presence of naloxone. The contribution of these sensory pathway interneurons to pattern generation and inter-segmental communication in the mammalian spinal cord should await further studies.

*Supported by US-Israel Binational Science Foundation.*

# Abstract: SYNAPTIC CHANGES IN RAT MUSCLE ACETYLCHOLINESTERASE FOLLOWING EXERCISE

**Authors:** E. Blotnick, L. Anglister

**Affiliations:** Department of Anatomy and Cell Biology, Medical School, The Hebrew University, Jerusalem 91120, Israel

Acetylcholinesterase (AChE) plays amajor role in neuromuscular transmission while its own level depends on neuromuscular activity. Because fast-twitch motor units are recruited with increased motor demand, we examined the regulation of AChE in predominantly fast-twitch rat muscles before and following short strenuous treadmill training. We have previously shown that the total AChE content (per protein) increased in exercised fast- but not slowtwitch muscles. Moreover, training significantly increased the level of the AChE-tetramer (25%–60%) in fast-twitch muscles while the other AChE isoforms in both fast- and slowtwitch muscles remained unchanged. To examine directly whether synaptic AChE is influenced by muscle exercise, EDL muscles of trained and control rats were double-labeled for synaptic acetylcholine receptors (AChRs) with TRITCalpha- bungarotoxin and for AChE with biotin-fasciculin (followed by FITC-streptavidin). Single fibers were isolated and synaptic staining was assessed using confocal fluorescencemicroscopy. Quantitative image analysis revealed a > 2- fold increase in total synaptic AChE intensity and area with a ∼10% elevation in AChE density at neuromuscular junctions of the trained EDL muscles compared to untrained controls. In the same preparations, training did not induce changes in synaptic AChRs. We conclude that the short strenuous training causes a selective increase in synaptic AChE in fast-twitch leg muscles but not in AChRs, and that following exercise, the elevated AChE-tetramer might become a significant component at the endplates of fast-twitch fibers.

*Supported by Israel Science Foundation 685/01 (LA).*

# Abstract: HIGH-ORDER COGNITIVE COMPUTATIONAL POTENCY IN NORMAN AND DYSFUNCTION AT 12 YEARS AND 20 YEARS POSTPARIETAL INJURY

**Authors:** B. Blum, A. Barbul

**Affiliations:** Department of Physiology and Pharmacology, Sackler School of Medicine, Tel Aviv University, Ramat Aviv 69978, Israel

The aim of this study was to explore posterior parietal (IPL) role and internal processes optimizing brain computational processing potentiality, in normal and in parietal injury dysfunction at 12 and 20 years post injury. Perceptual organization was examined for such potency in processing complementary local/global subcategories that differ in viewing modes, but assumed on-line additive to a single basis of decision, and cognate subcategories, additive within cognitive internal process. Pattern recognition psychophysical testing on differentiating multivariate stimuli upright/rotated, varying in acuity demands, display time, display distance, viewing modes showed highly accurate inverse (cc–1) and direct (cc+1) cross-correlation, in normal subjects at P < .05; in parietal injury case at 12 years post injury high cross correlated inverse reciprocities at P < .01, presumably one subcategory attended to, commensurately, at expense of the other, to accommodate attention deficiency. Pairing in direct and in inverse reciprocities proved characteristic of these processing, at various acuity demands and display times, in local and global viewing. In normal subjects a commensurate highly accurate inverse and direct reciprocity were observed; rA versus rB inverse cross-correlation for the full range cc = –0.916 significant at P < .03 and direct cross correlation cc = +0.96 verified in the data atP < .01. Parietal central role is indicated in this computationally-based insight/rationale saliency-determined strategy that allows economic sharing or mutually compensatory processing, with a manifestation of remarkable neuronal computational mathematical exactitude. Decreased reciprocities at 20 years post injury suggested deterioration in computational potency; lack of coherence in face of cooccurrence in impairments allowed envisaging distributed neural network relatable to proximity of mediating networks.

# Abstract: STATINS REDUCE NEUROFIBRILLARY-TANGLES BURDEN (NFT) IN A TRANSGENIC MOUSE MODEL FOR TAUOPATHY AND ALZHEIMER'S DISEASE (AD)

**Authors:** M. Boimel, N. Grigoriadis, A. Lourbopoulos, L. Rozenstein, O. Touloumi, O. Abramsky, H. Rosen Mann

**Affiliations:** ^1^The Department of Neurology, The Agnes Ginges Center, Hadassah University Hospital, Jerusalem, Israel; ^2^AHEPA University Hospital, Thessaloniki, Greece

*Background*. Statin treatment is associated with a reduced risk of developing AD and reduces amyloid deposition in AD mouse models. No data is available regarding the effect on NFT, the aggregates of hyperphosphorylated tau protein which are the best correlate of dementia. *Objective*. To study the effect of statins on NFT pathology in our tgmouse model for NFT (mutant P301S/K257T tau protein). *Methods*. (a) Mice receiving a low cholesterol diet (standard mouse diet) were treated with simvastatin (BBB permeable) for 1 month. (b) Mice receiving a high cholesterol diet (“Western countries' diet”) were treated with atorvastatin (BBB impermeable), for 5 months or with a nonstatin cholesterol lowering agent (cholesterol-absorbance-inhibitor, CAI). Brain sections were examined for NFT burden with Gallyas staining and with AT8 and AT180 immunostainings (anti-202/205 and 231 phosphorylated tau residues), as well as for glia. *Results*. One month treatment with simvastatin reduced the NFT burden by 25% relative to nontreated controls (*P* < .005), as indicated by Gallyas staining, as well as by AT8 and AT180 Abs; with a 26% decrease in microglia (*P* < .001). A larger decrease in NFT burden, 55%, was detected after 5 months with atorvastatin (*P* < .001), accompanied by a 20% reduction in microglia (*P* < .001). Interestingly, mice treated with the nonstatin CAI showed a decrease only in phosphorylated residue 231 (with AT180 Ab: 42%, *P* < .001) but not in 202/205 (with AT8 Ab); with no decrease in glial cells. *Conclusion*. (1) Statins significantly reduce NFT burden. (2) The effect is irrespective of BBB permeability, suggesting a peripheral emanating response. (3) The statin effect is greater than with CAI, and is evident with low cholesterol and with high cholesterol diet. This, together with the decrease in microglia (involved in tau hyperphosphorylation), suggests that the anti-NFT effect of statins is related to their anti-inflammatory properties and not only to cholesterol lowering.

# Abstract: PERCOLATION IN LIVING NEURAL NETWORKS

**Authors:** I. Breskin, J. Soriano, T. Tlusty, E. Moses

**Affiliations:** Physics of Complex Systems, Weizmann Institute of Science, PO Box 26, Rehovot 76100, Israel

We study neural connectivity in cultures of rat hippocampal neurons, and extract statistical properties of the underlying network. The neurons are excited by a global electrical stimulation applied to the entire network through bath electrodes and their response to ramping of the voltage is measured with a calcium sensitive dye. Gradual addition of CNQX blocks the neuro-receptors AMPA and decreases the neural connectivity. The process of disintegration of the network is described in terms of percolation on a graph, yielding a quantification of the connectivity in the network. With no CNQX the network comprises of one big cluster (giant component). Increasing the CNQX concentration, the network fragments into smaller clusters and the connectivity undergoes a percolation transition. The giant component disintegrates with a power law behavior, described by a universal critical exponent beta = 0.65. By blocking the inhibitory synapses with bicuculine, we show that beta is independent of the balance between excitatory and inhibitory neurons. This proves that it is an intrinsic property of the network. Together with numerical simulations we show that the neural connectivity corresponds to a Gaussian distribution rather than a power law distribution. This may be the crucial difference between neural networks grown with cultured neurons versus those grown naturally in the brain.

# Abstract: STUDYING THE AUTOIMMUNE HYPOTHESIS OF OBSESSIVE COMPULSIVE DISORDER IN A RAT MODEL

**Authors:** L. Brimberg, I. Benhar, M. Cunningham, D. Joel

**Affiliations:** ^1^Department of Psychology, Tel Aviv University, PO Box 39040, Tel Aviv 69978, Israel; ^2^Department of Molecular Microbiology and Biotechnology, Tel Aviv University, PO Box 39040, Tel Aviv 69978, Israel; ^3^Department of Microbiology and Immunology, University of Oklahoma, USA

A post-streptococcal autoimmune process has been suggested to be involved in the pathogenesis of a subgroup of children with tics and obsessive compulsive disorder (OCD), identified by the acronym PANDAS for pediatric autoimmune neuropsychiatric disorder associated with streptococcal infection. It has been suggested that antibodies produced against group A beta-hemolytic streptococci (GABHS) cross react with neural cells, in a process involving molecular mimicry. Although PANDAS has received much attention in recent years, no study to date assessed compulsive-like behaviors in an animal model of GABHS-related neuropsychiatric symptoms. The aim of the present study was therefore to test the hypothesis that group A streptococcal infection may lead to the emergence of OCD. To this end, Lewis rats were immunized with an extract prepared fromGABHS, and their compulsive behavior was assessed using two rat models of OCD, namely, the signal attenuation model and the induced grooming assay. Rats immunized with GABHS extract had higher serumantibodies cross reactive with GABHS compared to the control group; had IgG deposits in their brains, primarily in the striatum and thalamus (control rats brain showed low level, if any, of anti IgG immunolabeling); and were more compulsive, that is, exhibited higher levels of compulsive lever pressing and spent more time grooming compared to the control group. These preliminary results suggest that immunizing rats with GABHS extract can lead to the emergence of compulsive behavior.

# Abstract: CONSTITUTIVE GLIAL NITRIC-OXIDE RELEASE AND BEHAVIORAL PHENOTYPE IN MICE WITH NOS2 MUTATION

**Authors:** Y. Buskila, Y. Abu-Ghanem, E. Grauer, Y. Amitai

**Affiliations:** ^1^Department of Physiology, Faculty of Health Sciences, Ben Gurion University, Beer sheva 84105, Israel; ^2^The Biolgical Institute for Research, Ness-Ziona, Israel; ^3^Zlotowski Center for Neuroscience, Ben Gurion University, Beer Sheva 84105, Israel

Nitric oxide (NO), a cellular signaling molecule, is produced in the brain by both neurons and astrocytes. NO is synthesized from L-arginine by several isoforms of the enzyme nitric oxide synthase (NOS), depending on the conditions and the cell type. Recently, we developed a method to distinguish neurons from astrocytes using NO imaging in brain slices with the NO indicator DAF-2DA and to demonstrate NOS2-dependent astrocytic NO production that followed neuronal fluorescence with a short delay of seconds to few minutes that did not involved de-novo protein synthesis (Buskila et al, 2005). Further examination of the function of astrocytic NOS2 activity in the brain is made by studying the NOS2 knockout mice (B6;129P2-NOS2TM1LAU/J, Jackson Laboratory). These mice showed robust compensatory NO production by neocortical astrocytes. The results of the NO imaging and the NOS activity assays revealed that the nature of the compensation was constitutive and Ca2+ dependent. Exploratory and stress behavioral tests of these mice exhibit a clear behavioral phenotype in the NOS2 mutants comparing to their littermate controls. We concluded the following. (1) Astrocytes are capable of rapid and massive NO production in response to acute neuronal death. (2) The iNOS KO mouse exhibits compensatory astrocytic NO release with a time course that is faster than in WT mice, suggesting an activity of a constitutively expressed enzyme. (3) iNOS KO mice exhibit specific behavioral modifications. Our data indicate for the first time the possibility that astrocytic NO release is involved in instantaneous management of brain events.

# Abstract: ABNORMAL SIGNALS FROM THE PERIPHERAL NERVOUS SYSTEM AND NEUROPATHIC PAIN

**Authors:** J. N. Campbell

**Affiliations:** Department of Neurosurgery, School of Medicine, Johns Hopkins University, Baltimore, MD 21205, USA

Neuropathic pain refers to chronic pain states where the generator of pain stems from disease of the nervous system. One common feature to each of these disease processes is that they involve an axotomy. Accordingly, neuropathic pain can be modeled in animals merely by cutting peripheral nerve axons. Paradoxically, where one would think that axotomy would simply induce a loss of sensation in the affected target tissue (the skin), patients and animals actually develop hyperalgesia (enhanced pain to stimuli). Patients may develop allodynia, which is manifest as pain to light stroking stimuli. This abnormality stems from central sensitization such that tactile afferents acquire the capacity to activate painsignaling cells in the dorsal horn and/or more rostral sites. This sensitization is under dynamic control however, and likely is maintained by abnormal inputs from the periphery. From where do the abnormal inputs come? Candidates include the nerve injury site (the neuroma), and the cell bodies of the injured afferents in the dorsal root ganglion. But these targets are not the whole story. For example, an L5 rhizotomy after (or before) an L5 spinal nerve ligation does not abolish hyperalgesia. This and other observations indicate that the nociceptive afferents that are not involved in the injury, and which share the innervation territory of the injured nerve, develop spontaneous activity and become sensitized (“intact nociceptor hypothesis”). This discovery provides a biological rationale for why distal therapies (treatment directed, eg, to the skin) may provide pain relief. Sensitization of the nociceptors to catechols likely accounts for the abnormal coupling of neural activity between sympathetic efferent fibers and nociceptors seen in some neuropathic conditions. The mechanism for the abnormal responses of the “intact” nociceptors may relate to increased levels of trophic growth factors in the denervated skin and Schwann cells.

# Abstract: LOSS OF AUTOIMMUNE T CELLS CORRELATES WITH SCHIZOPHRENIA

**Authors:** M. Cardon, J. Kipnis, R. D. Strous, M. Schwartz

**Affiliations:** ^1^Department of Neurobiology, Weizmann Institute of Science, 76100 Rehovot, Israel; ^2^Department of Experimental Neuroscience, University of Nebraska Medical Center, Omaha, NE 68198-5800, USA; ^3^Beer Yaakov Mental Health Center, 70300 Beer Yaakov, Israel; ^4^Sackler Faculty of Medicine, Tel Aviv University, Ramat Gan, Israel

Well-controlled T cell-dependent autoimmunity has been implicated as a physiological mechanism in brain plasticity, homeostasis, and repair of the brain at adulthood. Uncontrolled autoimmunity, however, can culminate in autoimmune disease. Here we investigated the relationship between these apparently contradictory effects of autoimmunity in patients with schizophrenia, in whom immune aberrations have prompted speculation about an autoimmune contribution to the etiology. We show that although, in general, schizophrenic patients have an active immune system, autoimmune clones reactive to major myelin proteins are often absent or inactive in these patients. This finding, in conjunction with our previous discovery in rodents that the immune system plays a key role in normal cognitive functioning, led us to suggest that a cause of the onset or of the ongoing progression of schizophrenia is a deficiency of the relevant autoimmune cells (specificity or phenotype).

# Abstract: REDUCED SENSORY ADAPTATION AS A RESULT OF PERCEPTUAL LEARNING

**Authors:** N. Censor, D. Sagi

**Affiliations:** Department of Neurobiology, Brain Research, Weizmann Institute of Science, 76100 Rehovot, Israel

Experimental evidence from perceptual learning in texture discrimination implies long-term changes in sensory networks. We have recently shown that both discrimination thresholds and learning depend on the number of trials used during training: intense sessions produce higher discrimination thresholds possibly due to sensory adaptation. These sessions reduce between-session learning, pointing to an interaction between sensory adaptation and the generation of long-term memory. Here we tested effects of perceptual learning on sensory adaptation. The standard texture stimulus was used, briefly presented (40 ms) and backwardmasked as by Karni and Sagi (1993). Observers decided whether an array of 3 diagonal bars embedded in a background of horizontal bars (19 × 19) was horizontal or vertical. One group of subjects practiced the texture discrimination task with 12 trials/block and a second group practiced the task with 50 trials/block (more adaptation, previously shown to reduce between-session learning). Both groups returned for an additional intense test-session with 50 trials/block. Results showed that the average threshold in the 50 trials/block testsession was significantly lower for the subjects trained with sessions of 12 trials/block, as compared to those trained with sessions of 50 trials/block. These results show that short practice sessions may have a role in reducing future suppressive processes occurring in the following intense sessions, thereby yielding efficient performance. We suggest a link between memory generation and adaptation processes operating in the visual network: short practice sessions with a stimulus may have a role in generating an effective memory. The modified network does not show the suppressive effects that are related to within session adaptation. On this account, adaptation is due to inefficient sensory processing. This proposed link may have an essential role in the underlying mechanisms of perceptual learning.

*Supported by the Basic Research Foundation administered by the Israel Academy of Sciences.*

# Abstract: DEVELOPMENTAL SWITCH OF ACTION POTENTIAL INITIATION IN NEOCORTICAL NEURONS

**Authors:** M. Chistiakova, A. Malyshev, M. Volgushev

**Affiliations:** ^1^Department of Neurophysiology, Ruhr-University Bochum, Bochum 44780, Germany; ^2^Institute of Higher Nervous Activity and Neurophysiology, Russian Academy of Sciences, Moscow, Russia

The generation of action potentials (APs) is a key point in operation of neurons and neural networks. We have shown recently that key features of AP initiation in cortical neurons in vivo, the step-like onset, and large variability of the onset potential cannot be captured by the classical Hodgkin- Huxley theory. Here we addressed the question, whether the fast, step-like AP initiation dynamics is present in neocortical neurons already at birth, or appears only later, during the development? To this end, we recorded APs in neocortical neurons (*n* = 149) in rat slices starting from the first postnatal week to adult (P30). The AP initiation dynamics changes dramatically during this period. In the first postnatal week, the APs onset dynamics in most neurons was slow, exponential-like, and thus compatible with the Hodgkin- Huxley description. However, even as early as P4, some neurons already expressed the step-like AP initiation dynamics, which deviated clearly from the theoretically expected. The proportion of such cells increased rapidly during the first postnatal weeks, and after P17 all recorded cells exhibited the fast, step-like AP initiation. The switch of the dynamics of AP initiation was accompanied by modification of other AP characteristics: a substantial increase of the maximal rate of membrane potential change during the AP, a shift of the absolute threshold towards more hyperpolarized values, a decrease of the width, and a moderate increase of the amplitude. Also basic electrophysiological properties of neuronal membranes change during the development: the resting potential became more hyperpolarized, and the input resistance decreased. We conclude that the mechanisms, responsible for the rapid, step-like AP initiation dynamics in neocortical neurons are not present at birth, but develop during the 1–3 postnatal weeks, in parallel with the maturation of the other electrophysiological properties of neuronal membranes.

*Supported by theDGF and FoRUM.*

# Abstract: THE EPISODIC NATURE OF OLIVARY OSCILLATIONS IN VIVO

**Authors:** E. Chorev, Y. Yosef, I. Lampl

**Affiliations:** ^1^Department of Neurobioloy and ICNC, The Hebrew University, Jerusalem 91010, Israel; ^2^Department of Neurobiology, Weizmann Institute of Science, 76100 Rehovot, Israel

The classic view of cerebellar function is based on the assumption that the inferior olive delivers to the cerebellar cortex an error signal. This signal is then used to modify cerebellar connections in a way that will prevent the reoccurrence of the error. Experimental findings revealing the capacity of olivary neurons to self generate regenerative patterns and to synchronize those patterns over the network triggered the idea that the inferior olive serves as a timing device for cerebellar activity. The synchronized rhythmic behavior of olivary output is still under debate. Although all the in vitro observations unequivocally revealed subthreshold activity, the auto-rhythmicity of complex spikes (cs) was observed only in few studies. This discrepancy between the subthreshold oscillation in the inferior olive observed in vitro and the sporadic reports on rhythmicity of complex spikes, can only be reconciled by recording directly from inferior olive neurons in situ. Therefore, we performed intracellular recordings from olivary neurons in vivo. We show that olivary activity under in vivo conditions resembles that observed in vitro. Hence, significant subthreshold oscillations (5–12 Hz), organized in epochs of 0.5 to several seconds, govern the membrane potential of olivary neurons. The action potentials occur solely during these epochs of oscillations. However, since these oscillatory epochs are independent from one another and due to the low firing rate of olivary neurons, it is only, seldom that the 5–12 Hz rhythmicity is revealed in the spikes autocorrelograms. Thus, we conclude that indeed the inferior olive oscillates in vivo and thus can serve as a timing device. The output of the inferior olive is time locked to the underlying oscillatory rhythm and most likely synchronized through electrotonic connections between olivary neurons thus activating in concert the cerebellar cortex.

# Abstract: A ZINC SENSING RECEPTOR MEDIATES ZINC SIGNALING IN THE HIPPOCAMPUS

**Authors:** U. Chorin, L. Besser, I. Sekler, M. Hershfinkel

**Affiliations:** ^1^Department of Morphology and Zlotowski Center for Neuroscience, Ben Gurion University, Beer Sheva 84105, Israel; ^2^Department of Physiology and Zlotowski Center for Neuroscience, Ben Gurion University, Beer Sheva 84105, Israel

*Objectives*. Dynamic changes in zinc play a key role in regulating synaptic transmission, but are also a leading factor in neuronal death following excitotoxic syndromes. We have identified an extracellular zinc sensing receptor, ZnR, in epithelial cells and hypothesized that such a receptor mediates intracellular signaling in the brain. *Methods*. Acute brain slices from mice (P4-12) were loaded with Fura-2AM for the physiological measurements. The mossy fiber-CA3 pyramidal neuron responses were induced by the stimulation of mossy fiber axons using trains of stimuli. *Results*. Following the application of extracellular Zn2+, a fluorescence rise was observed in the CA3-hippocampal and neocortical regions. The Zn2+-dependent Ca2+ response was inhibited by the Gq inhibitor, YM-245180, the PLC inhibitor, U73122, and by emptying of intracellular Ca2+ stores using thapsigargin. Thus, our results indicate that the Zn2+-dependent Ca2+ release ismediated via a brain-ZnR which acts via the IP3 pathway. We show that ZnR-responsive cells are not stained by the astrocytic marker, SR101. Importantly, ZnR activity was induced by endogenous Zn2+, released by electrical stimulation of the mossy fibers, and attenuated in the presence of the extracellular zinc chelator, CaEDTA. A similar reduction was observed using the ZnT-3 KOmice, deficient of synaptic zinc. The Zn2+-dependent Ca2+ response persisted in the presence of the metabotropic glutamate receptor inhibitors. *Conclusions*. Our results suggest that synaptically released zinc, at the CA3 region, activates a specific zinc sensing receptor, ZnR. Our work therefore suggests a role for zinc acting as a neurotransmitter via a specific zinc sensing receptor located on CA3 neurons.

# Abstract: VOLTAGE-DEPENDENT INHIBITION OF K2P2.1 CHANNELS BY EXTERNAL PROTONS

**Authors:** A. Cohen, S. Hen, Y. Ben-Abou, N. Zilberberg

**Affiliations:** Department of Life Sciences and Zlotowski Center for Neuroscience, Ben Gurion University, Beer Sheva 84105, Israel

Potassium leak channels operate like K+-selective holes in an electric field as their currents are potassium selective and instantaneous and their conductance is independent of membrane potential. Leak currents play a critical role in determining cell resting membrane potential and thus controlling nerve and muscle excitability. K2P2.1 (KCNK2, TREK- 1), a member of the 2P-domain K+ channel (K2P) family, is expressed in the central and peripheral nervous system, as well as in the heart, kidney, and testis tissues. K2P2.1 activity is regulated by a variety of physical and chemical effectors including temperature, fatty acids, internal pH, mechanical stretch, and phosphorylation. Although K2P2.1 is classified as a leak channel, it was shown to posses an exceptional capability to reversibly convert between leak- and voltage-dependent phenotypes. This unique behavior can be mediated by phosphorylation of a unique serine at the carboxy-terminal tail of the channel. Here, we report that human K2P2.1 channel is strongly inhibited by external acidosis in a potassium-dependant manner (pKa = 7.3 at 4 mM external potassium). Two histidine residues (H87 and H141), located at the first external turret loop of the channel, were found to be crucial for proton sensitivity at physiological range. Interestingly, external pH (pHo) sensitivity was voltage-dependent, being more prominent at negative potentials. In addition, a mutant channel mimicking a constantly phosphorylated channel was significantly more inhibited by pHo than a mutant mimicking a constantly nonphosphorylated channel. Moreover, the voltage dependency of K2P2.1 was significantly reduced in the pHo insensitive mutant, turning it into an open rectifier channel. Taken together, our data suggest that conversion of the K2P2.1 channel between leak and voltage-dependent phenotypes involves an external pH sensor, identified here for the first time.

# Abstract: AN ELECTROPHYSIOLOGICAL CHARACTERIZATION OF THE PROJECTIONS BETWEEN THE ORBITOFRONTAL CORTEX AND THE PIRIFORM CORTEX

**Authors:** Y. Cohen, I. Reuveni, E. Barkai, M. Maroun

**Affiliations:** Department of Neurobiology and Ethology, Center for Brain and Behavior, University of Haifa, Haifa 31905, Israel

The orbitofrontal cortex (OFC) entails high olfactory cortical areas, in which information about the identity and also about the reward value of odors is represented. The piriform cortex (PC) is the traditional primary olfactory cortex and is thought to have an important role in olfactory learning tasks. It has been proposed that the piriform cortex may function as an olfactory association cortex, integrating sensory input from olfactory bulb with learned associative information from downstream regions. The purpose of the present study was to characterize the physiological properties of projection from the PFC to the PC. Stimulating electrodes applied at the OFC induced a graded response in layer II of the anterior PC (the pyramidal cells layer). The averaged delay of the response was 7.16 + 0.34 ms (*n* = 8), indicated that the connections to monosynaptic. The amplitude of the response increased linearly with increasing stimulus intensity: from 0.38 + 0.13 mV in response to a stimulus intensity of 0.3 mA to 0.99 + 0.49 in response to a stimulus intensity of 1 mA (*n* = 8). Following LTP induction with theta burst stimulation, the amplitude of the response increased significantly throughout the impost/output curve. For example, that amplitude in response to a 0.7 mA stimulation increased from 0.71 + 0.37 to 0.88 + 0.49 mV (a significant averaged increase of 23%, *n* = 8. LTP was measured 60 minutes after repetitive stimuli application). Our data support the notion that a monosynaptic response can be elicited in the anterior PC in response to an electrical stimulation applied in the OFC and that this response can be potentiated in an activitydependent manner.

*Supported by grant fromthe ISF.*

# Abstract: DEVELOPMENTAL REGULATION OF SPONTANEOUS ACTIVITY IN CULTURED HIPPOCAMPAL NEURONS

**Authors:** E. Cohen, M. Segal

**Affiliations:** Weizmann Institute of Science, Rehovot 76100, Israel

Correlated network activity is crucial for the normal development of the neonatal brain. This population activity is expressed as spontaneous calcium bursts (SCBs), which can propagate through neuronal tissue and are associated with neuronal growth. Though such spontaneous activity has been described for over 3 decades, the exact relationship between network SCBs and their underlying morphological substrate is still not well understood. In the present study we attempted to correlate developmental changes in SCBs, with morphological attributes of the network. Spontaneous activity of hippocampal cultures became synchronized between 2 and 3 days in vitro (DIV), and this attribute was not accompanied by any apparent morphological or synaptic maturation. Conversely, the profound morphological maturation that occurred in the ensuing 11 days was correlated with specific SCB properties. The spontaneous activity exhibited GABAAR and AMPAR dependency already at 3DIV and GABA switched from being excitatory to being inhibitory between 3 and 7DIV. Chronic blockade of NMDAR, AMPAR, or GABAAR for the first 3DIV did not prevent the emergence of SCBs at 3DIV; finally, NMDA receptors were not involved in the generation of SCBs at any developmental stage of the cultures, but played a significant role in the 14DIV cultures where the variance of burst amplitudes, hypothesized to be directly related to the information content carried by the activity, was blocked by an NMDA antagonist, APV. Thus, dissociated hippocampal culture proved a suitable preparation for developmental studies, reliably reconfirming earlier electrophysiological and pharmacological results found in slices and in vivo, while permitting rigorous quantifications of both population-wide activity patterns and morphological attributes in the same preparation.

*Supported by a grant from the Israel Science Foundation.*

# Abstract: OLFACTORY-LEARNING-INDUCED ENHANCEMENT NEURONAL EXCITABILITY IS RELATED TO THE COMPLEXITY OF THE TASK

**Authors:** S. I. Cohen-Matsliah, K. Rosenblum, E. Barkai

**Affiliations:** Department of Neurobiology and Ethology, Faculty of Sciences, University of Haifa, Haifa 31905, Israel

Acquisition of the skills to performcomplex behavioral tasks, such as the Morris water maze and olfactory-discrimination learning in four-armed maze, is accompanied by long-lasting enhanced neuronal excitability, which results from reduction in the post burst afterhypolrization (AHP) in pyramidal neurons. It has been suggested that AHP reduction is not a mechanism by which specific memories are stored. Rather, it enables the cortical network to enter into a learning mode, in which new memories can be stored rapidly and efficiently. Here we examined whether learning of a “simple” task within few trial in a single session would also result with such modifications. Our hypothesis was that such simple tasks would not be accompanied by intrinsic modifications. Rats were required to associate a specific odor with water reward in an open box. This task is rapidly acquired and well remembered over several days. Intracellular recordings were preformed in layer II piriform cortex pyramidal neurons, in brain slices, 12 hours, 1 day or 3 days after learning. In contrast to our observations after complex olfactory learning, simple olfactory learning was not accompanied by AHP reduction (3 days after learning: trained 6.3 mV + 1.6, *n* = 13; pseudotrained: 7.1+2.1, *n* = 15; 1 day after learning: trained 7.1 + 2.7, *n* = 20; pseudotrained: 6.4 + 1.7, *n* = 21; 12 hours after learning: trained 7.2 + 2, *n* = 20; pseudotrained: 6.6 + 2.7, *n* = 16; naïve: 7.0 + 2.2, *n* = 33). Interestingly, 1 day after learning we observed a reduction in the number of spikes generated during prolong depolarization, which indicate a reduction in excitability in those cells (trained: 15 + 8, *n* = 20; pseudotrained: 23 + 10, *n* = 17; naïve: 24 + 13, *n* = 30). Our data support the notion that the learning-induced enhancement in neuronal excitability is influenced by the complexity of the behavioral task. Thus, simple and intensive training differ by their underling cellular mechanisms.

*Supported by grant fromthe ISF.*

# Abstract: IMPAIRMENT OF THE BBB UPON EXPOSURE TO PRION

**Authors:** I. Cooper, M. Salmona, V. I. Teichberg

**Affiliations:** ^1^Department of Neurobiology, Weizmann Institute of Science, Rehovot 76100, Israel; Mario Negri Institute, Milan, Italy

The prion, a conformational variant of an endogenous protein, is the infectious particle responsible for transmissible spongiform encephalopathy (TSE), a fatal neurodegenerative disease of humans and animals. When the disease is acquired following the consumption of infectedmeat, the common dogma claims for neuroinvasion of the prion via neural pathways (vague and splanchnic nerves), which is usually preceded by the prion propagation into secondary lymphoid organs. The goal of this study was to examine the possibility that prion enters the brain directly from the blood stream by crossing the blood brain barrier (BBB). For this purpose we made use of an in vitro model of the BBB composed of primary cultured porcine brain microvessel endothelial cells seeded on semi-permeable membrane. Upon coculture with astrocytes the endothelial cells acquire a typical BBB feature, that is, high trans endothelial electrical resistance (TEER) of above 400 ohm×cm2. The interaction of the synthetic peptide corresponding to the 106–126 amyloidogenic region of the cellular human prion protein (PrPC) with the BBB was examined. This peptide is widely used in prion studies due to its biophysical properties resembling the infectious form of prion protein (prpsc). The prp 106–126 was observed to prevent the formation of high TEER when added 48 hours after the onset of the coculture with astrocytes. If added to the endothelial culture when a high TEER was achieved, the prp 106–126 was found to significantly decrease the TEER by about 50%. The effects were sequence specific as the scrambled prp 106–126 peptide did not produce any effect. Moreover, the effect on the BBB was blood-side specific implying that a prion penetrating into the brain via the blood stream is a plausible event. This possibility raises questions about the risk of iatrogenic prion spread through blood transfusions and about new therapeutic strategies against this disease.

# Abstract: CANNABINOIDS AMELIORATE CEREBRAL DYSFUNCTION IN EXPERIMENTAL HEPATIC ENCEPHALOPATHY VIA AMP-ACTIVATED PROTEIN KINASE

**Authors:** Y. Dagon, Y. Avraham, Y. Ilan, R. Mechoulam, E. M. Berry

**Affiliations:** ^1^Department of Human Nutrition and Metabolism, Braun School of Public Health, Faculty of Medicine, The Hebrew University, Jerusalem 91120, Israel; ^2^The Liver Unit, Hadassah University Hospital, Ein Kerem, Jerusalem, Israel; ^3^Department of Medicinal Chemistry and Natural Product, Medical Faculty, The Hebrew University, Jerusalem 91120, Israel

Hepatic encephalopathy (HE) is a neuropsychiatric disorder of unknown pathogenesis caused by acute or chronic liver failure. We studied the etiology of cerebral dysfunction in a murine model of HE, induced by either bile duct ligation or thioacetamide administration. We report that stimulation of cerebral AMP activated protein kinase (AMPK), a major intracellular energy sensor, is a compensatory response to liver failure. This function of AMPK is regulated by endocannabinoids. The cannabinoid system controls systemic energy balance via both cannabinoid receptors type 1 (CB1) and 2 (CB2). Under normal circumstances AMPK is mediated by CB-1 whilst CB-2 is barely detected. In contrast, liver failure stimulates the expression of the cannabinoid receptor type 2 (CB-2). Administration of Δ9-tetrahydrocannabinol (THC) augmented AMPK activity and restored brain function inWT mice but not in their CB-2 KO littermates. These results suggest that HE is a disease of energy flux. CB-2 signaling is a cerebral stress response mechanism and makes AMPK a promising target for its treatment by manipulation of the cannabinoid system.

# Abstract: MISMATCH NEGATIVITY AND THE RELATIONS BETWEEN SENSITIVITY TO FREQUENCY DEVIANCE AND TO STANDARD PERTURBATION

**Authors:** L. Daikhin, M. Ahissar

**Affiliations:** ^1^Hadassah Medical School, The Institute of Medical Sciences, Jerusalem, Israel; ^2^Department of Psychology, The Hebrew University, Jerusalem 91010, Israel; ^3^Interdisciplinary Center for Neural Computation, The Hebrew University, Jerusalem 91010, Israel

Mismatch negativity (MMN) is an automatic response produced mainly by the auditory cortex to an oddball presented in a sequence of sounds. The magnitude of MMN monotonically increases with increasing degree of deviance. We now asked whether roving the standard stimulus interacts with MMN sensitivity to frequency deviance. We assessed the MMN of 10 participants to 3 different deviants (8, 40 and 100%) in 3 separate sessions. In each session 2 different paradigms were applied. In one (fixed), the 1000 Hz reference was presented with 90% probability, and deviant in 10%. In the second (roving), each of 10 stimuli was presented with 10% probability. Nine stimuli were clustered around 1000 Hz (980, 985, 990, 995, 1000, 1005, 1010, 1015, 1020) and the 10th was the deviant. We asked whether the MMN response would be similarly affected by roving the standard under each of the deviant conditions. We found that, when averaged across participants, MMNamplitude increases with increasing deviance (2.2 ± 1.6, 4.3 ± 1.7, 6.1 ± 1.8 microV, resp) and roving the standard decreases its amplitude to the same degree for all deviants (1.06 ± 0.8, 3.19 ± 1.1, 5.33 ± 1.8 microV, resp). Thus, at the group level, sensitivity to deviance and to standard consistency seem independent. However, this grand average results from opposing trends at the single subject level. Half the subjects showed increased MMN rovinginduced- impairment with increasing deviance, whereas half the subjects showed the reverse pattern. We conclude that, consistent with a passive model of adaptation determined by the probability of stimulus specific repetition, MMN sensitivity to the 2 manipulated factors seems independent. However, further within subject, assessments are required to determine whether this conclusion is true for single subject responses.

*Acknowledgments: we thank Yael Handler, Mor Nahum, and Sarit Szpiro for their contribution.*

*Supported by The Israeli Institute for Psychobiology and The Center of Excellence Grant from the ISF.*

# Abstract: REGULATION OF Ca2+ CHANNEL GATING BY THE CYTOSOLIC DOMAIN AND AUXILIARY SUBUNITS AND PROTEINS

**Authors:** N. Dascal

**Affiliations:** Sackler Faculty of Medicine, Tel Aviv University, Tel Aviv 69978, Israel

L-type Ca2+ channels are crucial for the contraction of cardiac and smooth muscle and play important roles in neuronal functions. The main subunit of these channels, Cav1.2, is encoded by a multiexon gene (more than 50 exons). Alternative splicing gives rise tomany isoforms that appear to be tissue-specific. Human Cav1.2 gene has two alternative initial exons that encode two channel isoforms: long NT alpha 1C starting with 46-amino acid (aa) encoded by exon 1a, and a short-NT Cav1.2 staring with 16-aa encoded by the alternative exon 1. The long-NT Cav1.2 is predominant in the heart, short-NT isoforms prevail in smooth muscle and brain. We show that the initial segment of the long- NT Cav1.2 is an inhibitory module (NTI module) that reduces the maximal open probability, Po, max, and its deletion greatly increases Ca2+ or Ba2+ currents via L-type channels. Removal of the NTI module reduces the enhancement of currents caused by coexpression of the beta subunit, Cavb. However, purified NT does not interact with the beta subunit, suggesting an allosteric effect. By mutagenesis we mapped the NTI module to the first 20 amino acids of the long-NT and demonstrated that only the cardiac but not the brain/smoothmuscle isoformpossesses that NTI module. By measurements of channel currents, surface expression and single-channel properties, we found that the NT inhibitory module exclusively regulates a single function of the Cav-b subunit: the increase in Po, max. The NT of Cav1.2 contains binding sites for calmodulin (CaM) and for the neuronal Ca2+-binding protein 1, CaBP1, but the function of these sites is not known. CaBP1 counteracts the CaM-dependent channel inactivation caused by Ca2+ influx. Deletions in NT lessen the effect of CaBP1 by shifting the voltage dependency of components of Ca-dependent inactivation. We propose a model that provides a framework in which to study how the cytosolic segments of Cav1.2, the beta subunit, CaM, and CaBP1 regulate channel function.

*Supported by BSF and ISF.*

# Abstract: ATTENTIONAL PERSEVERATION AFTER NEONATAL NITRIC OXIDE INHIBITION IS NORMALIZED BY CLOZAPINE AND GLYB AGONISTS BUT NOT HALOPERIDOL: A NEURODEVELOPMENTALMODEL OF NEGATIVE SYMPTOMS IN SCHIZOPHRENIA

**Authors:** A. De Levie, I. Weiner

**Affiliations:** Department of psychology, Tel Aviv University, Tel Aviv 69978, Israel

Animal models of schizophrenia increasingly reflect the consensus that schizophrenia is a neurodevelopmental disorder. Based on evidence of abnormalities in nitric oxide (NO) system in schizophrenia that are of a neurodevelopmental origin, we investigated the effects of early postnatal NO synthase (NOS) inhibition on the development of latent inhibition (LI) in rats. LI is a cross-species phenomenon, in which repeated nonreinforced preexposure to a stimulus results in the development of inattention to the stimulus, which interferes with its subsequent conditioning. We found that neonatal NOS inhibition did not affect LI at prepubertal age, but led to the emergence of an abnormally persistent LI at adulthood in male but not in female rats. Because attentional perseveration and gender differences are associated with negative symptomatology, abnormally persistent LI following neonatal NOS inhibition may be relevant to this symptomatology. In support, this cognitive abnormality exhibited a pharmacological profile characteristic of negative symptoms, whereby it was reversed by drugs enhancing NMDA receptor function glycine and D-cycloserine and by clozapine but not by haloperidol. In addition, we found that NOS inhibition led to decreased sensitivity to the locomotor-stimulating effects of amphetamine and this effect was evident already at prepubertal age, possibly representing a “prodromal state” fully expressed in adulthood. These results suggest that neonatal NOS inhibition May provide a neurodevelopmental model of negative symptoms of schizophrenia that mimics its temporal course and predicts responsiveness to atypical neuroleptics and putative drugs.

# Abstract: IMPACT OF THE MUSCARINIC-SENSITIVE POTASSIUM CURRENT (M-CURRENT) ON NEURONAL EXCITABILITY AND TRANSMITTER RELEASE IN HIPPOCAMPAL NEURONS

**Authors:** N. Degani-Katzav, A. Peretz, B. Attali

**Affiliations:** Department of Physiology and Pharmacology, Sackler Faculty of Medicine, Tel Aviv University, Tel Aviv 69978, Israel

The M-current is a subthreshold, slowly activating and noninactivating voltage-gated potassium current that controls neuronal excitability by limiting repetitive firing. The KCNQ2/Q3 channel complex was identified as the molecular correlate of the M-current. Mutations of the KCNQ2 and KCNQ3 genes lead to BFNC, a neonatal form of epilepsy. The objective of this research was to evaluate the impact of M-channels on neuronal excitability and transmitter release in hippocampal neurons. Spontaneous excitatory and inhibitory post synaptic currents (sEPSCs and sIPSCs) were recorded from cultured primary hippocampal neurons. The impact of the M-channels on sEPSCs and sIPSCs was evaluated using the inhibitor linopirdine, and the openers retigabine and diclofenac. Our results indicate that blockade of the M-channel activity by linopirdine markedly enhances the frequency of sEPSCs and sIPSCs. On the contrary, enhancing the M-channel activity markedly reduces the frequency of sEPSCs and sIPSCs. In addition, the spiking pattern of sEPSCs was changed following the addition of M-current blocker or openers. The impact of the M-channels on miniature EPSCs (mEPSCs) of hippocampal neurons was also evaluated. Preliminary results indicate that blockade of the M-channel activity by linopirdine enhanced the frequency of mEPSCs in two out of seven cells. Immunocytochemical experiments indicate the presence of KCNQ2 subunits in both glutamatergic and GABAergic hippocampal neurons as identified with antibodies directed against the synaptic markers vGlut1 and GAD 65, respectively. In addition to its somatic location, substantial KCNQ2 immunoreactivity was clearly co-localized with vGlut1 and GAD 65 at synaptic varicosities. In all, our data indicate that in addition to their somatic/ axonal location, presynaptic M-channels may play a substantial role in regulating the release of glutamate and GABA to dampen neuronal excitability.

*Supported by ISF (672/05) and the Keren Wolfson Family Funds to the third author.*

# Abstract: EXPRESSION OF SOLUBLE GUANYLYL CYCLASE ALPHA IN LOCUST EYES: A RE-EVALUATION

**Authors:** A. Delago, S. R. Ott, M. R. Elphick

**Affiliations:** ^1^Department of Life Sciences, Ben Gurion University of the Negev, Beer Sheva 84105, Israel; ^2^Department of Zoology, University of Cambridge, UK; ^3^School of Biological and Chemical Science, Queen Mary University of London, UK

One potential source of cGMP in the locust eye is the nitric oxide-activated enzyme soluble guanylyl cyclase (SGC). Functional SGC is a heterodimeric dimeric protein comprised of an alpha subunit and a beta subunit. Elphick and Jones (1998) developed antibodies to a conserved region of Drosophila SGC alpha to investigate the expression of this subunit in the locust nervous system. Three antisera, XA4, XB3, and XC3, recognized a SGC alpha-like protein (circa 65 kDa) in locust tissue (Elphick and Jones, 1998). Immunocytochemical analysis of locust brain with XA4 and XC3, but not with XB3, revealed staining in antennal mechanoreceptors and in antennal lobe olfactory interneurons. In the retina of locust eyes very strong staining was observed in the perirhabdomeric compartment of each ommatidium, suggesting that SGC may be involved in locust phototransduction. However, 65 kDa is less than the molecular mass of Drosophila SGC alpha (circa 75 kDa; Shah and Hyde, 1995). Moreover, the expression levels of the putative SGC alpha subunit in locust photoreceptor cells could not explain the twofold nitric oxide donor induced increase in cGMP levels in locust eyes (Jones and Elphick, 1999) that seemed very low in comparison to what was reported for other species (Shah and Hyde, 1995; Stone and Marletta, 1994). Using Western blot analysis of tissue homogenates of wild type and SGC alpha mutant Drosophila we thus first established that the antisera against the X-peptide region in Drosophila SGC alpha do recognise this subunit in flies. This analysis revealed that XB3, but not XA4 or XC3, is the most specific of the available X-peptide antisera. Using XB3 antisera we then reinvestigated the expression pattern of the SGC alpha-like protein in locust brain and eye tissue. It appears that this subunit might not be as abundant as previously assumed, shedding a different light on the role of NO-cGMP signaling in locust phototransduction.

*This work was supported by grants awarded to the third author by the BBSRC (Grant numbers S03858 and S11816).*

# Abstract: SENSORY-EVOKED CEREBRAL BLOOD FLOW RESPONSES IN AWAKE MONKEY V1 RECORDED IN LARGE VASCULAR NETWORKS AT MICRO-VASCULAR RESOLUTION

**Authors:** T. Deneux, I. Vanzetta, S. Fisher, A. Grinvald, O. Faugeras, G. S. Masson

**Affiliations:** ^1^Team “Odyssee,” Ecole Normale Superieure de Paris and INRIA Sophia-Antipolis, France; ^2^Team “Dynamique de la Vision et de l'Action,” INCM, CNRS-UMR 6193, Aix-Marseille University, France; ^3^Department of Neurobiology, Weizmann Institute of Science, Rehovot 76100, Israel

The widespread use of hemodynamics-based functional brain mapping techniques (in particular fMRI) underscores the importance of detailed, physiologically realistic, hemodynamic response models. The accuracy of such models has, however, remained limited due to an incomplete characterization of the various response components and their interplay. Their improvement requires measures at fine spatial scales, allowed by optical imaging techniques. Here, we present a new method based on computer vision algorithms to record cerebral blood flow (CBF) at micro-vascular resolution, by tracking moving red blood cells (RBCs) in individual vessels of the superficial cortical vasculature and estimating their velocity, from high spatiotemporal resolution (15micron, 5ms) optical imaging data. Experiments were held on the awake, fixating monkey, recording in the primary visual cortex. Using the periodic modulation of the CBF by the heart beat as a gauge signal, we first tested our motion extraction algorithms by comparison with simultaneously performed laser doppler CBF measurements. The much smaller CBF responses to a visual stimulus could also be detected, in individual elements of the cortical microvasculature. The results indicate the presence of compartmentspecific responses. Further development of the image processing algorithms such as automatic vessel selection will allow obtaining high-resolution CBF maps. Those will be useful to characterize, at fine spatial scale, the vascular dynamics resulting from neuronal activation, thus providing (i) phenomenological input for constraining multicompartment hemodynamic response models, as well as (ii) further insights on which part of the active vascular response best colocalizes with sites of increased neuronal activity.

*Partial support from the European project FACETS, FRM fellowship (IV).*

# Abstract: NEUROPATHIC PAIN: MECHANISMS AND HERITABILITY

**Authors:** M. Devor

**Affiliations:** Departments of Cell and Animal Biology and of Neurobiology, Life Science Institute and Research on pain Center, The Hebrew University, Jerusalem 91010, Israel

Injury to axons in the peripheral nervous system at least partially blocks the flow of afferent information from the periphery to the central nervous system. When this occurs, we expect there to be a loss, or at least a blunting, of sensation. Yet clinical experience shows that neural injury is frequently accompanied by amplified and distorted sensation in the form of allodynia, hyperalgesia, hyperpathia, and ongoing neuropathic pain. At its most extreme, for example, in the case of phantom limb pain in amputees, traumatic neuropathy appears to create elaborate sensations from no input at all. Accumulating evidence points to abnormal discharge originating in the peripheral nervous system as both a primary nociceptive signal, and as a factor that triggers and dynamically maintains central sensitization. This motivates examination of the causes of the abnormal discharge. Axotomy induces altered regulation of gene expression in primary sensory neurons in the affected dorsal root ganglia (DRGs), and to a certain extent, in intact neighboring ganglia. Key among these changes are alterations in the expresssion of ion channels and the consequent emergence of high-frequency subthreshold oscillatory potentials and depolarizing afterpotentials (DAPs). The emergence of intrinsic resonant properties in these neurons alters their fundamental impulse generating capability, and promotes abnormal repetitive firing. Axotomy also triggers a change in the expression of certain peptide neurotransmitters which may render neurons that formerly triggered only touch sensation now to trigger pain. Individual and strain differences have been identified in the extent to which these phenotypic changes occur. A consequence is major heritable differences in susceptibility to neuropathic pain.

# Abstract: ROLE OF PKC IN GNRH ACTIVATION OF EXTRACELLULAR SIGNAL-REGULATED KINASE (ERK) AND JUN N-TERMINAL KINASE (JNK)

**Authors:** M. Dobkin-Bekman, Z. Naor, R. Seger

**Affiliations:** ^1^Department of Biochemistry, Tel Aviv University, Tel Aviv 69978, Israel; ^2^Department of Biological Regulation, The Weizmann Institute of Science, Rehovot 76100, Israel

The role of PKC isoforms in GnRH-stimulated MAPK was examined in gonadotrope cell lines. Incubation of the cells with GnRH resulted in a protracted activation of ERK1/2 and a slower and transient activation of JNK1/2. GnRH induced a rapid (1–3 minutes) translocation of PKCalpha to the membrane, followed by redistribution to the cytosol. PKCdelta and PKCepsilon seem to localize to the golgi, followed by redistribution to the perinuclear zone and a slow translocation to the membrane (30 minutes) in GnRH-treated cells. The pan PKC isoforms inhibitor, GF 109203X, nearly abolished GnRH stimulation of ERK1/2, but only reduced JNK1/2 activation by 50%. The cPKC inhibitor, Go 6976, reduced GnRH stimulation of ERK1, but markedly inhibited ERK2 activation and abolished GnRH stimulation of JNK1/2. The selective PKCdelta inhibitor, rottlerin, markedly inhibited GnRH stimulation of ERK1/2 with little effect on JNK1/2. GF 109203X abolished TPA-stimulation of ERK1/2 and JNK1/2. As with GnRH, Go 6976 was a more potent inhibitor of TPA stimulation of ERK2 (60%) versus ERK1 (20%), but JNK1/2 activation was abolished. Unlike GnRH, rottlerin reduced significantly TPA signaling to JNK1/2. Coexpression of GFP epitope-tagged ERK and dominant negative plasmids of PKCalpha and PKCdelta revealed that PKCalpha plays a partial role in ERK activation by GnRH, with a prominent role for PKCdelta. A cPKC isoform is involved in both GnRH and TPA-stimulation of JNK1/2, while PKCdelta participates in TPA but not in GnRH signaling to JNK1/2. Comparing the alphaT3-1 to the more mature LbetaT-2 gonadotropes revealed that the role of PKC in GnRH to ERK signaling diminishes with maturation of pituitary gonadotropes and the maturation has produced a switch in the relative sensitivity of ERK1 versus ERK2 to the PKC isoforms inhibitors. The above data suggest that GnRH and TPA activate ERK and JNK by common and separate PKC isoforms and indicate for the first time differential role of PKC in ERK1 versus ERK2 activation.

*Supported by ISF, GIF, and Adams Super Centre for Brain Studies at Tel Aviv University.*

# Abstract: ISOFORM SPECIFIC EFFECTS OF ApoE4 ON THE FORMATION OF SOLUBLE ABETA OLIGOMERS IN VIVO

**Authors:** I. Dolev, D. M. Michaelson

**Affiliations:** Department of Neurobiochemistry, Tel Aviv University, Tel Aviv 69978, Israel

ApoE4, the major genetic risk factor of Alzheimer's disease (AD), is associated with increased deposition of amyloidbeta (Abeta). Recent evidence suggests that the pathological effects of Abeta are mediated by soluble oligomers which are formed during the early stages of the amyloid cascade. The objective of our work was to characterize the initial aggregation stages of Abeta in vivo and the effects thereon of apoE4. This was performed by prolonged inhibition of the Abeta degrading enzyme neprilysin and examination of the resulting effects on the levels, sizes, and chemical composition of brain Abeta oligomers in apoE transgenic mice. This revealed that the total levels of soluble Abeta increased similarly and progressively for at least one month in brains of apoE4 transgenic mice and of transgenic mice which express the AD benign allele, apoE3. In contrast, the extent and kinetics of oligomerization of Abeta were markedly affected by the apoE genotype. The extent to which soluble Abeta oligomerize was first examined by size filtration. This revealed the occurrence of a population of Abeta oligomers of which those sized about 30–50 kD were specifically elevated in apoE4 mice. Characterization of these apoE4 stimulated oligomers and determination of their exact size and cellular location revealed that they are composed of SDS stable bands with apparent molecular weight of 40 and 55 kD which were particularly elevated intracellularly. Parallel measurements revealed that the levels of intact apoE were similar in the apoE3 and apoE4mice. In contrast, the extent of apoE degradation was specifically elevated intracellularly in the apoE4 mice. These findings suggest that the 40 and 55 kD Abeta oligomers are preferentially stabilized by apoE4 intracellularly and that these oligomers may mediate the pathological effects of apoE4. The mechanism underlying the stimulation of Abeta oligomerization by apoE4 and the role of apoE fragmentation in this process will be discussed.

# Abstract: THE EFFECT OF COMBINED ADMINISTRATION OF ATYPICAL ANTIPSYCHOTICS AND SELECTIVE SEROTONIN REUPTAKE INHIBITORS ON THE FIRING ACTIVITY OF SEROTONIN AND NOREPINEPHRINE NEURONS

**Authors:** E. Dremencov, M. El Mansari, P. Blier

**Affiliations:** Institute of Mental Health Research, University of Ottawa, 1145 Carling Ave, Ottawa, Ontario, Canada K1Z7K4

The atypical antipsychotics risperidone and olanzapine were previously shown to reverse the escitalopram-induced inhibition of norepinephrine (NE) neuronal firing activity (Psychopharmacol 181 : 126, 2005; Dremencov et al, Biol Psychiatry Epub 2006, August 23). Thus, nonresponse to selective serotonin (5-HT) reuptake inhibitors (SSRIs) in some depressive patients may be explained by a decreased NE tone and the beneficial effect of atypical antipsychotics by its reversal. The present study aimed to determine if paliperidone (the 9-OH metabolite of risperidone) exerts distinct effects on 5-HT and NE neuronal activity fromthose of risperidone. It was found that the acute administration of risperidone (0.4 mg/kg, IV) produced a robust inhibition of firing rate of 5-HT neurons. This inhibition was partially reversed by the NE reuptake inhibitor desipramine (5 mg/kg, IV) or by the 5-HT1A antagonist WAY 100635 (0.1 mg/kg, IV) and completely reversed when both WAY 100635 and desipramine were given. The same degree of inhibition of 5-HT neurons was observed after 2 or 14 days of risperidone administration (1 mg/kg/d, SC). However, 1 mg/kg/d of paliperidone did not alter the firing rate of 5-HT neurons neither after 2 or 14 days of administration. Paliperidone, as observed with risperidone, did not alter the firing rate of NE neurons by itself but reversed the escitalopram-induced suppression of NE neuronal firing after 2 or 14 days of coadministration. However, differently from risperidone, paliperidone coadministered with escitalopram did not elevate the firing rate of NE neurons above the value of control animals (after 2 days of administration) and did not alter the effect of sustained escitalopram administration on 5-HT neuronal firing activity. The capacity of paliperidone to reverse the SSRI-induced inhibition of NE neuronal firing rate, without decreasing 5-HT neuronal activity, suggests that paliperidone may be beneficial in SSRI-resistant depression.

*Supported by Janssen Pharmaceuticals.*

# Abstract: CHARACTERIZATION AND FUNCTIONAL ANALYSIS OF THE NOVEL BRAIN GENE KIAA0863

**Authors:** E. Dresner, M. Kushnir, I. Gozes

**Affiliations:** Human Molecular Genetics and Biochemistry, Sackler Medical School, Tel Aviv University, Tel Aviv 69978, Israel

KIAA0863 was recently identified by bioinformatics tools as a paralog protein to activity-dependent neuroprotective protein (ADNP). ADNP is an 1102 amino acid protein, with widespread expression in the brain. It was found that ADNP protects neurons from various damages and is essential to brain formation and to neural tube closure. KIAA0863, its paralog protein, has 33% identity and 46% similarity with ADNP. Like ADNP, the protein contains zinc fingers motifs and a homodomain profile, which characterize transcription factors. KIAA0863 is ubiquitously expressed in the mouse tissues, especially in the brain. Its messenger RNA has been detected from developmental day 7.5 until birth. In the mouse brain, high levels of KIAA0863 gene expression have been noted. These high levels were sustained through embryogenesis and adulthood. In cancerous tissues such as breast and colon cancer, an increased expression level of KIAA0863 has been found (Michal Kushnir, MS Thesis). Knock down of KIAA0863 using RNA interference technology in P19 cells caused inhibition of the cell proliferation. When P19 cells were treated with hydrogen peroxide, knock down of KIAA0863 reduced its toxicity and improved cell survival. Due to the similarity between KIAA0863 and ADNP, and because of the involvement of KIAA0863 in proliferation and cell death processes, we believe that this gene has a critical role in the nervous system. Understanding of KIAA0863 function and mechanism may shed light on processes related with cell death, neuronal protection, and cancer. In ongoing experiments, we are examining the effect of KIAA0863 knock down on the proapoptotic protein, P53.

*BSF, ISF, Lily and Avraham Gildor Chair, Dr Diana, and Zelman Elton Lab and Allon Therapeutics.*

# Abstract: THE ENDOCRINE REGULATION OF BUTYRYL CHOLINESTERASE IN ADULTMALE DYSTROPHIN-DEFICIENT (MDX) MICE

**Authors:** A. R. Durrant, L. Tamayev, L. Dalkian, A. Sheinfeld, L. Anglister

**Affiliations:** Department of Anatomy and Cell Biology, The Hebrew University Medical School, Jerusalem 91120, Israel

Cholinesterases (ChEs) include acetylcholinesterase (AChE) and butyrylcholinesterase (pseudocholinesterase, BuChE) and are abundant in the nervous system and other tissues. While the role of AChE in terminating cholinergic transmission is well established, the role of BuChE is not clear, although it can substitute for AChE in neurotransmission (eg, in AChE knockout) and acts as a scavenger for anticholinesterases. Recent evidence suggests that both enzymes may function in normal development of the nervous systemand participate in neurodegenerative diseases. Our study examines the involvement of ChEs in the pathologies of dystrophin-deficient mutant (mdx) mice, the animal model of Duchennemuscular dystrophy. We have previously shown that adult female mdx muscles are malformed with distorted neuromuscular junctions and impaired regulation of acetylcholine receptors. Furthermore, the BuChE activity in female mdx sera was significantly lower than in wild-type (wt). Because serum BuChE in rats is modulated by gonadal steroids and is down-regulated via circulating testosterone (T) in males, we tested such an influence in mdx mutants. Sera of adult (17–23 weeks old), male mdx and wt mice were assayed for BuChE activities by hydrolysis of butyryl- and acetyl-thiocholine substrates, using selective inhibitors. We first confirmed that BuChE is also reduced in male mdx sera when compared to wt. Second, to determine the effect of removal of T on male ChE levels, we examined mdx and wt sera 6 days after orchidectomy. While total BuChE activity in wt sera increased by almost 19%, the BuChE levels in mdx sera stayed unchanged after castration, thus remaining significantly below normal wt level and almost 30% lower than wt-castrate. Together, it is possible that the lack of dystrophin in mdx-mice affects the endocrine regulation of circulating BuChE. The endocrine regulation of ChEs is further investigated at the level of neuromuscular junctions in mdx and wt-mice.

*Supported by MDA (LA).*

# Abstract: VOLTAGE-GATED POTASSIUM CHANNELS AND NEURONAL EXCITABILITY IN PROTEIN TYROSINE PHOSPHATASE EPSILON KO MICE

**Authors:** S. Ebner, Z. Tiran, A. Elson, B. Attali

**Affiliations:** ^1^Department of Physiology and Pharmacology, Sackler Faculty of Medicine, Tel Aviv University, Tel Aviv 69978, Israel; ^2^Department of Molecular Genetics, The Weizmann Institute of Science, Rehovot 76100, Israel

Protein tyrosine phosphatase epsilon (PTP epsilon) is strongly expressed in the nervous system; however, little is known about its physiological role. We have previously shown that mice lacking PTP epsilon (PTPe KO) exhibit increased activity of delayed-rectifier, voltage-gated potassium (Kv) channels in sciatic nerve tissue and in primary Schwann cells. In this study we show that PTPe KO mice exhibit a decreased current density of the delayed-rectifier Kv channels in cultured cortical pyramidal neurons. In addition, the extent of C-type inactivation of delayed-rectifier K+ currents was weaker in PTPe KO mice compared to WT. In transfected CHO cells, constitutively active Y527F Src kinase profoundly reduced the delayed-rectifier Kv1.2 and Kv7.2/Kv7.3 currents and cotransfection with PTP epsilon significantly relieved the Src kinase-induced inhibition. The PTP epsilon dependent increase in Kv1.2 current was not observed when a substrate-trappingmutant of PTP epsilon was used. In transfected HEK 293 cells, the levels of tyrosine phosphorylation of Kv1.2 channels is high in the presence of Y527F Src while it remains low upon expression of PTP epsilon. Hence, the effects of PTP epsilon on Kv channels are cell-type specific, inducing a decrease of current in Schwann cells and enhancing the delayed-rectifier current in cortical pyramidal neurons. This PTP dependent enhancement may be partly due to dephosphorylation and modulation of Kv1.2 activity. We are currently investigating whether PTP epsilon plays role in brain excitability through the regulation of delayed-rectifier Kv channels by shaping the firing pattern of pyramidal neurons.Protein tyrosine phosphatase epsilon (PTP epsilon) is strongly expressed in the nervous system; however, little is known about its physiological role. We have previously shown that mice lacking PTP epsilon (PTPe KO) exhibit increased activity of delayed-rectifier, voltage-gated potassium (Kv) channels in sciatic nerve tissue and in primary Schwann cells. In this study we show that PTPe KO mice exhibit a decreased current density of the delayed-rectifier Kv channels in cultured cortical pyramidal neurons. In addition, the extent of C-type inactivation of delayed-rectifier K+ currents was weaker in PTPe KO mice compared to WT. In transfected CHO cells, constitutively active Y527F Src kinase profoundly reduced the delayed-rectifier Kv1.2 and Kv7.2/Kv7.3 currents and cotransfection with PTP epsilon significantly relieved the Src kinase-induced inhibition. The PTP epsilon dependent increase in Kv1.2 current was not observed when a substrate-trappingmutant of PTP epsilon was used. In transfected HEK 293 cells, the levels of tyrosine phosphorylation of Kv1.2 channels is high in the presence of Y527F Src while it remains low upon expression of PTP epsilon. Hence, the effects of PTP epsilon on Kv channels are cell-type specific, inducing a decrease of current in Schwann cells and enhancing the delayed-rectifier current in cortical pyramidal neurons. This PTP dependent enhancement may be partly due to dephosphorylation and modulation of Kv1.2 activity. We are currently investigating whether PTP epsilon plays role in brain excitability through the regulation of delayed-rectifier Kv channels by shaping the firing pattern of pyramidal neurons.

*Supported by ISF (672/05) and the Keren Wolfson Family Funds to the third author.*

# Abstract: NEUROPATHIC PAIN: WHAT IS IT AND WHY IS IT INTERESTING?

**Authors:** E. Eisenberg

**Affiliations:** Pain Relief Unit, Department of Neurology, Rambam Medical Center, Haifa, Israel

According to recent estimates, two million Americans suffer from neuropathic pain. Neuropathic pain may result from a large variety of insults to the peripheral or central somatosensory nervous system such as trauma, inflammation, ischemia, and metabolic and neoplastic disorders. Common examples of peripheral neuropathic pain include diabetic neuropathy, post-herpetic neuralgia (PHN) and trigeminal neuralgia. Central post-stroke pain, pain in multiple sclerosis, and post-spinal cord injury pain are examples of central neuropathic pain. The main clinical characteristics of neuropathic pain are continuous or intermittent spontaneous pain, typically burning, aching, or shooting in quality, and abnormal sensitivity of the painful site to normally innocuous stimuli such as light touch by garments, running water or even wind (allodynia). Neuropathic pain, like many other forms of chronic pain, often negatively effects quality of life. Neuropathic pain is an interesting phenomenon for the following reasons. (a) While blocking pain pathways is an effective way of reducing “normal pain,” paradoxically injuries to pain pathways often produce “abnormal” neuropathic pain. (b) Patients sometimes experience neuropathic pain in an otherwise numb area. (c) Neuropathic pain sometimes spreads beyond the territory of initial nerve injury. (d) Neuropathic pain tends to persist for extended periods (even years). (e) Neuropathic pain tends not to respond to simple analgesics (ie, acetaminophen, nonsteroidal anti-inflammatory drugs), and only partially and unpredictably to antidepressants, anticonvulsants, opioids, and other drugs.

# Abstract: A GAIN MODEL TO EXPLAIN THE STATISTICAL PROPERTIES OF MOTOR CORTICAL TUNING CURVES

**Authors:** B. Engelhard, M. Safran, N. Zach, D. Inbar, Y. Grinvald, E. Vaadia, H. Sompolinsky

**Affiliations:** ^1^Interdisciplinary Center for Neural Computation (ICNC), The Hebrew University, Jerusalem 91010, Israel; ^2^Physiology Department, Hadassah Medical School, The Hebrew University, Jerusalem 91120, Israel; ^3^Racah Institute of Physics, The Hebrew University, Jerusalem 91010, Israel; ^4^Institute of Life Sciences, The Hebrew University, Jerusalem 91010, Israel

What features underlie the statistical properties of neural responses? In this work we have analyzed the directional tuning of single units recorded in motor cortex of monkeys performing a center-out task. We have found distinctive distributions of tuning shape parameters such as mean firing rates and modulation amplitude, and strong positive correlations between them. In order to explain these findings we propose a model that assumes that the synaptic inputs to different neurons are Gaussian sourcesmodulated by independent neuronal gain. The parameters of the Gaussian distributions of sources as well as the shape of the gain distribution were estimated by the expectation-maximization (EM) algorithm. We find that the resultant statistical model accounts for the observed shape of histograms of tuning properties as well as their correlations. We propose that gain modulation plays an important role in the diverse response properties of cortical neurons. Our work offers a useful method of recovering such factors from the observed distribution of responses in cortical populations.

*Supported by Israel Science Foundation, Bundesministerium für Bildung und Forschung, and US-Israel BSF.*

# Abstract: LAMINAR DIFFERENCES IN SENSORY-MOTOR CORTICAL ACTIVITY BETWEEN 6-OHDA AND CONTROL RATS

**Authors:** A. Eshel, G. Goelman

**Affiliations:** Department of Medical Biophysics and Nuclear Medicine, Hadassah Medical School, The Hebrew University, Jerusalem 91120, Israel

Parkinson's disease (PD) is a human neurodegenerative disorder caused by loss of dopaminergic brain cells. The lack of dopamine alters the function of the basal ganglia—cortex circuit, resulting in the well-known symptoms of the disease. We aim to test if the low dopamine levels alter cortical functional architecture in the laminar level. High spatial resolution fMRI data from somato-sensory cortex of unilateral 6- OHDA (a PD rat model) (*N* = 7, 22 sets) and sham operated (*N* = 7, 23 sets) rats were collected. The well-studied forepaw electric stimulation paradigm was used for sensory stimulation. fMRI activity maps have shown higher activity volume and value in the contralateral to the stimulation forepaw (which is also the 6-OHDA lesioned hemisphere) in the sensory cortices of both 6-OHDA and sham rats. However, we found an additional significant difference, in favorite to the same hemisphere, in the 6-OHDA motor cortex while the sham rat did not show any difference. In a deeper inspection of the cortex layers profile, we observed that the main activity volume was centered around layer 4 in the sham rats while in the 6-OHDA rats, stronger activity volume and value were observed in higher layers (around 2-3), decreasing moderately towards deeper layers.

# Abstract: RHYTHMIC MOTOR PATTERNS INDUCED BY ENDOGENOUS AND EXOGENOUS ACETYLCHOLINE IN THE ISOLATED SPINAL CORD OF THE NEONATAL RAT

**Authors:** A. Etlin, E. Finkel, L. Anglister, A. Lev-Tov

**Affiliations:** Department of Anatomy and Cell Biology, The Hebrew University Medical School, Jerusalem 91120, Israel

The ability of the neurotransmitter acetylcholine (ACh) to produce coordinated rhythmic patterns was studied in isolated brainstem spinal cord preparations of neonatal rats, using bilateral recordings from sacral and from flexor- and extensor-dominated lumbar ventral roots, and intracellular recordings from spinal motoneurons. Elevation of the endogenous concentration of ACh by application of the cholinesterase blocker edrophonium (Edro) produced nonstationary rhythmic activity in the thoracolumbar (TL) and sacrocaudal (SC) segments. Cross-wavelet analysis of the data revealed epochs of locomotor-like activity (left-right + flexor extensor alternation, 26% of the preparations) followed either by alternating left-right pattern with flexorextensor synchronicity, bilaterally synchronous rhythm, or irregular bursting patterns. The TL and SC rhythms exhibited strong longitudinal coupling under these conditions. Specific application of Edro/ACh to the TL or SC segments produced bursting activity only in the segments onto which the drugs were added. Stimulation of SC-afferents or the ventromedial medulla in the presence of Edro/ACh produced locomotor-like activity and alternating left-right bursting in the TL and SC segments, respectively. Immunocytochemical studies revealed clusters of cholinergic ventral horn and central canal-adjacent neurons in all studied segments (L2, L5, S1, S2). Cholinergic neurons were also found in the intermediolateral and sacral parasympathetic nuclei of L2 and S1. ACh is suggested to activate various components of the pattern generating circuitry at the TL and SC spinal segments and thereby produce diverse rhythmic patterns. The coupling between the rostral and caudal rhythmogenic networks is achieved only as the excitability of the TL and SC networks is elevated simultaneously. The association between the identified clusters of cholinergic neurons described above and the ACh induced motor patterns will be discussed.

# Abstract: EFFECTIVE CONNECTIVITYWITHIN THE DISTRIBUTED CORTICAL NETWORK FOR FACE PERCEPTION

**Authors:** S. Fairhall, A. Ishai

**Affiliations:** Institute of Neuroradiology, University of Zurich, Switzerland

Face perception elicits activation within a distributed cortical network that includes visual (“core”), as well as limbic and prefrontal (“extended”) regions. We used fMRI at 3T and dynamic causal modeling (DCM) to investigate the functional organization within and between the core and the extended systems. We predicted that, due to the pivotal role of the fusiform gyrus in face perception, ventral rather than dorsal regions of the core system would influence regions of the extended system. We further hypothesized that emotional and famous faces would differentially alter functional coupling within the extended system. Ten subjects viewed various face stimuli (unfamiliar, emotional, and famous faces) while functional images were acquired. Conventional SPM analysis revealed activation in visual, limbic, and prefrontal face-selective regions. DCM analysis revealed that the core system has a hierarchical, feed-forward architecture, with the inferior occipital gyrus separately and directly influencing ventral (fusiform gyrus) and dorsal (superior temporal sulcus) regions. To investigate the interaction between the core and extended system, the amydgala, inferior frontal gyrus, and the orbitofrontal cortex were selected, and models were compared, varying either fusiform gyrus or superior temporal sulcus influence on each region of the extended system. We found that the fusiform gyrus provided the dominant causal influence on the amygdala, inferior frontal gyrus and orbitofrontal cortex. Moreover, analysis of effective connectivity within the extended system showed that emotional faces increased coupling between the fusiform gyrus and the amygdala, whereas famous faces increased connectivity between the fusiform gyrus and the orbitoforntal cortex. Our results demonstrate content-specific dynamic alterations in the functional coupling between visual-limbic and visualprefrontal face-responsive pathways.

# Abstract: “SHAPING BEHAVIOR”: RELATING SHAPE, STRUCTURE, AND FUNCTION IN ONE-DIMENSIONAL NEURAL CULTURES

**Authors:** O. Feinerman, A. Rotem, E. Moses

**Affiliations:** ^1^Weizmann Institute of Science, Rehovot 76100, Israel; ^2^Memorial Sloan-Kettering Cancer Center, Israel

Patterning neural cultures into functional microcircuits [1– 3] has both conceptual implications for integrating single neuron properties into global brain function [4] and importance for brain-computer interfacing. However, two dimensional cultures grown in vitro connect randomly and as yet do not have computation capabilities. By tailoring networks onto a variety of 1D structures [5, 6], we controlled connectivity and shaped the activity patterns, yielding operational transmission lines, threshold devices, logical “ANDgates,” and “diodes.” These neuronal devices constitute an important step towards the design of in vitro microcircuits and the understanding of in vivo ones.

## References

[1] Zeck G, Fromherz P (2001). Noninvasive neuroelectronic interfacing with synaptically connected snail neurons immobilized on a semiconductor chip. *Proceedings of the National Academy of Sciences of the United States of America*.

[2] Chang JC, Brewer GJ, Wheeler BC (2001). Modulation of neural network activity by patterning. *Biosensors & Bioelectronics*.

[3] Wyart C, Ybert C, Bourdieu L, Herr C, Prinz C, Chatenay D (2002). Constrained synaptic connectivity in functional mammalian neuronal networks grown on patterned surfaces. *Journal of Neuroscience Methods*.

[4] Grillner S, Markram H, De Schutter E, Silberberg G, LeBeau FEN (2005). Microcircuits in action—from CPGs to neocortex. *Trends in Neurosciences*.

[5] Feinerman O, Segal M, Moses E (2005). Signal propagation along unidimensional neuronal networks. *Journal of Neurophysiology*.

[6] Feinerman O, Moses E (2003). A picoliter ‘fountain-pen’ using co-axial dual pipettes. *Journal of Neuroscience Methods*.

# Abstract: ANALYSIS OF VOLTAGE SENSITIVE DYE IMAGING DATA OBTAINED FROM A PRIMATE IN DIFFERENT STATES OF AROUSAL BY COMPUTATIONAL TOPOLOGY TECHNIQUES

**Authors:** T. Fekete, D. B. Omer, I. Pitowsky, A. Grinvald

**Affiliations:** ^1^Department of Neurobiology, Weizmann Institute of Science, Rehovot 76100, Israel; ^2^The Interdisciplinary Center for Neural Computation, The Hebrew University, Jerusalem 91010, Israel; ^3^Department of Cognitive Science, The Hebrew University, Jerusalem 91010, Israel

Organisms endowed with complex nervous systems exhibit different states of arousal (alertness, quiet wakefulness, sleep, etc). What are the systematic differences in the cortical activity which constitute such states? We reason that two types of differences should exist: (1) within a state instances of activity should exhibit characteristic structure, as indicated by macroscopic measurements of the cerebral electrical activity (ie, EEG), (2) the structure of the activity sets associated with different states should reflect the difference in the organism's perceptual (and cognitive) capacity given its state of arousal. To explore these ideas we analyze voltage sensitive dye imaging data recorded from a primate in three conditions (1) under anesthesia, (2) with closed eyes, and (3) while viewing visual stimuli. To explore the first question we extract from frames captured during 1- second recordings measures pertaining to characteristic structure, such as measurements of randomness and typical correlation structures. In order to characterize the structure of activity sets we employ different measures to probe the data ranging from quantitative (computation of homology) to more qualitative means which allow for visualization (such as geometric diffusion techniques). We find that given a state, cortical activity does indeed exhibit characteristic structure: activity becomes less random, more regularly distributed in space and time, more correlated, and has typical distribution of spectral energy in specific spatial-temporal bands, as arousal increases (eg, (1)⇒(2)⇒(3)). These phenomena are very robust and thus allow not only perfect classification of activity according to state, but also noticeable confidence margins. Also, we show preliminary results lending some support to the idea that the structure (eg, the topology and geometry) of activity sets is markedly different between the conditions examined.

# Abstract: POLYAMINES: ENDOGENOUS BLOCKERS OF Na CHANNELS IN NEOCORTICAL NEURONS

**Authors:** I. A. Fleidervish

**Affiliations:** Koret School of Veterinary Medicine, The Hebrew University of Jerusalem, Rehovot, Israel

Somata and processes of central neurons contain a mixture of molecularly distinct voltage-gated Na channels. Fastinactivating, transient current through these channels plays a central role in initiation and propagation of action potentials. The same channels give a rise to a more slowly inactivating, persistent Na current, whose functional importance relates to dynamic control of the neuronal input-output relationship. Here, We present the first evidence that the availability of both transient and late Na channels in neocortical neurons is controlled by ubiquitous polyamine (PA) substances, spermine, and spermidine. We also show that while somatodendritic Na channels are extremely sensitive to PA modulation, the axonal channels (presumably Nav1.6) are either less sensitive or resistant. These findings identify a novel mechanism whereby changes in PA metabolism, either associated with normal brain states and stimuli or with pathophysiological conditions, can profoundly influence Na channel availability, and thereby modify neuronal excitability.

*Supported by grants from the Israel Science Foundation and the United States-Israel BSF.*

# Abstract: SYNAPTIC ZINC REGULATES PROTEIN EXPRESSION OF ZNT-1 AND METALLOTHIONEIN I/II IN THEMOUSE BRAIN

**Authors:** M. Forberg, V. Elgazar, T. Kaisman, I. Sekler, Z. Silverman

**Affiliations:** Zlotowski Center for Neuroscience, Ben Gurion University of the Negev, Beer Sheva 84105, Israel

Release of glutamate at excitatory synapses in the mammalian forebrain is accompanied by release of chelatable zinc. The function of this zinc remains enigmatic, and though a role in intracellular signaling has been convincingly demonstrated in recent years. Zinc maintains a complex reciprocal relationship with the proteins that manage it intracellularly, including zinc transporters (ZnT) and metallothioneins (MT), termed here zinc homeostatic proteins (ZHPs). *Objectives*. To determine the role played by synaptic zinc on expression of ZHPs, we assessed relative expressions of ZnT-1 and MT I/II in the brains of ZnT- 3 knockout and strain-matched control mice. Mice missing the gene encoding ZnT-3, involved in the transport of zinc into presynaptic vesicles of glutamatergic neurons, lack synaptic zinc.*Methods*. Autometallography immunohistochemistry/ fluorescence Western blot analysis and densitometric analysis. *Results*. Immunofluorescence for ZnT-1 was significantly reduced in neurons in hippocampus (CA3,DG), OB (mitral, periglomerular), and cerebellar Purkinje cells of ZnT-3 KO mice from P19 compared to wild-type controls. Roughly 50% of control values for strongly labeled Purkinje cells were observed in the cerebellum and in the OB. Numbers of MT I/II-labeled astrocytes were also significantly reduced in the brains of the ZnT-3 KO mice. Western blots indicated a 4–50% reduction in ZnT-1 in all regions examined. *Conclusions*. The reduction in immunoreactive ZHPs observed in ZnT-3 KO mice indicates that while not required for initial expression of ZnT-1 and MT I/II, synaptic zinc is required for achievement or maintenance of normal levels of both proteins in the mouse brain.

*Supported by ISF Grant no 572/04 to the fifth author.*

# Abstract: CB1 RECEPTOR BLOCKADE IN NEWBORN MICE PREVENTS SUCKLING: RECEPTOR MECHANISM AND ORAL-MOTOR COMPETENCE

**Authors:** E. Fride, H. Braun, N. Arshavsky, H. Dahan, H. Seltzman

**Affiliations:** ^1^Department of Behavioral Sciences, College of Judea and Samaria, Ariel, Israel; ^2^Department of Molecular Biology, College of Judea and Samaria, Ariel, Israel; ^3^RTI International, Research Triangle Park, NC, USA

We have shown previously that blockade of the cannabinoid CB1 receptor in newborn mice interferes with the initiation of milk suckling and thus severely impairs growth and development. These studies utilized the CB1 receptor antagonist/ inverse agonist rimonabant (SR141716) and suggested that an oral-motor deficit underlies the impaired suckling. However, generalmotor development was also affected by rimonabant. Here, we extend our observations (1) on the role of the sucking mechanism in the growth deficit and (2) to affirm neutral blockade of the CB1 receptor, as opposed to an inverse agonism mechanism by the use of a novel, neutral CB1 antagonist. Experiment 1. Pups were injected with rimonabant (10–20 mg/kg). In order to investigate whether CB1 receptor blockade impairs sucking, independently of a generalmotor deficit, neonates were placed in a dish containing amilk/creammixture. Growth and survival were now significantly improved in rimonabant-treated pups. Experiment 2. Pups were injected with HS1 (12.5–25 mg/kg) and were assessed daily for weight gain, milk ingestion, and body temperature. Significant developmental delays were recorded in the HS1 pups. Conclusions. (a) Neutral CB1 receptor antagonism is responsible for the rimonabant/HB1-induced growth failure and (b) oral motor competence is the psychobiological mechanism underlying the impaired feeding in the CB1 receptor-blocked pups. Further, “Nonorganic failureto- thrive” (NOFTT) afflicts 2–4% of infants and is characterized by growth failure without any known organic cause. Researchers have hypothesized a “biological vulnerability” leading to hypotonia, deficient oral-motor function and impaired suckling. Our studies suggest that the syndrome observed in newborn pups with blocked CB1 receptors may serve as the first animal model for NOFTT.

*Suppported by Danone Research Institute Israel; by CJS fellowships to the second, third, and fourth authors; and by EF Internal CJS grant.*

# Abstract: REVERSAL OF MEMORY LOSS BY LIPOPHILIC MEMORY MODULATORS IN THE 3XTGAD TRANSGENIC MOUSE MODEL OF ALZHEIMER'S DISEASE

**Authors:** J. E. Friedman, V. K. Madala Halagappa, M. P. Mattson, A. Kozak

**Affiliations:** ^1^D-Pharm, Ltd, Rehovot, Israel; ^2^Laboratory of Neuroscience, National Institute on Aging/NIH, Baltimore, MD, USA

Transition metals such as zinc and copper contribute to the pathologies inherent in Alzheimer's disease (AD), including APP processing, amyloid-beta aggregation and release of cytokines. We have shown that the membrane active metal chelator (MAC) DP-109 inhibits plaque formation in aged female APP-transgenic Tg2576 mice [1], as well as reduces the release of Il-1b and TNFa from microglia. The question remains as to whether such treatment can reduce or prevent memory loss? We tested a triple transgenic mouse model (3xTgAD) of AD, expressing mutations in presenilin- 1, APP (Swedish mutation) and tau (P301L), at 5–9 and 14–18 months of age. Mice were administered either vehicle, DP-109 (5 mg/kg/day), or DP-460 (10mg/kg/day), po starting at 5 or 14mo of age for 3 months, followed by Morris water maze testing (MWM) while continuing treatment. Nine mo mice learned to find the hidden platform in the MWM with no memory impairment nor any significant difference between the groups, whereas 18 mo 3xTgAD mice did demonstrate significant memory acquisition impairments. On the fourth day of the MWM trial, mice treated with the MAC molecules demonstrated a significantly improved ability to find the hidden platform as compared to the naïve and vehicle-treated mice, 20 ± 4 s versus 37 ± 4.6 s, *P* < .01, respectively. By the fifth day of testing DP-109 and DP-460 treated mice acquired the hidden platform as effectively as did the 9 mo old mice. Performance in the probe trial task was also significantly improved in aged mice treated with DP-109 and DP-460. The swimming speed remained fairly constant across the groups. These results further support the idea that endogenous metals contribute to the pathology of AD and that administration of therapeutic agents, even after the reported onset of AD pathology, can reverse progressive memory loss in 3xTg-AD mice. Lipophilic metal chelators such as DP-109 and DP-460 appear to be promising therapeutic agents against AD.

*The first and fourth authors are employees of D-Pharm, Ltd. This work was supported by NIH Intramural Funding.*

## References

[1] Lee JY, Friedman JE, Angel I, Kozak A, Koh JY (2004). The lipophilic metal chelator DP-109 reduces amyloid pathology in brains of human beta-amyloid precursor protein transgenic mice. *Neurobiology of Aging*.

# Abstract: DOC2B MODULATES THE EXOCYTOTIC RESPONSE IN A CALCIUM-DEPENDANT MANNER

**Authors:** R. Friedrich, A. J. Groffen, O. Gutman, Y. I. Henis, M. Verhage, U. Ashery

**Affiliations:** ^1^Department of Neurobiochemistry, The George S Wise Faculty of Life Sciences, Tel Aviv University, Israel; ^2^Department of Functional Genomics, Center for Neurogenomics and Cognition Research, The Netherlands

Calcium-dependent exocytosis is regulated by a vast number of proteins. The aim of our work is to investigate the role of DOC2 in the exocytosis process. DOC2 protein family contains 3 isoforms which have two tandem C2 domains; C2 domains associate with phospholipids in Ca2+-dependent manner. We use adrenal chromaffin cells as our model system for neurosecretion and overexpressed DOC2 in these cells using the Semliki Forest Virus system. Both DOC2A and -B have high calcium sensitivity; they translocated from the cytosol to the plasma membrane at extremely low [Ca2+]i, half-maximal translocation occurred at 478 nM and 175 nM [Ca2+]i for DOC2A and -B, respectively. Thus, DOC2A and -B are extremely sensitive to small changes in [Ca2+]i. These changes occur when the vesicles in the neurosecretory cell undergo priming. We hypothesized that DOC2 affects exocytosis and that its translocation has a physiological role. Therefore we created a mutant DOC2B that is constantly at the plasma membrane. To check the differences between the effect of the wt and the mutant DOC2B on the exocytotic response, we performed amperometry experiments where we depolarized the cells using KCl in a repetitive manner. Both wt andmutant DOC2B enhanced exocytosis in the first stimulation. Yet, while the wt DOC2B increased the exocytotic response in a constant manner throughout the six applications, the ability of the mutant DOC2B to increase the exocytotic response decreased within each application. This suggests that translocation of DOC2B is important for its activity. To pinpoint the exact step where DOC2B is involved, we used membrane capacitancemeasurements and flash photolysis of caged-Ca2+. We found that the wt DOC2B increased the fast burst, while the mutant DOC2B had a smaller effect on the fast burst. The second flash revealed that while the wt DOC2B increased the effect, the mutant did not differ from control cells. Our results suggest that DOC2B modulates the exocytotic response.

# Abstract: BRAIN NETWORK SUPPORTING LONG-TERM MEMORY FORMATION UNDER REAL-LIFE-LIKE VIEWING CONDITIONS

**Authors:** O. Furman, U. Hasson, D. Clark, Y. Dudai, L. Davachi

**Affiliations:** ^1^Department of Neurobiology, Weizmann Institute of Science, Rehovot 76100, Israel; ^2^Department of Psychology and Center for Neural Science, New York University, USA

Episodic memory formation and its neural correlates are commonly studied using paradigms employing individual trials devoid of a continuous context outside of the laboratory setting. In contrast, real-life episodic memory is the result of on-going encoding within a highly contextualized and dynamically changing framework. In order to investigate human Memory under conditions that more closely mimic reallife encoding situations, we had subjects view on audio-visual TV sitcom while undergoing fMRI scanning and eye-motion monitoring. Three weeks later, a subsequent memory test, comprised of 77 questions targeting unique narrative events dispersed evenly (∼20 s) throughout the movie, was administered. Behavioral measures indicate that subjects' memory performance for details of the narrative that were probed was very good (∼68% correct). To analyze the fMRI data, we used linear modeling and a novel analysis based on intersubject correlation to isolate regions relevant to free viewing (Hasson et al, 2004) and regions correlated with later memory performance. Specifically, we created maps revealing activated voxels that are more correlated across all of our subjects when subjects later recall or recognize that portion of the sitcom compared to when they do not remember it. First, we replicate the results of Hasson et al (2004). Second, our analysis reveals a network of brain regions in which the intersubject correlations based on later memory outcome are distinct from those that show correlations when memory formation is not considered. Convergent, yet interestingly different results were obtained using the more conventional general linear modeling. These differences will be discussed. These data extend traditional subsequent memory findings of differential activity in task-relevant regions to a rich and dynamic situation that is more ecologically valid than more traditional experimental learning paradigms.

*Supported by the NYUWeizmann Institute Collaborative Fund in the Neuroscience (to the fifth and fourth authors) and an HFSP long-term fellowship (to the second author).*

# Abstract: A “DEPRESSIVE-LIKE” STATE INDUCED IN COCKROACHES BY AWASP'S STING INTO BRAIN

**Authors:** R. Gal, T. Gavra, T. Libersat

**Affiliations:** Zlotowski Center for Neuroscience, Ben Gurion University of the Negev, Beer Sheva 84105, Israel

The parasitoid wasp *A compressa* stings a cockroach and injects a venom cocktail directly into its brain. The sting dramatically reduces motor activity and the stung cockroach fails to initiate spontaneous locomotion and escape responses to tactile stimuli. Although the stung cockroach is not paralyzed, it appears to have lost the motivation to initiate movement as if in a depressive-like state. To explore this idea, we compared stung to control cockroaches in two behavioral paradigms. First, by applying electric shocks of measurable amplitude, we show that the threshold voltage required to elicit escape is significantly higher in stung cockroaches. This elevated threshold can be only partially explained by an unspecific anesthesia since the startle (nociceptive) response to the shock is modulated to a lesser extent. Second, by submitting cockroaches to the forced swimming test, we show that swimming duration is significantly reduced in stung cockroaches. Yet, during swimming episodes, stung cockroaches show normal interleg coordination, stepping frequency range and correlation between stance duration, discharge rate of legmuscles, and cycle frequency.However, for a given stepping frequency, the discharge rate of leg muscles is decreased in stung cockroaches, suggesting a reduction in excitability of motor centers. These results suggest that the unique venom of *A compressa* manipulates central mechanisms involved in modulating the “motivational” state of its insect prey. Furthermore, this behavioral manipulation can be analyzed and quantified using established paradigms in mammalian research.

# Abstract: NEURORESCUE (NEUROGENESIS) AND LIMITED TYRAMINE POTENTIATION BY THE NOVEL MULTIFUNCTIONAL ANTI-PARKINSON'S DRUG, M30 [5-(N-METHYL-N- PROPARGYAMINOMETHYL)-8- HYDROXYQUINOLINE]

**Authors:** S. Gal-Benari, H. Zheng, M. Fridkin, M. B. H. Youdim

**Affiliations:** ^1^Eve Topf Center, Faculty of Medicine, Technion–Israel Institute of Technology, Haifa 32000, Israel; ^2^TheWeizmann Institute of Science, Rehovot 76100, Israel

M30 is a propargylamine-derived multifunctional neuroprotective drug, in vitro and in vivo, with iron chelating-radical scavenging and selective brain monoamine oxidase (MAO)- A and B inhibitory activity. In culture studies it rescues SHSY5Y cells from apoptotic cell death induced by serum withdrawal and induces GAP-43 and neurite proliferation. In the present study, its in vivo neurorescue activity has been studied in chronic doses (0.5, 1, 2.5 mg/kg/day, 14 days) administered orally to mice subsequently to MPTP (4 × 24 mg/kg). It significantly rescued cell function of striatal dopaminergic neurons as indicated by dopamine elevation and decrease in the levels of its metabolites, dihydroxyphenyl acetic acid, and homovanilic acid at all doses examined. Furthermore, increased compensatory tyrosine-hydroxylase activity was noted. In these regards it behaves very similarly to the established neurogenesis activity of rasagiline, which involves activation of tyrosine kinase receptor signaling pathway, resulting from its propargyl moiety and not MAO inhibition. One of the limitations of MAO inhibitors as antidepressants or anti-Parkinson's drugs is their ability to potentiate the cardiovascular effect of tyramine, resulting from inhibition of systemic MAO-A. M30 (5, 10 mg/kg IP) selectively inhibited brain (striatum and hippocampus) MAO-AB by more than 85% and raised striatal levels of dopamine, noradrenaline and serotonin, with little inhibition of liver and small intestine enzymes. In contrast to the nonselective MAO inhibitor tranylcypromine (10 mg/kg IP), which fully inhibits MAOAB in the brain and systemic organs and potentiates tyramine cardiovascular effect, M30 exhibited a limited effect. Its tyramine potentiation limitation, the ability to raise brain levels of aminergic neurotransmitters, and its neurorescue activity in the MPTP model make this drug potentially an important agent for prevention and treatment of PD and depression.

# Abstract: IS THE OPIOID SYSTEM INVOLVED IN COCKROACH HYPOKINESIA INDUCED BY A PARASITOIDWASP?

**Authors:** T. Gavra, R. Gal, F. Libersat

**Affiliations:** Department of Life Sciences and the Zlotowski Center for Neuroscience, Ben Gurion University, Beer Sheva 84105, Israel

Opioid receptors and their endogenous ligands are known to exist in insects. Opioid-like substances have a wide range of effects, from “analgesia” and modulation of the threshold for escape, to feeding and heart rate variability. A cockroach stung by the wasp *A compressa*, although not paralyzed, becomes hypokinetic and fails to initiate escape responses to nociceptive stimuli. The venom is injected directly into the cockroach cephalic ganglia, and its effect takes place 30 minutes after the sting and persists for a few days. To explore the possibility that the venom induces hypokinesia by manipulating opioid systems, we first injected cockroaches with the nonspecific opioid antagonist Naloxone and tested their responses to electric shocks of different amplitudes. Naloxone induced a minor, dose-dependant “analgesic” effect persisting for two hours. However, escape responses were readily initiated in these cockroaches when the nociceptive threshold was reached. Next, we injected cockroaches with Naloxone just prior to stinging by *A compressa*. In contrast to stung cockroaches injected with saline, stung cockroaches treated with Naloxone readily initiated escape responses similar to control cockroaches injected with Naloxone. Furthermore, this effect disappeared after two hours, consistent with the time course of Naloxone's effect alone. Thus, the opioid antagonist delayed the effect of the venom on threshold for escape. These results suggest that the venom of *A compressa* may act via opioid systems in the CNS of its cockroach prey to induce hypokinesia. If true, to our knowledge, this would be the first evidence of a behavioral manipulation of one insect by another mediated via the opioid system.

*Supported by BSF Grant 2001044.*

# Abstract: THE CONNECTION BETWEEN EATING BEHAVIOR AND THE ESTROUS CYCLE IN OLETF RATS LACKING CCK-1 RECEPTORS

**Authors:** V. M. Gelbar, M. Schroeder, A. Krause, A. Weller, O. Zagoory-Sharon

**Affiliations:** Bar-Ilan University, Ramat Gan, Israel

The OLETF rat has been extensively studied as a model of hyperphagia, obesity, and diabetes mellitus. Different animal models of obesity exhibit a correlation between obesity and infertility. These rats exhibit disruptions in their estrous cycles that are expressed by longer cycles and less days of ovulation. Furthermore, it is known that eating behavior of females varies across the estrous cycle, also because of fluctuations in levels of estradiol and its interaction with CCK. Previous studies, on different strains, have shown that weight loss is associated with the return of different fertility functions, and the normalization of the estrous cycle. The purpose of the present study is to better understand the influence of obesity on the estrous cycle of the OLETF rats, from a developmental point of view, beginning at puberty until early adulthood. In addition, we are assessing the eating behavior of the rats across the different stages of the estrous cycle. This was of special interest because of the unique mutation (lack of CCK-1 receptors) of our rats. The first group consists of LETO females fed ad libitum (control). The second group consists of OLETF females fed ad libitum. The third group of rats is undergoing weight normalization through food restriction to the amount of food eaten by the control groupthe LETOrats (pair feeding). The fourth group of OLETF has permanent access to running wheels. A fifth group controls for the effect of exercise on the estrous cycle and intake, in the LETO rat. Preliminary results on the effect of weight loss on the estrous cycle and eating behavior will be presented.

*Supported by US-Israel BSF.*

# Abstract: ANALYSIS OF PAR6C GENE EXPRESSION AND SCREENING OF PAR6C-NULL MICE FOR BEHAVIORAL ABNORMALITIES

**Authors:** S. Gelkop, H. Karpman, H. Golan, N. Isakov

**Affiliations:** ^1^Department of Microbiology and Immunology, Ben Gurion University of the Negev, Beer Sheva 84105, Israel; ^2^Department of Developmental and Molecular Genetics, Ben Gurion University of the Negev, Beer Sheva 84105, Israel

The multimeric complex that includes Par6, Par3, and atypical protein kinase C (aPKC) is evolutionarily conserved and functions in various cell polarization events in a wide range of organisms. It is essential for early development of many tissues where it tags subcellular domains at locations in which polarization takes place. In order to learn about the in vivo function of the murine Par6C gene and to test its role in embryonic tissue development, we have generated Par6C null mice by introducing the â-galactosidase reporter gene into the Par6C locus. Reporter expression analysis of the whole mount of brains of Par6C-/- mice revealed that Par6C is expressed in restricted areas within the brain, predominantly hippocampus and cerebellum, suggesting the potential involvement of Par6C in motor functions and learning and memory. No staining was detected in brain sample of wildtype mice. Within the hippocampus, Par6C was expressed predominantly at CA1, CA2, and the dentate gyrus. Staining of the Cerebellum was detected predominantly at the granular layer. Par6C expression was observed also at the Pontine nuclei and several other brain regions. Par6C mice showed no obvious abnormal physical, developmental, or neurological features. For behavioral screening we tested the mice for locomotion activities using the open field test, anxietyrelated responses in-light dark exploration test, motor skills using balance beam and vertical pole assay. Significant differences between wt and Par6C-/- mice were obtained at the open field assay where the Par6C-/- mice displayed decreased locomotor activity. Motor coordination of the Par6C-/- mice was also significantly impaired as detected using the vertical pole assay. While normal mice stayed on the pole and walked up the entire length of the pole, the Par6C-/- mice were less motile, tended to walk down the pole, and fell down after relatively short time periods, suggesting that Par6C may be involved in motor coordination and balance.

# Abstract: THE ANTIDEPRESSANT EFFECT OF SUBCONVULSIVE ELECTRICAL STIMULATION OF THE PREFRONTAL CORTEX AS COMPARED TO ELECTROCONVULSIVE THERAPY

**Authors:** R. Gersner, I. Musseri, D. Taliaz, A. Zangen

**Affiliations:** Weizmann Institute of Science, Rehovot 76100, Israel

Electroconvulsive therapy (ECT) is the most effective treatment for depression. Despite the great efficacy of this treatment it is not widely used due the associated adverse side effects (eg, short term memory loss). Although it has been used for almost a century, the exact mechanism of its action is not clear. ECT stimulates the whole brain, however it is likely that only specific brain regions are responsible for mediation of the therapeutic effect. Our aim was to test whether subconvulsive electrical stimulation (SCES) of the prefrontal cortex can induce antidepressant effects and alter levels of BDNF in reward-related brain regions. We used the chronic mild stress (CMS) model in rats, which induces depressive behaviors, followed by repeated (10 days) application of ECT or SCES delivered to specific brain sites by unilateral (left side) implantation of an electrode. Each treatment group was compared to a sham stimulated control group. Anhedonia (sucrose preference), exploration and home-cage locomotion were measured after treatment. Rats were sacrificed and punches of various brain regions were taken for BDNF evaluation by ELISA. Both ECT and SCES of the prefrontal cortex had therapeutic effect in some behavioral tests and induced changes in BDNF levels. However, only ECT induced impairment in short-term memory. These results suggest that subconvulsive electrical stimulation is as effective as ECT without its side effect and point to the important role of the PFC in the pathology of depression. Finding of brain regions in which SCES is effective in treating depressive behavior may advance development of new therapeutic strategies such as deep transcranial magnetic stimulation.

# Abstract: TWO DIFFERENT STRATEGIES TO ACCESS SEMANTIC KNOWLEDGE

**Authors:** A. Gigi, K. Shultz

**Affiliations:** ^1^Department of Behavioral Sciences, College of Judea and Samaria, Ariel 44837, Israel; ^2^The Joseph Sagol Neuroscience Center, Sheba Medical Center, Tel Hashomer 52621, Israel

People are known to differ in their cognitive style (eg, visual/ semantic). The current research explored whether these two cognitive styled people use different approaches in retrieving information from semantic knowledge. Nineteen participants (aged 13–50) completed two computerized tests: the first is naming test that includes 40 objects pictures; half of them are pictured from a conventional viewpoint (usual) and other half pictured from unconventional viewpoints (unusual). Participants were instructed to name the object as fast as they can. Answers and reaction times (RT; ms) were recorded (Gigi et al [1]). The second test included 20 stimuli; each has four items with two extraordinary items, one functional and the other is visual. Participants were instructed to choose one extraordinary item out of the four. Answers and RT were recorded. For each participant a semantic index was created (according to his/her visual/functional extraordinary selections) and each participant was noted as having either a “visual” or “semantic” style. Accordingly, the sample was splited into two groups: 13 semantic and 6 visual. Mean age was similar in the two cognitive styled groups. In addition, there was no difference in the percent of the correct answers between groups in the naming test (usual or unusual conditions). However the “semantic” group responded significantly faster than the “visual” one in the two tests. According to Evans dual task theory [2], we assume that there are two possible strategies for accessing the “semantic” knowledge. The “semantic strategy” uses more automatically processes while the “visual strategy” uses imaginary and conscious processes.

*The study was supported by Samaria and Jordan Rift R & D Center.*

## References

[1] Gigi A, Babai R, Katzav E, Atkins S, Hendler T Prefrontal and parietal regions are involved in naming of objects seen from unusual viewpoints.

[2] Evans JS (2003). In two minds: dual-process accounts of reasoning. *Trends in Cognitive Sciences*.

# Abstract: JEWISH RELIGIOUS BELIEF: IS IT ENOUGH TO REDUCE ANXIETY?

**Authors:** A. Gigi, M. Papirovitz

**Affiliations:** ^1^Department of Behavioral Sciences, College of Judea and Samaria, Ariel, Israel; ^2^The Joseph Sagol Neuroscience Center, Sheba Medical Center, Tel Hashomer 52621, Israel

Several studies have shown a correlation between the degree of religiousness and the intensity of symptoms in patients with obsessive-compulsive disorder (OCD). In these cases, a similarity between religious practices (rituals) and compulsive rituals in OCD patients was suggested (Greenberg et al [1]). In addition, studies have found that varied aspects of religiousness often correlate with better mental health and lower-anxiety levels (Gigi et al [2]). However, these studies did not distinguish religious belief from religious rituals. In the current study, we inquired healthy subjects; all were without psychiatric or neurological diagnosis (by self report). We used Spielberger questioner [3] to assess level of anxiety, and a religiousness questioner that included 25 items covering two domains of religiosity: religious belief and religious rituals. A significant correlation was found between level of religious faith and level of religious rituals. Nevertheless, a negative correlation was found between level of anxiety and the participants' strictness for Jewish religious rituals. No significant differences were found between anxiety and religious belief. To the best of our knowledge, this is the first study to separate religious belief and religious practices and to show association between level of anxiety and religious rituals. Accordingly, we suggest that Jewish religious rituals can reduce anxiety levels in healthy people.

## References

[1] Greenberg D (1984). Are religious compulsions religious or compulsive: a phenomenological study. *American Journal of Psychotherapy*.

[2] Gigi A, Papirovitz M, Masika H Memory functioning following terror attack and the suggested immunity power of religious faith.

[3] Spielberger CD, Gorsuch RL, Lushene RE (1970). *STAI Manual*.

# Abstract: REGIONALLY SPECIFIC ADAPTATION DYNAMICS IN HUMAN VENTRAL STREAM VISUAL AREAS

**Authors:** S. Gilaie-Dotan, Y. Nir, R. Malach

**Affiliations:** Neurobiology Department, Weizmann Institute of Science, Rehovot 76100, Israel

The phenomenon of fMR-adaptation (fMR-A), that is, reduced activity to a repeatedly presented image, has been observed in high-level object areas, and serves as a tool to reveal traits of underlying neuronal populations. Here we tested the dynamics of fMR-A, and investigated whether they are determined by cortical region, or depend on the type of stimuli being viewed. Nine healthy subjects underwent fMRI scans (GE 1.5T), while viewing a single grayscale image (either face or house) for 21 s followed by a fixation period of 21 s. Subjects were engaged in a demanding attentional task, reporting minute contrast changes in the stimuli that rarely occurred. Category-selective regions and retinotopic borders were defined separately. Our results show a clear differentiation in adaptation dynamics within the ventral stream. Face-selective regions, both ventrally (FFA) and more dorsally (OFA) showed a moderate initial adaptation effect followed by a sustained level of activity for both face and house images. In contrast, the house-related CoS region showed a rapid initial decline followed by sustained activity for houses, while dropping essentially to baseline for faces. The object-related LO exhibited slower continuous adaptation decline throughout the block for both categories. Interestingly, within the regions, the decline of the signal from the peak activation did not depend on the viewed category (preferred or nonpreferred). Thus, our results demonstrate that the functional differentiation in ventral stream regions is evident not only in their functional selectivity but also in their adaptation dynamics. Since the more peripherally-biased, house-related CoS region showed a sharp clear-cut drop in its activity levels, compared to the more foveally-biased face- and object-related regions, we would like to propose that the neuronal computational mechanisms within object-selective cortex are driven by center-periphery organization, and not by category-specific features.

*Supported by ISF Center of Excellence, The Clore Center, The Dominique Center, and The Moscona Fund.*

# Abstract: ALTERATIONS IN RAT BRAIN mRNA LEVELS FOLLOWING SARIN EXPOSURE

**Authors:** V. Givant-Horwitz, I. Rabinovitz, J. Kapon, E. Gilat

**Affiliations:** Department of Pharmacology, Israel Institute of Biological Research, Ness-Ziona, Israel

Convulsions are common features of organophosphates (OP) exposure. The initiation and the early phase of OPinduced seizures are predominated by cholinergic mechanisms and the propagation and the maintenance are mainly noncholinergic. In the present study, the changes in mRNA levels of acetylcholinesterase (AChE), choline acetyltrasnsferase (ChAT), glutamic acid decarboxylase (GAD) 65 and 67, bax and bcl-2 were measured in rat hippocampus and cortex, 2 hours and 24 hours post sarin exposure, using RT-PCR. The effects of antidotal treatments including TMB-4 and atropine (TA) 1min post sarin, alone or with the addition of immediate (1 min post sarin) or delayed (15min post sarin) midazolam were evaluated as well. Two hours following sarin, the hippocampal mRNA levels of all the genes, except for GAD67, were reduced in convulsive untreated animals. At 24 hours post exposure, elevations of hippocampal mRNA levels of ChAT, GAD67, and bax, increments of cortical mRNA levels of GAD65, GAD67, and bax, together with significant loss of body weight and poor physical conditions, were observed in the untreated rats. Similar results were obtained in rats treated with TA alone. When immediate midazolam was added, mRNA levels and clinical conditions were as in naïve animals. However, if midazolam was delayed, mRNA levels and clinical conditions varied. These findings imply that in the hippocampus, 24 hours post sarin exposure, there are requirements for acetylcholine (ACh) and gama-butyric acid (GABA) synthesis, and possible cell death process, reflected by the elevation in mRNA levels of ChAT, GAD67, and bax, respectively. Similar indications for ACh and GABA requirements were obtained when inadequate treatment, TA alone, was used. GABA deficiency and cell death process were indicated in cortex at 24 hours post sarin in cases of inadequate treatments. Overall, these findings may lead to the development of novel therapeutic options for cases of insufficient early treatments.

# Abstract: DIFFERENTIAL GENE EXPRESSION IN THE FETAL CEREBELUM FOLLOWING MATERNAL HYPOXIA AND MAGNESIUM SULFAT LOAD

**Authors:** H. M. Golan, T. Levav, V. Caspi, R. J. Perez-Polo

**Affiliations:** ^1^Developmental Genetics and Zlotowski Center for Neuroscience, Ben Gurion University, Beer Sheva 84105, Israel; ^2^Bioinformatics Core Acility, NIAN, Ben Gurion University, Beer Sheva 84105, Israel; ^3^Department of Biochemistry and Molecular Biology, UTMB, Galveston, TX, USA

Prenatal hypoxia ischemia is a major cause of neurodevelopmental impairment in the newborn, with life span motor and intellectual outcomes. It was suggested that MgSO4 (Mg) protects the fetus from excitotoxic brain injury caused by hypoxia. In our previous studies in a mouse model of maternal hypoxia (H) we have shown thatMg partially prevents damage to the Purkinje cells and the granular layer in the cerebellum. In the current study we apply gene chipmethodology in order to identify genes affected by maternal H and Mg. This was tested in the cerebellum of mice fetus (embryonic day 17), 2 hours after H. Gene expression was analyzed using Affymetrix Gene Chips (mo 430 2.0). Comparison of gene expression in the control group (saline treated followed by exposure to atmosphere air) to H group (saline, followed by 2 hours exposure to 9% oxygen and 3% CO2) revealed that the expression of 124 genes was modified (*P* < .01). H induced upregulation of 40 genes and downregulation of 84 genes. Maternal pretreatment with Mg (4 hours, followed by 2 hours H) altered the expression of 103 genes compared to the control (*P* < .01). Of these genes, 75 were upregulated and 28 downregulated. Only 15 similar genes were modified in these two comparisons, indicating that Mg prevented the majority of changes stimulated by H but on the other hand induced robust alteration in other genes. Two genes involved in neuron proliferation and migration were further tested. A significant increase in the levels of 14-3-3e protein was observed in the cerebellum 2 hours after H, with or without Mg pretreatment (*P* < .01). In the hippocampus, H and MgH increased the expression of 14-3-3e mRNA (*P* < .05), however, this was not translated to changes in 14-3-3e protein. In addition, an increase was observed in CDC42 protein in the cerebellum following H (*P* < .05) and was prevented by Mg. In the hippocampus a tendency of decrease in CDC42 was induced by H. Overall, H and Mg modify a different set of genes related to neurogenesis.

*Supported by BSF Grant no 2003166.*

# Abstract: DATA-DRIVEN CLUSTERING REVEALS A FUNDAMENTAL SUBDIVISION OF THE HUMAN CORTEX INTO TWO GLOBAL SYSTEMS

**Authors:** Y. Golland, P. Golland, S. Bentin, R. Malach

**Affiliations:** ^1^Department of Psychology, The Hebrew University of Jerusalem, Jerusalem 91010, Israel; ^2^Department of Neurobiology, Weizmann Institute of Science, Rehovot 76100, Israel; ^3^Computer Science and Artificial Intelligence Laboratory, MIT, Cambridge, MA, USA

It is clear that major neuroanatomical organizations—apart from their anatomical importance—also signify fundamental functional distinctions and specializations.Here, in search for such global organizational principles, we applied the well-known unsupervised clustering algorithm (*K*-means) to fMRI data collected from several different experiments. The experiments included continuous audio-visual movie, a block-design visual experiment, an experiment involving internal mental tasks in the absence of input/output, and a rest-state scan. The analysis was performed on the entire cortical space and was limited to two-class subdivision. Our results show that the entire cerebral cortex is naturally partitioned into two global systems. Importantly, this fundamental divide was highly consistent anatomically across different subjects. This clustering-based division of the cortex into a bipartite organization is in a good agreement with our previous study which was based on different methods and paradigm. In that study we suggested that the human posterior cortex can be divided into two global systems: one dealing with the external environement—the “extrinsic” system, and one dealing with internal—endogenous information— the “intrinsic system.” The intrinsic system shows substantial anatomical similarity to the “default-mode” network, which was shown to be deactivated by task-oriented paradigms by several studies in the literature. To summerize, we show here, using an objective, data-driven clustering approach, that the human cerebral cortex has a fundamental “bi-partite” organization consisting of two complementary global systems, dealing with the internal and external worlds.

*Supported by ISF COE, Dominique Center (to the fourth author), and NIMH Grant no R01 MH 64458 (to the third author).*

# Abstract: DELAY, STUTTERING, AND SUBTHRESHOLD OSCILLATIONS IN FAST-SPIKING CORTICAL INTERNEURONS

**Authors:** D. Golomb, K. Donner, L. Shaham, D. Shlosberg, Y. Amitai, D. Hansel

**Affiliations:** ^1^Department of Physiology and Zlotowski Center for Neuroscience, Ben Gurion University, Beer Sheva 84105, Israel; ^2^Department of Neurophysics and Physiology, CNRS, France

Cortical fast-spiking (FS) interneurons display diverse firing patterns, such as a prolonged delay followed by highfrequency firing in response to just suprathresold step applied current, stuttering (rhythmic bursting), and subthreshold noisy oscillations. We hypothesize that the various patterns are generated by an interplay between a slowlyinactivating d-type potassium current and the window current of the sodium current, namely the overlap between the activation and inactivation curves of this current. To test this hypothesis, we construct a minimal, conductance-based model of FS cells and investigate its behavior using the fastslow technique and bifurcation theory. When the sodium window current is small, the model neuron fires at a relatively high rate following the delay, the subthreshold voltage varies almost linearly during firing periods, the delay duration depends strongly on noise, and noisy subthreshold oscillations appear. The neuron may stutter if the conductance of the d-current is strong enough. When the sodium window current is large, the neuron may fire at low rates, the subthreshold voltage during firing shows inflection points, there are no subthreshold oscillations and no stuttering, and the delay duration is almost noise-independent. Intracellular recording reveal examples for the two classes of firing patterns. We conclude that biological heterogeneity in the properties of ionic channels, together with dynamical system properties, can account for the distinct firing patterns FS interneurons exhibition.

# Abstract: HUNTINGTIN INTERACTING PROTEIN 1 (HIP1) IN CLATHRIN-MEDIATED ENDOCYTOSIS AND SYNAPTIC VESICLES RECYCLING

**Authors:** I. Gottfried, O. Yizhar, S. Waelter, E. Wanker, U. Ashery

**Affiliations:** ^1^Department of Neurobiochemistry, Tel Aviv University, Tel Aviv 69978, Israel; ^2^Neuroproteomics, Max Delbrueck Center for Molecular Medicine (MDC), Berlin-Buch, Germany

Huntington's disease (HD) is a late-onset progressive neurodegenerative disorder caused by an expansion of a poly-Q tract in the huntingtin (Htt) protein. In an attempt to reveal Htt function, several studies have tried to identify interacting proteins. One of these is the Htt-interacting protein 1 (HIP1). So far it has been shown that HIP1 is related to the process of clathrin-mediated endocytosis (CME), however, the precise step in which it participates and its exact role are still a debate. CME is important formany central cell functions, and in neurons it has a central role in recycling of synaptic vesicles after stimulation. We study the involvement of HIP1 and the contribution of its interaction with clathrin in endocytosis. We show that the central fragment of HIP1 (HIP1(218–604)) is mislocalized and creates big cellular structures that contain clathrin, AP2, and EEA1. These structures are comprised from smaller units that attach and detach from each other dynamically. In addition, we found some HIP1(218–604) in smaller clusters close to the plasma membrane. To learn about the involvement of HIP1 in early steps of CME, we followed the dynamics of fluorescently tagged clathrin and/or HIP1 or HIP1(218–604), using total internal reflection fluorescent microscopy (TIRFM). Our preliminary results demonstrate that the full length HIP1 particles stay close to the plasma membrane for longer times than clathrin and are less mobile. However, HIP1(218–604) clusters behave in a way that resembles clathrin clusters, implying it lost its functionality in this step. Simultaneous expression of both clathrin and HIP1(218–604) shows mostly overlapping patterns both in the plasma membrane and in inner parts of the cell. In addition, we examined HIP1's effect on synaptic vesicles recycling using styryl dyes uptake assays in hippocampal neurons. We show that overexpression of HIP1 enhances styryl dyes uptake to the synapse, while vesicles pools remain unchanged.

# Abstract: NAP: NEUROPROTECTION BY REDUCTION OF TAU HYPERPHOSPHORYLATION

**Authors:** I. Gozes, I. Divinski, I. Vulih-Shultzman, K. Vered, N. Shiryaev, Y. Matsuoka, P. S. Aisen

**Affiliations:** ^1^Department of Human Molecular Genetics and Biochemistry,* Sackler School of Medicine, Tel Aviv University, Israel; ^2^Georgetown University Medical Center, Washington, DC, USA; *Name pending approval

NAP (NAPVSIPQ) is a short neuroprotective peptide derived from activity-dependent neuroprotective protein (ADNP), a protein essential for brain formation (Bassan et al [1]; Gozes et al [2]; Pinhasov et al [3]). Original studies have shown that ADNP (Furman et al [4]) decorate microtubules. Further studies showed that NAP stimulates microtubule assembly and reduces tau hyperphosphorylation in astrocytes in culture (Gozes & Divinski [5]; Divinski et al [6]. Divinski et al [7]). It is hypothesized that NAP acts, in large part, through a reduction in tau phosphorylation with resultant effects on microtubule stabilization. This is supported by a number of studies. In vivo studies showed that in ADNP-deficient mice there is tau hyperphosphorylation that can be decreased by chronic NAP treatment. In models of neurodegenerative diseases such as experimental encephalomyelitis, NAP treatment reduced tau hyperphosphorylation in the brain. In a model of tauopathy (triple transgenic mice expressing an Alzheimer's disease like phenotype), NAP reduced tau hyperphosphorylation. Part of the NAP effect may be also associated with altered regulation of the kinases that control tau hyperphosphorylation. The functional outcome of the increased hyperphosphorylation of tau is diminished cognitive functions in the case of ADNP deficiency, which in parallel studies was ameliorated by NAP treatment. In the case of EAE, the functional outcome is paralysis, which was ameliorated in part by NAP treatment. Based on these preclinical findings, Allon Therapeutics Inc, a Vancouver-based company, is conducting phase II clinical trials on NAP as a potential neuroprotective drug candidate.

*Supported by BSF, ISF, Gildor Chair, Elton Lab, NIA, NICHD Intramural, Allon Therapeutics Inc.*

## References

[1] Bassan M, Zamostiano R, Davidson A (1999). Complete sequence of a novel protein containing a femtomolar-activity-dependent neuroprotective peptide. *Journal of Neurochemistry*.

[2] Gozes I, Morimoto BH, Tiong J (2005). NAP: research and development of a peptide derived from activity-dependent neuroprotective protein (ADNP). *CNS Drug Reviews*.

[3] Pinhasov A, Mandel S, Torchinsky A (2003). Activity-dependent neuroprotective protein: a novel gene essential for brain formation. *Developmental Brain Research*.

[4] Furman S, Steingart RA, Mandel S, Hauser JM, Brenneman DE, Gozes I (2004). Subcellular localization and secretion of activity-dependent neuroprotective protein in astrocytes. *Neuron Glia Biology*.

[5] Gozes I, Divinski I (2004). The femtomolar-acting NAP interacts with microtubules: novel aspects of astrocyte protection. *Journal of Alzheimer's Disease*.

[6] Divinski I, Mittelman L, Gozes I (2004). A femtomolar acting octapeptide interacts with tubulin and protects astrocytes against zinc intoxication. *Journal of Biological Chemistry*.

[7] Divinski I, Holtser-Cochav M, Vulih-Schultzman I, Steingart RA, Gozes I (2006). Peptide neuroprotection through specific interaction with brain tubulin. *Journal of Neurochemistry*.

# Abstract: CORRELATED SINGLE-UNIT ACTIVITY BETWEEN DIFFERENT EPOCHS OF SINGLE BEHAVIORAL TRIALS IS OBSERVED IN PRIMATE MOTOR CORTEX

**Authors:** Y. Grinvald, D. Inbar, N. Zach, E. Vaadia

**Affiliations:** ^1^Physiology Department, Hadassah Medical School, The Hebrew University, Jerusalem 91120, Israel; ^2^Interdisciplinary Center for Neural Computation (ICNC), The Hebrew University, Jerusalem 91010, Israel

Common analysis of neuronal data involves averaging of activity across many trials or events. Hence, these techniques fail to consider trial-by-trial variability. The objective of this study is to examine dynamic features of neural activity as it evolves within a single trial, while taking into consideration the variability between trials. Thus, we asked the following questions on a single trial basis: are the activities in early and late epochs of a trial correlated? How does the activity in different epochs of a trial correlate with behavioral parameters? To address these questions we recorded, simultaneously, single unit activity, using 32 independently moveable microelectrodes from primary motor (M1) and premotor areas of the monkey during performance of behavioral tasks. The basic task consists of a “center-out” reaching movement performed in a horizontal plane. The analysis reveals signs for correlated activity in different epochs of the trial. For example, activity before the onset of a directional cue (pre-cue) could be correlated with later epochs, after the cue is presented (post-cue). We also found that the degree of correlation changes at different time windows. Furthermore, preliminary analyses suggest that firing rate in the pre-cue interval may be correlated with behavioral parameters (eg, reaction time (RT)). These correlatesmay improve the prediction of behavior on the basis of neural activity in a single trial as well as explain the behavioral variability.

*Supported by BMBF-DIP, BSF, ISF.*

# Abstract: CONSTRAINING KINETICS OF VOLTAGE-GATED CHANNELS USING A GENETIC ALGORITHM

**Authors:** M. Gurkiewicz, A. Korngreen

**Affiliations:** ^1^Mina and Everard Goodman Faculty of Life Sciences, Bar-Ilan University, Israel; ^2^Leslie and Susan Gonda Interdisciplinary Brain Research Center, Bar-Ilan University, Israel

Trans-membrane protein mechanisms such as ion-channels and their activity are at the essence of neuronal transmission. The most accurate method, so far, for determining ionchannel kinetic mechanisms is single-channel recording and analysis. Nevertheless, single-channel recordings carry several holdups and complexities, especially when dealing with voltage-gated channels. Here we show that genetic search algorithms (GAs) can be used to fit whole-cell voltage-clamp data to kineticmodels with a high degree of accuracy. The approach takes into consideration the full range of stimulation protocols used when analyzing voltage-gated ion channels. Unlike most previous analyses done, protocols' results were not analyzed individually, but rather as an entire set of traces from all protocols for a simultaneous analysis. The algorithm was initially tested over simulated current traces produced using several simpleHodgkin-Huxley-like models of voltagegated potassium and sodium channels. Currents were also produced simulating levels of noise expected from actual patch recordings. Finally, the algorithm was used for finding the kinetic parameters of several voltage-gated sodium and potassium channels models via matching its results to data recorded, in nucleated configuration, from layer 5 pyramidal neurons of the rat cortex. Theminimization scheme provides a tool for electrophysiologists in mimicking and simulating voltage-gated ion-channel kinetics on the cellular level.

# Abstract: 3D MAP OF WHISKER REPRESENTATIONS IN TWO VPM COMPARTMENTS

**Authors:** S. Haidarliu, C. Yu, N. Rubin, E. Ahissar

**Affiliations:** Department of Neurobiology, Weizmann Institute of Science, Rehovot 76100, Israel

The dorsolateral part of the ventral posteromedial thalamic nucleus (VPM) is occupied by vibrissal representations known as barreloids. In coronal sections stained for cytochrome oxidase (CO), VPM appears as a homologous structure. However, double-labeling experiments revealed that barreloids are divided into dorsomedial (dm, core) and ventrolateral (vl, tail) compartments (Pierret et al [1]). Recently, we have shown that (1) the extent of VPMdmand VPMvl can be determined by cutting the brain at specific angles, and staining for CO (Haidarliu and Ahissar [2]); (2) neuronal populations of the two compartments of the VPM convey different electrophysiological information during whisking and object touch (Yu et al [3]). Because of such functional segregation, these two compartments can be considered as different sub-nuclei, and their stereotaxic identification in standard sectioning planes becomes important. Here, we apply a method for coordinate transformation based on morphological normalization to convert oblique to coronal coordinates. Using this method we determined the border between VPMdm and VPMvl in serial coronal sections, and created a compartmentalized tree-dimensional model of the VPM. Most of the rostral part of the VPM (∼40%) belongs to VPMdm and contains the rows E and D. In more caudal sections, VPMvl becomes gradually more dominant and occupies between 20% to 60% of the medio-lateral extent of the VPM. The most caudal coronal slice of the VPM belongs to VPMvl containing rows A and B. We suggest that the coronal coordinates of the VPMdm/VPMvl border will be added to standard atlas descriptions of the rat thalamus.

*Supported by The United States-Israel Binational Science Foundation, and the BMBF-MOST Foundation.*

## References

[1] Pierret T, Lavallee P, Deschenes MJ (2000). *Neuroscience*.

[2] Haidarliu S, Ahissar EJ (2000). Size gradients of barreloids in the rat thalamus. *The Journal of Comparative Neurology*.

[3] Yu C, Derdikman D, Haidarliu S, Ahissar E (2006). Parallel thalamic pathways for whisking and touch signals in the rat. *PLoS Biology*.

# Abstract: STATIC AND DYNAMIC FRET STUDIES OF THE CARDIAC IKS POTASSIUM CHANNEL COMPLEX

**Authors:** Y. Haitin, B. Attali

**Affiliations:** Department of Physiology and Pharmacology, Sackler Faculty of Medicine, Tel Aviv University, Tel Aviv 69978, Israel

KCNQ1 channels (Kv7.1) belong to a subfamily of voltagegated K+ channels. KCNQ1 alpha subunits coassemble with calmodulin and KCNE1 beta subunits to generate the Iks potassium current, which is critical for normal repolarization of the cardiac action potential. Mutations in either KCNQ1 or KCNE1 genes produce the long QT syndrome, a life-threatening ventricular arrhythmia. To study both static proximity and voltage-dependent molecular rearrangements of the Iks channel complex, KCNQ1, KCNE1, and calmodulin were C-terminally fused with enhanced cyan fluorescent protein and/or enhanced yellow fluorescent protein and expressed in Xenopus oocytes. Using the spectral analysis of a Zeiss 510 Meta confocal microscope, we studied the fluorescence resonance energy transfer (FRET) simultaneously combined with two-electrode voltage-clamp recording of K+ currents. At –80 mV, a holding potential where no K+ currents flow through the closed Iks channel, a strong constitutive FRET signal was observed suggesting that a distance of less than 10 nM exists between the fluorescently tagged Ctermini of KCNQ1 and KCNE1 in the closed channel conformation. A marked voltage-dependent, FRET signal change was recorded at +30 mV concomitantly with Iks K+ currents, suggesting spatial rearrangement of the KCNQ1 and KCNE1 subunits during the gating process. No FRET signal was observed when 1 : 1 molar ratio of C-terminally tagged KCNQ1-CFP and KCNQ1-YFP was coexpressed despite the simultaneous record of K+ currents, suggesting at least 10 nM spacing in the C-termini of KCNQ1 alpha subunits (adjacent or diagonally facing) or an inappropriate angle of fluorophore polarization for energy transfer. We currently analyze the spatial organization of the Iks complex during the gating process and examine the impact of LQT mutants on these voltage-dependent molecular rearrangements.

*Supported by ISF (672/05) and the Keren Wolfson Family Funds to the second author.*

# Abstract: THE MID-HINDBRAIN ORGANIZER CONTROLS THE DEVELOPMENT OF NORADRENERGIC NEURONS

**Authors:** V. Hakim, K. Liser, H. Tilleman, O. Scheffner, C. Brodski

**Affiliations:** Zlotowski Center for Neuroscience, Faculty of Health Sciences, Ben Gurion University of the Negev, Israel

Dopaminergic neurons originate from the midbrain, close to the mid-hindbrain border, whereas noradrenergic (NA) and serotonergic neurons develop juxtaposed in the hindbrain. The junction between the developing mid and hindbrain harbors cells secreting signals that direct the development of the brain stem, and is hence termed the mid-hindbrain organizer (MHO). The transcription factor Otx2 is expressed in the midbrain and plays a central role in positioning the MHO, defining midbrain patterning and cell fates. In an earlier study, we demonstrated that the position of the MHO determines the location and size of dopaminergic and serotonergic cell populations. In contrast, the development of adjacent cranial nerve nuclei is not influenced by the position of the MHO. Here, we use mouse mutants with a caudally shiftedMHO(En1+/Otx2) to investigate the influence of this organizer on the development of the locus coeruleus, the major NA nucleus. We report that the locus coeruleus develops close to the MHO and is reduced in En1+/Otx2 mutants. Surprisingly, we found that there is a substantial overlap between the ectopically expressed Otx2 and NA marker. This was unexpected since Otx2 was shown in other experiments to be sufficient to induce midbrain cell fate identity. Based on loss of function experiments, Fgf8 is known to be required for the development of NA neurons. However, the interpretation of these results is hampered by the fact that cell survival in the entire rostral hindbrain depends on Fgf8. Therefore, we tested whether the decreased NA cell population in En1+/Otx2 mutants is associated with an altered Fgf8 expression. Interestingly, we found that the Fgf8 domain is in fact enlarged; suggesting that the decrease in the NA cell population is not likely to be mediated through this secreted molecule. Currently we are investigating the possibility that the MHO affects NA neurons via Bmps.

*Supported by the National Institute for Psychobiology Grant no 7-2004-5 and ISF Grant no 864/05 to the fourth author.*

# Abstract: CATEGORY LEARNING FROM PAIRWISE RELATIONS: A DEVELOPMENTAL PERSPECTIVE

**Authors:** R. Hammer, G. Diesendruck, D. Weinshall, S. Hochstein

**Affiliations:** ^1^Interdisciplinary Center for Neural Computation, The Hebrew University, Jerusalem 91010, Israel; ^2^Neurobiology Department, Institute of Life Sciences, The Hebrew University, Jerusalem 91010, Israel; ^3^School of Computer Sciences and Engineering, Hebrew University, Jerusalem 91010, Israel; ^4^Gonda Brain Research Center and Department of Psychology, Bar-Ilan University, Ramat Gan, Israel

Information for category learning can be provided as positive or negative equivalence constraints (PECs/NECs)—an indication that some exemplars belong to the same category or to different categories. In our previous study (Hammer et al [1]), human participants learned new categories from either PECs or NECs. It was found that most participants learned new categories quite well when provided with only PECs while many failed in properly using NECs, even when the provided constraints contained all the information needed for performing the experimental task correctly. This difference in the use of PECs versus NECs may be explained by the ecological observation, that NECs are rarely informative in everyday life while all PECs are informative. This disadvantage of NECs may explain the unavailability of inherent, or early-learned, strategy for properly using even informative NECs. In order to further investigate this hypothesis, we compared the performance of adults and young children when using PECs and NECs. If strategies for using PECs are indeed inherent or acquired early in life, while strategies for using NECs are only sometimes acquired at a latter stage of development, then we expect to see that young children will be able to successfully use PECs as often as adults, but will only rarely use NECs as well as adults.

*Supported by Israel Science Foundation “Center of Excellence;” US-Israel Binational Science Foundation, and EU DIRAC Integrated Project IST-027787.*

## References

[1] Hammer

# Abstract: THE POSTERIOR PARIETAL LOBES AND RECOGNITION MEMORY

**Authors:** S. Haramati, N. Soroker, Y. Dudai, D. A. Levy

**Affiliations:** ^1^Department of Neurobiology, Weizmann Institute of Science, Tel-Aviv University, Rehovot 76100, Israel; ^2^Loewenstein Rehabilitation Hospital, Israel; ^3^Sackler Faculty of Medicine, Tel-Aviv University, Tel Aviv 69978, Israel; ^4^Gonda (Goldschmied)Multidisciplinary Brain Research Center, Bar-Ilan University, Israel

Unlike some other brain regions, notably medial, temporal, and prefrontal cortices, the parietal lobes have not usually been assigned a specific role in long-term declarative memory. However, several recent fMRI studies have reported robust bilateral (left > right) activation in lateral posterior parietal cortex and the precuneus during the retrieval stages of recognition memory tasks. It has not yet been determined what cognitive processes are represented by these activations. In order to examine whether parietal lobe-based processes are necessary for basic episodic recognition abilities, we tested a group of first-incident CVA patients whose cortical damage included (but was not limited to) unilateral (RH or LH) posterior parietal lesions. These patients performed a series of tasks, similar to those yielding parietal activations in fMRI studies: yes/no recognition judgments on visual words, and on colored object pictures and identifiable environMental sounds. The extant of each patient's damage was measured, and percentage of lesion in parietal (and other) structures was determined using a lesion analysis tool (ABLe). Then, correlations were performed between lesion size in ROIs and performance. In addition, voxel-based lesion symptommapping (VLSM) analysis was applied to the whole brain in order to reveal lesion sites that affect behavior. Patients with LH lesions were not impaired at any of the tasks compared to a matched control group. Patients with RH lesions were not impaired in memory for visual words, but were impaired in recognition of object pictures, and had significantly higher false alarm rate in sound recognition. Correlations and VLSM analysis did not reveal significant parietal contribution to behavioral impairments, and imply that these impairments result from perceptual and monitoring problems caused by their damage outside the parietal lobes. This suggests that parietal activations reported in fMRI studies reflect peri-retrieval processes.

# Abstract: HYPOMETHYLATION CAUSED BY EXPOSURE TO ANTIEPILEPTIC DRUGS AS AN EPIGENETIC FACTOR FOR NEURODEVELOPMENT DEFECTS

**Authors:** O. Haramati, T. Levav, H. Golan, D. Galron

**Affiliations:** ^1^Developmental Genetics, Zlotowski Center for Neuroscience, Ben Gurion University, Israel; ^2^Soroka Medical Center, Beer Sheva 84107, Israel

Exposure to several antiepileptic drugs (AEDs) during pregnancy is associated with developmental delay and behavior disorders ranging from reduced learning abilities to autistic features. In newborn rats, prenatal exposure to the AED valproate (VPA) was shown to induce autistic-like features. We have recently reported developmental delay and hypoactivity in adult mice following perinatal exposure to daily application of vigabatrin (GVG) during P4–P14. Compared to control mice behavior in the open field arena, GVG treated mice showed hyperactivity, at P14; a longer distance moved (383 versus 160 cm), and increased velocity (1.29 versus 0.53 cm/s; *P* < .05, *n* = 10). This behavior switched to hypoactivity in GVG treated mice examined at adulthood: distance moved is 1243 versus 1647 cm, velocity is 3.34 versus 4.87 cm/s (*P* < .05, *n* = 10). Repeated, weekly examination abolished the difference in all parameters between GVG and control mice, suggesting that the hyper/hypoactivities are not due to motor deficiency. We suggest that VPA and GVG may modify the developmental program by active DNA demethylation. We used a model system of HEK cells transfected with methylated CMV-GFP plasmid. Plasmid was methylated in vitro by SssI CpG methyltransferase in a buffer containing S-adenosylmethionine (SAM). Complete methylation of the plasmid was confirmed by observing full protection from HpaII digestion. Transfected cells were treated with VPA and GVG for 48 hours. DNA was extracted and plasmid methylation was tested by protection from HpaII digestion and Southern blot analysis. VPA (20 mM) induced partial demethylation of plasmid DNA, whereas GVG (0.05, 0.1 mM) did not induce demethylation of the plasmid. Our results partially support possible involvement of DNA demethylation caused by AEDs as an epigenetic mechanism affecting development. However, different drugs may act through different mechanism.

# Abstract: VISUAL TASK PERFORMANCE AFFECTS THE AUDITORY MISMATCH NEGATIVITY

**Authors:** K. Haroush, S. Hochstein, L. Y. Deouell

**Affiliations:** ^1^Department of Neurobiology, The Hebrew University, Jerusalem 91010, Israel; ^2^Department of Psychology, The Hebrew University, Jerusalem 91010, Israel; ^3^Interdisciplinary Center for Neural Computation, The Hebrew University, Jerusalem, Israel

While attentional resources are invested in a goal-directed manner, remaining resources enable a constant scan of our environ Ment for potentially important deviant events. Detection of deviation may trigger in turn attention reallocation. In addition, this process is reflected in the mismatch negativity (MMN) event-related potential (ERP). A fundamental debate regards the extent to which this signal, generally considered to be “automatic,” is susceptible to attentional load. Previously, we found that the MMN is robust and its magnitude is only slightly decreased despite heavy visual attentional load [1]. However, in that study even the baseline condition was quite demanding, so a floor effect could not be excluded. In the current study, we increased the load difference between the two conditions. In an “attentional blink” paradigm, participants identified two visual targets within a rapid visual stream, while ignoring background test-signal tones of standard pitch, mixed with infrequent pitch deviants. Load was manipulated mainly by changing the contrast level of the visual stream and the contrast ratio between the targets and distractors. Subtracting the ERP of the standard from that of the deviant sounds elicited the MMN signal. Only mismatch responses for stimuli appearing before the visual targets were analyzed. Task performance decreased significantly with increasing load. Whereas the MMN remained robust with both loads, its amplitude was significantly decreased for the high load condition compared to the low load condition and its latency was significantly shorter. Moreover, in the high load condition, MMN peak amplitude and latency was conspicuously smaller in amplitude in correct trials compared to incorrect trials, reflecting the transient attentional state of the participant. These findings suggest that the auditory change detection process is disturbed by perceptual load and attentional factors.

*Supported by ISF “Center of Excellence,” US-Israel BSF (SH); and National Institute for Psychobiology (to the third author).*

## References

[1] (2005). *Reviews in the Neurosciences*.

# Abstract: THE CONTRIBUTION OF THE HEAD GANGLIA TO VENOM-INDUCED HYPOKINESIA IN AN INSECT PREY

**Authors:** M. Haskel, L. Rosenberg, F. Libersat

**Affiliations:** Department of Life Sciences and Zlotowski Center of Neuroscience, Ben Gurion University, Beer Sheva 84105, Israel

The wasp Ampulex compressa uses a direct injection of venom into the head ganglia of its prey the cockroach to induce hypokinesia that lasts for several days. This hypokinesia is characterized by a decrease in spontaneous walking and by the inability to escape. We hypothesize that the venom injection reduces or abolishes the activity of neurons in the head ganglia and that these neurons regulate the excitability of thoracic motor circuits. To test this hypothesis, we performed the following two experiments. First, we performed extra cellular recordings from one neck connective, which contains descending axons fromneurons in the head ganglia. The descending activity in stung animals was decreased by 64% compared to that of control. Such a decrease could indicate a reduction in excitatory drive from the head ganglia to the thoracic motor circuitries. If this is correct, then, artificially decreasing the activity from head ganglia should induce motor impairments similar to those observed in stung animals. To test this, we used procaine, a reversible blocker of voltage-dependant Na+ channel. First, using extra cellular recording we verified that 100 nL of 50% procaine injected into the cockroach CNS is sufficient to block all neuronal activity for at least one hour. Using behavioral tests, we found that during the effective period of procaine, cockroaches injected into the brain spontaneously walked significantly more than controls. In contrast cockroaches injected into the SEG did not spontaneously walk at all and were unable to escape. Then, the later behaved like stung cockroaches. All cockroaches recovered 2 hours after the injection and their behavior did not differ from that of controls. These results indicate that the injection of procaine only in the SEG mimics the effect of venom injection by the wasp. Thus, this also suggests that it is the SEG, rather than the brain, which has a more significant role in venom induced hypokinesia.

*Supported by BSF Grant 2001044.*

# Abstract: A SOLUTION TO THE STEREO CORRESPONDENCE PROBLEM BASED ON CONSTRAINTS IMPOSED BY PHYSICAL SURFACES AND THE DISPARITY GRADIENT LIMIT

**Authors:** T. Hendel, M. Gur

**Affiliations:** Department of Biomedical Engineering, Technion–Institute of Technology, Haifa 32000, Israel

The first stage in the attainMent of stereoscopic depth is the establishment of correspondence between matching features in the two retinal images. The brain seems to solve this correspondence problem easily, best demonstrated by the vivid sense of stereoscopic depth that we get from randomdot stereograms, but efforts to understand how the brain achieves this feat have shown the problem to be surprisingly difficult. We analyze the binocular correspondence problem assuming that the goal of stereoscopic vision is not depth perse, but, rather, the construction of rigid continuous surfaces. Using this assumption we assign to every point in every retinal projection a small neighborhood in its vicinity. If the neighborhood is small enough, it can serve as an approximation to the projection of a tangent plane to the 3D surface that stereopsis tries to construct. Since the two eyes view the same surface, tangent planes in corresponding points should be similar. Therefore every point can use its neighborhood as a signature and try to find a similar signature in the other retinal projection, presumably belonging to the sought for corresponding point. The hard part is deciding what qualifies as “similar.” We do this through the analysis of constraints imposed by 3D surfaces and the disparity gradient limit on binocular fusion. This allows the algorithm to go beyond simple cross-correlation schemes that have been used before. The idea was implemented and tested on randomdot stereograms and natural images (preprocessed with V1- like orientation-selective filters) with good results. We argue that our approach can explain the puzzling existence of two noncongruent ideas on the limit of binocular fusion, namely, Panum's fusional areas and the disparity gradient limit for fusion, and also offers a synthesis of the two. Lastly, we suggest that the motion correspondence problem, in optic flow or in constructing structure from motion, could be solved along similar lines.

# Abstract: ZINC ACTS VIA A SPECIFIC ZINC-SENSING RECEPTOR, IN THE CA3 HIPPOCAMPAL REGION

**Authors:** M. Hershfinkel, L. Besser, E. Chorin

**Affiliations:** Department of Morphology and Zlotowski Center for Neusoscience, Ben Gurion University of the Negev, Beer Sheva 84105, Israel

Zinc plays an important structural and catalytic role in living cells, that is particularly versatile and dynamic in the brain. During embryogenesis zinc is essential for proper brain growth and development. Indeed zinc deficiency postnatally, among other symptoms, leads to the onset of seizures. During neuronal activity there is a remarkable rise in free zinc released from glutamatergic nerve terminals. These steep changes in brain zinc are suggested to play a key physiological role in synaptic transmission, and are also a leading factor in neuronal death following ischemia and epilepsy. We have identified an extracellular zinc sensing receptor, ZnR, in the brain. This is a G-protein-coupled receptor that mediates intracellular calcium release in the brain via the IP3 pathway. Our results suggest that the ZnR is found on the pyramidal post-synaptic neurons in the CA3-hippocampal region. ZnR activity was induced by synaptically released zinc and was attenuated in the presence of the extracellular zinc chelator, CaEDTA. Furthermore, knockout mice lacking synaptic zinc (ZnT-3 KO) show a significant reduction in the ZnR activity following synaptic release. Finally, activity of the ZnR regulates the K+/Cl− cotransporter (KCC), and thereby may modulate the cellular Cl− gradients. We therefore suggest that the ZnR is the link between synaptically released zinc and the zinc-dependent effects on cell signaling and ion transport in post synaptic cells.

# Abstract: LEARNING BYWATCHING: DOES EXPERTISE MAKE A DIFFERENCE?

**Authors:** U. A. Hertz, N. S. Zagury, K. Sella, O. Grinharsh, R. Zur, O. Donchin

**Affiliations:** Ben Gurion University of the Negev, Beer Sheva 84105, Israel

An unusual, although not unique, characteristic of human beings is their ability to learn sophisticated motor tasks through observation of others performing the same task. This ability has been documented repeatedly in the recent literature, and imaging studies are now unraveling the complex network we use when we watch others performing motor tasks. One hypothesis that has been proposed on the basis of this growing literature is that action observation activates our motor system in much the same way that actual practice does. This would suggest that learning from observation is, at root, a very similar process to learning through practice. We sought to test this hypothesis by examining the how action observation interacts with expertise in the task being observed. Human subjects practiced one of two motor tasks— reduced juggling or devil stick dribbling—for thirty minutes each day over the course of three days. On each day, their practice was interrupted twice with an observation session. Each observation session lasted ten minutes. In the observation session, subjects either watched the task they were practicing (“same observation”) or the other task (“different observation”). We find a significant interaction between the day of observation and the difference in performance between the groups. Post hoc testing showed that, when compared to the different observation group, the same observation group performed better on the second and third day but not on the first. We interpret this finding to mean that a certain familiarity with the task is necessary in order to improve from action observation. We made a further effort to understand these results by comparing improvement from one training set to the next when the two sets were separated by an observation period and when they were not separated by an observation set. However, in this analysis we did not find any significant differences between the rate of improvement in the two sets.

# Abstract: EXPOSING RATS TO JUVENILE STRESS AFFECTS BODY WEIGHT AND OPEN FIELD INDICES BOTH SOON AFTER THE EXPOSURE AND IN ADULTHOOD, BUT IN OPPOSITE DIRECTIONS

**Authors:** O. Horovitz, S. Jacobson-Pick, M. M. Tsoory, G. Richter-Levin

**Affiliations:** Department of Psychology and The Brain and Behavior Research Center, University of Haifa, Haifa 31905, Israel

*Background*. Epidemiological studies indicate that childhood trauma is predominantly associated with higher rates of both mood and anxiety disorders, which were associated with altered limbic system functioning. Exposing rats to stress during juvenility (27–29 days of age) has comparable effects and was suggested as a model of induced predisposition for these disorders (Avital and Richter-Levin, 2005; Tsoory and Richter-Levin, 2005; Tsoory et al, 2006). *Objectives*. The current study utilized the juvenile stress model and examined its effects on body weight and exploratory behaviors in the open field test both soon after the exposure and in adulthood. *Results*. Juvenile stressed rats exhibited less body weight gain than control (unexposed) rats soon after the exposure (34 days of age). However, these differences were not evident in adulthood (62 days of age). In comparison with controls, juvenile stressed rats explored the open field more when tested 1hr following the juvenile stress protocol, however, this was not evident at the age of 34 days. Moreover, when tested in adulthood juvenile stressed rats exhibited less exploratory behavior than controls. A similar pattern of results was found also for the anxiety index of time spent in the center of the open field. Compared with controls, juvenile stressed rats spent more time in the center of the open field when tested 1hr after the juvenile stress protocol, however, this was not evident at the age of 34 days. Moreover, when tested in adulthood juvenile stressed rats spent less time in the center of the open field than controls.*Conclusions*. Taken together with previous findings the data suggests that exposure to juvenile stress interacts with maturation processes and may imply alterations in the development of the limbic system, which may underlie the predisposition to exhibitmood and/or anxiety disorders symptoms in adulthood.

# Abstract: BAYESIAN-LIKE INFERENCE UNDERLIES CONTRACTION BIAS

**Authors:** P. Hosseini, Y. Loewenstein

**Affiliations:** ^1^Brain and Cognitive Sciences Department, Massachusetts Institute of Technology, Cambridge, MA 02139-4307, USA; ^2^Howard Hughes Medical Institute, Chery Chase, MD 20815-6789, USA

When subjects judge the magnitude of analog variables, they tend to overestimate large magnitudes and underestimate small magnitudes, an illusion known as contraction bias. We show that an ideal observer that optimally combines noisy memory with prior information will exhibit contraction bias, suggesting that this illusion results from Bayesian inference. This hypothesis predicts that the larger the uncertainty in the memory is the more pronounced the contraction bias will be. In order to test this hypothesis experimentally we constructed a visual memory discrimination task in which subjects were instructed to memorize for several seconds the length of a bar presented on a computer screen, and report its length by comparing it to the length of a reference bar. Analyzing the performance of subjects in this task we show that subjects tend to overestimate large magnitudes and underestimate small magnitudes, in agreement with contraction bias. In order to control the uncertainty in the length of the memorized bar, we added a distracting task to some of the trials. As expected from our hypothesis, the contraction bias in the trials with the distractor was significantly larger than that in the control trials, in agreement with Bayesian decision making. We further show that the prior information used in the Bayesian inference is approximated from a very small number of observations. These results indicate that humans utilize Bayesian inference in order to optimize their performance in working memory discrimination tasks.

# Abstract: MODULATION OF DJ-1 EXPRESSION IN EAE: IMPLICATIONS FOR OXIDATIVE STRESS IN MULTIPLE SCLEROSIS

**Authors:** D. Ickowicz, N. Lev, Y. Barhum, N. Blondheim, E. Melamed, D. Offen

**Affiliations:** *These authors contributed equally to the paper. Laboratory of Neuroscience, FMRC, Rabin Medical Center, Tel Aviv University, Petah Tiqva 49100, Israel

*Background*. Mutations in DJ-1 were discovered recently to cause early onset Parkinson's disease. Accumulating data indicate that DJ-1 plays an important role in oxidative stress, and is involved in various neurodegenerative diseases. Oxidative stress has a central role in the pathogenesis of multiple sclerosis (MS). *Aim*. To examine whether there are changes in DJ-1 expression in an animal model of MS, experimental autoimmune encephalomyelitis (EAE). *Methods*. Chronic EAE was induced in 30 mice that were sacrificed at different disease severities. U-87 human glioma cells exposed to oxidative stress induced by SIN-I were used as an in vitro model. DJ-1 mRNA and protein levels were quantified by real-time PCR and Western blotting, respectively. Changes in DJ-1 isoelectric point were evaluated by isoelectric focusing. MTT and DCF assays were used to quantify viability and intracellular reactive oxygen species (ROS), respectively. Results. We found upregulation of DJ-1 mRNA and protein expression levels in EAE and a correlation between disease severity and increased DJ-1 levels. While DJ-1 isoforms were more alkaline in controls, in EAE there was a shift towards acidic isoforms. ROS induced by SIN-I exposure led to an increase in DJ-1 mRNA and protein levels in human glioma cells. *Conclusions*. This is the first report of modulation of DJ-1 expression in EAE. Upregulation of DJ-1 was noted in EAE, and similar results were observed in glioma cells exposed to ROS. Monitoring DJ-1 levels and oxidized isoforms may serve as a biomarker for evaluation of disease activity and therapeutic response. In view of the accumulating evidence on the central role of oxidative stress in MS, and the importance of DJ-1 in oxidative stress management by the CNS, we believe that DJ-1 will be found to have a central role in MS.

# Abstract: DOWNREGULATION OF T AND B CELL FUNCTION BY THE ONCOFETAL GLYCOPROTEIN ALPHA-FETOPROTEIN

**Authors:** M. Irony-Tur Sinai, N. Grigoriadis, C. Sicsic, O. Abramsky, T. Brenner

**Affiliations:** ^1^The Agnes Ginges Center for Human Neurogenetics, Hadassah Hebrew University Medical Center, Jerusalem 91120, Israel; ^2^Aristotle University, Thessaloniki, Greece

Alpha-fetoprotein (AFP) is a 65 kd oncofetal glycoprotein found in fetal and maternal fluids during pregnancy. Clinical remissions during pregnancy have been observed in several autoimmune diseases, such as multiple sclerosis (MS) and have been attributed to the presence of pregnancyassociated natural immune-reactive substances, including AFP which can exert immunomodulatory effects on immune cells. In this study, we tested the effect of recombinant human AFP (rhAFP) isolated from transgenic goats, which contain the genomic DNA for hAFP, on the autoimmuneinflammatory process in experimental autoimmune encephalomyelitis (EAE), a T cell mediated disease that serves as an animal model for MS. RhAFP treatment markedly improved the clinical manifestations of EAE, preventing central nervous system inflammation and axonal degeneration. RhAFP exerted a broad immunomodulating activity, influencing the various populations of immune cells. Activated T cells derived from treated mice had significantly reduced activity towards the encephalitogenic peptide of myelin oligodendrocyte glycoprotein (MOG), exhibiting less proliferation and reduced Th1 cytokine secretion. Moreover, passive transfer of those T cells into naïve nice resulted in modified clinical disease. In addition, AFP affected the humoral response, causing an inhibition in MOG-specific antibody production. The expression of CD11b, MHC class II and the chemokine receptor CCR5 were also downregulated. The exact mechanism of action is not clear but our results indicate that it affects antigen presentation, signal transduction pathways associated with inflammatory response and apoptosis signaling. In light of our findings, rhAFP may serve as a potential candidate for treatment of MS and other autoimmune diseases.

# Abstract: NEURAL CORRELATES OF OBJECTS INDETERMINACY IN ART COMPOSITIONS

**Authors:** A. Ishai, S. Fairhall

**Affiliations:** Institute of Neuroradiology, University of Zurich, Switzerland

Indeterminate art invokes a perceptual dilemma in which apparently detailed and vivid images resist identification. We used event-related fMRI to study visual perception of representational, indeterminate, and abstract paintings. We hypothesized increased activation along a gradient of posteriorto- anterior ventral visual areas with increased object resolution. Moreover, we postulated that in order to identify ambiguous or indeterminate paintings, subjects would invoke visual mental imagery. Behaviorally, subjects were faster to recognize familiar objects in representational than both indeterminate and abstract paintings. We found activation within a distributed cortical network that includes visual, parietal, limbic, and prefrontal regions. Representational paintings, which depict scenes cluttered with familiar objects, evoked stronger activation than indeterminate and abstract paintings in higher-tier visual areas. Moreover, representational paintings evoked BOLD responses with earlier peaks than indeterminate and abstract paintings. Perception of meaningless scrambled paintings was associated with imagery-related activation in the precuneus and prefrontal cortex. Finally, representational paintings evoked stronger activation than indeterminate paintings in the temporoparietal junction, a region that mediates the binding of object form and spatial location within cluttered visual scenes. Our results suggest that perception of familiar content in art works is mediated by object recognition, memory recall and mental imagery, cognitive processes that evoke activation within a distributed cortical network.

# Abstract: EYEMOVEMENTS DURING SEARCH FOR A SET

**Authors:** M. Jacob, S. Hochstein

**Affiliations:** Interdisciplinary Center for Neural Computation and Department of Neurobiology, Institute of Life Sciences, The Hebrew University, Jerusalem 91010, Israel

Eye movements may reveal patterns of perceptual search and the order of cognitive events. We are using the SET game to learn about higher-order perceptual and cognitive processes, and introduce here a study of eye movements of subjects playing this card game in order to learn about possible explicit and implicit search strategies, with the ultimate goal of inferring underlying mechanisms involved in playing the game. We measured participant eye movements while they played the SET game on a computer screen, after they had previously trained for several sessions with the game and had acquired a good level of rapid Set detection. Eye movements were monitored using an Eyelink eye-movement tracker. We divided fixations into bins representing the 12 cards on the screen and the spaces between them. We measured total time of each fixation, total number of fixations, sampling rate— the frequency of sampling each card belonging to a set, and the “sequential distance” between the 3 cards of the set, comparing fixations of the identified set to those which were not identified. We found that generally the cards of the sets that were ultimately found were among the most observed cards, that is, the total time of fixations was longer for the found (reported) set compared to the unfound sets present at the same display. We raise the important question of what comes first: more intense observation of certain cards leading to finding within them a set, or alternatively, a sense that among certain cards there is a set, leading to observation of these cards, and then consciously perceiving the set.

*Supported by an ISF “Center of Excellence” and the US-Israel Binational Science Foundation.*

# Abstract: ONE-DIMENSIONAL NEURAL CULTURES SUPPORT PROPAGATING ACTIVITYWITH VARIABLE AMPLITUDE AND VELOCITY

**Authors:** S. Jacobi, E. Moses

**Affiliations:** Physics of Complex Systems, Weizmann Institute of Sciences, Rehovot 76100, Israel

We find that neural activity with variable amplitudes can propagate over long distances in one-dimensional cultures of rat hippocampal neurons. The activity is measured by either multielectrode arrays or by calcium fluorescence imaging. Variability of an order of magnitude in both propagation velocity and firing rate amplitudes of spontaneous activity fronts is detected, with a linear relation between velocity and amplitude. Initiation of waves can occur spontaneously or by electrical or chemical stimulation, with different resulting velocities and amplitudes. The validity of current models for signal propagation in neural media, including synfire chain models, is discussed in the light of these new results.

# Abstract: NEURAL CORRELATES OF TREMOR EPOCHS IN A RAT MODEL OF ESSENTIAL TREMOR

**Authors:** G. Jacobson, I. Lev, D. Porrat, D. Cohen, Y. Yarom

**Affiliations:** ^1^Department of Neurobiology, Life Science Institute, The Hebrew University, Jerusalem 91010, Israel; ^2^The Interdisciplinary Center for Neural Computation, The Hebrew University, Jerusalem 91010, Israel; ^3^The Benin School of Engineering and Computer Science, The Hebrew University, Jerusalem 91010, Israel; ^4^The Gonda Multidisiplinary Brain Research Center, Bar-Ilan University, Ramat Gan 52900, Israel

Essential tremor (ET) is the most common movement disorder and one of the most prevalent neurological disorders affecting 0.5%–5% of the population. ET is characterised by episodic muscle tremor at around 10 Hz, amplified during movement. Harmaline-induced tremor is one of the classical animal models for ET, exhibiting similar characteristics and similar drug relief, and is known to involve the inferior olive. The olivary hypothesis proposes that harmalineinduced tremor results from a direct effect of harmaline on T-type calcium currents in olivary neurons, increasing their tendency to oscillate. However, it is yet unclear if and how this neuronalmechanism underlies the episodic nature of essential tremor. To resolve this question, we recorded neuronal activity in the cerebellar cortex of freely moving rats using chronically implanted microelectrode arrays before and after harmaline injection (15mg/kg, Ip). After harmaline application, 6 Hz–10 Hz rhythmicity appeared in the multiunit activity, reflecting olivary input through the climbing fibres. The rats exhibited strong, episodic tremor in the same frequency range, as recorded by EMG electrodes implanted in the limbs. We hypothesized that tremor episodes result from the phase-locking of continuously oscillating olivary units. To study the phase coupling across the different channels, we extracted the amplitude envelopes of the multiunit channels, and studied the frequency band elevated after harmaline application (usually in the 7.5 Hz–15 Hz range). We developed a measure of population phase-locking of the recorded units, and showed that after harmaline application, episodes of phase-locking, lasting up to several seconds, occur much more often than expected assuming independent oscillations. We propose that these phase-locking epochs may underlie the epochs of elevated tremor.

# Abstract: JUVENILE STRESS-INDUCED ALTERATION OF GABAA RECEPTOR ALPHA SUBUNIT MATURATION IN THE RAT

**Authors:** S. Jacobson-Pick, K. Rosenblum, G. Richter-Levin

**Affiliations:** The Brain and Behavior Research Center, University of Haifa, Mount Carmel, Haifa 31905, Israel

Profound evidence indicates that GABAARs are important in the control of the physiological response to stress and anxiety. The GABAARs are best distinguished by their type of alpha subunit. The alpha 2, 3, 5 subunits are predominantly expressed in the brain during the embryonic and early postnatal period, whilst alpha1 are most prominent during later developmental stages. We have shown before that juvenile stress impairs coping behavior in adulthood. The present study examined the short- and long-term effects of juvenile stress on GABAA subunit expression in both the amygdala and hippocampus. At juvenileness (d 26–28), either elevated platform stress or variable stress was applied. The open field and the plus maze were used to assess anxiety level alterations 24 hours or 1 month following the exposure to juvenile stress. Following the behavioral assessment, we quantitatively determined the level of expression of alpha1, 2, and 3 in the hippocampus and amygdala. Abnormal pattern of alpha1, 2, and 3 subunits expression was found mainly in the amygdala one month, but not 24 hours after the exposure to juvenile stress. These results suggest that juvenile stress induces a faulty maturation of the GABAergic system, particularly in the amygdala.

*This work was supported by a NARSAD Independent Investigator Award (2002) to the third author.*

# Abstract: IMPROVED LEARNING ABILITIES AND ANXIOLYTIC BEHAVIOR IN MIDDLE-AGED TAU-TRANSGENIC MICE

**Authors:** Y. Jouroukhin, N. Shiryaev, I. Gozes

**Affiliations:** Human Molecular Genetics and Biochemistry, Sackler Medical School, Tel Aviv University, Tel Aviv 69978, Israel

Tauopathy is a group of disorders characterized by the accumulation of hyperphosphorylated tau protein in the central nervous system (CNS) resulting in dementia. The current research proposed to evaluate tau-related pathology in a mouse model overexpressing the shortest human brain tau isoform in the CNS. Tau-transgenics and wt control group were tested at 7 months of age. This age was previously characterized as exhibiting a peak of molecular neuropathology in these transgenic mice. Here, animal behavioral pathology was evaluated by cognitive, motor, and emotional tests. Our results suggested that these tau-transgenic mice had significantly higher weights as compared to their wt littermates. Comparative analyses of emotional performance, using the open field test (OFT) and the elevated-plus maze (EPM), demonstrated that the tau-transgenic mice exhibited higher explorative behavior and were also less anxious than the controls. The OFT and the EPM also suggested higher motor activity in the tau-transgenic mice. It was previously assumed, however, that these tests could be influenced by the emotional status of the animal. Nonspatial learning abilities were analyzed by the passive avoidance test. This test is known to assess learning and memory of emotional events such as an electric shock. The test results demonstrated successive learning in the tau-transgenic mice, but not in their wt counterparts. In order to estimate whether such reduced anxiety, increased motor functions and learning ability in the tau-transgenic animals are correlated with brain pathology, 8-month-old animals from each group were sacrificed and their brains and spinal cords are going to be analyzed in further biochemical and immunohistochemical analyses. This preliminary data suggests that tau over expression may result in increased cognitive function in 7-month-old mice. Ongoing characterization of potential tau hyperphosphorylation will be important to the interpretation of the data.

*This work is supported by BSF, ISF, Gildor Chair, NIA, NICHD Intramural, and Allon Therapeutics Inc.*

# Abstract: PLASTICITY IN MOTOR NETWORK FOLLOWING ISCHEMIC STROKE

**Authors:** M. Kafri, I. Bova, T. Hendler, O. Aizenstein, N. Bornstein, Y. Assaf

**Affiliations:** ^1^L-E-G Institute for Human Brain Mapping, Tel Aviv Sourasky Medical Center, Tel Aviv University, Tel Aviv 69978, Israel; ^2^Sackler Faculty of Medicine, Tel Aviv University, Tel Aviv 69978, Israel; ^3^Department of Neurology, Tel Aviv Sourasky Medical Center, Tel Aviv University, Tel Aviv 69978, Israel; ^4^Department of Radiology, Tel Aviv Sourasky Medical Center, Tel Aviv University, Tel Aviv 69978, Israel; ^5^Department of Neurobiochemistry, Faculty of Life Sciences, Tel Aviv University, Tel Aviv 69978, Israel

Recovery of motor function after stroke is associated with reorganization in central motor network. Functional imaging has demonstrated recovery-dependent alternations in brain activation patterns when compared to healthy controls. Many factors identified as contributing to this variability. In the current part of the study we aimed to characterize the evolution of brain activation in stroke patients with variable degree of motor status and motor recovery. Nine stroke patients underwent motor 7 function testing and fMRI in the acute/subacute and chronic phases following ischemic stroke. Brain activation was mapped during passive movement of the paretic hand. The patients were divided into three subgroups according to the motor performance in the acute phase and degree of motor recovery. Different patterns of reorganization were identified. In the group of good motor status and good motor recovery, upregulation and spreading of the brain activity was observed in the acute phase followed by normalization of the motor brain activity in the chronic phase. Decreased activity in the motor network in the acute phase was observed in all the patients with poor motor performance. While patients with good recovery demonstrated increase and lateralization of the motor network activity in the chronic phase, patients with poor recovery demonstrated increase and spreading of the brain activation. These findings may suggest that the motor status may reflect the pattern in which the motor network reorganized after stroke.

*This work is supported by Adams Super Center for Brain Studies, Tel Aviv University.*

# Abstract: PC12 CELLS RESPOND TO SIGNALS FROM SERTOLI CELLS

**Authors:** N. Kahn, I. Sekler, T. Kaisman, Z. Silverman

**Affiliations:** Zlotowski Center for Neuroscience, Ben Gurion University of the Negev, Beer Sheva 84105, Israel

Identification of the stem cell-nurturing/regulating “niche” in mature mammals is an increasingly sought-after goal in biology. Among the only defintively identified examples to date are the Sertoli cells, which maintain and regulate the stem cell population of the testis. Because Sertoli cells produce a remarkable variety of secretable proteins, including neurotrophic factors, neurofilaments, and so forth, we hypothesized that these cells might be capable of influencing the development of nontestis precursor cells. To test this hypothesis, we established cocultures of postnatal mouse Sertoli cells and PC12 cells transfected with yellow fluorescent protein. These cells produce catecholamines, and when treated with nerve growth factor, stop dividing, grow neurites, and develop electrical excitability. Sertoli cells were harvested from week old mice, and were seeded together with PC12 cells on coverslips. At various times, the cultures were fixed and processed for immunofluorescence for neuronal markers, including tyrosine hydroxylase (TH), NeuN and MAP-2 and glial markers (eg, clusterin. GFAP and CNPase). Two weeks after exposure to the Sertoli cells, the PC12 cells in the cocultures extended long branched processes and began to resemble neurons, while the PC12 cells cultured alone were morphologically unchanged. Other changes observed included upregulation of clusterin in the PC12 exposed to Sertoli compared to the PC12 cells alone. Our data indicate that Sertoli cells are able to influence undifferentiated, nontesticular cells towards a neural fate. This may suggest that niche cells, represented here by the Sertoli cells, possess a broad potential for progenitor cell interactions.

*Supported by ISF Grant 572/04 to the third author.*

# Abstract: ZINC HOMEOSTASIS IN SUPPORTING CELLS: MOUSE TESTIS CULTUREMODEL

**Authors:** T. Kaisman, N. Kahn, M. Hershfinkel, Z. Silverman

**Affiliations:** Zlotowski Center for Neuroscience, Ben Gurion University of the Negev, Beer Sheva 84105, Israel

Humor aside, the coincidence of proteins common to CNS and testis has been of interest to neurobiologists for some time. These are typically glial proteins such as GFAP, vimentin, clusterin and metallothionein I/II, but also some neuronal ones such as neurofilament protein and neurotrophin receptors. In characterizing another zinc homeostatic protein, ZnT-1, its expression was observed very strongly in oligodendrocytes and the Sertoli cells of the testis. To explore how these two, very different yet surprisingly similar supporting cells manage and utilize zinc, we assessed the influence of zinc and zinc homeostatic proteins in an ex vivo model of testis. Immunofluorescence for phenotypic markers of Sertoli and germ cells indicates that the cultures were highly similar to acute slices taken from postnatal mouse testis. The existence of L-type calcium channels (LTCC), through which zinc and cadmium (Cd2+) as well as calcium permeate into Sertoli cells, was demonstrated by means of fluorescent live cell imaging. After loading the explants with the calcium-sensitive dye, Fura-2AM, depolarizing conditions were created and fluorescence changes monitored over time. Application of the LTCC-blocker, nimodipine, significantly decreased Ca2+, Zn2+, and Cd2+ influx, attesting to the presence of an active LTCC. We then demonstrated that exposure of the explants to Zn2+ and Cd2+ induced upregulation of the glycoprotein, clusterin, which has been associated with apoptosis in both germ cells and neurons, as well as ZnT-1, which was recently shown to regulate the LTCC. Thus, Zn, which normally permeates into both Sertoli and neural cells through the LTCC, upregulates the pro-apoptotic clusterin. Countering this is ZnT-1, also regulated by Zn2+ (and Cd2+), and which itself reduces the entry of Zn2+ and thereby the production of clusterin. These findings provide evidence for common mechanisms involving zinc signaling in neural and nonneural tissue.

*Supported by ISF Grant 572/04 to the fourth author.*

# Abstract: STRESS CONDITIONING TO ODOR AS PART OF CONTEXTUAL REQUIRES LONG-TERM EXPOSURE

**Authors:** S. Kamchi, S. Zhuchenko, G. Richter Levin

**Affiliations:** The Brain and Behavior Research Center, University of Haifa, Mount Carmel, Haifa 31905, Israel

Memory for odors that are associated with intense emotional experiences is often strongly engraved. Odors are claimed to be more closely connected to affect than other sensory experiences. They can serve as potent contextual cues for memory formation and emotional conditioning. Though traumarelated smells have long been noted by clinicians to be precipitants of traumatic memories in patients with posttraumatic stress disorder (PTSD), very few reports have been published that document this. Recently it has been found that an olfactory stimulus can serve effectively as a CS in fear conditioning paradigms and that the basic principles governing the acquisition of emotional responses conditioned to auditory and visual CSs can be extended to the olfactory system. However, it is not clear whether as opposed to serving as apperceptive, cue olfaction can serve effectively as part of the context. In this work our main objective is to establish a methodological approach for associating odor as contextualcue with a traumatic event, based on the assumption that continuous, long lasting but not a brief exposure to the odor (in the presence of another salient cue, from other modality) will be sufficient for odor-contextual conditioning. Five or 6 days of contextual-odor foot-shock association paradigm was sufficient to establish long term contextual conditioning to the odor. The traumatic memory for odor was assessed as freezing behavior measured in a different context with the same odor as part of the new context. However, 1 day contextualodor stress association paradigm was not sufficient to create contextual odor conditioning. The results indicate that contextual odor conditioning is slow to develop. The possibility that contextual odor conditioning is slow to develop but once developed it forms a robust emotional memory will be assessed in future studies.

*This study was supported by an EU project grant (no 512012) to the third author.*

# Abstract: FOREBRAIN m1 MUSCARINIC ACETYLCHOLINE RECEPTORS MODULATE HIPPOCAMPAL DEPRESSION AND FORGETTING OF LABILE SPATIAL MEMORY

**Authors:** A. Kamsler, T. J. McHugh, D. Gerber, S. Y. Huang, S. Tonegawa

**Affiliations:** Picower Institute for Learning and Memory, Massachusetts Institute of Technology, Cambridge, MA, USA

Understanding the neuromodulatory role of acetylcholine in central nervous system networks has been difficult due to the ubiquitous nature of this neurotransmitter and molecular similarity of receptor subtypes. We therefore generated conditional knock out (ko) mice carrying a floxed allele of this gene. These mice have been crossed with mice carrying a cre transgene driven by the Emx1 promoter (m1 fl/fl Cre(+) mice), resulting in postnatal M1 gene deletion only in the excitatory neurons of the forebrain. These mice exhibit normal overall behavior and morphology and do not demonstrate hyperactive behavior in an activity chamber. Stimulation of Schaffer collaterals in hippocampal slices from m1 fl/fl(+) mice resulted in EPSPs that were similar to controls. LTP was normal but paired pulse facilitation was enhanced. A stimulation protocol that produces LTD in young slices had no effect on the controls and induced LTP in m1 fl/fl(+) slices. Perfusion of DHPG produced mGluR-dependent LTD in controls but not in m1 fl/fl(+). In vivo recording of field potentials evoked by perforant path stimulation exhibited decreased paired pulse facilitation in m1 fl/fl(+) mice. M1 fl/fl(+) mice were similar to controls in hippocampusdependent forms of fear conditioning and in the Morris water maze. Changing the position of the platform resulted in a marked tendency of m1 fl/fl(+) mice, but not m1 fl/fl(–) mice, to swim to the previously learned location when the initial location was learned in a rapid manner. These data suggest a role for forebrain m1 muscarinic receptors in modulating the plasticity of the hippocampal network and facilitating the ability of the hippocampus to react rapidly to a changing environment.

# Abstract: THE EFFECT OF TISSUE FIXATION ON AGING RESEARCH

**Authors:** T. Katzir, B. Omanuti, Y. Assaf

**Affiliations:** Department of Neurobiochemistry, Faculty of Life Sciences, Tel Aviv University, Tel Aviv 69978, Israel

Aging is a complicated, multifactorial andmultiregional process affecting cognitive abilities. The effects of aging on the brain are widespread and have multiple etiologies. The main can be summarized in these following points: (1) cognitive changes—memory decline as well as increase in preservative behavior (decreased executive function). (2) Physical changes—a reduction in prefrontal cortical volume and increased subcortical white matter lesions. (3) Biochemical changes—the neurotransmitters most discussed with regard to aging are dopamine and serotonin. Dopamine levels decline by around 10% per decade from early adulthood. Serotonin and brain derived neurotrophic factor levels also fall with increasing age may be implicated in the regulation of synaptic plasticity and neurogenesis in the adult brain. Due to its complexity, rodent models of ageing are valuable in assessing the neurodegenerative processes leading to the cognitive decline. In this work we have performed quantitative T2 MRI examination of young (4 months) versus adult (12–14 months) rats in vivo and in vitro following formalin fixation. Voxel-based morphometry was used to statistically compare between the two groups on a pixel-by-pixel basis. In vitro ANOVA maps of the two groups showed (at P < .05) specific changes in white matter regions (corpus callosum and internal capsule). The T2 in the white matter of the young age group was higher than that of the adult group, opposite to what expected in vivo. Indeed, the in vivo ANOVA maps revealed a completely different pattern of changes. There T2 was increased at the adult group mainly in the thalamus and cingulated cortex. These results suggest that formalin fixation has differential effect on the aging brain as it most probably fixates the adult brain differently than the young ones. In addition, this fixation artifact seems to be much more significant in areas of white matter. Therefore we need to examine carefully fixated brains in aging.

# Abstract: ELECTRICAL STIMULATION OF THE BASOLATERAL AMYGDALA AFTER RETRIEVAL DISRUPTS EXTINCTION BUT NOT RECONSOLIDATION OF DRUG-ASSOCIATED CUES

**Authors:** A. Katzir, A. Zangen

**Affiliations:** Department of Neurobiology, The Weizmann Institute of Science, Rehovot 76100, Israel

Chronic vulnerability to relapse is a difficult challenge for the treatment of drug addiction. This relapse is often precipitated by an environmental stimulus (conditioned stimulus— CS) previously associated with drug-taking (the unconditioned stimulus—US). Numerous studies have implicated the basolateral amygdala (BLA) as essential for establishment of drug-taking behavior and the association between environmental cues and the reinforcer. Following retrieval, two opposite processes can be activated: reconsolidation, which acts on the original memory (CS-US) and extinction which involves the formation of distinct alternative memory (CS— no US). The aim of this study was to disrupt reconsolidation of drug-associated cues in order to reduce cue-induced relapse and to disrupt the extinction of drug-associated cues, in respect to the retrieval period duration, using localized electrical stimulation. We hypothesized that low frequency (1 Hz) electrical stimulation of the BLA after retrieval would interfere with the ongoing neuronal plasticity and consequently interfere with the stabilization of the activated memory trace. Therefore, we assessed the effect of BLA electrical stimulation, following different retrieval durations, on drugseeking behavior, as expressed by lever-presses. We found that BLA electrical stimulation following the longer retrieval period (5 min) induced elevation in responding during the drug-seeking test, indicating disruption of the extinction process. On the other hand, BLA stimulation following a shorter retrieval period (1min) did not affect responding in the drug-seeking test. These results suggest that low frequency electrical stimulation into the BLA can interfere with extinction, but not reconsolidation of the memory trace. Additional investigation is needed to explore the possible applications of electrical stimulation of specific brain regions in addiction therapy.

# Abstract: NEURONAL ARTS DIFFER FROM PERIPHERAL ARTS IN MOLECULAR SIZE AND INTRACELLULAR LOCALIZATION

**Authors:** S. Kemeny, P. Toidman, Y. Loboda, S. Larisch, J. P. Finberg

**Affiliations:** ^1^Pharmacology Department, Rappaport Faculty of Medicine, Technion–Institute of Technology, Haifa 32000, Israel; ^2^Pathology Department, Rambam Hospital, Haifa, Israel

The proapoptotic protein ARTS is a member of the filament-forming septin family, but being derived by alternate splicing from the H5/PNUTL2/hcdcrel2a/2b gene, it differs in its C-terminal structure from other products of this gene, and from other septins. Inmany cell lines derived from peripheral tissues, ARTS is localized to mitochondria, but upon apoptotic stimuli it translocates to the nucleus (Larisch et al [1]). Neuronal ARTS (nARTS) is expressed in most areas of rat brain as seen by immunohistochemistry, but in rat brain tissue, as well as in primary cultures of rat cortical and cerebellar granule neurons, is expressed in a shorter form (25 kDa) than peripheral ARTS (32 kDa). nARTS lacks part of the Nterminal amino acid sequence of peripheral ARTS, as shown by its binding to an antibody specific to the C-terminal but not the N-terminal sequence by immunoblotting. In addition, confocal microscopy showed that in primary rat cortical neurons prepared from E-18 embryos and grown for 7 days IV in neurobasal medium plus B27supplement, nARTS is localized mainly to the nucleus but not to the mitochondria. In SH-SY5Y, however, nARTS localizes to mitochondria. Levels of nARTS were elevated as a result of different insults in vitro as well as in vivo. In primary rat cortical neurons, following withdrawal of B27 from the growth medium there was a strong upregulation of nARTS, especially after 48 hours. In SH-SY5Y cells, nARTS occurs as the 25 kDa variant, but following transfection with full-length ARTS and treatment with the proteasome inhibitor MG132, the full length form is also seen. This data indicates that nARTS might be involved in neuronal death, but it is unclear whether its role is proapoptotic or otherwise, which is currently under investigation.

## References

[1] Larisch S, Yi Y, Lotan R (2000). A novel mitochondrial septin-like protein, ARTS, mediates apoptosis dependent on its P-loop motif. *Nature Cell Biology*.

# Abstract: DYNAMICAL DISTRIBUTED CURRENT SOURCE MODEL FOR ERP-BASED ON ANATOMY AND CONNECTIVITY

**Authors:** A. S. Keren, A. B. Geva, L. Y. Deouell

**Affiliations:** ^1^The Interdisciplinary Center for Neural Computation, The Hebrew University, Jerusalem 91010, Israel; ^2^Department of Psychology, The Hebrew University, Jerusalem 91010, Israel; ^3^Department of Electrical and Computer Engineering, Ben Gurion University of the Negev, Beer Sheva 84105, Israel

Separating event-related potentials (ERP) into basic components and localizing their cerebral sources are major challenges for cognitive neuroscience, as the problem is ill posed. Since there are more sources of electric field than sensors, an infinite number of neural activity configurations can produce the same measured potentials, and therefore constraints must be set in order to limit the possible solutions. The majority of current methods rely on some biologically or physically implausible assumption, such as temporal independence between regions of activity, vast spatial smoothness, or approximation of large volumes with single dipoles. Despite interesting developments in recent years, current methods are yet to show robust and reliable results with real data. In our study we seek to incorporate spatiotemporal constraints derived from anatomy, connectivity, and electrophysiology. We are developing a distributed current source model, with spatial constraints for the locations and orientations derived from structural anatomy (sMRI). In order to apply temporal constraints based on anatomical and physiological data, a dynamical network model is devised incorporating previously studied neural population connectivity, conduction delays, and impulse responses. The model is formulated in discrete time difference equations, whichmake its analysis and implementationmore efficient and straight forward. The dynamics is defined up to parameters, which are to be determined such that the projection of the activity onto the scalp electrodes best fits the measurements. Once the dynamics parameters are determined, the spatiotemporal evoked stream of activation can be reconstructed and simulated. This approach may eventually allow not only inference on sources distribution across time, but functional connectivity as well. We present here the model equations and some simulation results of the dynamics with arbitrary parameters.

*Supported by Grant no I-G-815-8.4/2004 by the German Israeli Foundation (GIF) to the third author.*

# Abstract: THE EFFECTS OF HIGH-FREQUENCY STIMULATION OF THE SUBTHALAMIC NUCLEUS IN THE SIGNAL ATTENUATION RAT MODEL OF OBSESSIVE COMPULSIVE DISORDER

**Authors:** O. Klavir, C. Winter, S. Flash, D. Joel

**Affiliations:** ^1^Department of Psychology, Tel Aviv University, Tel Aviv 69978, Israel; ^2^Department of Psychiatry and Psychotherapy, University Medicine Charité, Berlin, Germany

In the signal attenuation rat model of obsessive compulsive disorder (OCD), compulsive behavior is induced by attenuating a signal indicating that a lever-press response was effective in producing food. Compulsive behavior is evident as excessive lever-presses that are not followed by an attempt to collect food. The most effective treatments known today for OCD are pharmacological treatments that use selective serotonin reuptake inhibitors and a behavioral treatment that uses a technique of exposure and response prevention. In severe refractory cases neurosurgery is sometimes performed. Following the success of high-frequency stimulation (HFS) in replacing lesions in neurologically-related neuropsychiatric disorders including Parkinson's disease, dystonia, and Tourette syndrome, the present study tested this technique by assessing the effects of HFS of the subthalamic nucleus (STN) on compulsive behavior in the model. In order to differentiate between the effects of signal attenuation and extinction per se, sham and STN HFS rats were tested in an extinction test that was or was not preceded by signal attenuation (post-training signal attenuation procedure and regular extinction procedure, resp). STN HFS abolished the “compulsive”-like behavior in rats undergoing signal attenuation. These results might support the acceptance of HFS as a treatment for refractory OCD and might suggest the STN as a possible target area.

# Abstract: TOWARD ANIMAL MODELS OF FOOD DISORDERS: TASTE-DEPENDENT SOCIOPHOBIA IN THE RAT

**Authors:** Y. Klin, M. Guitton, Y. Dudai

**Affiliations:** ^1^Department of Neurobiology, The Weizmann Institute of Science, Rehovot 76100, Israel

The conditioned response (CR) in laboratory paradigms of classical conditioning is selected by the experimenter among the behavioral repertoire emitted in response to the conditioned stimulus (CS). In conditioned taste aversion (CTA), the subject learns to associate a tastant (the CS) with delayed toxicosis (an unconditioned stimulus, US) to yield taste aversion (the CR). Although USs that induce visceral malaise are particularly effective in CTA, the association of taste with delayed negative internal states that could generate CRs that are different from taste aversion should not be neglected. Such acquired associations may contribute to the ontogenesis and reinforcement of some types of eating and digestive disorders. We have recently reported that a delayed anxiety-like state, induced by the anxiogenic drug metachlorophenylpiperazine, can specifically associate with taste to produce CTA in the rat (Guitton and Dudai [1]). Here we report that a similar protocol results in a long-term impairment in social behavior in response to the conditioned taste. This possibly represents a component of the anxiety that generates a social CR in a type of protocol that commonly quantifies only gustatory CRs. The acquired association of food and social behavior may bear relevance to some eating and social pathologies. This is hence a learned situation in which food and company do not mix well.

## References

[1] Guitton MJ, Dudai Y (2004). Anxiety-like state associates with taste to produce conditioned taste aversion. *Biological Psychiatry*.

# Abstract: GENETIC VARIATION IN THE ARGININE VASOPRESSIN 1A RECEPTOR (AVPR1A) PREDICTS ALTRUISM SHOWN BY INDIVIDUAL DIFFERENCES IN DICTATOR GAME AND SOCIAL VALUES ORIENTATIONS MONEY ALLOCATIONS

**Authors:** A. Knafo, S. Israel, R. Bachner-Melman, L. Cohen, E. Lerer, E. Laiba, Y. Raz, L. Nemanov, I. Gritsenko, C. Dina, G. Bornstein, R. P. Ebstein

**Affiliations:** ^1^Psychology Department, The Hebrew University, Jerusalem 91010, Israel; ^2^Human Genetics, The Hebrew University, Jerusalem 91010, Israel; ^3^Brain and Behavioral Sciences, The Hebrew University, Jerusalem 91010, Israel; ^4^S. Herzog Memorial Hospital, Jerusalem, Israel; ^5^Centre National de la Recherche Scientifique, Lille, France

We provide the first evidence that genes partially account for individual differences in altruism as assessed by money donations in experimental games. Games are the foundation of experimental economics and informative regarding economic and social behaviors. This report examines the role of the arginine vasopressin 1a receptor (AVPR1a), associated in lower vertebrates with social communication, and in humans with autism, with game behavior that reveals differences in altruism. To examine whether two AVPR1a promoter-region repeat elements (RS1, RS3) are associated with differences in money allocations in games that measure prosocial behavior and to test whether there is a relationship between repeat length and gene transcription. Participants played an online version of the Dictator Game and the Social Values Orientation task for real money payoffs. Additionally, AVPR1a mRNA was measured in 20 lymphoblast cell lines genotyped for RS1 and RS3 using real time PCR. The sample consisted of University students (60 men, 70 women) whose average age was 22.24 (SD = 3.82). Association using a tdt test was observed for RS3 and sum of money allocated in the Dictator Game (UNPHASED: global *P* value = .026). In particular, RS3 length predicted allocation in the Dictator Game (1- way ANOVA: *F* = 4.959, P = .027). There was also a strong positive correlation between RS1 length and average monetary allocation (*r* = 0.926, *P* = .003). A negative correlation between AVPR1a RS1 length and mRNA levels was significant and very strong (*r* = 0.963, *P* = .007). These findings strengthen the association between AVPR1a and social cognition skills in man and suggest both psychological and molecular biological mechanisms by which length variations in this receptor contribute to both normal and psychopathological behaviors.

*Research was partially supported by the Israel Academy of Sciences and Humanities and by a grant from the Israel National Institute for Psychobiology.*

# Abstract: EXPLORATORY PATTERNS OF AN ACTIVE PROXIMAL SENSING SYSTEM

**Authors:** P. M. Knutsen, A. Biess, A. Yohanan, I. Harel, E. Ahissar

**Affiliations:** ^1^Department of Neurobiology, Weizmann Institute of Science, Rehovot 76100, Israel; ^2^Department of Mathematics, Weizmann Institute of Science, Rehovot 76100, Israel; ^3^Department of Biological Regulation, Weizmann Institute of Science, Rehovot 76100, Israel

The vibrissal system of rodents is an active sensing system, whereby movements of the whiskers enable exceptional proximal sensing capabilities. The sensitivity of the vibrissae system to direction, amplitude, and frequency of whisker stimulation implies that whisker movements should be precisely controlled, despite their stereotyped patterning. We decomposed whisker movements into translational and rotational components, and found that the proximal 70% of the whisker shaft lies flat in a plane and exhibits negligible deformation during whisking in free air. Analysis of this rigid portion of the shaft reveals that whisking involves rhythmic movements in all dimensions. Some of the movement components kept constant phase relationships from bout to bout and some not, and whisking frequencies were modulated at a rate 0–5 Hz/s. The mystacial pad made translations during whisking and usually not parallel to the whisker rows. The pad may assume different vertical set-points, suggesting rats target specific vertical zones for exploration. We found that azimuthal rotation was coupled with torsional rotation of the whisker shaft. Thus, a particular object can be scanned along the circumference of the whisker follicle. These observations add significantly to the complexity and repertoire of vibrissal behavior and of mechanical encoding within the follicle, and provide vital insights on how stimulus selectivity of the trigeminal pathway can be exploited by fine motor control. Altogether, these observations show that, although stereotypical, whisking cannot be reduced to a onedimensional movement.

*Supported by Israel Science, Minerva, and BMBF-MOST Foundations, and the Human Frontiers Science Program.*

# Abstract: THE IMMEDIATE EARLY GENE ARC IS ASSOCIATED WITH BEHAVIORAL RESILIENCE TO STRESS EXPOSURE IN AN ANIMAL MODEL OF POSTTRAUMATIC STRESS DISORDER

**Authors:** N. Kozlovsky, Z. Kaplan, J. Zohar, M. A. Matar, H. Cohen

**Affiliations:** ^1^Anxiety and Stress Research Unit, Mental Health Center, Ben Gurion University of the Negev, Beer Sheva 84105, Israel; ^2^Division of Psychiatry, The Chaim Sheba Medical Center, Tel Aviv University, Tel Aviv 69978, Israel

Changes in neural plasticity and synaptic efficacy in various brain areas appear to play pivotal roles in the pathophysiology of posttraumatic stress disorder (PTSD). Mechanisms which mediate adaptive and maladaptive changes in the brain in response to stress exposure are thus important. Activity-regulated cytoskeletal-associated protein (Arc) is an effector immediate early gene (IEG) which has direct effects on intracellular homeostatic functions. Increased expression of Arc has been associated with increased neuronal activity and with consolidation of long-term memory. It may thus play an important role in mediating experience-induced reorganization and/or development of synaptic connections. This study assessed the long-term expression of mRNA for the Arc gene in selected brain areas in relation to prevalence rates of behavioral response patterns and levels of circulating corticosterone in an animal model of PTSD. The hippocampal CA1 and CA3 subregions of individuals whose behavior was minimally or partially disrupted in response to predator scent stress demonstrated significantly increased levels of Arc mRNA, than did unexposed controls. Rats whose behavior was severely disrupted demonstrated no such upregulation. Consistent with the hypothesis that the Arc gene has a promoting effect on neuronal function and/or structural changes, this study raises the possibility that Arc may be associated with resilience and/or recovery after stress exposure.

# Abstract: LONG-TERM DOWNREGULATION OF BDNF MRNA IN RAT HIPPOCAMPAL CA1 SUBREGION CORRELATES WITH PTSD-LIKE BEHAVIORAL STRESS RESPONSE

**Authors:** N. Kozlovsky, Z. Kaplan, J. Zohar, M. A. Matar, H. Cohen

**Affiliations:** ^1^Anxiety and Stress Research Unit, Mental Health Center, Ben Gurion University of the Negev, Beer Sheva 84105, Israel; ^2^Division of Psychiatry, The Chaim Sheba Medical Center, Tel Aviv University, Tel Aviv 69978, Israel

Brain-derived neurotrophic factor (BDNF) and its intracellular kinase-activating receptor TrkB have been implicated in the neurobiological mechanisms underlying the clinical manifestations of posttraumatic stress disorder (PTSD), especially those related to synaptic efficacy and neural plasticity. BDNF interacts with components of the stress response such as corticosterone, and plays an important role in growth, maintenance, and functioning of several neuronal systems. This study employed an animal model of PTSD to investigate the relationship between prevalence rates of distinct patterns of behavioral responses to predator stress, circulating levels of corticosterone and local levels of mRNA for BDNF, TrkB, neurotrophin-3 (NT3) and nerve growth factor (NGF) in selected brain areas. Animals whose behavior was extremely disrupted by exposure selectively displayed significant downregulation of mRNA for BDNF and upregulation of TrkB mRNA in the CA1 subregion of the hippocampus, compared to animals whose behavior was minimally or partially affected and to unexposed controls. The response was consistent throughout the entire study only in CA1. The consistent long-term BDNF downregulation and TrkB upregulation associated with extreme behavioral compromise may be associated with chronic stress-induced psychopathologic processes, especially in the hippocampus. The corresponding changes in neural plasticity and synaptic functioning may mediate clinical manifestations of PTSD.

# Abstract: REGION-DEPENDENT NMDA RECEPTOR LOSS IN ApoE KNOCKOUT MICE

**Authors:** E. Kravitz, M. Nawrocky, A. Biegon

**Affiliations:** ^1^The Joseph Sagol Neuroscience Center, The Chaim Sheba Medical Center, Tel Aviv University, Tel Aviv 69978, Israel; ^2^Sackler Faculty of Medicine, Tel Aviv University, Tel Aviv 69978, Israel; ^3^Brookhaven National Laboratory, Israel

Alzheimer's disease (AD) is a devastating neurodegenerative disorder which preferentially strikes women. The hall mark of AD is cognitive decline, and the glutamatergic system, specifically the NMDA receptors, plays a major role in cognitive processes. ApoE knockout (APOKO) is an animal model of AD. We tested the hypothesis that NMDA receptor availability will be lower in APOKO mice than in wildtype mice. C57bl mice (wildtype and APOKO, 8–10 months old) were used in the experiments. Animals were decapitated and brains were quickly removed and frozen for cryosectioning in multiple consecutive series. Coronal brain sections at the level of striatum and hippocampus were incubated with the selective noncompetitive NMDA antagonist [3H]MK801. Nonspecific binding was assessed on consecutive sections in the presence of excess unlabeled MK801. Washed and dried sections were scanned by beta imaging and quantitative regional analyses were performed with beta vision software. APOKO females (*N* = 11) had significantly decreased NMDA binding compared to control females (*N* = 7), which was also region-dependent [significant 2 way ANOVA by genotype (*P* = .004) and region (*P* = .001)]. The decrease was especially pronounced in hippocampal regions (eg, CA1, 39.1%; DG, 38.4%; CA3, 37.5%) which are linked to cognitive performance, while there was no apparent difference in the striatum and cingulate cortex. Since APO-related cognitive deficits were described, which were larger in APOKO females than APOKO males (Raber et al, PNAS 1998), we also tested for differences between APOKO females (*N* = 11) and APOKO males (*N* = 10). While there was a trend for lower NMDA binding in the females, it did not reach statistical significance. In conclusion, APOKO animals, which have been shown to exhibit cognitive deficits compared to wildtype, also have decreased NMDA receptor availability in regions that are important in cognition.

*This work is supported by J. Sagol doctoral fellowship.*

# Abstract: BRAIN INTERLEUKIN-1 MEDIATES CHRONIC STRESS-INDUCED DEPRESSION IN MICE VIA ADRENOCORTICAL ACTIVATION AND HIPPOCAMPAL NEUROGENESIS SUPPRESSION

**Authors:** T. Kreisel, I. Goshen, O. Ben-Menachem-Zidon, J. Weidenfeld, T. Ben-Hur, R. Yirmiya

**Affiliations:** ^1^Department of Psychology, The Hebrew University, Jerusalem 91010, Israel; ^2^Department of Neurology, The Agnes Ginges Center for Human Neurogenetics, Hadassah University Hospital, Israel

Several lines of evidence implicate that the pro-inflammatory cytokine interleukin-1 (IL-1) is the etiology and pathophysiology of major depression. To explore the role of IL-1 in chronic stress-induced depression and in some of its underlying biological mechanisms, we used the chronic mild stress (CMS) model of depression. Mice subjected to CMS for 5 weeks exhibited depressive-like symptoms, including decreased sucrose preference, reduced social exploration and adrenocortical activation, concomitantly with increased IL- 1â levels in the hippocampus. In contrast, mice with deletion of the IL-1 receptor type I (IL-1rKO) or mice with transgenic, brain-restricted over-expression of IL-1 receptor antagonist did not display any CMS-induced behavioral or neuroendocrine changes. The blunting of the adrenocortical activation in IL-1rKO mice may play a causal role in their resistance to depression, because removal of endogenous glucocorticoids by adrenalctomy also abolished the depressive-like effects of CMS. Reduced hippocampal neurogenesis, another putative mechanism of depression, may also underlie IL-1's involvement in CMS-induced depression, because whereas in WT mice CMS significantly reduced neurogenesis, measured by both BrdU and doublecortin immunohistochemistry, no such decrease was observed in IL-1rKO mice. Moreover, the effects of CMS on both behavioral depression and neurogenesis could bemimicked by exogenous administration of IL-1â via osmotic minipumps for 4 weeks. These findings indicate that elevation in brain IL-1 levels, which characterizes many medical conditions, is both necessary and sufficient for producing the high incidence of depression found in these conditions. Thus, procedures aiming at reducing brain IL-1 levels may have potent antidepressive actions.

# Abstract: ERUCYLPHOSPHOHOMOCHOLINE-INDUCED CELL DEATH IN HUMAN GLIOMA CELLS: ROLE OF REACTIVE OXYGEN SPECIES

**Authors:** W. Kugler, K. Linnemannstöns, L. Veenman, M. Gavish, M. Lakomek

**Affiliations:** ^1^Universitäts-Kinderklinik, 37075 Göttingen, Germany; ^2^Technion–Israel Institute of Technology, Haifa 31096, Israel

In contrast to standard chemotherapeutics, alkylphosphocholines (APC) are membranophile agents not directly targeting cellular DNA. Erucylphosphohomocholine (ErPC3) is a promising APC member for parenteral administration. It exerts strong anticancer activity and is a potent inducer of apoptosis, even in highly chemoresistant cells. Initial mechanistic studies indicate that mitochondria are central to ErPC3-induced apoptosis. Mitochondria are one of the major sources of reactive oxygen species (ROS). Oxidative stress and the redox state of a cell are supposed to play a pivotal role in regulating apoptosis. The 18 kDa peripheral-type benzodiazepine receptor (PBR) exerts various cell functions and is involved in a functional structure designated as the mitochondrial permeability transition pore. Published data suggest that PBR is a target for ROS which induce the formation of PBR polymers modulating PBR-mediated functions. We have shown previously that the classical PBR ligands PK11195 and Ro5-4864 both prevented apoptosis induction by ErPC3 in human glioma cells. In a first attempt to analyse a possible correlation between ErPC3, PBR, and ROS we investigated the role of ROS in ErPC3-induced cell death, using A172 and U373MG cells and different ROS inhibitors. The antioxidant butylated hydroxyanisole was able to inhibit ErPC3-induced apoptosis and cytotoxicity in both cell lines whereas iron chelators (deferoxamine, phenanthroline) blocked apoptosis in U373MG cells only. Sulfhydryl reagents (dithiotreitol, glutathione), superoxide dismutase, N-acetylcysteine and Trolox had no effect on ErPC3-induced cell death. Catalase partly inhibited ErPC3-mediated apoptosis in A172 cells. These findings are consistent with the hypothesis that hydrogen peroxide induced by ErPC3 acts close to its site of generation in A172 cells, whereas hydroxyl radicals produced in U373MG cells trigger the execution of apoptosis by inducing opening of the mitochondrial permeability pores.

*This work was supported by a grant from the Volkswagen- Stiftung to the first, third, fourth, and fifth authors(Joint Lower Saxony—Israeli Research Projects; VWZN2047).*

# Abstract: VOLTAGE-DEPENDENT RELIEF OF A TONIC BLOCK IMPOSED ON THE RELEASE MACHINERY BY PRESYNAPTIC GROUP II-LIKEMGLURS INITIATES RELEASE AT THE CRAYFISH NEUROMUSCULAR JUNCTION

**Authors:** Y. M. Kupchik, H. Parnas, I. Parnas

**Affiliations:** Department of Neurobiology, The Hebrew University, Jerusalem 91010, Israel

The role of a presynaptic, metabotropic, glutamate autoreceptor in the crayfish neuromuscular junction was studied. Pertussis toxin abolished glutamate-induced feedback inhibition while LY379268, a group II mGluR agonist, induced feedback inhibition. LY341495, a group II mGluR antagonist, relieved a tonic block imposed on release at control (physiological) conditions and increased evoked release 4.22-fold and asynchronous release rate 3.3-fold. LY341495 also decreased the log m/log pulse amplitude (PA) slope from 6.63 to 3.88, and shortened the minimal delay of release from 1.4 millisecond to 1.0 millisecond. A brief and strong depolarizing prepulse (−1.0 microA, 0.1 millisecond) administered 1 millisecond before the test pulse had the same effect as that of the antagonist on the log m/log PA slope, and its effect was occluded by the antagonist. This prepulse was shown not to affect twin-pulse facilitation or Ca2+ currents. The overall results are compatible with the hypothesis that initiation of release at the crayfish neuromuscular junction occurs by depolarization-mediated Ca2+-independent tonic block imposed by presynaptic group II mGluRs on the release machinery.

# Abstract: THE RELEVANCE OF ACTIVIN ANTIAPOPTOTIC ACTIVITY FOR NEURODEGENERATIVE DISEASES

**Authors:** L. Kupershmidt, T. Amit, Z. Blumenfeld, M. B. H. Youdim

**Affiliations:** ^1^Eve Topf Center, Technion–Israel Institute of Technology, Haifa 32000, Israel; ^2^Rambam Hospital, Technion–Israel Institute of Technology, Haifa 32000, Israel

Activin is a member of the transforming growth factor (TGF) beta super family which comprises a growing list of multifunctional proteins that function as modulators of cell proliferation, differentiation, hormone secretion, and neuronal survival. The aim of the present study was to gain new insight into the mechanism by which processes activin A and B regulate neuronal neuroprotection. We show that activin A or B (10–25 ng per mL) significantly reduced cell death, as induced either by serum deprivation, the parkinsonism-inducing neurotoxin, 6-hydroxydopamine (6- OHDA), or the peroxynitrite donor, SIN-1 in human SHSY5Y neuroblastoma cells. We found that transient transfection of activin A and B in SH-SY5Y, rat pheochromocytoma PC12 and human U-87-MG glioma cells resulted in protection of cells from apoptosis, compared with respective cells transfected with a control plasmid. The neuroprotective effect involved inhibition of the cleavage and activation of procaspase-3 and poly ADP-ribose polymerase (PARP), induction of antiapoptotic proteins, Bcl-2 and BclxL, and reduction in the proapoptotic protein, Bad. This was accompanied by inducing the levels of tyrosine hydroxylase expression. These results indicate that both activin A and B share the potential to induce neuroprotective activity and thus may have positive impact on aging and neurodegenerative diseases to retard the accelerated rate of neuronal degeneration.

# Abstract: MODELING THE ONGOING CORTICAL DYNAMICS INHERENT IN THE LOCAL FIELD POTENTIAL IN THE MOTOR CORTEX

**Authors:** H. Lalazar, E. Vaadia

**Affiliations:** ^1^Interdisciplinary Center for Neural Computation, The Hebrew University, Jerusalem 91010, Israel; ^2^Physiology Department, Hadassah Medical School, Israel

Understanding the role of the motor cortex in volitional movements requires a model which can predict the neuronal activity around a given movement, in a single trial. However, the intertrial variability has been found to be much larger than the average response. In a series of studies, A. Arieli and colleagues have shown that a large portion of this variability can be accounted for by the spontaneous (ongoing) network state before the trial begins. To examine the nature of this ongoing activity and its relation to behavioral trials we modeled the dynamics of the ongoing network states in motor cortex inherent in the local field potentials, from simultaneously recorded multielectrodes in behaving monkeys. We used the LFP as a measure of the network state, as it is believed to reflect the synaptic activity from a large ensemble of neurons in the vicinity of the microelectrode. We trained a linear dynamical system with Gaussian noise (Kalman filter), using the LFP signal from one electrode as our observations, and learned to predict the LFP signal of another electrode— our states. After training the model on data taken from periods of ongoing activity (when the monkey is resting from the task and not moving the manipulandum), we tested the model on the trial-by-trial fluctuations (single trial minus average) during reaching trials. Our model significantly outperformed the correlations inherent between the electrodes. Thus the statistics of the ongoing activity can account for a portion of the intertrial variability during trials. We examined the stationarity of these statistics by training the model on data from different parts of the day, as well as, fromdifferent epochs of ongoing activity. In addition, we examined the predictive power of the model on data segments with oscillations in specific frequencies (eg, gamma-band oscillations), or on limited frequency bands of the LFP signal.

*Supported by a center of excellence Grant (1564/04) by the ISF, by the BMBFDIP, and by the BSF.*

# Abstract: RESPONSES TO VIRTUAL SPACE STIMULI IN THE AUDITORY CORTEX OF THE CAT

**Authors:** L. Las, A. Shapira, I. Nelken

**Affiliations:** ^1^Department of Neurobiology, The Hebrew University, Jerusalem 91010, Israel; ^2^Interdisciplinary Center for Neural Computation, The Hebrew University, Jerusalem 91010, Israel

The anterior ectosylvian sulcus (AES) contains auditory neurons whose activity is believed to be important for sound localization. However, the response properties of neurons in AES have not been characterized in detail. In particular, the location of the relevant auditory field in AES has been under some debate. We recorded neuronal activity in AES and in A1 of halothane-anesthetized cats in response to pure tones and to virtual acoustic space (VAS) stimuli that mimicked sound sources from the frontal hemisphere. Most of the neurons in A1 and in AES responded to both pure tone and VAS stimuli. Themajority of neurons that had significant response to VAS stimuli showed significant association between sound location and elicited spike counts, and sometimes also with 1st spike latency. Space-selective neurons were preferentially located in the posterior AES (pAES) where their proportion among all auditory-sensitive neurons was higher than in anterior AES (aAES) or in A1. Most A1 neurons responded preferentially to contralateral sounds. Neurons in AES had their spatial selectivity distributed more homogenously than A1, with 25 percent of the space selective neurons tuned to central locations, three times more than in A1. Furthermore, the proportion of neurons preferring frontal locations was somewhat higher in pAES compared to aAES. Thus, pAES may show a specialization for representing frontal space.

# Abstract: EVIDENCE FOR NERVE GROWTH FACTOR AND GLUCOCORTICOID RECEPTOR CROSS-TALK IN PC12 CELLS

**Authors:** S. Lecht, D. W. Fink, P. Lazarovici

**Affiliations:** ^1^Department of Pharmacology, School of Pharmacy, The Hebrew University, Jerusalem 91010, Israel; ^2^Cell Therapy Branch, Division of Cellular and Gene Therapies, FDA, Rockville, MD 20857-0001, USA

Cross-talk between signaling cascades for corticosteroid and nerve growth factor (NGF) receptors has been poorly investigated. Demonstration of interactive signaling could prove relevant with respect to the treatment of several neurological disorders. This cross-talk aspect was addressed in the model system of PC12 cells (wild type, trkA-negative, and trkA overexpressors). Chronic treatment of PC12 cell clones with the glucocorticoid dexamethasone elicited a 50% reduction in p75NTR neurotrophin receptor mRNA and protein expression levels, as evidenced by results obtained from RT-PCR, northern, and western blotting experiments. This down regulation of p75NTR was effectively antagonized by the selective antagonist for corticosteroid type-2 receptor (GR-2), mifepriston (RU-486) which is considered for Alzheimer's disease treatment. The dexamethasone-induced down regulation of p75NTR was associated with an enhancement of both basal and NGF-stimulated trkA phosphorylation. These results suggest that a transcriptional mechanism underlies glucocorticosteroid induced-down regulation of p75NTR receptor that, in turn, may affects the cooperative interaction between p75NTR and trkA receptors. Since chronic exposure to high glucocorticoid levels had been associated with attention, concentration, and memory deficits, the glucocorticosteroid induced-down regulation of p75NTR receptor, which was also reported in vivo, may represent a novel, additional mechanism contributing to the neurodegeneration.

# Abstract: FUNCTIONAL ANALYSIS OF ZEBRAFISH GnRH-I PROMOTER

**Authors:** S. Leibman-Barak, Y. Gothilf

**Affiliations:** Department of Neurobiochemistry, George S. Wise Faculty of Life Sciences, Tel Aviv University, Tel Aviv 69978, Israel

Gonadotropin-releasing hormone (GnRH) is a key peptide in the central control of reproduction in all vertebrates. There are three main populations of GnRH neurons, located in the terminal nerve ganglions (TNg), hypothalamus and midbrain (MB), which express different forms of GnRH. In zebrafish (Danio rerio) the TNg and hypothalamic populations express GnRH-I and the MB population expresses GnRHII. The embryonic origin of GnRH-I neurons is the olfactory placode. During development, they undergo posterior migration and settle in the TNg and hypothalamus. Towards understanding the molecular mechanism that determines their site-specific expression, their promoters are being functionally analyzed in vivo using transient expression assays in developing embryos. A construct expressing EGFP under the control of the GnRH-I promoter (1.3 kb) and the first intron (1.1 kb) was microinjected into zebrafish zygotes and EGFP expression was continuously documented in live embryos for several days. Specific expression was identified within the olfactory bulbs as early as 1 day post fertilization. Promoter deletions have identified a minimal promoter of 253 bp which was sufficient in driving specific expression. Further promoter deletions resulted in reduced specific expression of EGFP, increased ectopic expression, and finally in no expression. These results indicate a complex control mechanism of GnRH-I expression, probably involving multiple factors which are responsible for enhancement of specific expression of GnRH-I gene. Putative transcription factors that are involved in this mechanism will be identified and will be tested in a transgenic zebrafish line that expresses EGFP under the control of the GnRH-I promoter.

# Abstract: OXYTOCIN AND AUTISM IN A FAMILY-BASED STUDY

**Authors:** E. Lerer, N. Yirmiya, S. Levy, R. P. Ebstein

**Affiliations:** ^1^Psychology Department, The Hebrew University, Jerusalem 91010, Israel; ^2^Department of Human Genetics, The Hebrew University, Jerusalem 91010, Israel; ^3^S Herzog Memorial Hospital, Jerusalem, Israel

In the rat, the neuropeptide oxytocin (OXT) plays a central role in behavioral regulation, particularly in positive social interactions, in addition to its most well-known peripheral role in parturition and lactation. OXT is expressed in limbic as well as autonomic brain regions. OXT is also released during stress and is an important moderator of anxiety and fear responses. Notably, oxytocin infusion reduced repetitive behavior in patients with autism. Recently, a positive association of the oxytocin receptor gene (OXTR) with autism was reported in 195 Chinese Han trios. Four OXTR SNPs were examined. We now attempt to confirm as well as to extend the Chinese study by aiming at a comprehensive analysis of the oxytocin receptor gene by genotyping tagging SNPs (tSNPs) using the CEPH panel from the HapMap database. A total of 18 SNPs were selected using Haploview. Association between single SNPs and haplotypes in 132 families (152 probands fulfilling DSM IV criteria and diagnosed using ADI-R and ADOS-G) was tested using robust family-based association tests (HAPLOVIEW, UNPHASED, and FBAT/PBAT). Preliminary results from genotyping 52 families (53 probands) showed nominal association between 2 SNPs (rs237885, *P* = .02 and rs2268494, *P* = .003) and autism. Association was also found between 3 SNPs and IQ (rs2268490, *P* = .041; rs2254298, *P* = .048, and rs237889, *P* = .048). The full sample will shortly be genotyped and association will be analyzed by both categorical and quantitative (QTL) definitions of autism using both single SNPs and haplotypes.

# Abstract: PARKINSON'S DISEASE-ASSOCIATED PROTEIN DJ-1 IS PROTECTIVE AGAINST DOPAMINE TOXICITY

**Authors:** N. Lev, E. Melamed, D. Offen

**Affiliations:** Laboratory of Neuroscience and Department of Neurology, Rabin Medical Center, FMRC, Tel Aviv University, Tel Aviv 69978, Israel

Parkinson's disease (PD) is one of the most common neurodegenerative disorders and is characterized mainly by progressive loss of dopaminergic neurons. In the past decade, mutations in several genes have been identified as mediating familial PD. Mutations in DJ-1 lead to early onset autosomal recessive PD. Recently, changes in DJ-1 protein have been demonstrated in brains and cerebrospinal fluid of patients with sporadic PD, implying that DJ-1 may also have a role in the more common nonhereditary form of the illness. So far, studies have shown that DJ-1 is protective against oxidative stress, but the exact mechanism of action is unknown. The metabolism of dopamine induces formation of toxic reactive oxygen species (ROS) and their accumulation is implicated in the special sensitivity of midbrain dopaminergic neurons to oxidative stress and degeneration in PD. Here we show that exposure of human neuroblastoma cells to dopamine or to toxins implicated in PD led to rapid upregulation of DJ-1. This upregulation of DJ-1 was abolished by treatment with the antioxidant N-acetyl cysteine, indicating that DJ-1 upregulation was mediated by ROS. Overexpression of DJ- 1 increased cell resistance to dopamine toxicity and reduced intracellular ROS. Contrary effects were achieved when DJ-1 levels were reduced by SiRNA. Moreover, we found that DJ-1 affects intracellular dopamine homeostasis and reduced free intracellular dopamine. These observations suggest a novel mechanism in which ROS, generated by increased cytoplasmic dopamine, lead to rapid upregulation of DJ-1, which in turn protectively reduced free intracellular dopamine. This might explain why mutations in DJ-1 trigger early onset PD and suggest that pharmacologic upregulation of DJ-1 may represent a novel therapeutic target for PD.

# Abstract: CEREBELLAR CORTICAL NEURONS EXHIBIT BIMODALITY IN FREELY MOVING ANIMALS

**Authors:** I. Lev, G. Jacobson, Y. Yarom, D. Cohen

**Affiliations:** ^1^The Gonda Multidisciplinary Brain Research Center, Bar-Ilan University, Ramat Gan 52900, Israel; ^2^Department of Neurobiology, Life Science Institute, Edmond Safra Campus, Jerusalem 91904, Israel; ^3^The Interdisciplinary Center for Neural Computation, Edmond Safra Campus, Jerusalem 91904, Israel

The information content transferred by neurons is encoded in their time dependent firing patterns. Some neurons exhibit bimodal firing patterns in which neurons alternate between periods of high firing rates (up state) to periods of quiescence (down state). Intracellular recordings from Purkinje cells in cerebellar slices showed that the bimodal firing pattern reflects a bistable membrane potential. Recently it has been shown that the membrane potential of Purkinje cells in intact, anesthetized brain is also bistable. This finding was challenged by a report claiming that Purkinje cells in awake animals are continuously in their up state and quiescent periods of Purkinje cells could not be detected in awake animals. We reexamined this issue by implanting microwire arrays into the cerebellar cortex and recording activity of cerebellar cortical neurons while animals walked freely in their home cages. An array of 32 isonel coated tungsten microwires (35 microns in diameter) was implanted into the posterior part of the cerebellar vermis. After about 10 days of recovery, we observed high levels of spontaneous activity. The majority of the clearly sorted single units displayed epochs of high firing rates followed by prolonged quiescent periods. For the identification of Purkinje cells we compared these recordings with recordings under isoflorane anesthesia of the same animals. Although the transition from anesthetized to awake state was accompanied by an increase in firing rate, the bimodal pattern was clearly observed. These results show beyond a doubt that cerebellar cortical neurons recorded chronically in freely moving animals exhibit bimodal firing patterns.

*Supported by European Comission to the fourth author.*

# Abstract: AUTISTIC-LIKE BEHAVIOR AND ALTERED SYNAPTOGENESIS FOLLOWING PERINATAL GABA ENHANCEMENT

**Authors:** T. Levav, R. Weiss, H. M. Golan

**Affiliations:** Developmental Molecular Genetics and Zlotowski Center for Neuroscience, Ben Gurion University of the Negev, Beer Sheva 84105, Israel

Children born to women treated for epilepsy during pregnancy have an increased risk for fetal anticonvulsant syndrome. Among the behavioral problems related to this syndrome are hyperactivity and autistic-like behavior. In the present study we examined the effect of perinatal GABA enhancement on mice behavior, and the developmental regulation of the GABA synthesizing enzyme (GAD) as a possible underlying mechanism. Newborn balb/c mice were injected (subcutaneous) daily with the antiepileptic drug vigabatrin (GVG; 50mg/kg) or vehicle (CT)—during postnatal days 4 (P4)–P14. The adult mice were tested for social behavior in a three-compartment arena, GVG adult mice showed lower frequency of entries to the area consisting a cage with a mouse (*P* < .03, *F* = 4.3), and a similar number of entries to the area consisting an empty cage. GVG adult mice showed a lower rate of entries to the area with an unfamiliar mouse (*P* < .02, *F* = 5.1), while no differences were observed in entries to a familiar mouse area. In addition, hypoactivity was shown in GVG mice in all compartments, as expressed in slower movement and a shorter distance. Thus, we suggest that the lack of initiation of social interaction by GVG mice is specific to social orientated behavior. Hypoactivity of GVG adult mice correlates with previous results in the open field test. In contrary, P14 mice showed hyperactivity. Analysis of mice hippocampi by western blot showed a suppression of GAD, on P14 GVG treated newborns (65% of CT; *P* < .05). The long-term consequence on GAD was an up-regulation, as demonstrated by an increase in GVG mice (147%; *P* < .05). The synaptic vesicle protein VAMP increased in GVG adult mice hippocampi by 2 folds compared to CT (*P* <5), suggesting that the increase in GAD was due to synaptogenesis. We conclude that perinatal GABA enhancement induces differential short- and long-term behavioral modifications, which might be directly mediated by alterations in GABAergic synapses.

# Abstract: LYMPHOCYTE G-PROTEIN RECEPTOR KINASE (GRK)3 mRNA LEVELS IN BIPOLAR DISORDER

**Authors:** I. Levi, G. Shaltiel, A. Shamir, Y. Bersudsky, G. Agam

**Affiliations:** Stanley Research Center and Zlotowski Center for Neuroscience, Ben Gurion University of the Negev, Beer Sheva 84105, and Mental Health Center, Beer sheva, Israel

Linkage studies in bipolar disorder were positive for markers in the region of chromosome 22q12.1 including the gene coding for G-protein receptor kinase (GRK)3. Two of six variants of the GRK3 5-prime-UTR-promoter were reported to be associated with bipolar disorder. GRK3 protein levels in lymphoblastoid cell lines derived from bipolar patients originating from families with linkage to chromosome 22q11 were reported to be decreased compared to those of control subjects and correlated with disease severity. We compared GRK3 mRNA levels in fresh lymphocytes from 31 bipolar patients versus 26 control subjects, using real-time RT-PCR. No overall difference was found between patients and controls. However, GRK3 mRNA levels were markedly and significantly reduced in the subgroup of patients with no family history of a major psychiatric disorder compared with patients with family history. It is possible that minor gene effects contribute to the disorder in patients without family history, consistent with a more polygenic transmission. GRK3 may also fall into this category of genes.

# Abstract: ApoE4 ACTIVATES NEUROGENESIS UNDER REGULAR CONDITIONS AND TRIGGERS APOPTOSIS UPON ENVIRONMENTAL STIMULATION

**Authors:** O. Levi, D. M. Michaelson

**Affiliations:** Department of Neurobiochemistry, Tel Aviv University, Tel Aviv, Tel Aviv 69978, Israel

*Background*. Neurogenesis in the adult mammalian brain is part of a compensatory plastic-like mechanism that, under regular conditions, maintains homeostasis between neuronal cell birth and death. We have recently shown that the allele E4 of apolipoprotein E4 (ApoE4), the most prevalent genetic risk factor of Alzheimer's disease (AD), inhibits synaptic plasticity and prevents the improvements in learning and memory which are induced by environmental stimulation. *Objective(s)*. In the present study, we investigated the extent to which the cognitive and synaptic impairments of the apoE4 mice following environmental stimulation are related to the effects of apoE4 on either neuronal death or neuronal birth. *Methods*. This revealed that the level of neurogenesis in the hippocampal dentate gyrus (DG) subfield under regular conditions is elevated isoform specifically in the apoE4 transgenic mice. Furthermore, environmental stimulation, which elevated the levels of neurogenesis in the DG of apoE3 transgenic and wild-type mice, had the opposite effect on the apoE4 mice, in which it triggered apoptosis and reduced neurogenesis. Analysis of the stages of the neurogenesis cascade which are affected by apoE revealed that the first lineage, which is activated by apoE3 upon environmental stimulation, is progenitor cells which contain double-cortin and no nestin, and that the stimulation of neurogenesis by apoE4 under regular conditions is further upstream and is associated with activation of progenitor cells which contain both doublecortin and nestin. *Conclusions*. These animal model findings suggest that, whereas measures such as environmental enrichment which enhance neurogenesis may be beneficial to apoE3 positive patients, they could be harmful when applied to patients who carry apoE4. Conversely, antiapoptotic treatments may be more effective in apoE4 than in apoE3 patients.

# Abstract: INHIBITION OF THE LEFT DORSOLATERAL PREFRONTAL CORTEX PROMOTES HEALTHIER FUNCTION IN SCHIZOPHRENIA AND PROVIDES EVIDENCE FOR AMAL-CONNECTED NETWORK

**Authors:** N. Levit-Binnun, N. Handzy, I. Modai, A. Peled, E. Moses

**Affiliations:** ^1^Department of Physics of Complex Systems, Weizmann Instiute of Science, Rehovot 76100, Israel; ^2^Institute of Psychiatric Studies, Shaar Menashe Mental Health Center, Isreal; ^3^Faculty of Medicine, Technion–Isreal Institute of Technology, Haifa 32000, Isreal

The application of transcranial magnetic stimulation (TMS) to schizophrenia is a potentially far-reaching research and clinical tool, but there is no indication yet on how to access the core of this devastating disease. We previously showed that perturbation of a motor task (finger tapping) allows for the separation of motor and timing mechanisms, and reported the appearance of two abnormal finger trajectories that resulted from TMS (“stalls” and “doubles”), of which both healthy subjects and patients with schizophrenia were unaware. A phenomenon that appears only in schizophrenia patients is the appearance of cognitive lapses during finger tapping, in which the subjects miss a tap or two. Lapses were found to be correlated with patients' positive symptoms as diagnosed by PANSS tests. We report here how lapses in schizophrenia patients can be manipulated by external intervention. In the first condition the attention component was manipulated by introducing a parallel mental arithmetic task. This significantly aggravated the occurrence of lapses in patients but not in healthy controls. In the second condition the left dorsolateral prefrontal cortex (L-DLPFC) was inhibited prior to the initiation of the tapping task. Surprisingly, this almost completely relieved these lapses. Interestingly, the DLPFC has been previously associated with high executive functions and altered activation of it seems to be specific to the core disease process of schizophrenia. The fact that inhibition of the L-DLPFC improved the performance of schizophrenia subjects suggests that it is a component in the network governing finger tapping. Moreover, this network seems mal functional in schizophrenia, and removal of the L-DLPFC component can restore it to healthier function. Our results support the disconnection hypothesis of Schizophrenia and demonstrate the ability to use TMS as a means to manipulate and probe the components of a distributed network in both the healthy brain and the ill brain.

# Abstract: DIFFERENCES IN THE TMS-EVOKED EEG RESPONSES BETWEEN SCHIZOPHRENIA PATIENTS AND NORMAL CONTROLS

**Authors:** N. Levit-Binnun, V. Litvak, N. Handzy, E. Segre, M. Zaaroor, E. Moses, H. Pratt, A. Peled

**Affiliations:** *These authors contributed equally to the project.; ^1^Department of Physics of Complex Systems, Weizmann Instiute of Science, Rehovot 76100, Israel; ^2^Evoked Potentials Laboratory, Faculty of Biomedical Engineering, Technion–Isreal Institute of Technology, Haifa 32000, Isreal; ^3^Faculty of Medicine, Technion–Isreal Institute of Technology, Haifa 32000, Isreal; ^4^Evoked Potentials Laboratory, Faculty of Medicine, Technion–Isreal Institute of Technology, Haifa 32000, Isreal; ^5^Institute of Psychiatric Studies, Shaar Menashe Mental Health Center, Israel

Pathological connectivity is suggested to underlie cognitive deficits and symptoms in schizophrenia. Recently the combination of transcranial magnetic stimulation (TMS) and electroencephalography (EEG) was suggested as a means to study functional connectivity in the intact human brain. In this preliminary study a widely used 24-bit EEG recording system was used together with a novel artifact correction method to study EEG responses to TMS perturbations in patients diagnosed with schizophrenia and in healthy subjects. In healthy controls TMS stimulation at the vertex evoked a sequence of three clearly circumscribed peaks around 29ms, 43 ms, and 59 ms after the stimulus. In patients no clear response pattern was observed. Some activation peaks seen in controls were completely absent in patients while others were reduced in amplitude and differed in their scalp potential distribution. These findings demonstrate that there is a difference between healthy subjects and patients with respect to the EEG responses evoked by TMS and suggest that these responses can be used to study changes in brain connectivity and responsiveness in schizophrenia. Our observations are consistent with the results of a recent study where recordings were performed using a sophisticated artifactresistant EEG system. However, the method introduced here opens a way for a much wider group of researchers and clinicians to study TMS evoked potentials which may lead in the future to their use as a diagnostic tool in routine clinical practice.

# Abstract: ENDOGENOUS POLYAMINES REGULATE CORTICAL NEURONAL EXCITABILITY VIA ACTIVITY-DEPENDANT BLOCKADE OF VOLTAGE-GATED Na+ CHANNELS

**Authors:** L. Libman, M. J. Gutnick, I. A. Fleidervish

**Affiliations:** Koret School of Veterinary Medicine, The Hebrew University of Jerusalem, Rehovot, Israel

Our evidence in Layer 5 pyramidal cells in situ indicates that Na+ channels in the soma and proximal dendrites of these neurons produce surprisingly few late openings as compared to dissociated cell preparations. We therefore postulated that there exists a soluble factor extrinsic to the Na+ channel protein that constrains these late openings. We focus our attention on polyamine (PA) substances (spermine, spermidine, and putrescine) which are present in all eukaryotic cells, can be released from the cells, and are known to affect gating of numerous types of ion channels. Using cell-attached and whole cell recordings from Layer 5 pyramidal neurons in neocortical slices we found that partial depletion of PAs by disrupting their synthesis causes a dramatic increase in the probability of late openings of somatic Na+ channels and in the amplitude of whole cell persistent Na+ current (INaP). Restoration of PA levels by adding the exogenous PAs blocked late channel activities and INaP. Our data suggest that these effects are due to activity-dependent blockade of Na+ channels by PAs. Thus, when Layer 5 pyramidal cells were dialyzed with spermine-containing intracellular solution, there was a dramatic frequency-dependent change in action potential attributes, including a decrease in the maximumrate of rise and a depression in spike amplitudes. These changes, which were observed during 20 Hz spike trains, were very similar to the activity-dependent effects of local anesthetics and some anticonvulsants. Our findings identify a novel mechanism whereby changes in PA metabolism, either associated with normal brain states and stimuli or with pathophysiological conditions, can profoundly influence Na+ channel availability, and thereby modify neuronal excitability.

*Supported by the ISF and the United States-Israel BSF.*

# Abstract: GENETIC AND PHARMACOLOGICAL BLOCKADE OF INTERLEUKIN-1 (IL-1) SIGNALING ATTENUATES INCISIONAL PAIN IN MICE

**Authors:** D. Livshits, G. Wolf, S. Agasi, B. Beilin, R. Yirmiya, Y. Shavit

**Affiliations:** ^1^Department of Psychology, The Hebrew University, Jerusalem 91905, Israel; ^2^Department of Anesthesiology, Rabin Medical Center, Golda-Hasharon Campus, Petah Tiqva, Israel

Postoperative incisional pain is characterized by persistent hypernociception in the incision area, involving sensitization of primary nociceptors and of wide-dynamic range neurons in the spinal dorsal horn. It is also associated with the release of proinflammatory cytokines, including IL-1, following tissue damage-induced immune activation. IL-1 signaling plays an important hyperalgesic role in basal and inflammatory conditions. Specifically, we have demonstrated that mice with genetic or pharmacological blockade of IL-1 signaling exhibit reduced basal pain sensitivity, do not develop neuropathic pain following peripheral nerve injury, and display delayed and attenuated autotomy scores. The present study examined the hypothesis that IL-1 signaling also plays a role in incisional pain, using (1) mice chronically treated with IL-1 receptor antagonist (IL-1ra) via osmotic micropumps implanted 3 days prior to incision, and their vehicle-treated or untreated controls; (2) three mouse strains with genetic impairment of IL-1 signaling: deletion of the IL-1 type I receptor (IL-1rKO), deletion of this receptor with congenic background (IL-1rKOCg), or transgenic over-expression of the IL-1 receptor antagonist (IL-1raTG), and their wild-type (WT) controls. Postoperative pain was induced using the plantar incision model, and mechanosensitivity was assessed using the von-Frey filament test, before, and up to 5 days following the incision. Whereas the three wild-type strains developed significant allodynia in the operated, compared to the nonoperated, hind-paw, all 3 mutant strains did not display increased mechanical pain sensitivity in either hind-paw. Vehicle-treated mice displayed significantly increased mechanical pain sensitivity in the operated, compared to the nonoperated, hind-paw. In contrast, IL-1ratreated mice did not develop allodynia in either hind-paw. These findings indicate that IL-1 plays a pivotal role in the development of acute postoperative pain.

# Abstract: THE ROLE OF POLYUNSATURATED FATTY ACIDS IN PARKINSON'S DISEASE-RELATED MITOCHONDRIAL DYSFUNCTION

**Authors:** V. Loeb, A. Saada-Reich, D. Selkoe, R. Sharon

**Affiliations:** ^1^Department of Cellular Biochemitry and Human Genetics, The Hebrew University, Ein Kerem, Jerusalem 91010, Israel; ^2^Metabolic Disease Unit, Hadassah Hospital, Ein Kerem, Jerusalem 91010, Israel; ^3^Center for Neurologic Diseases, Harvard Medical School, Brigham andWomen's Hospital, Boston, MA 02115, USA

Parkinson's disease (PD) is a neurodegenerative movement disorder. One of the common sporadic forms of PD is linked to mitochondrial dysfunction. Specifically, reduced activity of mitochondrial complex I and increased oxidative stress are observed in brains of patients with sporadic PD. The neuronal cytoplasmic protein, alpha-Synuclein (aS), has been implicated in the pathogenesis of Parkinson's disease (PD) at both the genetic and cytopathological levels. Several lines of evidences now suggest that aS has a role in the normal mitochondrial function. Previously we have shown evidences that aS binds fatty acids and acts to enrich cellular membranes with polyunsaturated fatty acids (PUFA) as part of its physiological role. We now wish to study the possible role of aS interactions with PUFA in mitochondrial activity. Our preliminary results indicate that PUFA inhibits complex I and III, but not complex IV or citrate synthase, a matrix protein. Moreover, we found that aS over expressing cells are more sensitive to alterations of cellular PUFA concentrations. Further study is needed to elucidate the role of aS interactions with PUFA in mitochondrial activity.

# Abstract: MATCHIMIZING: A TYPE OF BOUNDED RATIONALITY

**Authors:** Y. Loewenstein, D. Prelec, H. S. Seung

**Affiliations:** ^1^Howard Hughes Medical Institute and Brain and Cognitive Sciences Department, Massachusetts Institute of Technology, Cambridge, MA 02139, USA; ^2^Sloan School of Management, Massachusetts Institute of Technology, Cambridge, MA 02139, USA

Matching and maximizing are two equilibrium models used to explain how agents distribute their choices between alternatives based on the rewards they receive. Here we show that both types of choice behaviors correspond to indifference points, in which the generalized values of the actions are equal. These generalized value functions differ in the amount of temporal discounting of future rewards. In the case of strong temporal discounting, the indifference point corresponds to matching behavior. In contrast, when the temporal discounting is weak the indifference point corresponds to maximizing. We introduce a new concept called “matchimizing,” which is an interpolation between matching and maximizing. Matchimizing corresponds to the indifference point of the generalized value function when temporal discounting is intermediate. A dynamical model of choice behavior that converges to the matchimizing indifference point is discussed. In thatmodel, the actions of the subject are generated by tossing a biased coin, where the bias of the coin is adjusted based on the product of reward and an eligibility trace that keeps track of actions in the recent past. The properties of matchimizing behavior are described for a model for addiction, in which the delivery of rewards depends not only on current action of a subject but also on its past choices.

# Abstract: OPERANT MATCHING IS A GENERIC OUTCOME OF SYNAPTIC PLASTICITY BASED ON THE COVARIANCE BETWEEN REWARD AND NEURAL ACTIVITY

**Authors:** Y. Loewenstein, H. S. Seung

**Affiliations:** Howard Hughes Medical Institute and Brain and Cognitive Sciences Department, Massachusetts Institute of Technology, Cambridge, MA 02139, USA

The probability of choosing an alternative in a long sequence of repeated choices is proportional to the total reward derived from that alternative, a phenomenon known as Herrnstein's matching law. This behavior is remarkably conserved across species and experimental conditions, but its underlying neural mechanisms are still unknown. Here we propose a neural explanation of this empirical law of behavior. We hypothesize that there are forms of synaptic plasticity driven by the covariance between reward and neural activity, and prove mathematically that matching is a generic outcome of such plasticity. Two hypothetical types of synaptic plasticity, embedded in decision making neural network models, are shown to yield matching behavior in numerical simulations, in accordance with our general theorem. We show how this class of models can be tested experimentally by making reward not only contingent upon the choices of the subject but also directly contingent upon fluctuations in neural activity. Maximization is shown to be a generic outcome of synaptic plasticity driven by the sum of the covariances between reward and all past neural activities.

# Abstract: TEXTURE SIGNALS IN WHISKERS VIBRATIONS

**Authors:** E. Lottem, R. Azouz

**Affiliations:** Department of Physiology, Faculty of Health Sciences, Ben Gurion University, Beer Sheva 84105, Israel

Rodents in their natural environment use their whiskers to distinguish between surfaces having subtly different textures and shapes. They do so by actively sweeping their whiskers across surfaces in a rhythmic forward and backward motion. To determine how textures are transformed into vibration signals in whiskers, we induced active whisking in anaesthetized rats and collected records of the natural movement of whiskers across surfaces. We found that vibrations of whiskers across different textures are translated into distinct frequency and amplitude profiles. These response profiles vary across whiskers and are dependent on the radial distance of the texture and the resonant frequency of the stimulated whisker. Finally, these response profiles are influenced by the velocity of head movements and are highly variable. These results suggest that texture discrimination may require the integration of signals from multiple vibrissae to disambiguate differences in whisker properties and spatial frequency components between textures.

*Supported by the Israeli Science Foundation.*

# Abstract: BRAIN CORRELATES OF THE ENCODING AND MEMORY OF CAMOUFLAGE IMAGES

**Authors:** R. Ludmer, Y. Dudai, N. Rubin

**Affiliations:** ^1^Department of Neurobiology, Weizamnn Institute of Science, Rehovot 76100, Israel; ^2^Center for Neural Science, New York University, New York, NY 10003, USA

Brain mechanisms of the abrupt encoding and long-term memory of brief salient stimuli are far from being understood. Camouflage images, in which the original is initially unrecognized but then suddenly identified, either spontaneously (insight) or with the aid of a visual or cognitive hint (induced insight), are particularly suitable for investigating these mechanisms. We have generated a set of camouflage images by convolving pictures of real-world scenes with a Gaussian kernel followed by black and white binarization. Behavioral experiments verified that exposure to the original produced abrupt switches from not seeing the figure in the camouflage to seeing it vividly. We used fMRI to investigate brain correlates of the encoding and longterm memory of these stimuli. During the scan, participants observed 30 images. Each trial consisted of a presentation of the camouflage, followed by a solution event in which the original and the camouflage alternated four times at 2 Hz. Finally, the participants were asked whether they recognized the object in the camouflage image before seeing the original. Participants returned a week later and performed a recognition test. The recognition of a camouflage was verified by presenting the image with a grid-map of numbers on it, and requesting the participants to identify the location of a selected feature in the image by its gridmap number. A voxel-by-voxel analysis of events that were subsequently remembered versus those not remembered revealed activation during the solution event in the right and left superior frontal gyri and in the lateral occipital cortex (LO). Weaker activations were also found in the left middle frontal gyrus, right IPS, and right precuneus. In addition, each observer was subjected to a localizer run, which is a two-condition block design, alternating between pictures of everyday objects and scrambled versions of the same objects. This analysis reveals a significant encoding effect in the LO regions.

*Supported by the NYU-Weizmann Institute Collaborative Fund in the Neuroscience.*

# Abstract: 2-AG IMPROVES COGNITIVE AND NEUROLOGICAL FUNCTION IN A MODEL OF SECONDARY BILIARY CIRRHOSIS IN THE MICE

**Authors:** I. Magen, Y. Avraham, Z. Ackerman, R. Mechoulam, E. Berry

**Affiliations:** ^1^Department of Human Nutrition and Metabolism, Hadassah University Medical School, Israel; ^2^Department of Internal Medicine, Hadassah Medical Center and The Hebrew University, Mount Scopus, Israel; ^3^Department of Medicinal Chemistry and Natural Products, Medical Faculty, The Hebrew University, Jerusalem 91120, Israel

*Background*. Hepatic encephalopathy (HE) is a major neuropsychiatric complication of both acute and chronic liver failure. However, its pathogenesis is still unknown. It has been suggested that the cognitive deficits characterizing this state result from changes in some neurotransmitter systems in the brain including the glutamatergic, cholinergic, and monoaminergic systems. Endocannabinoids (EC) function as neuromodulators via specific receptors. Recently the endocannabinoid system was found to be involved in the vasodilated state associated with liver cirrhosis. We hypothesize that it might be involved also in hepatic encephalopathy. *Methods*. Female Sabra mice were subjected to ligation of the bile duct (BDL). Sham operated animals were used as controls. 10 days post-surgery, animals receiving either vehicle or 1mg/kg 2- AG were evaluated for cognitive function in the eight-arm maze test. Neurological function was evaluated in the neurological severity score (NSS) test, 3 weeks post-surgery. The animals were sacrificed and their livers and brains were analyzed for 2-AG levels by GC-MS analysis. Oxidative stress in the liver was determined using TBARSmethod. *Results*. Brain 2-AG levels were not different between Sham and BDL mice 3 weeks post-surgery, but liver 2-AG levels elevated twofold in BDL mice in comparison to Sham mice. Cognitive and neurological functions were significantly impaired in BDL mice and 2-AG ameliorated these deficits. MDA levels in the liver, an indication to lipid peroxidation, elevated 1.5-fold in BDL mice and returned to normal values following treatment with 2-AG. *Conclusion*. These results indicate an involvement of the endocannabinoid system in the pathogenesis of HE. It seems that its activation might have therapeutic potential. 2- AG levels may rise in the brain in an earlier stage of the disease and that may be the reason for a lack of difference after 3 weeks. 2-AG elevation in the liver may have an antifibrotic effect.

# Abstract: CAN TOOL-USE CHANGE OUR MINDS?

**Authors:** T. Makin, A. McKyton, E. Zohary

**Affiliations:** ^1^Neurobiology Department, The Alexander Silberman Institute for Life Sciences, The Hebrew University, Jerusalem, Israel; ^2^Interdiciplinary Center for Neural Computation, The Hebrew University, Jerusalem, Israel

We commonly use tools to act upon objects that are outside our reach. By this, it has been claimed, we are actively extending our peripersonal space—the immediate space surrounding the body. This might be achieved by an incorporation of the tool into our body schema, such that the tool becomes an extension of our own hands. Indeed, evidences from electrophysiological, neuropsychological, and behavioural studies show that the representation of peripersonal space is modified following the use of tools. Alternatively, it has been suggested that these modifications of peripersonal space could be explained by attentional components related to the use of tools. In this fMRI study, we set out to find if the use of a tool can change the cortical representation of visual stimuli with respect to the subjects' hands. We presented three visual stimuli, located either “near,” “far,” or at an “intermediate” distance from the subjects' hands both before and after a 5-minute period of using a tool to move an object by the “far” stimulus. To control for the sensory, motor, and attentional components of the tool-use training, in an additional experiment, subjects were scanned after 5 minutes of pointing at the object while it was being moved by the experimenter. Following the control training, we found an increased BOLD response (compared to pre-tool-use) along the intrapareital sulcus (IPS) for the “far” condition. This area has previously been found to be activated specifically by visual stimuli near the hand. We also found a smaller increase for the “far” stimulus after tool-use in the anterior IPS. We suggest that the cortical changes following tool-use cannot be explained simply by the sum of sensory, motor, and attentional components of tool-use.

*Supported by Israel Science Foundation “Center of Excellence.”*

# Abstract: AUXILIARY ROLE OF SK Ca2 ACTIVATED K CHANNELS IN CONTROLLING SPIKE OUTPUT IN RAT CA1 PYRAMIDAL CELLS

**Authors:** U. Maler, Y. Yaari

**Affiliations:** Department of Physiology, Institute of Medical Sciences, Faculty of Medicine, The Hebrew University, Jerusalem 91120, Israel

Like many CNS neurons, CA1 pyramidal cells (PCs) express small conductance (SK) Ca2 activated K channels generating the medium afterhyperpolarization current (IAHP). Paradoxically, blocking IAHP with apamin does not affect spike output, in contrast to what was found in other types of CNS neurons. Thus, the function of these channels in CA1 PCs remains enigmatic. We have previously shown that KV7 (KCNQ) K channels generating the low-threshold, noninactivating M-type K current (IM), limit the magnitude of the spike afterdepolarization (ADP), thus conferring a regular firing phenotype in CA1 PCs. When IM is blocked (eg, by linopirdine), the spike ADP markedly increases in size, causing the PC to fire in burst mode. To solve the mystery regarding SK channel function in these neurons, we have put forward the following hypothesis. In normal conditions IM activates more readily than IAHP, thereby minimizing IAHPs influence on neuronal excitability. However, when IM is down-modulated, IAHP activation becomes critical for preventing excessive excitation. Indeed, using a realistic computer model of a CA1 PC, we could show that reducing IAHP hardly affects the spike ADP as long as IM is operative. However, reducing IM causes IAHP to play an increasingly more critical role in reducing spike output. Congruently, when tested in hippocampal slices, blocking IAHP with apamin had negligible effects on the spike ADP and spike output in ordinary conditions. However, when apamin was applied to linopirdine-treated neurons, spike discharge wasmarkedly enhanced. These data show that the role of SK channels in terminating spike discharge is secondary to that of KV7 channels. Only when the latter channels are down-modulated, SK channels furnish a critical repolarizing drive. Given that KV7 channels are subjected to down-modulation by many neuro-transmitters, it is likely that in some physiological situations SK channels play a critical role in controlling the spike output of CA1 PCs.

*Supported by the BSF.*

# Abstract: ENDOGENOUS AND EXOGENOUS BDNFS MEDIATE RESILIENCE TO STRESS EXPOSURE: THE EFFICACY OF TRAINING ON SYNAPTIC PLASTICITY, COGNITION, AND POSTTRAUMATIC STRESS RESPONSES

**Authors:** Y. Malka, N. Kozlovsky, Z. Kaplan, M. Matar, H. Cohen

**Affiliations:** Ministry of Mental Health Center, Anxiety and Stress Research Unit, Ben Gurion University, Beer Sheva 84105, Israel

*Background*. The neurobiological mechanisms underlying the clinical manifestations of posttraumatic stress disorder (PTSD) include changes in synaptic efficacy and plasticity in various brain areas. Brain-derived neurotrophic factor (BDNF) plays an important role in the growth, development, and function of several neuronal systems. *Aims*. To study the effect of in-context training prior to stress exposure on vulnerability and resilience to the development of acute and long-term behavioral changes in an animal model of PTSD and to seek evidence for the involvement of neurotrophic factors. *Methods*. Rats were divided into 4 *groups*. Trained group: rats were trained in the Morris water-maze (MWM) for 2 days to locate the hidden platform. Trained + stress group: rats were trained in the MWM and were exposed to stress (under-water trauma) 24 hours later. Naïve group: naïve rats. Naïve + stress group: naïve rats exposed to stress. Behavioral performance in an elevated plus-maze, acoustic startle response, and the MWM was tested 1 day and 7 days after exposure. Animals were subsequently sacrificed, and brain areas dissected and analyzed for mRNA BDNF and TrkB levels. *Results*. The expression of mRNA BDNF in the hippocampus was downregulated by exposure to stress. In contrast, in-context training prior to stress exposure prevented the stress sequelae, and was strongly correlated with enhanced BDNF and TrkB expression. Microinfusion of recombinant BDNF into the lateral ventricles prior to stress exposure attenuated the posttraumatic stress responses. *Conclusions*. We suggest that training prior to stress exposure may activate production of endogenous neurotrophic signaling in the hippocampus, and thus provides intrinsic cortical neurons with more neurotrophic support, enhancing plastic changes in synaptic function and neuronal connectivity. These changes could reinforce against the susceptibility to stress exposure or enhance recovery from the initial response.

# Abstract: AGGRESSIVE BEHAVIOR AND HPA AXIS HORMONES AFTER SOCIAL ISOLATION IN ADULT RATS OF TWO DIFFERENT GENETIC ANIMAL MODELS FOR DEPRESSION

**Authors:** O. Malkesman, R. Maayan, A. Weizman, A. Weller

**Affiliations:** ^1^Bar-Ilan University, Ramat Gan, Israel; ^2^Felsenstein Medical Center, Tel Aviv University, Israel

Behavioral and endocrinological effects of exposure to social isolation were examined during adulthood in two different genetic animal models of depression, the Flinder sensitive line (FSL) and their controls, Sprague-Dawley (SD) rats and the Wistar-Kyoto (WKY) strain and their controls, Wistar rats. Behavioral patterns of the different strains in coping with an intruder were studied in the “aggression”/residentintruder test. Basal plasma levels of the hypothalamicpituitary- adrenal (HPA) axis hormones corticosterone and ACTH were measured as well as their levels after chronic isolation stress. Significant alterations in the levels of HPA hormones after social isolation were noted in the “depressedlike” strains. There were no significant behavioral differences between FSL and SD rats in the “aggression” test. In contrast, WKY rats exhibited less frequent aggressive-like and social behavior compared to Wistar controls. The results suggest that the FSL and WKY strains, both genetic animal models of depression, exhibit separate patterns of HPA axis modulation and aggressive-like behavior after chronic stress. These different patterns may reflect two different types of depression. The clearest of these is an “avoidant” or socially-inhibited type of depressive-like behavior, observed in the WKY strain. In a previous study we observed a similar pattern in prepubertal WKY rats. Similar distinct subtypes of depression exist in human childhood and adult depression.

*Supported by ISF.*

# Abstract: GREEN TEA POLYPHENOL (-)-EPIGALLOCATECHIN- 3-GALLATE PROMOTES A RAPID PROTEIN KINASE CAND PROTEASOME-MEDIATED DEGRADATION OF BAD: IMPLICATION FOR NEUROPROTECTION

**Authors:** S. A. Mandel, L. Kalfon, M. B. H. Youdim

**Affiliations:** Eve Topf Center, Faculty of Medicine, Technion–Israel Institution of Technology, Haifa 32000, Isreal

The major green tea polyphenol, (-)-epigallocatechin-3- gallate (EGCG) exerts potent neuroprotection/neurorescue activity which is mediated by activation of protein kinase C (PKC). The aim of the present study was to gain a deeper insight into the cell signaling pathways involved in neuroprotection/ neurorescue activity of EGCG. EGCG (1 microM) caused an immediate (30minutes) down-regulation (∼40%) of Bad protein levels and a more pronounced reduction after 24 hours (55%) in the human neuroblastoma cell line SH-SY5Y. Cotreatment with EGCG and the protein synthesis inhibitor cycloheximide prominently shortened Bad halflife, with as little as 30% of its protein content remaining after 2 hours, suggesting an effect of EGCG on Bad protein degradation. Accordingly, the proteasome inhibitors, MG-132, and lactacystin blunted Bad down-regulation by EGCG. The general PKC inhibitor GF109203X or the downregulation of conventional and novel PKC isoforms abolished EGCG-induced Bad decline. However, no inhibition was seen with the cell-permeable myristoylated pseudosubstrate inhibitor of the atypical PKC isoform zeta. Enforced expression of Bad up to 72 hours rendered the cells more susceptible to serum deprivation-induced cell death, whereas EGCG treatment significantly improved cell viability (up to 1.6 fold). The present study reveals a novel pathway in the neuroprotective mechanism of action of EGCG, which involves a rapid PKC-mediated degradation of Bad by the proteasome.

# Abstract: NEURODEGENERATION RESEARCH IN THE “OMICS” ERA

**Authors:** S. A. Mandel, M. B. H. Youdim

**Affiliations:** Eve Topf Center for Neurodegenerative Diseases Research, Faculty of Medicine, Technion–Israel Institute of Technology, Haifa 32000, Israel

One major contribution of this millennium to brain research is the advent of large-scale, high-throughput analysis of neurological disorders. Genomics, transcriptomics, proteomics, metabolomics and beyond have enlightened our understanding of how normal and pathological brain aging/ degeneration is determined at a molecular level. They could complement and extend the biochemical findings that have accumulated in the past decades about the molecular pathways participating in the neurotoxic cascade leading to the demise of particular neurons in the disease-related tissues. The signatures unveiled by these approaches could provide crucial information on (i) diagnosis and development of surrogate markers for a disease, (ii) highly reliable candidate genes as predictive biomarkers to identify individuals at risk, before disease onset (early detection), and (iii) early pharmacological intervention and future development of CNS “magic bullets” targeted drugs.

# Abstract: LOCAL FIELD POTENTIAL GAMMA OSCILLATIONS DURING MOVEMENT PREPARATION: EVOLUTION ALONG TASK REPETITIONS AND CORRELATION WITH REACTION TIME

**Authors:** Y. Mandelblat, R. Paz, E. Vaadia

**Affiliations:** ^1^Department of Physiology, Faculty of Medicine, The Hebrew University, Jerusalem 91120, Israel; ^2^The Interdisciplinary Center for Neural Computation (ICNC), The Hebrew University, Jerusalem 91010, Israel

To investigate the expression of gamma oscillations, we recorded local field potential (LFPs) in M1 and compared evoked responses in 89 cortical sites in two task conditions: (1) performance of standard (STD) task and (2) adaptation to visuomotor rotation. Monkeys were over trained on the STD task but had to learn the visuomotor rotation task within a single session (Paz et al 2003). We hypothesized that the appearance of oscillations may have a relationship to motor planning and performance. We found that oscillations start to emerge after trial initiation and terminate, with low variability, after target appearance (defined as “oscillating period”). Furthermore, quite surprisingly, we found in each task condition a significant linear relationship between mean power of oscillations during oscillating period and motor reaction time (time from go signal to initiation of movement)—a high power implies a relatively long reaction time. When comparing between tasks, standard trials showed reaction time and power of oscillations lower than during visuomotor rotation trials. When examining the dynamics of oscillating period along the recording day, we discovered that both the tendency of oscillations to appear and the mean power of these oscillations gradually increase. Changes are pronounced in the beginning of the standard trials, following stabilization and subsequent changes when switching to visuomotor rotation. These results strengthen the linkage of gamma oscillations to preparatory functions, alertness, and attention. We speculate that strong oscillations may represent the recruitment of learning mechanisms and attention that can cause a longer reaction time.

# Abstract: DRUG DEVELOPMENT FOR THE PREVENTION OF SECONDARY BRAIN INJURY

**Authors:** I. Maniv, L. Veenman, A. Shterenberg, W. Kugler, M. Lakomek, M. Gavish

**Affiliations:** ^1^Technion–Israel Institute of Technology, Haifa 31096, Israel; ^2^Universitäts-Kinderklinik, 37075 Goettingen, Germany

Traumatic brain injury (TBI) may result from penetrating brain wounds or shock waves, for example, due to explosions, accidents, or violence. It is well known that the primary injury of TBI is followed for hours and days by a process of secondary injury. Mitochondria are one of the cell organelles involved in secondary brain injury. Our studies suggest that the mitochondrial translocator protein (TSPO) plays an important role in the process leading to neuronal cell death with secondary brain injury. We have shown in vivo that classical TSPO ligands, such as PK 11195, can prevent neurodegeneration due to excitatory amino acids, an important factor in secondary brain injury after TBI. We also found that reducing TSPO expression by genetic manipulation reduced apoptotic levels. In particular, such TSPO knockdown completely prevented apoptosis caused by a major contributor of neuronal cell death, the excitatory amino acid glutamate, and also by Abeta(1–42), one of the causative agents for the neuronal cell death in the neurodegenerative disease of Alzheimer. Recently, we have developed compounds that bind with high affinity to the TSPO and apparently block its apoptotic function. These novel compounds reduced basal apoptotic levels in neuronal cells, as well as apoptosis induced by glutamate, which is known to be an important causative agent for secondary brain damage, and also takes part in neurodegenerative diseases. We envision that secondary brain injury due to TBI may be prevented by providing soldiers and paramedics with one of the drugs we have developed, that is, soldiers and paramedics could carry such medication with them and use it on site. This may reduce the incidence of disabilities presently occurring in the aftermath of TBI suffered from explosions, including terrorist attacks, accidents, and other forms of violence. In addition, such a drug might also find application in the treatment of neurodegenerative diseases.

*Supported by Volkswagen-Stiftung (LV, WK, ML, MG) Center for Absorption in Science, Israel (LV).*

# Abstract: THE EFFECT OF LITHIUM ON ADENYLYL CYCLASE ISOFORMS

**Authors:** L. Mann, G. Shaltiel, E. Heldman, R. H. Belmaker, G. Agam

**Affiliations:** ^1^Department of Clinical Biochemistry, Ben Gurion University of the Negev, Beer Sheva 84105, Israel; ^2^Stanley Research Center and Mental Health Center, Beer Sheva, Israel

Lithium salts (Li) are a mood stabilizing drug used as the front-line treatment of bipolar disorder over 50 years, however, the mechanism underlying the therapeutic action of this drug is not completely understood. Li have been shown to have a significant effect on the receptor-coupled second messenger systems, the adenylyl cyclase (AC), by inhibiting the generation of cAMP. However, this effect of Li received little attention in recent years because AC is widespread, making it difficult to understand its specificity. Recently it became known that AC is the product of ten different genes encoding ten isoforms which vary in structure properties and tissue distribution. Since lithium may have differential effects on various AC isoforms, we have studied the effect of lithium on AC activity in tissue culture where cells can be transfected with a gene for a single form of AC. We further examined the effect of Li on AC inhibition by quinpirole, a D2 dopamine receptor agonist and on a phenomenon referred to as AC superactivation, reflected in an overshoot increase in cAMP accumulation occurring after chronic activation of Gi/o-coupled receptors followed by withdrawal of the inhibitory agonist. Li at 1 or 2mM did not inhibit basal AC activity of any AC isoform but selectively inhibits AC5 activity by 25–30% when stimulates by forskolin, a direct activator of ACs, implicating a direct effect of the drug on the enzymatic protein. Li significantly inhibits D1 dopamine receptor stimulated AC5 by ∼50% suggesting the involvement of Gs in this effect. Lack of an additive effect of Li and quinpirole suggests that both effectors affect AC5 by interacting with a similar site on the enzyme. Li does not attenuate AC5 superactivation. Li's selective inhibition of AC5, an isoform involved in mediation of dopaminergic transmission in the brain, suggests a possible dopaminergic region-speceific mechanism for the mood stabilizing effect of Li.

# Abstract: ASSOCIATION BETWEEN TRYPOTPHAN HYDROXYLASE 2, PERFORMANCE ON A CONTINUANCE PERFORMANCE TEST, AND RESPONSE TO METHYLPHENIDATE IN ADHD PARTICIPANTS

**Authors:** I. Manor, J. Eisenberg, S. Meidad, E. Laiba, E. Lerer, S. Israel, I. Gritsenko, S. V. Faraone, R. P. Ebstein

**Affiliations:** ^1^ADHD Center, Sackler School of Medicine, Tel Aviv University, Tel Aviv 91120, Israel; ^2^S. Herzog Memorial Hospital, Jerusalem, Israel; ^3^Human Genetics, The Hebrew University, Jerusalem 91010, Israel; ^4^Medical Genetics Research Center, SUNY Upstate Medical University, Syracuse, NY 13210, USA; ^5^Scheinfeld Center, Department of Psychology, The Hebrew University, Jerusalem 91010, Israel

*Objective*. The main objective of this study was to examine neuropsychological mechanisms mediating the association between tryptophan hydroxylase 2 (TPH2) and attention deficit hyperactivity disorder (ADHD). TPH2 encodes the rate limiting brain enzyme in the synthesis of serotonin. *Methods*. A continuous performance test (TOVA) was administered to 344 participants diagnosed with DSM IV ADHD who were also genotyped for 8 TPH2 intronic SNPs. Association between TPH2 (single SNPs and haplotypes) and ADHD and performance on the TOVA were tested using robust family-based association tests as implemented in the UNPHASED set of programs. *Results*. Weak evidence for association was observed between 7 and 8 locus haplotypes and participants with ADHD DSM IV combined type (*P* < .05). Much stronger evidence for association was observed between TPH2 single SNPs as well as haploytpes and performance on the TOVA (errors of omission, response time, and response time variability). Significant association was also observed between TPH2 and improvement in TOVA scores following acute methylphenidate (MPH) treatment. *Conclusions*. At least three studies now show association between TPH2 and ADHD. The current investigation strengthens these findings. The current first study shows that risk for ADHD conferred by TPH2 variants is partially mediated by serotonergic mechanisms impacting on some facets of executive function monitored by scores on a continuous performance test. Importantly, improvement in TOVA performance, especially on response time variability, following acute MPH administration was also associated with TPH2 single SNPs and haplotypes. Future studies could therefore gainfully address the function of serotonergic genes, and especially TPH2, in predicting the long-term efficacy of psychostimulants in treatment of this disorder.

# Abstract: IMMEDIATE POST-STRESSOR ALPRAZOLAMDOES NOT ATTENUATE BEHAVIORAL RESPONSIVENESS AND INCREASES VULNERABILITY TO REEXPOSURE IN AN ANIMAL MODEL FOR PTSD

**Authors:** M. A. Matar, J. Zohar, Z. Kaplan, H. Cohen

**Affiliations:** ^1^Anxiety and Stress Research Unit, Ministry of Health Mental Health Center, Ben Gurion University, Beer Sheva 84105, Israel; ^2^The Chaim Sheba Medical Center, Sackler Medical School, Tel Aviv University, Israel

The therapeutic value of benzodiazepines after traumatic stress has been questioned. The long-term therapeutic efficacy of a brief intervention with Alprazolam was assessed in a prospectively designed animal model for PTSD, in terms of the relative prevalence rates of severely, minimally, and partially disrupted behavioral responses thirty days after exposure to predator scent stress. The potential protective effect of the intervention was assessed by repeated exposure before and soon after treatment. Well-validated behavioral cut-off criteria were applied to observed behavioral responses on the elevated plus-maze and acoustic startle response paradigms. Alprazolam was administered intraperitoneally and compared to saline and untreated controls. The robustness of the long-term effects was assessed by freezing behavior upon exposure to a trauma-cue on day 31. No significant effect was observed in term of behavioral responses to single exposure. Cue induced freezing was significantly greater in treated individuals than in either control group. Alprazolam treatment significantly increased vulnerability for sever long-term behavioral disrupted upon re-exposure. Immediate post-stress treatment with Alprazolam did not result in improved behavioral response patterns after single exposure. Moreover, this intervention significantly increased vulnerability to exposure to a trauma-cue and even so to reexposure to the same stressor. Administration of Alprazolam in the immediate aftermath of stress-exposure may not only be ineffective, but detrimental.

# Abstract: CONNECTIVITY FROM THE RAT DENTATE NUCLEUS USINGMANGANESE-ENHANCED MRI

**Authors:** B. A. Matta, G. Goelman

**Affiliations:** Human Biology Research Center, Department of Medical Biophysics and Nuclear Medicine, Hadassah, Ein Karem, Israel

The dentate nucleus of the cerebellum and basal ganglia are both involved in motor control and play a role in motorrelated pathologies. The goal of this study is to determine the functional connections between these two regions of the brain and investigate whether changes occur in the state of Parkinson's disease. Principle component and region-ofinterest analysis of our results (*n* = 9 rats) demonstrate that manganese, an anterograde trans-synaptic neuronal marker and T1 contrast agent, injected into the dentate nucleus is taken up by neurons and moves into the contralateral superior cerebellar peduncle within 10 to 24 hours post-injection, and eventually moving out of this region over the next 72 hours. The superior cerebellar peduncle is the principle efferent bundle of the cerebellum, with neurons arising from the deep cerebellar nuclei, decuassating at the level of the inferior colliculus in the dorsal pons andmost terminating in the red nucleus or continuing to motor-related nuclei within the ventral lateral and ventral anterior thalamus which project to motor-related cortical areas. Connectivity between the dentate nucleus and globus pallidus via the thalamus has been previously demonstrated in histological studies on primates using rabies as a viral retrograde neuronal marker. In addition to corroborating with previous research, using the MEMRI technique here allows tracking of neuronal pathways over a slower time course than viral markers, in vivo. Used in this context it may provide a valuable tool in the study of motor control and functional connectivity of the basal ganglia with cerebellar nuclei.

# Abstract: EFFECTS OF STRESS EXPOSURE DURING LACTATION ON MATERNAL BEHAVIOR AND OFFSPRING RESPONSE TO STRESS IN ADULTHOOD

**Authors:** A. Mazor, M. Matar, N. Kozlovsky, Z. Kaplan, H. Cohen

**Affiliations:** Anxiety and Stress Research Unit, Ministry of Health Mental Health Center, Ben Gurion University, Beer Sheva 84105, Israel

Studies have demonstrated the critical impact of maternal behavior and specific features of maternal care on the development of individual differences in stress responsiveness of their offspring in adulthood. Pups provided more licking and grooming by their mothers during the first weeks of life showed enhance hippocampal development and function in adulthood. In contrast, lack of handling during infancy has been shown to induce higher cortisol secretion and cognitive deficits in offspring. Moreover, stress administered to pregnant rodents has been shown to lead to biological and behavioral alterations in both mother and pups, indicating that hormones of both the reproductive and the stress axes can directly and indirectly influence behavioral responses in the long term. This study sought to examine the effect of behavioral responses to early post-partum stress on the response to adulthood stress of offspring, and thereby to model a “second-generation” effect where altered maternal behavior, rather than possible neuro-hormonal effects, is assumed to be the pivotal variable. In order to model natural conditions female rats underwent a single 10-minute predator scent exposure stress at postpartum day 4. The effects of this procedure were assessed quantitatively by observing the dams' behavioral responses in the elevated plus-maze and acoustic startle response paradigms. Maternal pup-care behavior was observed and recorded.Offspring were subsequently exposed to the stress paradigm at adulthood and their responses on the EPM and ASR evaluated, and then correlated with the behavioral data for the dams. This study models a “secondgeneration” effect, that is, the effect of parental stress response on the responsiveness of offspring, in terms of parenting behavior as the pivotal variable.

# Abstract: THE COORDINATE FRAME OF POP-OUT LEARNING

**Authors:** A. McKyton, E. Zohary

**Affiliations:** ^1^Neurobiology Department; ^2^Interdisciplinary Center for Neural Computation, The Hebrew University, Jerusalem 91010, Israel

*Background*. Pop-out detection, that is, detecting a target in an array of distractors, improves with learning. This improvement, however, is specific to the target's position. In this study we further explore this position specificity to better understand the processing stage in which learning may take place. *Methods*. Subjects viewed an array of spheres that appeared briefly and was followed by a mask. In half of the times one of the spheres in a specific location was odd in its shape from shading information. Subjects judged whether this sphere was an odd man out. After a few practice sessions performance increased and then the target position was changed in one of three different ways: (1) in its retinal position; (2) in its relative position with respect to the distractors; or (3) in its position relative to the subject's head. *Hypothesis*. If learning is in a specific reference frame (retinal, objectbased, or head-based) only displacement of the target in that reference frame should lead to performance decrease. *Results*. When the target position was displaced in its retinal location compared to the learned position, performance decreased to pre-learning level. In contrast, the other manipulations did not affect performance level that was reached by the learning process. *Conclusion*. Perceptual learning of shape from shading is specific to the target retinal position but independent of the target position relative to the screen or its relative position within the elements array. This suggests that learning of complex shape features occurs in the early visual stages, in which the representation is strictly retinotopic.

*Supported by Israel Science Foundation “Center of Excellence.”*

# Abstract: UbMunc13-2 ENHANCES VESICLES RECRUITMENT IN A CALMODULIN-DEPENDENT MANNER

**Authors:** A. Mezer, D. Zikich, M. Gutman, E. Nachliel, R. Melamed, H. Junge, N. Brose, U. Ashery

**Affiliations:** ^1^Wise Faculty of Life Sciences, Tel Aviv University, Tel Aviv 69978, Israel; ^2^Max-Planck-Institute for Experimental Medicine, Goettingen, Germany

Catecholamine release from chromaffin cells is coordinated by a large number of proteins and involves the fusion of large dense core vesicles with the cell plasma membrane. We study the roles of Munc13 in exocytosis and the effects of calcium on these processes. Munc13 protein family contains a conserved calmodulin-binding domain and calcium-binding motifs. As calcium is known to accelerate vesicle priming in chromaffin cells, we investigated the physiological relevance of calmodulin binding to Munc13 activity. The exocytotic response of cells overexpressing ubMunc13-2W387R, a mutant that is calmodulin-binding deficient, was considerably lower compared to the responses of cells overexpressing the WT protein, yet still larger as compared to control cells. To evaluate if Munc13's priming activity depends on [Ca2+], exocytosis was evoked under conditions of low basal calcium concentration (< 100 nM), conditions that do not support vesicle priming. Under these conditions, both proteins showed proportionally reduced priming activity. However, when calcium was kept high for several seconds, secretion accelerated only in cells overexpressing the wild-type ubMunc13-2 (that bind calmodulin), giving rise to S-shaped release kinetics. These results demonstrate that the interaction of calmodulin with ubMunc13-2 enhances vesicle recruitment at high [Ca2+]. Next, we kinetically analyze the result using our new kinetic model that comprehensibly reproduces the dynamics of exocytosis with high accuracy. Using the model we conclude that calmodulin's interaction with ubMunc13-2 has a regulatory nature and that this interaction is important for boosting the activity of ubmunc13-2 when intracellular [Ca2+] rises. Furthermore, it seems that the ubMunc13-2 have a unique effect on the release which is different from theWT chromaffin cells.

# Abstract: ADNP mRNA KNOCK DOWN: A NOVEL APPROACH FOR BRAIN CANCER TREATMENT

**Authors:** I. Michailovici, D. Dangoor, I. Gozes

**Affiliations:** Department of Human Molecular Genetics and Biochemistry, Sackler Medical School, Tel Aviv University, Israel

Each year about 19 000 people in the United States are diagnosed with primary brain cancer. Although there is no generally accepted therapeutic management for primary brain tumors, a surgical approach is taken in most cases. However, even following an apparently complete surgical removal, tumor recurrence is still common. Therefore, novel noninvasive therapies are required for that management of brain tumors. The human activity-dependent neuroprotective protein (ADNP) gene was mapped to chromosome 20q12-13.2, a region associated with aggressive tumor growth, frequently amplified in many neoplasias. ADNP was identified as a protein secreted by glial cells that is regulated by vasoactive intestinal peptide (Bassan et al, 1999). In human and mouse brains, ADNP is expressed predominately in the cerebellum, hippocampus, and cerebral cortex (Zamostiano et al, 2001). The recent study of Steingart and Gozes (2006) showed a neuroprotective activity for the protein ADNP. Furthermore, it was demonstrated that ADNP regulates the expression level of the proapoptotic protein P53 as part of the mechanism by which ADNP protected neurons. This result corroborates previous findings showing that inhibition of ADNP expression by antisense oligodeoxynucleutides results in HT- 29 (colon cancer) cell death associated with increases in P53 expression. Here we show a dramatic enhancement of the ADNP antisense effect on HT-29 cell viability through the use of a transfection reagent that increases the uptake of the ADNP antisense into the cells. We hypothesize that due to the high expression level of ADNP in the central nervous system and due to the potent effect of ADNP knock down on cancer cell viability, ADNP antisense could present an alternative therapy for brain tumor. Future experiments are ongoing to explore the aforementioned hypothesis.

*Supported by Lily and Avraham Gildor Chair, Dr Diana, and Zelman Elton Lab and Nofar.*

# Abstract: TOWARDS LOCALIZING A SYNAPSIN-DEPENDENT OLFACTORY MEMORY TRACE IN THE BRAIN OF LARVAL DROSOPHILA

**Authors:** B. Michels, H. Tanimoto, S. Diegelmann, E. Buchner, B. Gerber

**Affiliations:** University of Wurzburg, Wurzburg, Germany

Synapsins are presynaptic phosphoproteins regulating the balance between the reserve pool and the readily releasable pool of synaptic vesicles (Hilfiker 1999 Philosophical Transactions of the Royal Society (B)). As regulation of transmitter release is a prerequisite for synaptic plasticity, we use the fruit fly Drosophila to ask whether Synapsin has a role in behavioural plasticity as well. We tackle this question for associative olfactory learning in larval Drosophila by using the protein-null mutant syn97 CS (Godenschwege et al 2004 European Journal of Neuroscience). We find that olfactory associative learning in syn97 CS larvae is reduced to approximately half of wild-type; responsiveness to the to-beassociated stimuli and all required motor faculties, however, are normal (Michels et al 2005 Learning and Memory). This learning phenotype now is the basis for localizing the cellular site(s) of Synapsin-dependent olfactory memory in the larval brain. Therefore, we have used the GAL4 binary transcription activation system to determine in which part(s) of the brain Synapsin expression is sufficient and/or necessary for memory formation. Concerning sufficiency, both almost pan-neural and mushroom body restricted expression fully rescue the memory defect of the syn97 CS mutant. The expression pattern of Synapsin in these transgenic animals is confirmed by anti-Synapsin immunohistochemistry. Therefore, the mushroom bodies are a sufficient site for Synapsin-dependent memory; this conforms with the role of the mushroom bodies in adult flies (Gerber et al 2004 Current Opinion in Neurobiology). Concerning necessity, two different approaches, with either local suppression of GAL4 by GAL80 or by RNAi are currently used to test whether mushroom-body specific knock-down of Synapsin impairs learning.

# Abstract: DUAL ROLE OF KININS IN PROSTAGLANDIN SYNTHESIS REGULATION IN ASTROCYTES

**Authors:** I. Miller, A. Danon, S. Fleisher-Berkovich

**Affiliations:** Department of Clinical Pharmacology, Ben Gurion University of the Negev, Beer Sheva 84105, Israel

Kinins, important biologically active peptides, are majormediators of peripheral inflammation. Although kinins are also present in the central nervous system, their involvement in neurological disease is not clear thus far. Kinins act via B1 and B2 receptors. B2 receptors are constitutively expressed under physiological conditions. By contrast, the B1 receptors are highly induced by proinflammatory agents. Kinins have been shown to induce cyclooxygenase-2 (COX-2) expression and synthesis of prostaglandins (PGs), major mediators of inflammation. Release of proinflammatory molecules by astrocytes and increased COX-2 expression in the brain have been implicated in a number of neurodegenerative diseases including Alzheimer's disease (AD). Furthermore, functional differences in kinase signaling induced by kinin receptors have been linked to AD. Recent studies indicate that kinins may be involved in induction of protein synthesis, pointing to long-term effect of kinins. We examined the role of BK and des-arg10-kallidin (a B1 agonist) in long-term regulation of basal and lipopolysaccharide (LPS)-induced synthesis of PGs in primary neonatal rat astrocyte cultures. Treatment of astrocytes with B1 agonist or BK for 15 hours enhanced basal PGE2 synthesis about 2 fold, as measured by radioimmunoassay. Also, BK increased LPS (0.1 μg/mL)- induced PGE2 production by 1.5 fold. However, the B1 agonist reduced LPS-induced PGE2 production by 35%. To investigate COX-2 involvement in mediating kinins effects, COX-2 protein expression was measured. Both BK and B1 augmented COX-2 levels by about 2 fold. On the other hand, while BK elevated LPS-induced COX-2 levels by 2 fold, the B1 agonist decreased LPS-induced COX-2 to control levels. Our results imply, for the first time, a dual role of kinins in regulation of inflammatory mediators in astrocytes.

# Abstract: HOWLONG AND HOWLOUD IS CONSTANT: EVIDENCE FROM THE N1 COMPLEX TO GAPS IN NOISE

**Authors:** N. Mittelman, N. Bleich, H. J. Michalewski, A. Starr, H. Pratt

**Affiliations:** ^1^Evoked Potentials Laboratory, Technion, Haifa 32000, Israel; ^2^Human Electrophysiology Unit, University of California, Irvine, CA 92697, USA

*Objective*. To study the duration and intensity combinations that determine sound as constant (rather than transient) by measuring the N1 complex (N1a and N1b) of eventrelated potentials (ERPs) to cessation of noise. *Methods*. ERPs were recorded from normal subjects in response to gaps in bursts of binaural white noise. Within each burst, the pregap noise was one of four durations at three intensities. Analysis included waveform peak measurements and intracranial source current density estimations, and statistical assessment of the effects of pregap noise parameters on N1a and N1b and their estimated intracranial source activity. *Results*. The N-Complex was detected at about 100 ms and the minimum noise duration for a double-peaked N-Complex was between tens of milliseconds and just under 500 ms, depending on noise intensity. In general, latency of N1a (at ∼90 ms) increased while that of N1b (at ∼150 ms) decreased with decreasing duration of the preceding noise. Their amplitudes were affected by the preceding noise intensity. Source current density was most prominent, under all stimulus conditions, in the temporo-parietal regions, with the first peak (N1a) lateralized to the left hemisphere and the second peak (N1b)— to the right. *Conclusions*. Constancy of sound is determined by an interaction of its duration and intensity.

# Abstract: IMAGING DENDRITIC DEVELOPMENT OF ADULT-BORN NEURONS IN THE MOUSE OLFACTORY BULB USING IN VIVO TWO PHOTON MICROSCOPY

**Authors:** A. Mizrahi

**Affiliations:** Department of Neurobiology and Interdisciplinary Center for Neuroscience, The Hebrew University, Jerusalem, Israel

The mammalian brain maintains few developmental niches where neurogenesis persists into adulthood. One niche is located within the olfactory system where the olfactory bulb (OB) continuously receives newborn neurons that integrate into the network as functional interneurons. I used in vivo two-photon microscopy of lentiviral labeled newborn neurons to directly image their development and maintenance in the OB. Time-lapse imaging of newborn neurons over several days revealed that dendritic formation is highly dynamic. I describe two distinct behaviors of dendritic development, those of spiny dendrites and those of nonspiny dendrites. Spiny dendrites are stable bearing a highly dynamic spine population. In contrast, nonspiny dendrites continuously form and retract. More mature adult-born neurons maintain high levels of structural dynamics. These experiments provide a novel experimental system to directly study the pool of regenerating neurons in the intact mammalian brain and suggest that synaptic turnover in the OB is highly dynamic.

*Supported by grants from Israeli Science Foundation and the Human Frontier Science Organization.*

# Abstract: ANALYSIS OF NONSTATIONARY RHYTHMIC PATTERNS PRODUCED BY MAMMALIAN CENTRAL PATTERN GENERATORS

**Authors:** Y. Mor, A. Lev Tov

**Affiliations:** Department of Anatomy and Cell Biology, The Hebrew University Medical School, Jerusalem 91120, Israel

A network of spinal neurons known as central pattern generator (CPG) produces the rhythmic motor patterns required for coordinated swimming, walking, and running in mammals. The output of this network is not constant. It varies with time during execution of different motor programs, during frequency modulation of neural signals (facilitation, depression, fatigue) and during the deterioration of experimental preparations. Therefore, analyses of the CPG output should take into account the nonstationary nature of the recorded signals. The present work uses uni- and bivariate short-time Fourier transform(STFT) and wavelet-transform (WT) algorithms to analyze nonstationary rhythmic signals produced in human muscles and in isolated spinal cords of neonatal rats. The STFT algorithm divides the time series into consecutive overlapping or non-overlapping windows and repeatedly applies the Fourier transform across the signal. The WT algorithm decomposes the signal using a family of wavelets varying in scale, resulting in a set of wavelet coefficients presented onto a continuous frequency range over time. Applying these techniques to computer synthesized signals and to the recorded motor rhythmic patterns mentioned above revealed that a modified Morlet WT algorithm was the tool of choice for this type of analysis. Cross-WT and wavelet coherence were then used to determine interrelations between pairs of time series in time and frequency domains and the Monte Carlo simulations against white noise were used to examine the critical value for statistical significance. The ability of the cross Morlet WT and cross-WT coherence algorithms to efficiently extract the rhythmic parameters over a wide range of frequencies (0.01–500 Hz) with time will be demonstrated and its possible implications to the analysis of complex nonstationary output of spinal pattern generators will be discussed.

*Supported by US-Israel Binational Science Foundation and the Israel Science Foundation.*

# Abstract: CAN HIGH PRESSURE MODULATION OF NMDA RECEPTOR RESPONSE EXPLAIN NEURONAL HYPEREXCITABILITY AND POTENTIAL NEUROTOXICITY?

**Authors:** A. Mor, Y. Grossman

**Affiliations:** Ben Gurion University of the Negev, Beer Sheva 84105, Israel

Hyperbaric environment (> 1.1MPa) induces the high pressure neurological syndrome (HPNS). HPNS is characterized in humans by confusion, dizziness, impairment of cognitive and motor performance, tremor, EEG changes, myoclonia, and convulsions at higher pressures. Previous studies suggested that such CNS hyperexcitability may be associated with increased NMDA receptor (NMDAR) activity. Recently, long-term exposure to high pressure was implicated with professional divers neurological and memory deficits. Glutamate excitotoxicity that induce neuronal death is also attributed to excessive Ca2+ influx through NMDAR. Therefore, we studied pressure effects on the isolated NMDAR currents. Conventional rat hippocampal coronal brain slices were prepared, constantly superfused with physiological solutions gas-saturated at normobaric pressure, and compressed up to 10.1 MPa with helium. Evoked field EPSPs (fEPSP) were recorded from the CA1 pyramidal neurons. Isolated NMDAR fEPSP were obtained under the conditions of AMPA blocking (DNQX, 20 microM), GABAA blocking (picrotoxin, 50 microM), and [Mg2+]_0_ = 0. The single fEPSP maximal initial slope (29%), amplitude (80%), decay time (95%), and (ms*mV, 350%) were significantly increased despite the known general decrease in glutamate synaptic release. When 5 stimuli at 50–100 Hz were used, high pressure increased the frequency-dependent depression (FDD) of the fEPSPs; however, the signal time integral remained the same. When 5 stimuli at 25Hz were used, high pressure induced a larger FDD, but the time integral was significantly increased by 185%. The immediate changes in NMDAR fEPSP may explain the short-term HPNS hyperexcitability. Furthermore, the robust increase in fEPSP time integral, which indicates Ca2+/Na2+ influx, suggests elevation of cytosolic [Ca2+] to toxic levels. This may provide a novel working hypothesis for explaining long-term pressure exposure deleterious effects on professional divers.

# Abstract: ALTERATIONS IN HIPPOCAMPAL LONG-TERM POTENTIATION FOLLOWING FEAR AND EXTINCTION

**Authors:** H. K. Motanis, M. Maroun

**Affiliations:** Department of Neurobiology and Ethology, Center of Brain and Behavior, University of Haifa, Haifa 31905, Israel

Predisposition for activity-induced strengthening and weakening of synaptic connections is highly dependent on the history of the synapse. It has been shown that prior activity could be achieved either by a learning paradigm or by electrical stimulation. It has been also suggested that stressful experience could also affect the ability to induce synaptic plasticity. However, most of the studies that examined the question of how stress affects long-term potentiation (LTP) and long-term depression (LTD) in the hippocampus examined the effect of an aversive and inescapable stimulus only. In the current study we assessed the potential differences that an aversive learning paradigm (contextual fear conditioning) could elicit in hippocampal LTP/ LTD as compared to exposure to elevated platform stressor. Three groups were tested: control rats, rats that were exposed to the elevated platform prior electrophysiological recording, and a group of rats that was trained for contextual fear conditioning before recording. In accordance with previous results, exposure to the elevated platform stress completely inhibited the induction of CA1 LTP compared to controls. In contrast, the level of potentiation of the rats that underwent fear conditioning was not significantly different from the controls, suggesting that stress and fear conditioning could differently affect CA1 LTP. The effect of fear conditioning learning on the induction of LTD is under current investigation.

# Abstract: WHEN SPEECH COMPREHENSION IMPEDES LISTENING ACCURACY: EVIDENCE FROM BINAURAL INEFFICIENCY MECHANISMS

**Authors:** M. Nahum, I. Nelken, M. Ahissar

**Affiliations:** ^1^Interdisciplinary Center for Neural Computation (ICNC), The Hebrew University, Jerusalem 91010, Israel; ^2^Psychology Department, The Hebrew University, Jerusalem 91010, Israel; ^3^Neurobiology Department, The Hebrew University, Jerusalem 91010, Israel

Psychophysical accounts of auditory performance usually assume that information calculated at low levels of the hierarchy is always available for perception. We now questioned this view, by systematically characterizing the conditions under which the auditory system fails to optimally utilize lowlevel binaural information for different perceptual tasks. We measured binaural advantage for speech perception in noise under diotic and dichotic listening conditions. Stimulus sets included word pairs, which were either perceptually different or perceptually similar. We manipulated factors that could potentially hamper access to low-level information: nature of perceptual task (identification or semantic-association) and the consistency of presentation of binaural information (consistent within a block or mixed). In addition, we conducted a computer simulation aimed to quantify binaural advantage given optimal information processing. We found that utilization of low-level information depended on highlevel factors. Specifically, for perceptually different words, binaural advantage was maximal (9 dB), independent of task and protocol. However, when high-level perceptual similarity was imposed, maximal binaural advantage, as predicted by the simulation, could only be attained under limited conditions: in the identification task, it was only obtained in the separate protocol. In the semantic-association task, optimal benefit was never obtained. Our findings suggest that the ability of our auditory system to efficiently utilize low-level information is restricted, and dictated by higher level factors, as perceptual similarity, rather than by low-level acoustical similarity. Moreover, they imply that when on-line access to high-level representations is required, access to precise information may be impaired, even if it is necessary for task resolution. We interpret these results in terms of reverse hierarchy theory (Ahissar and Hochstein, 1997).

*Supported by the Israeli Institute for Psychobiology and the Center of Excellence grant from ISF.*

# Abstract: PERCEPTUAL LEARNING AND THE UTILIZATION OF BINAURAL INFORMATION

**Authors:** M. Nahum, I. Nelken, M. Ahissar

**Affiliations:** ^1^Interdisciplinary Center for Neural Computation (ICNC), The Hebrew University, Jerusalem 91010, Israel; ^2^Psychology Department, The Hebrew University, Jerusalem 91010, Israel; ^3^Neurobiology Department, The Hebrew University, Jerusalem 91010, Israel

Previously, we tested listeners' ability to utilize low-level binaural information for the identification of words in noise, under diotic and dichotic listening conditions. We manipulated the consistency of binaural presentations, using two protocols: consistent, in which binaural information was either diotic or dichotic within a block, andmixed, in which diotic and dichotic trials were randomly interleaved. We found that utilization of binaural information in the mixed protocol was inefficient (5 dB compared to 9.3 dB for consistent protocol). We now tested an intermediate condition (“1-1”), in which diotic and dichotic trials were alternated throughout the block. Under this protocol, binaural advantage was inefficient, similar to that of the mixed protocol, although the binaural condition could be fully predicted on a trial-by-trial basis. We further asked whether training on the 1-1 protocol will improve the utilization of binaural information. We trained two groups of subjects for 7 successive sessions on one of the two protocols (1-1 and mixed, resp), and tested transfer of learning to the other protocol. Subjects trained on the 1-1 protocol gradually attained maximal binaural advantage (∼9 dB) within 5–7 sessions and fully transferred this benefit to the mixed protocol. In contrast, subjects trained on the mixed protocol attained smaller improvements in absolute thresholds, and gained almost no benefit from binaural information. We conclude that training can help utilize lowlevel information, which is not initially accessible when stimulus conditions vary across trials. However, improvement is contingent upon some consistent structure in the sequence of the presentation. Once acquired, this information is accessible even under variable trial-by-trial conditions, as was the case with the mixed protocol.

*Supported by the Israeli Institute for Psychobiology and the Center of Excellence grant from ISF.*

# Abstract: A NOVEL INSIDE-OUT PROSTANOID SIGNALING PATHWAY THATMEDIATES GNRH RECEPTOR AUTO-REGULATION

**Authors:** Z. Naor, M. Naidich, H. N. Jabbour, A. J. Pawson, K. Morgan, S. Battersby, M. R. Millar, P. Brown, R. P. Millar

**Affiliations:** ^1^Department of Biochemistry, Tel Aviv University, Ramat Aviv 69978, Israel; ^2^MRC Human Reproduction Sciences Unit, Edinburgh EH16 4TJ, UK

The asynchronous phased secretion of LH and FSH in response to GnRH during the female reproductive cycle is a central dogma, the mechanism of which remains unresolved. GnRH stimulates arachidonic acid (AA) release from the gonadotrope LbetaT2 cells followed by a marked induction of COX-1 and COX-2 by GnRH, which was mediated by the PKC/c-Src/PI3K/MAPK pathway but not via transactivation of the EGF receptor. COX1/2 act on AA to produce prostaglandins (PGs) and GnRH stimulates PGE2, PGI2, and PGF2alpha production. We considered that these PGs may act in an autocrine manner to regulate gonadotrope function and demonstrated that rat pituitary gonadotropes express the prostanoids receptors EP1, EP2, FP, and IP while EP3 and EP4 were localized to the prolactin and growth hormone producing cells, respectively. PGF2alpha and PGI2, but not PGE2, inhibit GnRH receptor expression through FP and IP receptors. The inhibitory effect of PGF2alpha and PGI2 seems to be mediated by inhibition of GnRHstimulated phosphoinositide turnover. PGF2alpha but not PGE2 or PGI2, reduced GnRH-induction of LHbeta, but like PGE2 and PGI2 had no effect on the induction of common alpha, or FSHbeta. PGF2alpha, or the COX1/2 inhibitor, indomethacin, inhibited and enhanced GnRH-induced LH secretion, respectively, from rat pituitaries, but both had no effect on FSH secretion. PGE2 and PGI2 had no effect on LH and FSH secretion induced by GnRH. Hence, a novel insideout signaling pathwaymediated by PGF2alpha-FP and PGI2- IP, acting in an autocrine/paracrine loop, limits GnRH regulation of the GnRH receptor, while PGF2alpha inhibits also GnRH stimulation of LH but not FSH release. This mechanism may provide a means for the cyclical responsiveness of pituitary gonadotropes and the asynchronous LH and FSH release during the female reproductive cycle.

*Supported by ISF, GIF, and Adams Super Centre for Brain Studies at Tel Aviv University.*

# Abstract: SPECIALIZED CIRCUITS RELAYING PARALLEL VISUAL PATHWAYS TO AREA MT OF MACAQUE MONKEY

**Authors:** J. J. Nassi, E. M. Callaway

**Affiliations:** ^1^Systems Neurobiology Laboratory, Salk Institute for Biological Studies, PO Box 85800, San Diego, CA 92186-5800, USA; ^2^Neurosciences Department, University of California, San Diego, CA 92093, USA

Identifying the circuits that mediate the complex interactions between magnocellular (M) and parvocellular (P) pathways, and understanding how they contribute to perception are critical to our understanding of the primate visual system. Recent studies from our lab have shown the first conclusive anatomical evidence for a strong P input to area MT of macaque monkey. Both M and P layers of the LGN connect disynaptically with areaMT, bypassing the more prominent pathway through layer 4C of primary visual cortex (V1) and likely reaching MT via Meynert cells in layer 6. Here we use rabies virus as a retrograde transynaptic tracer to study the contributions of M and P pathways to area MT through layer 4C of V1. After MT injections with a 3 day survival time, disynaptic label in V1 was found almost exclusively in M-dominated layer 4Calpha with little in P-dominated layer 4Cbeta. These results suggest that the most direct input stream through layer 4C of V1 to MT is indeed dominated by the M pathway. However, after an MT injection with a 6 day survival time, allowing rabies virus to transport across up to three additional synapses, transynaptic label was found in all layers of V1 including P-dominated layer 4Cbeta, confirming that MT receives strong indirect input from the P pathway through layer 4C of V1. In an attempt to elucidate the route by which this P pathway input reaches MT, we made injections into areas V3 and V2, which also provide input toMT. Only after certain injections into V2, likely those that involved a cytochrome oxidase (CO) thick stripe were substantial disynaptic label found in P-dominated layer 4Cbeta, making the thick stripes of V2 the most likely relay for P pathway input through layer 4C of V1 to MT. Further studies will be necessary to elucidate the functional contributions of each of these unique input pathways toMT and to better understand the complex interactions between the M and P pathways of the primate visual system.

*Supported by National Institutes of Health EY 010742, EMC; 5 T32 AG00216 and 2 T32 MH20002, JJN.*

# Abstract: RELATIONSHIP OF SINGLE-UNIT ACTIVITY TO GAMMA-LFP AND BOLD-fMRI DEPENDS ON THE DEGREE OF LOCAL COHERENCE IN THE HUMAN CEREBRAL CORTEX

**Authors:** Y. Nir, L. Fisch, R. Mukamel, H. Gelbard-Sagiv, A. Arieli, I. Fried, R. Malach

**Affiliations:** ^1^Department of Neurobiology, Weizmann Institute of Science, Rehovot 76100, Israel; ^2^Ahmanson Lovelace Brain Mapping Center, University of California, Los Angeles, CA 90095, USA; ^3^Division of Neurosurgery, University of California, Los Angeles, CA 90095, USA; ^4^Functional Neurosurgery Unit, TLV Medical Center and Sackler School of Medicine, Tel Aviv, Israel

The relationship between the firing rate of individual neurons, the gamma-band power in the local field potential (LFP), and the blood oxygenation level-dependent (BOLD) fMRI signal is of high significance, both theoretically and methodologically. Recently, conflicting measures of this coupling have been reported. Here we show, by simultaneously recording single-unit and gamma-LFP from multiple electrodes in human auditory cortex, that the coupling of individual neurons to gamma-LFP is variable.However, this variability could be largely accounted for (*r* = 0.66, *p* ≪ 0.001) by the extent of correlated activity between neighboring neurons. When comparing single-unit activity to BOLD signals, the neuron-to-BOLD coupling level could similarly be explained by the degree of correlated activity among neighboring neurons. Thus, our results suggest that the gamma-LFP and the BOLD signal correlate to the neuronal population firing rate, and instances where they appear not to correlate to single-unit activity may be due to locally decoherent firing patterns.

*Funded by ISF COE and Dominique Center to R. Malach and US-Israel BSF grant to I. Fried and R. Malach.*

# Abstract: STIMULUS-SPECIFIC ADAPTATION IN THE SOMATOSENSORY THALAMUS

**Authors:** V. Nitzan, R. Azouz

**Affiliations:** Department of Physiology, Faculty of Health Sciences, Ben Gurion University, Beer Sheva 84105, Israel

Sensory inputs from the whiskers reach the primary somatosensory thalamus through the principal trigeminal nucleus. The main role of the thalamus is to relay these sensory inputs to the cortex. One important aspect of sensory information flow that is particularly sensitive to adaptation is the relay of high-frequency inputs. To determine the role of adaptation in sensory information transmission, we performed extracellular recordings from the ventral posterior medial nucleus of the thalamus of the anaesthetized rat while presenting simulated texture stimuli. We found, as others, that thalamic responses depress with repetitive constant velocity whisker stimulation. In contrast, however, a change in stimulus velocity relives this depression. Even more surprising is the fact that neuronal responses hardly ever adapt to complex whisker deflections which contain a broad range of whisker velocities. These results suggest that thalamic neurons may integrate synaptic inputs which may carry distinct attributes of unitary stimulus.

*Supported by the Israeli Science Foundation.*

# Abstract: ANTI-INFLAMMATORY PROPERTIES OF CHOLINERGIC UPREGULATION: A NEW ROLE FOR ACETYLCHOLINESTRASE INHIBITORS

**Authors:** E. Nizri, Y. Hamra-Amitai, C. Sicsic, I. Lavon, T. Brenner

**Affiliations:** Laboratory of Neuroimmunology, Department of Neurology, Hadassah Hebrew University Medical Center, Israel

We investigated the anti-inflammatory effects of acetylcholinesterase inhibitors (AChEI) at the cellular and molecular levels. AChEI suppressed lymphocyte proliferation and proinflammatory cytokine production, as well as extracellular esterase activity. Anti-inflammatory activity wasmediated by the alpha-7 nicotinic receptor (neuronal) acetylcholine receptor; the muscarinic receptor had the opposite effect. Treatment of the central nervous system (CNS) inflammatory disease, experimental autoimmune encephalomyelitis (EAE), with EN101, an antisense oligodeoxynucleotides, targeted to AChEmRNA, reduced the clinical severity of the disease and CNS inflammation intensity. The results of our experiments suggest that AChEI increase the concentration of extracellular acetylcholine (ACh), rendering it available for interaction with a nicotinic receptor expressed on lymphocytes. Our findings point to a novel role for AChEI which may be relevant in CNS inflammatory diseases such as EAE and multiple sclerosis. They also emphasize the importance of cholinergic balance in neurological disorders, such as Alzheimer's disease and Myasthenia Gravis, in which these drugs are used.

# Abstract: THE EFFECT OF VARIOUS ANTIDEPRESSANTS ON THE IGF-1 SYSTEM IN RAT BRAIN AND IN A HUMAN GLIOMA CELL-LINE: RELEVANCE TO NEUROGENESIS

**Authors:** N. Novak, M. Taler, R. Zilberman, A. Weizman, I. Gil-Ad, R. Weizman

**Affiliations:** ^1^Laboratory of Biological Psychiatry, FMRC, Rabin Medical Center, Tel Aviv University, Tel Aviv 69978, Israel; ^2^Brull Community Mental Health Center, Sackler Faculty of Medicine, Tel Aviv University, Tel Aviv 69978, Israel

Antidepressants were found to facilitate synaptic connections, neuroplasticity, and cognition by stimulating trophic factors such as BDNF. Insulin-like-growth-factor-1 (IGF-1) is a potent neurotrophic factor in the brain. Previous studies have demonstrated that IGF-1 accelerates brain growth and neuroplasticity. IGF-1 is regulated by different neurotransmitters such as norepinephrine (NE) and serotonin (5-HT). Our aimwas to evaluate the effect of various antidepressants, which act differently on 5-HT and on NE neurotransmitters, on the expression of IGF-1 mRNA and receptor (IGF- 1R) in different regions of the rat brain and in U87, a human glioma cell-line.Hypothalamus, frontal cortex, and hippocampus were dissected fromWistar male rats treated with clomipramine, reboxetine and fluoxetine administered orally (15mg/kg × 3 days or 21 days). In addition, U87 cells were treated with the same antidepressants (0.1micM–20 micM). IGF-1R expression was determined by Western blot analysis. IGF-1 mRNA levels were assessed by semiquantitive PCR reaction. After 3 days, reboxetine and fluoxetine slightly decreased IGF-1R in the hypothalamus. After 21 days, reboxetine and fluoxetine increased IGF-1 mRNA in the frontal cortex and decreased it in the hippocampus. Clomipramine increased IGF-1R and reduced IGF-1 mRNA in the frontal cortex. In the hippocampus, clomipramine decreased IGF-1 mRNA. In U87 cells a high concentration of clomipramine increased IGF-1R. Reboxetine and fluoxetine increased IGF- 1 mRNA and IGF-1R at low concentrations, and decreased both parameters at high concentrations. *Conclusions*. Different antidepressants affect the IGF-1 system in different brain areas in various ways. Reboxetine and fluoxetine seem to possess the same pattern of activity. After 21 days, in U87 cells, a biphasic effect was observed with reboxetine and fluoxetine. We suggest that antidepressants, mainly selective 5-HT and NE modulators, may affect neurogenesis by regulating the IGF-I system.

*This research is supported by the National Institute for Psychobiology in Israel.*

# Abstract: A POTENTIAL EXISTENCE OF A NON-REWARD-RELATED REINFORCEMENT DURING PERFORMANCE OF REACHING MOVEMENTS

**Authors:** I. Novick, F. I. Arce, E. Vaadia

**Affiliations:** ^1^Department of Physiology, The Hebrew University, Jerusalem 91010, Israel; ^2^Interdisciplinary Center for Neural Computation, The Hebrew University, Jerusalem 91010, Israel

The conventional way to explain an increasing probability of a behavior appearance is strengthening the connection between a stimulus and a certain response, as a consequence of a positive reward that was later achieved. The existence of habits and even the appearance of repetitive behaviors that seem to give rise to negative reward are usually also explained by an invisible positive reinforcer. However, other processes could result in an increasing probability to execute a response in addition to the reward system. In this study, we tested reinforcement that is related to iterative behavior. Twenty human subjects volunteered to perform hundreds of repetitions of a simple reaching movement. Analysis of behavioral parameters (eg, maximal velocity, repeating an error) in consecutive trials, as well as parameters of EMG activity, reveals high tendency to repeat a behavior, not only as a result of a positive reward, suggesting the existence of an additional component in the classic reinforcement learning model.

*Supported by Bundesministerium fur Bildung und Forschung (BMBF-DIP), BSF, Jack Skirball Chair and Research Fund.*

# Abstract: BRAIN ACTIVITY RELATED TO POSITIVE AND NEGATIVE SUBJECTIVELY SIGNIFICANT VERBAL STIMULI: AN ERP FUNCTIONAL IMAGING STUDY

**Authors:** E. Ofek, H. Pratt

**Affiliations:** Evoked Potentials Laboratory, Medical School, Technion–Israel Institute of Technology, Haifa 32000, Israel

*Objective*. Study neural correlates of subjectively positive and negative verbal stimuli, using the high temporal resolution of ERP (event-related potentials) and LORETA (low-resolution electromagnetic tomography). We have previously shown brain response to negative stimuli, here we compare the response to positive and negative stimuli. *Methods*. ERPs were recorded from twelve right-handed subjects while listening to first names and detecting 3 emotionally neutral names that ended with a specific consonant from a list of 25–30 names. Subjective affective valence of verbal distracters was determined separately for each subject after the experiment, using a structured interview based on a validated questionnaire. For each subject, for each name, 3 scores were computed based on the interview results: general subjective significance, negative valence, and positive valence. For each subject, the name of the person who hurt the subject the most was chosen as the negative stimulus. A name with the highest positive valence score and the lowest negative valence score was chosen for each subject as the positive stimulus. Event-related potentials (ERPs) were averaged separately for each name, for each subject. The time courses of brain activity to names with subjectively negative and positive valence were compared to neutral names using LORETA. *Results*. Enhanced activation to stimuli with subjectively negative and positive valence (compared to neutral stimuli) involved prefrontal cortex and auditory cortex and lasted until at least 950 ms after stimulus onset. In general, activity was lateralized to the left hemisphere (mainly prefrontal cortex) for negative stimuli, and to both hemispheres for positive stimuli. *Conclusions*. Brain response to subjectively negative stimuli is more extended and bilateral compared to subjectively positive stimuli. It seems possible to differentiate the subjective affective valence of stimuli based on event related potentials.

# Abstract: THE ROLE OF ZINC TRANSPORTERS IN ZINC AND HEAVYMETAL HOMEOSTASIS IN CORTICAL NEURONS

**Authors:** E. Ohana, M. Hershfinkel, I. Sekler

**Affiliations:** ^1^Department of Physiology, Faculty of Health Sciences, Ben Gurion University of The Negev, PO Box 653, Beer Sheva 84105, Israel; ^2^The Zlotowski Center for Neuroscience, Ben Gurion University of The Negev, Beer Sheva 84105, Israel

The large zinc gradient across the plasma membrane of neurons in several areas of the brain, taken together with zinc toxicity, suggests that zinc permeation and sequestration into organelles are highly regulated. Indeed, numerous zinc regulating proteins are known, most prominently the ZnT family. Using cellular imaging together with molecular tools for overexpression and silencing, we have identified the cellular mechanisms which underlie the reduction of toxic Zn2+ accumulation. The cellular system which maintains Zn2+ homeostasis consists of plasma membrane proteins, which regulate zinc influx or extrusion and intracellular transporters that sequester zinc into intracellular organelles. Our work focuses on three major proteins, resembeling each of these “modes”: the Na+/Zn2+ exchanger and ZnT-1 on the plasma membrane, and ZnT-5 on vesicular membranes. We show that a Na+/Zn2+ exchanger mediates an active extrusion of Zn2+ and that ZnT-1 modulates the LTCC (L-type calcium channel) in neurons and thus reducing Zn2+ influx through this channel. In addition, we show that ZnT- 5 actively sequesters Zn2+ into intracellular compartments. Working together, these proteins make a robust systemwhich reduces cytoplasmic zinc concentrations, thus protecting the cells from Zn2+ toxicity.

# Abstract: THE DYNAMICS OF ONGOING ACTIVITY IN THE PRIMARY VISUAL CORTEX OF THE AWAKE MONKEY

**Authors:** D. B. Omer, L. Rom, A. Grinvald

**Affiliations:** Department of Neurobiology, Weizmann Institute of Science, Rehovot 76100, Israel

What happens in primary sensory areas in the absence of sensory input (eg, primary visual cortex when eyes are closed)? Previous findings from voltage sensitive dye imaging (VSDI) experiments done on anesthetized cats (Grinvald et al, 1988; Arieli et al, 1995; Arieli et al, 1996; Tsodyks et al, 1999; Kenet et al, 2003) indicated that activity in the visual cortex depends not only on the nature of visual inputs but also on the state of the cortex at the time of stimulation. Furthermore, patterns that looked like orientation columnsmaps appeared spontaneously. Do those spontaneous cortical states appear also during the awake state and have any functional significance? We combined simultaneous VSDI with electrophysiological recordings of local field potentials (LFP) and multiunit activities. During ongoing activity sessions, the VSDI revealed coherent spatio-temporal activity over the primary visual area. We found that in comparison to the ongoing activity in the anesthetized preparation, the dynamics of the ongoing activity in the awake monkey is much faster, and the coherence-length is much smaller. We also detected in the LFPs short episodes of high-energy oscillations in the ∼30 Hz range. Those short episodes were not detected in the evoked sessions, in contrast with the situation reported for anesthetized cat (Gray and Singer 1989). During the evoked sessions, cortical columns with similar orientation preference were phase coherent. We observed a clear phase shift for the orthogonal orientation columns in V1. We report here that the averaged coherence over space between the VSD signals and single unit activity was significantly higher than between VSD-VSD signals and VSD-LFP signals. The cortical evoked response showed maximal coherence between

*Supported by grants from the ISF, EU, the Goldsmith Foundation, and the Grodetsky Center.*

# Abstract: TRP CHANNELS: KEY PROTEINS OF SIGNAL TRANSDUCTION

**Authors:** M. Parnas, B. Katz, B. Minke

**Affiliations:** Department of Physiology, Faculty of Medicine, The Hebrew University, Jerusalem 91120, Israel

Transient receptor potential (TRP) channels are universal biological sensors that detect changes in the environment in response to myriad of stimuli including cold/hot temperatures, natural chemical compounds, mechanical stimuli, or changes in the composition of the lipid bilayer. TRP channels are crucially involved in physiological processes, for example, photoreception, pheromone sensing, taste perception, thermosensation, pain perception, mechanosensation, perception of pungent compounds, renal Ca2+/Mg2+ handling, smooth muscle tone, and blood pressure regulation. The light activated channels of*Drosophila* photoreceptors TRP and TRP-like (TRPL) show voltage-dependent conductance during illumination. Recent studies conducted on expressed channels implied that mammalian members of the TRP family, which belong to the TRPV and TRPM subfamilies, are intrinsically voltage-gated channels. These channels reveal temperature and agonist-dependent shift of their voltage dependence suggesting that voltage dependence underlies their gating mechanism. It is, however, unclear whether other subfamilies of TRP channels share the same voltagedependent gating mechanism. Exploring the voltage dependence of *Drosophila* TRPL channel, we found that it is not an intrinsic property. Furthermore, Ca2+ blocked the TRPL channels with high affinity via an open channel block mechanism, which underlies the hitherto unknown mechanism of TRPL voltage dependence. A mathematicalmodel describing channel-Ca2+ interaction indicated that it is the dissociation of Ca2+ from the open channel that underlies the observed voltage dependence of the channel. Whole cell recordings from a Drosophila mutant expressing only the TRPL channels indicate that Ca2+ block also accounts for the voltage dependence of the native TRPL channels in the photoreceptor cells. The relatively low concentration of Ca2+ required for blocking TRPL improves the signal-to-noise ratio of the light response during medium and intense lights.

# Abstract: DESCRIBING PARAMETERS DISTRIBUTION AND ROBUSTNESS FACTORS IN COMPARTMENTAL MODELS OF NEURONS

**Authors:** N. Peled, A. Korngreen

**Affiliations:** ^1^The Mina and Everard Goodman Faculty of Life Sciences, Bar-Ilan University, Ramat Gan, Israel; ^2^The Leslie and Susan Gonda (Goldschmied) Multidisiplinary Brain Research Center, Bar-Ilan University, Ramat Gan, Israel

Our understanding of the input-output function of single cells has been substantially advanced by accurate biophysically multicompartmental models. The large number of parameters in these models has raised the necessity to use automatic approaches for finding their true values. This approach attempts to converge to a global minimum in a very high dimensional parameters' phase-plane. Due to an inherent noise in the neurophysiologic measurements equipment, we cannot be sure how accurate the values that we converged to are. Here we suggest that finding the parameters' distribution via Monte Carlo approach can tell us how much each parameter is sensitive to noise, and how its value distribute in real neurons. After finding the parameters' distributions, we now have a much smaller parameters-plane, which allows rigorous methods to find: (1) the sensitiveness of the model to each parameter; (2) how the quantitative variance of the data obtained can be explained by each parameter. Finally, we suggest applications, based on these methods: (1) an iterative method of dealing with the difficulty of convergence to a global minimum in a high-dimensional parameters-plane; (2) defining the amount of information obtainable when using a specific model; (3) separating between similar and nonsimilar global minimum.

# Abstract: OPTIMIZERS 2050: THE FUTURE OF PSYCHIATRY

**Authors:** A. Peled

**Affiliations:** ^1^Technion–Israel Institute of technology, Haifa 32000, Israel; ^2^Shaar Menashe MHC, Israel

Psychiatry suffers from a lack of a scientific brain-related etiological knowledge about mental disorders. The advancement toward such knowledge is further hampered by the lack of a theoretical framework or “language” that translates clinical findings of mental disorders to brain disturbances. Correlates between clinical manifestations and presumed neuronal network disturbances are proposed in the form of a practical diagnostic system titled “Brain Profiling.” Three dimensions make up Brain Profiling, “neural complexity disorders,” “neuronal resilience insufficiency,” and “context-sensitive processing decline.” The first dimension relates to disturbances occurring to fast neuronal activations in the millisecond range, it incorporates connectivity and hierarchical imbalances appertaining typically to psychotic and schizophrenic clinical manifestations. The second dimension relates to disturbances of long-term synaptic modulations, and incorporates disturbances to optimization and constraint satisfactions within relevant neuronal circuitry. Finally, the level of internal representations related to personality disorders is presented by a “context-sensitive process decline.” Brain profiling offers testable predictions about the etiology of mental disorders because being brain-related it lends readily to brain imaging investigations. Brain profiling follow-up can be presented by a trajectory in a threedimensional space representing an easy-to-see clinical history of the patient. Brain profiling relates to brain disorder rather than person disorder thus less stigmatic. Brainprofiling diagnosis of mental disorders could be a bold new step toward a future neuropsychiatric diagnosis of mental disorders. In the future psychiatrists will be called “Optimizers” because they will cure mental disorders by optimizing brain organization.

# Abstract: EXTERNAL CALCIUM CONTROLS SLOWINACTIVATION GATING OF A POTASSIUM CHANNEL

**Authors:** A. Peretz, L. Shamgar, Y. Haitin, G. Gibor, Y. Paas, B. Attali

**Affiliations:** ^1^Department of Physiology and Pharmacology, Sackler Faculty of Medicine, Tel Aviv University, Tel Aviv 69978, Israel; ^2^The Mina and Everard Goodman Faculty of Life Sciences, Bar-Ilan University, Israel

Inactivation is an inherent property of most voltage-gated K+ channels (Kv). Inactivation of Kv channels can occur by N-type or/and C-type mechanisms. Inactivation of KCNQ1 (Kv7.1) channels does not exhibit the hallmarks of N- and C-type inactivation. Inactivation of wild-type KCNQ1 channels is invisiblemacroscopically (hidden) but can be revealed by hooked tail currents which reflect recovery from inactivation. Here, we found that removal of external Ca2+ produced a striking voltage-dependent macroscopic inactivation in WT KCNQ1 channels when expressed in CHO cells. Adding external Ca2+ suppresses the macroscopic inactivation with an EC50 of 1.5 micromolar and leaves intact the hidden inactivation. This modulation of macroscopic inactivation was specific for Ca2+, as adding external Mg2+ and other divalent cations, except for Sr2+, could not suppress the macroscopic inactivation induced in Ca2+-free solutions. Interestingly, high external K+ (50mM) did not prevent the macroscopic inactivation evoked in Ca2+-free solutions. We show that in WT KCNQ1, two distinct inactivation processes coexist in Ca2+-free solutions, a hidden and a macroscopic inactivation, which display distinct kinetics. We elaborated a kinetic model that closely matches the experimental currents, assuming two closed states (C1 and C2), two voltage-dependent open states (O1 and O2), a fast voltage-independent inactivated state I2 (“hidden” inactivation) and a slow voltage-dependent inactivated state I1 (slow macroscopic inactivation). Mutagenesis studies and structural modeling indicate that external Ca2+ ions are tightly coordinated by two glutamate residues located at the outer pore in the turret region. In all, our results reveal a new mechanism of inactivation whereby external Ca2+ exquisitely controls KCNQ1 channel gating by preventing relaxation into slow macroscopic inactivation.

# Abstract: PRETREATMENT WITH CLOZAPINE BUT NOT PAROXETINE PREVENTS DISRUPTION OF LATENT INHIBITION INDUCED PRENATALLY BY POLY I : C

**Authors:** Y. Piontkewitz, I. Weiner

**Affiliations:** Department of Psychology, Tel Aviv University, Tel Aviv 69978, Israel

There is an increased interest in the prodromal stage of schizophrenia as an optimal stage to begin intervention with antipsychotics. Neurodevelopmental models of schizophrenia, which mimic the characteristic maturational delay of the disorder, offer a valuable tool for the investigation of preventive interventions. We previously showed that a single exposure of pregnant rat dams to the cytokine-releaser, polyinosinic-polycytidilic acid (poly I : C; 4 mg/kg) on gestation day 15, led in the offspring to postpubertal emergence of impaired capacity to ignore irrelevant stimuli, as manifested in the loss of latent inhibition (LI).Here we used this prenatal poly I : C model to test the capacity of pretreatment with the atypical antipsychotic clozapine or the antidepressant paroxetine on postnatal days 35–47, to prevent LI disruption at adulthood. LI, indexed as poorer fear conditioning of rats that received nonreinforced tone exposure prior to conditioning compared to rats for whom the tone was novel, was assessed in 90 days old female and male offspring of salineinjected dams pretreated with saline and the offspring of poly I : C-injected dams pretreated with clozapine (7.5 mg/kg), vehicle, paroxetine (5 mg/kg), or saline (*n* per group = 16). LI was present in the offspring of saline-injected dams pretreated with saline but was disrupted in the offspring of poly I : C-injected dams pretreated with saline or vehicle. Clozapine, but not paroxetine, prevented the prenatal poly I : Cinduced LI disruption in the adult offspring (preexposure × pretreatment interaction, *P* < .0025). These findings support the predictive validity of the poly I : C model in identifying treatments preventing first-episode psychosis.

# Abstract: MODIFYING MEMORIES BY GRADUAL EXPOSURE TO MORPHED STIMULI

**Authors:** S. Preminger, D. Sagi, M. Tsodyks

**Affiliations:** Weizmann Institute of Science, PO Box 26, Rehovot 76100, Israel

Our memory system is constantly updated to capture gradual changes in visual stimuli such as aging or maturing of faces. To investigate the mechanisms that underlie the ability of the brain to maintain such an adaptable representation of visual objects we designed the following psychophysical paradigm. The memory of a face was established by training observers to recognize a small set of faces by names. Subsequently, for multiple daily sessions, observers repeated a task of identifying whether a presented face belonged to the memorized set or not. Stimuli were presented in randomly interleaved trials of memorized faces, new faces, and faces from a morph sequence—face stimuli gradually transforming from one memorized face (source) to another distinguishable, nonmemorized face (target). In the present study, in each daily session observers were exposed only to a portion of the morph sequence: starting from the source, every day the morph faces that were shown drifted slightly towards the target. Initially, only the morph images close to the source were identified as belonging of the memorized set. As the task continued, for 3 out of 4 observers this protocol led to a gradual drift in the identification of the morph sequence— gradually additional morph images were identified as memorized, until at the end of practice even the target face was identified as memorized. After the practice these observers named the target as the source, rated all images of the morph sequence as very similar to their memory of source, and, unlike with the previous repetitive protocol (ISFN 2005), rated the source as less familiar than before practice. These results point to the potential of this protocol to implicitly affect memory of prelearned faces, and demonstrate that longterm memory of objects can be modified by exposure to stimuli of gradually changing similarity. The results will be discussed within the context of an attractor-based neural network with novelty-dependent learning.

# Abstract: EVIDENCE THAT PRENATAL MATERNAL SSRIS THERAPY INDUCES CHANGES IN ANTHROPOMETRICAL PARAMETERS AND IN THE HORMONAL PROFILE OF NEWBORNS

**Authors:** D. Prokonov, R. Maayan, I. Gil-Ad, D. Harel, A. Weizman, S. Davidson

**Affiliations:** ^1^Department of Neonates, Beilinson Medical Center, Campus Rabin, Petah Tiqva, Israel; ^2^Laboratory of Biological Psychiatry, FMRC, Beilinson Medical Center, Tel Aviv University, Israel; ^3^Laboratory of Biochemistry, Beilinson Medical Center, Campus Rabin, Petah Tiqva, Israel

The data on the somatic growth of infants exposed to selective serotonin reuptake inhibitors (SSRIs) in utero is limited and controversial. There is also a very limited data on the hypothalamic-pituitary-adrenal (HPA) axes in SSRIs exposed neonates. The aim of our study was to determine the anthropometrical parameters and the hormonal profile of infants exposed to prenatal maternal SSRIs. Twenty two infants of mothers treated with different SSRIs during pregnancy and 20 control infants were included in the study. Cord blood level of cortisol, IGF-1, Prolactin, TSH, DHEA, DHEAS, and urine 5HIAA (serotonine metabolite in the urine) were determined in the two groups. Neonatal withdrawal symptoms were obtained in the SSRIs group using the Finnegan score. We have found in newborn males that birth weight, length, head circumference as well as IGF-1 and DHEAS levels were significantly lower than parameters found in males control group. In female neonates, length and cortisol levels were significantly lower than in female controls. In both sexes of SSRIs-exposed neonates, plasma TSH levels were significantly higher than in controls and a total of 27% of the newborns showed hypothyroidism. In the SSRIs infants, the Finnegan score was positively correlated, though not significantly, with cord blood cortisol (*r* = 0.4, *P* = .09) and negatively correlated, close to significance, with ECG QTc interval (*r* = −0.45, *P* = .07). Birth weight was positively correlated with cord IGF-1(*r* = 0.6, *P* < .005) and negatively correlated with urine 5HIAA(*r* = −0.52, *P* < .02). Cord IGF-1 and urine 5HIAA were negatively correlated (*r* = −0.42, *P* = .06). In conclusion, infants exposed to SSRIs in utero show impaired intrauterine growth, a tendency toward decreased IGF-1, and high frequency of hypothyroidism. It seems that follow-up of these children is of great importance.

# Abstract: MEMORY COMPENSATION IN THE BLIND: A SPECIFIC ADVANTAGE IN SERIAL MEMORY

**Authors:** N. Raz, E. Striem, G. Pundak, T. Orlov, v Zohary

**Affiliations:** Hebrew University, Jerusalem 91010, Israel

In the absence of vision, which naturally provides a full picture of the scene, the perception of space is highly dependent on memory. In specific, serial memory, indicating the order in which items are encountered, may be especially important for the blind to generate a mental picture of the world. We compared the performance of 19 congenitally and totally blind participants and 19 sighted controls in two types of memory tasks: item memory (IM) and serial memory (SM). We found that on average the congenitally blind are better than their sighted peers in both item memory and serial memory tasks. However, the blind's advantage is far greater in serial memory, and it is most pronounced in the recall of long sequences of words in their correct order. The blind are superior to sighted in their serial memory capabilities even in conditions in which IM performance is equal in the two groups, indicating that their SM advantage is not a byproduct of better item memory. Furthermore, the blind improve their performance across repetitions in both IM and SM more than their sighted peers. Once again, the blind's superior improvement (compared to the sighted) is the greatest in SM while it is more modest in the IM tasks. Blind's superior improvement is even more conspicuous for the recall of long sequences. We argue that the blind' clear advantage in SM capabilities is a result of their blindness: unlike their sighted peers, blind people typically experience the world as sequences of events. Holistic impressions of scenes are therefore highly dependent on memorizing the events according to their serial order. We conclude that blind's superior performance in serial memory is therefore a classical case of cognitive compensatory adjustment.

*Supported by Israel Science Foundation “Center of Excellence” and US-Israel Binational Science Foundation.*

# Abstract: MULTIPLE PATHWAYS FOR NOVELTY DETECTION IN THE AUDITORY SYSTEM OF THE BARN OWL

**Authors:** A. Reches, Y. Gutfreund

**Affiliations:** Technion–Israel Institute of Technology, Haifa 32000, Israel

The behavioral response to a stimulus is largely dependent on its context. Novelty detection is an example of a vital context-dependent task performed by the brain. Here we attempt to characterize a neuro-correlate of novelty detection, stimulus-specific adaptation (SSA) in the midbrain and forebrain sound localization pathways of the barn owl. Two paradigms were used to characterize SSA. One is the oddball paradigm, based on presenting probabilistic stimuli in which two differing stimuli are presented with different probabilities: rare and frequent. The second is a novel paradigm of repetitive stimulations in a constant order, creating the equivalent effect of rare and frequent stimuli. We find that responses to rare stimuli are indeed significantly stronger (SSA effect) both in the midbrain and forebrain. However, in the midbrain we report a distinction between the inferior colliculus (IC) and the optic tectum (OT). Similar to the forebrain, the OT showed an SSA effect both to changes in frequency and in interaural time difference (ITD), whereas in the IC SSA effect emerged only in frequency and not in ITD. Interestingly, we find that there is a corresponding SSA effect in response latency in the forebrain and OT, but not in the IC. In addition we report SSA effects for sound intensities which can not be explained by synaptic depression. SSA effects in the intensity domain were absent in IC but clear in the OT. Our results demonstrate that novelty detection is an invariant property of the localization pathways of the barn owl. Different sound properties with diverse neural representations show similar SSA effects, highlighting the importance of novelty in the internal representation of sensory information. The absence of SSA effects in the IC leads us to suggest that the forebrain circuitry is engaged in novelty detection and that novelty signals are conveyed from the forebrain to the midbrain to control behavioral responses in a context dependent manner.

# Abstract: NOVEL CONJUGATES OF ANTIDEPRESSANTS AND GABA POSSESS IMPROVED ANTINOCICEPTIVE ACTIVITY

**Authors:** A. Rephaeli, I. Gil-Ad, I. Terasenko, Y. Geffen, K. Savitsky, A. Aharoni, A. Weizman, A. A. Nudelman

**Affiliations:** ^1^Laboratory of Pharmacology and Experimental Oncology FMRC Beilinson Medical Center, Tel Aviv University, Israel; ^2^Laboratory of Biological Psychiatry, FMRC Beilinson Medical Center, Tel Aviv University, Israel; ^3^BioLineRx Ltd, Jerusalem, Israel; ^4^Department of Chemistry, Bar-Ilan University, Ramat Gan, Israel

Previously we have shown that conjugation of antischizophrenic drugs to gama-amino butyric acid (GABA) resulted in enhanced therapeutic activity and in a reduction of adverse side effects (Geffen et al). In the present study we tested amides of the antidepressant nortriptyline (BL1021) or fluoxetine (BL1024) and GABA in three animal models of pain. In the hot plate model (52 ± 0.2°C) dose response plots demonstrated significantly longer latency of nociceptive reaction to heat in Balb/c mice treated po with the nortriptyline-GABA conjugate BL1021 compared to an equimolar dose of nortriptyline. Similar results were obtained with the fluoxetine-GABA conjugate BL1024. In addition, these conjugates showed more rapid onset and longer duration of their protective effects against pain as compared to the parent compounds. Intraplantar injection of formalin to the paw of Balb/c mice resulted in typical biphasic flinching behavior of the injected paw. BL1021 and Bl1024 significantly reduced the early neurogenic response and the late inflammatory peripheral response compared to equimolar doses of the parent drugs nortriptyline or fluoxetine. Intraplantar injection of carrageenan to the paws of Wistar rats was used to assess the anti-inflammatory effects of BL1021 and BL1024, which were found to be significantly better in inhibiting the edema than nortriptyline or fluoxetine. The significantly better efficacy of amides BL1024 and BL1021, compared to their parent compounds towards both the central perception and the peripheral phase of pain, makes them promising candidates for the treatment of acute and chronic pain conditions. The compounds are currently developed by BioLineRX, the licensors of BL1021 and BL1024.

# Abstract: HUMAN EMBRYONIC STEM CELLS PRODUCE NERVE MYELINATING CELLS

**Authors:** M. Revel, J. Chebath, M. Izrael, P. Zhang, J. Itskovitz-Eldor

**Affiliations:** ^1^Department of Molecular Genetics, Weizmann Institute of Science, Rehovot 76100, Israel; ^2^Department of Obstetrics and Gynecology, Rambam Medical Center, Haifa, Israel

Human embryonic stem (huES) cells are a potential largescale source of neural cells that could be used for transplantation to regenerate neural functions affected by disease or injury. We have producedmature oligodendrocytes fromhuES cells, by applying conditions that induce lineage specific transcription factors Olig1/2, as well as Nkx2.2 and Sox10 which are needed for maturation. The conditions for huES cell differentiation included retinoic acid treatment followed by addition of noggin, an antagonist of bone morphogenetic proteins (BMPs). We found that retinoic acid induces BMPs in huES cells and that noggin was essential to obtain the efficient formation of highly branched mature oligodendrocytes producing myelin basic protein (MBP). Starting from huES cells, we derived highly enriched populations of oligodendrocyte precursors that can be expanded and passaged repeatedly, and subsequently differentiated into mature cells. Transplantation of such precursors showed that pretreatment by noggin markedly stimulates their capacity to myelinate in the brain of MBP-deficient shiverer mice, in organotypic cultures, and in living animals. Arrays of numerous long MBP+ fibers were generated over extended areas in the brain, with evidence of cell migration after transplantation and with formation of compactmyelin sheaths. The possibility to regenerate myelin around axons in the CNS may have therapeutical applications in congenital myelin deficiencies (leukodystrophies), in post-inflammatory demyelinating diseases, and in spinal cord injuries.

# Abstract: GREEN TEA POLYPHENOL EPIGALLOCATECHIN GALLATE REGULATES THE EXPRESSION OF THE IRON-RESPONSIVE PROTEINS APP AND TRANSFERRIN RECEPTOR REDUCES THE ALZHEIMER'S DISEASE-RELATED BETA AMYLOID PEPTIDE

**Authors:** L. Reznichenko, T. Amit, H. Zheng, Y. Avramovich-Tirosh, O. Weinreb, M. B. H. Youdim, S. A. Mandel

**Affiliations:** ^1^The Eve Topf and US NPF and Department of Pharmacology, Faculty of Medicine, Technion–Isreal Institute of Technology , Israel; ^2^Department of Organic Chemistry and Neurobiology, TheWeizmann Institute of Science, Rehovot 76100, Israel

Brain iron dysregulation, resulting in reactive oxygen species initiated oxidative stress, and its association with amyloid precursor protein (APP) regulation and plaque formation have been implicated in Alzheimer's disease (AD) pathology, and thus, iron chelation could be a rational therapeutic strategy for the treatment of AD. The objective of this study was to analyze the effect of the major green tea polyphenol (-)-epigallocatechin-3-gallate (EGCG), which has been shown to possess potent iron chelating properties and radical scavenging activity, on the expression of iron responsive APP and the iron metabolism-related protein transferrin receptor (TfR) at the gene and protein levels. EGCG exhibited potent iron chelating activity comparable to that of the prototype iron chelator desferrioxamine (DFO) and dose dependently (1–10 microM) increased TfR protein and mRNA levels in human SH-SY5Y neuroblastoma cells. Both the immature and full-length cellular holo-APP were significantly reduced by EGCG, as shown by two-dimensional gel electrophoresis, without altering APP mRNA levels, suggesting a posttranscriptional action. Indeed, EGCG suppressed the translation of a luciferase reporter gene fused to the APP-5'-mRNA untranslated region, encompassing the APP iron-responsive element. The finding that Fe2SO4 reversed EGCG action on APP and TfR proteins reinforces the likelihood that these effects are mediated through modulation of the intracellular iron pool. Also, EGCG reduced toxic betaamyloid peptides generation in CHO cells over-expressing the APP “Swedish” mutation. Thus, the natural nontoxic brain-permeable EGCG may provide a potential therapeutic approach for AD and other iron-associated disorders.

# Abstract: FUNCTIONAL IMAGING OF LIMBIC SYSTEM ACTIVITY DURING TWO-WAY ACTIVE AVOIDANCE TRAINING IN ADOLESCENT RATS

**Authors:** A. Riedel, J. Bock, M. Gruss, K. Braun

**Affiliations:** Department of Zoology/Developmental Neurobiology, Otto von Guericke University, Magdeburg, Germany

Learning and memory formation are complex events mirrored by an intricate activation of a variety of limbic regions. Using 2-fluorodesoxyglucose (2FDG) autoradiography, we tested whether acquisition and retrieval in a twoway- active avoidance paradigm are accompanied by changes of metabolic activity patterns in specific brain regions which are involved in this task. Adolescent female rats (6 weeks old), were assigned to four experimental groups: (i) retrieval group: animals which received training in the shuttle box during 5 consecutive days, 50 trials/day, each consisting of a 2.4 kHz tone (CS) and an electric footshock (0.6mA; UCS); and (ii) acquisition group: one day shuttle box training. The respective control groups were exposed to the shuttle box without receiving training with CS and UCS on (iii) five days or (iv) one day, respectively. The animals were injected with 2-FDG (IP; 18microC/100 g) prior to the 5th (test for retrieval) or the 1st (test for acquisition) session. In general, rats of all groups displayed high metabolic activity in the hippocampal formation, and in the subicular complex, whereas CA regions, dentate gyrus, and entorhinal cortex showedmoderatemetabolic activity. In the amygdaloid complex, only the basolateral nucleus displayed a circumscribed prominent activation. In addition, orbitofrontal, cingulate and retrosplenial cortices as well as several neocortical areas, specific and unspecific thalamic nuclei, and caudateputamen showed high 2-FDG uptake. Quantitative analysis revealed reduced 2-FDG uptake in the basolateral amygdala, the anterior and posterior cingulate, as well as retrosplenial cortices in both groups of trained animals (acquisition and retrieval groups) compared to the respective controls. Comparison between acquisition and retrieval groups as well as of additional control groups is under current investigation.

*Supported by grants from the state of Saxony-Anhalt and from GIF.*

# Abstract: CHARACTERIZATION OF THE SIGNALING PROPERTIES OF THE NOVEL ENDOGENOUS LIPID N-PALMITOYL GLYCINE SUGGESTS A ROLE IN NOCICEPTION

**Authors:** N. Rimmerman, H. B. Bradshaw, J. C. Chen, D. K. O`Dell, J. M. Walker

**Affiliations:** ^1^Gill Center for Biomolecular Science, Indiana University, IN, USA; ^2^Department of Psychological and Brain Sciences, Indiana University, IN, USA

Here we investigated the distribution and signaling properties of five novel endogenous putative signaling lipids (Bradshaw et al, 2005; ICRS abstracts online, 120): Npalmitoyl glycine (PalGly), N-stearoyl glycine (StrGly), Noleoyl glycine (OlGly), N-linoleoyl glycine (LinGly), and Ndocosahexaenoyl glycine (DHGly). Their levels were determined by LC/MS/MS in rat CNS, visceral organs, skin, and in the F-11 (DRG X Neuroblastoma) cell line. Calcium mobilization in F-11 cells and the effects of PalGly on heatevoked firing of nociceptive neurons were studied. *Results*. Nacyl glycines were found in all tissues with the highest levels in skin and spinal cord. The compounds were also detected in F-11 cell media. An intradermal, submicrogram dose of PalGly inhibited heat-evoked firing of nociceptive neurons in anesthetized rats. Single cell calcium imaging revealed a dose-dependent (EC50 5microM), immediate, and robust intracellular calcium mobilization following application of PalGly. StrGly showed reduced yet significant effects on calcium mobilization;N-palmitoyl alanine that differs fromPal- Gly by one carbon was inactive. The effect of PalGly was not blocked by SR141716A, SR144528, or MK801. Calcium mobilization was dependent on the presence of extracellular calcium and blocked by the transient potential (TRP) channel blocker Ruthenium red (10 microM) but not by the TRPV1 antagonist 5' iodoresiniferatoxin. PalGly-induced calcium mobilization was attenuated by the receptor operated or voltage-gated calcium channel inhibitor SK&F 96365. *Conclusions*. The newly discovered endogenous family of Nacyl glycine compounds is prevalent throughout the body and at least one member, PalGly, induces calcium mobilization in a dorsal root ganglion neuronal cell line and inhibits firing of nociceptive neurons.

*Supported by NIH/NIDA (DA-018224, DA-020402), Gill Center for Biomolecular Science, Indiana University, and the Lilly Foundation Inc.*

# Abstract: MECHANISM OF ACTIVATION OF THE G-PROTEINCOUPLED K CHANNELS: MOLECULAR MOTIONS REVEALED BY OPTICAL AND ELECTRICAL MEANS

**Authors:** I. Riven, S. Iwanir, E. Reuveny

**Affiliations:** Department of Biological Chemistry, Weizmann Institute of Science, Rehovot 76100, Israel

Regulation of cellular excitability is mediated, in part, by the activation of G protein coupled receptors. These receptors, through specific activation of associated G proteins, directly and/or indirectly modulate the activity of ion channels and various transporters. One of the classical examples for such regulation is the activation of the G-protein-coupled potassium channels (GIRK) by G proteins. This activation involves the intimate association of the G-protein with the channels in concert with other intracellular factors to stabilize the channel's open conformation. To understand such interactions it is necessary to develop sensitive means for the detection of intrinsic subtle motions of the channel molecule during activation, and of its associated G protein in vivo. This can be accomplished by using fluorescence resonance energy transfer (FRET) as a molecular ruler for such motions. One of the limitations of such an approach is the fact that it is rather difficult to specifically fluorescently label the cytosolic face of membrane proteins in situ. To overcome this limitation, genetically encoded labeling is used in conjunction with membrane-restricted FRET measurements. An example will be given using the GIRK channel as a model for a G protein effector, its intrinsic conformational rearrangements and its mode of association with G proteins during resting and activated states will be shown.

# Abstract: MOLECULAR LAYER INHIBITION SHAPES THE TIME COURSE BUT NOT THE SPATIAL ORGANIZATION OF MOSSY FIBER RESPONSES IN THE CEREBELLAR CORTEX

**Authors:** D. Rokni, Y. Yarom

**Affiliations:** Department of Neurobiology, and Interdisciplinary Center for Neural Computation, The Hebrew University, Jerusalem 91010, Israel

It was suggested that the role of the inhibition exerted by the molecular layer interneurons (MLI) in the cerebellar cortex is to limit the time window for synaptic integration and to ensure a precisely timed spike output of Purkinje cells (PCs) (Mittmann et al, 2005). Alternatively, Bower suggested that the absence of mossy fiber (MF) evoked beams of activity is due to the fast activation of the MLIs (Bower, 2002). Both suggested functions, however, can only apply to very brief inputs because of presynaptic inhibition that the MLIs exerts on each other (Cohen and Yarom, 2000; Mann-Metzer and Yarom, 2002). We used the voltage-sensitive dye RH-414 to describe the spatio-temporal responses of the cerebellar cortex to the climbing fiber (CF) and MF inputs and the effect of the MLI inhibition on these responses in the isolated cerebellum of a Guinea pig. Surface stimulation elicited a propagating beam of activity along the Pf axis followed by lateral inhibition. WM stimulation elicited a variety of responses. We devised a method to classify these responses as Cf or Mf responses according to spatio-temporal characteristics. In short, principal component analysis of the *x*, *y* coordinates of responding diodes was used to quantify the elongation and orientation of the response. Parasagitally oriented responses with long durations were attributed to Cf activation, and radial responses with short durations were attributed to Mf activation. Beams of activity, propagating along the Pf axis, were never encountered in response toWM stimulation. This was not due to the MLI inhibition as application of GABAzine prolonged the response without affecting its spatial distribution. Similarly, prolonged responses were observed when Mf activation was preceded by inhibition evoked by surface stimulation. Conversely, Cf responses were not affected by the preceding inhibition. Thus inhibition in the cerebellar cortex might serve to increase responsiveness to prolonged Mf input.

# Abstract: TEMPORAL SUMMATION IN THE EARLY VISUAL SYSTEM

**Authors:** A. Rosen, M. Belkin, U. Polat

**Affiliations:** Goldschleger Eye Research Institute, Tel Aviv University, Tel Hashomer, Israel

Temporal summation is a fundamental feature that underlies information processing of neurons, but it remains an unresolved aspect of the visual system. Previously we reported that contrast threshold (CT) decreases with increasing presentation time of a target up to at least 480 ms. This result suggests a relatively long integration time, which may be considered as a long persistence time of neurons in the primary visual areas. However, a short integration time (approx 20 ms–40ms) of neurons was found when a summation of 2 brief pulses of flashed light was measured. We sought to test two-pulse summation at the cortical level using smalloriented Gabor patches (GP, 12 cpd, approx 0.25° diameter). CT for the target GP, using a two-alternative forcedchoice paradigm and staircase method, was measured under conditions of a double pulse of identical stimuli (GPs), and a pedestal (set to 0.5 or 2 times the subjects CT) and target. The pedestal was presented in the two intervals before the target at SOAs of 0ms, 30 ms, 45ms, 60 ms, 75ms, and 90 ms (ISI = 0 (same onset), 0 ms, 15ms, 30 ms, 45ms, and 60 ms, resp). The target and the pedestal had 30 ms durations each whereas the target was presented only in one interval. We found that there was a significant decrease in CT with SOAs of 0ms, 30 ms, and 45 ms. Thus, the summation of the pedestal with the target decreased when the gap increased between the presentations, culminating in no effect after an ISI (gap) of 30ms. To control for the retinal effect, we repeated the experiment with orthogonal orientations of the pedestal and target. The results show no effect of summation and the CT was constant over all SOAs. Since the effect of summation is orientation-specific, the results indicate that the pulse summation has a cortical level. Thus, the short duration of the pulse summation, in contrast to the much longer effect of integration time, suggests a different time course of temporal summation for sustained and transient processing.

# Abstract: POSSIBLE MECHANISM FOR FUNCTIONAL PLASTICITY IN THE ADULT BRAIN: A PROSPECTIVE CASE STUDY OF LANGUAGE fMRI

**Authors:** K. Rosenberg, R. Liebling, G. Avidan, T. Siman-Tov, F. Andelman, Z. Ram, T. Hendler

**Affiliations:** ^1^Tel Aviv Sourasky Medical Center, Israel; ^2^Tel Aviv University, Tel Aviv 69978, Israel; ^3^Ben Gurion University, Beer Sheva 84105, Israel

Plasticity in the adult brain is critical for functional recovery following a localized lesion. One possible mechanism for such plasticity is the recruitment of the unaffected hemisphere. Studies showed that even extensive lesion of the left hemisphere, acquired either prenatally or in early childhood, can result in nearly normal development of language. Functional imaging in such cases at adulthood demonstrated normal distribution of language activation in the right hemisphere. However, it is yet unclear if lesions in the language dominant hemisphere acquired in adulthood can also result in a functional recovery. The only way to test this assertion is by performing consecutive functional brain imaging following an acquired lesion in adults. Here we present a case study of patient JT, a 28 years old, right handed male who was diagnosed with left fronto-temporal low-grade glioma (lesion size: 44∗65∗48 mm). As part of a presurgical assessment JT underwent an fMRI-based mapping of his language related areas. The fMRI exam showed left hemispheric dominance, with lateralization index (LI) = 1 in Broca's area. Although frontal language activations were in close vicinity to the lesion, there was no deficit in his language function. Surgical intervention was not applied at that time. Two years later there was a 10% increase in the tumor volume yet with no deficits in language function. Surgical option was reconsidered and fMRI exam was performed again. In comparison to the first fMRI exam there was an increased activation for language in the right hemisphere resulting in altered LI in the Broca's area to −0.93. Interestingly, there was no such change in posterior language areas. fMRI exams following surgery showed the same pattern of right hemispheric dominance with almost no activations in the left hemisphere (LI = −1). This unique case study supports the idea that interhemispheric functional transfer may underlie potential functional plasticity in the adult human brain.

# Abstract: IMAGING SODIUM CHANGES IN THE AXONS OF CNS NEURONS

**Authors:** W. N. Ross

**Affiliations:** Department of Physiology, New York Medical College, Valhalla, NY, USA

Sodium concentration changes in neurons can be examined using the selective indicator SBFI similarly to the way fura-2 and other indicators have been used to examine calcium changes in neurons. Using this approach we looked at sodium changes in cerebellar Purkinje cells and both hippocampal and neocortical pyramidal neurons in slices from young rats. We could see clear increases from both long subthreshold pulses (activating the persistent current) and action potentials (activating the transient current) in these cells. In neocortical pyramidal neurons the changes from both currents were much larger in axons compared to the soma and dendrites. Since the axons and basal dendrites were of comparable diameter this implies that the channel density ismuch higher in the axon than in the dendrites and is probably higher than in the soma. In both Purkinje cells and pyramidal cells distinct signals could be detected from both the initial segment and first node of Ranvier. Transients recovered rapidly after the end of stimulation but were insensitive to ouabain. Simulations demonstrate that the rapid recovery time course is due to the diffusion of sodium away from localized sites of entry. Differences between cell types and the significance of diffusional regulation will be discussed.

*Supported by BSF and NIH.*

# Abstract: MAGNETIC STIMULATION OF ONE-DIMENSIONAL NEURAL CULTURES IN VITRO

**Authors:** A. Rotem, E. Moses

**Affiliations:** Weizmann Institute of Science, Rehovot 76100, Israel

Magnetic stimulation of nerves is attracting increased attention recently, as it has been found to be useful in therapy of neural disorders in humans. In an effort to uncover the mechanisms of magnetic stimulation we apply magnetic stimulation on ex vivo neuronal preparations. Preliminary work on sciatic nerves demonstrated the dependence of magnetic stimulation on neuronal morphology and in particular the importance of curvature of axonal bundles. A more recent work demonstrates the first magnetically evoked activity in cultures. We will show how magnetic pulses initiate neuronal activity in 1D patterned cultures of hippocampal neurons, and explore the effects of properties such as geometry of the system, axonal morphology, pharmacology, and neuronal density on the threshold of magnetic stimulation. We will also describe our findings regarding the effect of repetitive magnetic stimulation on neuronal activity (see [1]).

## References

[1] Rotem A, Moses E (2006). Magnetic stimulation of curved nerves. *IEEE Transactions on Biomedical Engineering*.

# Abstract: CHARACTERIZING NETWORK DYNAMICS AND FUNCTIONAL ARCHITECTURE OF THE MOUSE AUDITORY CORTEX USING IN VIVO 2-PHOTON CALCIUM IMAGING

**Authors:** G. Rothschild, A. Mizrahi

**Affiliations:** ^1^Interdisciplinary Center for Neural Computation, The Hebrew University, Jerusalem 91010, Israel; ^2^Department of Neurobiology, The Hebrew University, Jerusalem 91010, Israel

The auditory cortex (AC) of the house mouse has thus far been mainly studied using electrophysiological recordings. These studies have used best-frequency maps obtained using local field potential recordings to draw a gross outline of the functional architecture of the AC. By exhausting these best frequency maps throughout the AC, the different functional subregions of the AC could be identified (A1, AAF, etc). However, due to technical limitations, the functional micro-architecture of the AC remains unknown. Recently, 2-photon Laser scanning microscopy (2PLSM) has proven useful in revealing the micro-architecture of the neocortex with unprecedented single cell resolution (Ohki et al, Nature, 2005). The aim of the current study has therefore been to reveal the microarchitecture of the AC using 2PLSM of calcium signals, and using the house mouse as a model. To this end, we have combined classical electrophysiology with 2PLSM. First, using extracellular recordings, we obtained cortical responses to auditory stimuli in order to identify the AC. Then, we performed bulk loading of a calcium sensitive dye into the AC, and imaged the network activity of tens of cells simultaneously in response to auditory stimuli at single-cell resolution. By analyzing the change in fluorescence of each cell in response to broad band noise and to pure tones, we could determine its response properties, and thus reconstruct the functional microarchitecture of the network. This experimental strategy will allow us not only to reveal the functional microarchitecture of the AC but also to describe its network dynamics, and eventually set the stage to study its plasticity.

# Abstract: GALECTIN-3/MAC-2, K-RAS, AND PI3K REGULATE MYELIN PHAGOCYTOSIS IN MICROGLIA

**Authors:** S. Rotshenker, F. Reichert, G. Elad-Sfadia, R. Haklai, Y. Kloog

**Affiliations:** ^1^Faculty of Medicine, The Hebrew University, Jerusalem 91120, Israel; ^2^Faculty of Life Sciences, Tel Aviv University, Tel Aviv 69978, Israel

The removal of degenerated myelin is essential for repair following traumatic injury to axons duringWallerian degeneration, and for minimizing damage to intact axons and myelin in multiple sclerosis. Microglia and macrophages remove degenerated myelin by phagocytosis. We focus presently on complement receptor-3 (CR3/MAC-1) and scavenger receptor-AI/II (SRAI/II) mediated myelin phagocytosis in microglia. Paradoxically, these receptors are expressed in microglia after injury to CNS axons but myelin is not phagocytosed, suggesting that phagocytosis is subject to modulation between efficient and inefficient states. Furthermore, Galectin-3/MAC-2 is expressed in microglia that phagocytose but not in microglia that do not phagocytose, thus raising the possibility that Galectin-3/MAC-2 is instrumental in regulating phagocytosis. We presently test for the regulatory roles of Galectin-3/MAC-2, K-RAS, and phosphatidylinositol 3-kinase (PI3K) in myelin phagocytosis. Myelin phagocytosis was studied in cultured mice microglia. S-trans,transfarnesylthiosalicylic (FTS), which inhibits Galectin-3/MAC- 2 dependent activation of PI3K through K-RAS-GTP, the active form of K-RAS, inhibited myelin phagocytosis. K-RASGTP levels, PI3K activity, and phospho-Akt levels increased during normal phagocytosis and decreased during phagocytosis in the presence of FTS. Galectin-3/MAC-2, which binds and stabilizes K-RAS-GTP that then activates PI3K, coimmunoprecipitated with RAS, and levels of the coimmunoprecipitate, increased dramatically during phagocytosis. These observations suggest, altogether, a role for Galectin-3/MAC- 2 dependent activation of PI3K through K-RAS-GTP in myelin phagocytosis mediated by CR3/MAC-1 and SRAI/II. An explanation is offered, thereby, for efficient phagocytosis by microglia that express CR3/MAC-1 and SRAI/II along with Gal-3/MAC-2, and for deficient phagocytosis by microglia that express CR3/MAC-1 and SRAI/II without Galectin-3/MAC-2.

# Abstract: GALPHA-I3 PRIMES THE G PROTEIN-ACTIVATED K+ CHANNELS FOR ACTIVATION BY COEXPRESSED GBETAGAMMA IN INTACT XENOPUS OOCYTES

**Authors:** M. Rubinstein, S. Peleg, S. Berlin, N. Dascal

**Affiliations:** Department of Physiology and Pharmacology, Sackler School of Medicine, Tel Aviv University, Tel Aviv 69978, Isreal

G protein activated K+ channels (GIRK) mediate postsynaptic inhibitory effects of neurotransmitters in the atrium and in the brain by coupling to G-protein-coupled receptors (GPCRs). In neurotransmitter-dependent GIRK signaling, Gbetagamma is released from the heterotrimeric Galpha-betagamma complex upon GPCR activation, activating the channel and attenuating its rectification. Today it becomes clear that Galpha is more than a mere Gbetagamma donor. We have proposed that Galpha-i-GDP regulates GIRK gating, keeping its basal activity low but priming (predisposing) the channel for activation by agonist in intact cells, and by Gbetagamma in excised patches. Here we have further investigated GIRK priming by Galpha-i3 using a paradigm in which the channel was activated by coexpression of Gbetagamma, and the currents were measured in intact xenopus oocytes using the two-electrode voltage clamp. This method enables to bypass the GPCR activation, and to examine the regulation of GIRK rectification by Galpha, in intact cells. Using this method, we further characterize the priming phenomenon. We tested and excluded the possibility that our estimates of priming are affected by artifacts caused by series resistance or large K+ fluxes. We demonstrate that Galpha-i3 reduces the basal channel activity similarly to a membrane-attached Gbetagamma scavenger protein, m-phosducin. However, Galpha-i3 allows robust channel activation by coexpressed Gbetagamma, in sharp contrast to m-phosducin which causes a substantial reduction in the total Gbetagamma-induced current. Furthermore, Galpha-i3 also does not impair the Gbetagamma-dependent attenuation of the channel rectification, in contrast to m-phosducin which prevents this Gbetagamma-induced modulation. The G Galpha-i3-induced enhancement of direct activation of GIRK by Gbetagamma, demonstrated here for the first time in intact cells, strongly supports the hypothesis that Galpha-i regulates GIRK gating under physiological conditions.

*This work was supported by Segol Fellowship from the Adams Brain Research Center to the first author.*

# Abstract: LEARNING-INDUCED ENHANCEMENT OF THE SIZE OF UNITARY SYNAPTIC EVENTS

**Authors:** D. Saar, D. Keren, E. Barkai

**Affiliations:** ^1^Departments of Biology and Neurobiology, Faculty of Sciences, University of Haifa, Haifa 31905, Israel; ^2^Department of Computer Sciences, Faculty of Social Sciences, University of Haifa, Haifa 31905, Israel

Rats were trained in four-arm maze to discriminate between odours in pairs. As previously shown, rats require 6–8 consecutive training days to learn to distinguish between a pair of odours, but to learn a second pair of odors only requires 1-2 training days (rule learning). Piriformcortex brain slices were prepared 4 days after rule-learning, and wholecell voltage-clamp recordings were obtained from layer II pyramidal neurons at 300 C. With Vm held at −80 mV, the averaged amplitude of the minimal (quantal) spontaneous events was significantly larger in neurons from trained rats (8.11 + 3.2 pA, *n* = 13) compared to neurons from pseudotrained rats (6.01 + 0.91 pA, *n* = 12, *P* < .05). Thus, the single quantum increases after rule-learning. Accordingly, The averaged amplitude of the spontaneous events was significantly larger in neurons form trained rats (11.96 + 5.5 pA, *n* = 13) compared to pseudotrained rats (8.17 + 1.69 pA, *n* = 12, *P* < .05). In contrast, the frequency of spontaneous events was similar in both groups (96 + 60 events per minute in neurons from pseudotrained rats, compared with 76 + 65 in neurons from trained rats), indicating that the probability of release is not modified after learning. Our data support the notion that olfactory learning is accompanied by a longlasting post-synaptic modifications of glutamatergic transmission onto layer II piriform cortex pyramidal neurons.

*Supported by the National Institute for Psychobiology in Israel and the ISF.*

# Abstract: PRINCIPLES UNDERLYING INFORMATION TRANSDUCTION ALONG ALLOSTERIC COMMUNICATION NETWORKS IN VOLTAGE-ACTIVATED POTASSIUM CHANNELS

**Authors:** Y. Sadovski, O. Yifrach

**Affiliations:** Department of Life Sciences, Ben Gurion University of the Negev, Beer Sheva 84105, Israel

Efficient energy propagation through a protein is an important property underlying many biological phenomena. The flow of information between distal elements of a protein may rely on allosteric communication networks along the protein's tertiary or quaternary structure. To unravel the underlying features of energy parsing along allosteric pathways recently detected in voltage-gated K+ channels, highorder thermodynamic coupling analysis was performed. We report that such allosteric trajectories are functionally conserved and delineated by sharply defined boundaries. Moreover, allosteric trajectories assume a hierarchical organization whereby increasingly stronger layers of cooperative residue interactions act to ensure efficient and cooperative long-range coupling between distal channel regions. This long-range communication is brought about by a coupling of local and global conformational changes suggesting that the allosteric trajectory also corresponds to a pathway of physical deformation. These trajectory features may be a general property of allosterically regulated proteins and might explain how the lower activation and upper inactivation pore gates of voltage-activated K+ channels communicate.

# Abstract: DYNAMIC GAIN CONTROL IS AN INTRINSIC PROPERTY OF FLYMOTION DETECTORS

**Authors:** M. N. Safran, V. L. Flanagin, A. Borst, H. Sompolinsky

**Affiliations:** ^1^Institute of Life Sciences, The Hebrew University, Jerusalem 91904, Israel; ^2^Center for Neural Computation, The Hebrew University, Jerusalem 91904, Israel; ^3^Max Planck Institute of Neurobiology, 82152 Martinsried, Germany; ^4^Racah Institute of Physics, The Hebrew University, Jerusalem 91904, Israel

Adaptation is an important property of many sensory systems. In this work we investigate the mechanisms underlying the adaptive behavior of a motion-sensitive neuron of the fly visual system, H1. This neuron has been shown to rapidly adapt its velocity response gain to the dynamic range of stimulus velocity fluctuations. We ask two questions. (1) Which parameters of the motion detection systemin the fly adapt to stimulus statistics? (2) Does the observed velocity gain control result from adaptation of the parameters of the motion detection system, or is it an automatic result of the nonlinearity of the system, as previously suggested (Borst et al, 2005)? To address these questions, we model H1 as an array of Reichardtmotion detectors, consisting of a high-pass filter (HPF), a low-pass filter (LPF), amultiplier, and a subtraction stage, followed by a static nonlinearity. The parameters of the model are fit to spike trains recorded from H1 while flies were stimulated by amoving grating with a low-pass filtered white noise velocity profile with various velocity fluctuation amplitudes. In order to determine which, if any, of the parameters of the system change with the stimulus statistics, we calculate the optimal parameters for each stimulus condition. We find that the shape of the static nonlinearity is weakly dependent on stimulus amplitude. The strongest dependence is exhibited by the HPF time constant, which shortens considerably with increasing velocity fluctuations. However, adaptation of the velocity response gain to the amplitude of velocity fluctuations turns out to be largely independent of these parameter changes. This is shown by comparing the above model to a motion detector with fixed optimal parameters, which exhibits similar gain control. We conclude that the dynamic gain control exhibited by H1 arises from the intrinsic properties of the underlying motion detector, independent of parameter change.

# Abstract: MODELING A L4-TO-L2/3MODULE OF A SINGLE COLUMN IN RAT NEOCORTEX: INTERWEAVING IN VITRO AND IN VIVO EXPERIMENTAL OBSERVATIONS

**Authors:** L. Sarid, D. Feldmeyer, B. Sakmann, I. Segev

**Affiliations:** ^1^Department of Neurobiology, Institute of Life Sciences, Jerusalem 91904, Israel; ^2^Interdisciplinary Center for Neural Computation, The Hebrew University, Jerusalem 91904, Israel; ^3^Department of Cell Physiology, Max Planck Institute for Medical Research, 69120 Heidelberg, Germany; ^4^Cellular Neurobiology Group, Institute of Medicine, Research Centre Julich, 52425 Julich, Germany

We report here a first step in constructing an in silico model of a neocortical column, focusing on the synaptic connection between layer 4 (L4) spiny neurons and layer 2/3 (L2/3) pyramidal cells in rat barrel cortex. It is based firstly on a detailed morphological and functional characterization of synaptically-connected pairs of L4-L2/3 neurons from in vitro recordings and secondly on in vivo recordings of voltage responses of L2/3 pyramids to current steps as well as on their response evoked by a whisker deflection. In vitro data, combined with a detailed compartmental model of L2/3 cells, enable us to extract their specific passive properties and spatial distribution of L4-L2/3 synaptic contacts. The specific membrane resistivity and capacitance of L2/3 cells are around 16,000 ohmcm2 and 0.8microF/cm2, respectively, and the peak conductance per L4 synaptic contact is 0.1nS–0.48nS for AMPA receptors and around 0.2nS for NMDA—receptors. The in vivo voltage response for current steps were then used to calibrate the L2/3 compartmental model for in vivo conditions in the DOWN state. Consequently, the effect of a single whisker deflection on the voltage response of an L2/3 pyramidal cell was modeled by activating a population of, on average, 350 L4 axons (1,575 synaptic contacts) converging onto the modeled L2/3 cell. Based on conductance values per synaptic contact, their spatial distribution on L2/3 dendrites and the in vivo firing probability of L4 spiny neurons, the model predicts that the feed-forward L4-L2/3 connection, on its own assuming no correlation between the firing of the presynaptic L4 cells, cannot fire the L2/3 pyramidal cell. Possible elaboration of the model will be presented.

*Supported by a grant from the HFSP and from the Safra Foundation.*

# Abstract: AGE-RELATED COGNITIVE DECLINE CORRELATES WITH REGIONAL BRAIN CHANGES MEASURED BY Q-SPACE MRI

**Authors:** E. Sasson, G. M. Dolinger, Y. Assaf

**Affiliations:** ^1^Department of Neurobiochemistry, Faculty of Life Sciences, Tel Aviv University, Ramat Aviv, Tel Aviv 69978, Israel; ^2^L-E-G Institute of Functional Brain Imaging, Tel Aviv University and Tel Aviv Sourasky Medical Center, Israel; ^3^Department of Clinical Science, NeuroTrax Corporation, New York, NY, USA

Aging is a complex heterogeneous process accompanied by a cognitive decline. Although defined as a natural condition, many pathological neurodegenerative processes are involved in aging, manifested by reduced memory, executive function, motor abilities, and processing speed. The main hypothesis of this research is that age-related cognitive decline is a complex, not specific and multiregional process without repeated pattern across subjects. In the present research we used Q space imaging (QSI), which is a noninvasive, highly sensitive MRI method for detection of brain changes. QSI was performed in brains of 50 subjects, age 25–82 y. Subjects also underwent a series of neuropsychological tests outside the scanner (including memory performance, executive function, motor abilities, visual-spatial, and verbal function). The subjects' test performance as well as subject's age served as covariate correlation inputs for voxel-based morphometry (VBM) of MRI scans. Region of interest (ROI) analysis was performed based on the VBM results and the correlation coefficient was extracted for each test in each ROI. The results showed that age is highly correlated with QSI indices in all ROIs, while the correlation of cognitive performance with QSI indices in each ROI was task specific. For example, in the right thalamus, the correlation coefficient with memory was *r* = 0.46, while in other tasks (motor, visual spatial, and verbal tasks) a correlation coefficient smaller than 0.35 (*r* < 0.35) was found. An exception was the stroop interference task, which showed a high correlation in all ROIs, implying that it is a nonspecific multiregional task. In conclusion, although brain changes with age are multiregional, the cognitive abilities of subjects show region specific brain changes, which allow a more focused observation of the heterogeneous aging process.

# Abstract: LEARNING AND THE PRESYNAPSE: LACK OF THE PRESYNAPTIC PROTEIN SAP47 IMPAIRS OLFACTORY ASSOCIATIVE LEARNING IN LARVAL DROSOPHILA

**Authors:** T. Saumweber, B. Michels, N. Funk, E. Buchner, B. Gerber

**Affiliations:** University of Wurzburg, Germany

The SAP47 protein was discovered in Drosophila by an antibody screen for presynaptic proteins (Reichmuth et al 1995, Brain Res Mol Brain Res; Funk et al 2004, BMC Neurosci). It is a member to a novel, phylogenetically conserved, gene family. Here we take the first step to understand its function. In Drosophila, the SAP47 protein is encoded by one single gene for which a deletion mutant (Sap47156) is available, suffering a 2.1 kb deletion in the regulatory region and the first exon of the gene. We outcrossed this mutant strain to wild-type (WT) for nine generations to achieve isogenic background. These strains, after describing the expression pattern of SAP47 in wild-type larvae and showing the absence of protein in Sap47156 mutants, were tested in an associative learning task in larval fruit flies. For this task, larvae are trained to associate an odour with a food reward. We report that Sap47156 mutants show reduced learning when compared to wild-type (WT). Sensory ability with respect to detection of the to-be-associated stimuli and motor performance, however, was normal in these mutants. This underscores that presynaptic function in invertebrates is critical for learning. We now pursue an RNAi approach to test where in the brain SAP47 function is necessary for learning.

# Abstract: TEMPORAL INACTIVATION, BUT NOT LESION, OF THE DORSAL STRIATUM REDUCES COMPULSIVE BEHAVIOR IN A RAT MODEL OF OBSESSIVE-COMPULSIVE DISORDER

**Authors:** E. A. Schilman, D. Joel

**Affiliations:** Tel Aviv University, Israel

The orbitofrontal cortex and the striatum have been consistently implicated in the pathophysiology of obsessivecompulsive disorder (OCD). Using a rat model of OCD we have recently found that lesions to the orbitofrontal cortex led to an increase in “compulsive” behavior that was paralleled by an increase in the density of the striatal serotonin transporter. The aim of the present study was to assess the involvement of the dorsal striatum in “compulsive” behavior. We performed either temporal inactivation or excitotoxic lesions of the dorsal striatum in an area we have previously found to be directly innervated by the orbital cortex. We found that temporal inactivation led to a decrease in “compulsive” behavior in the model whereas lesions had no effect. These results are in line with data from OCD patients which implicate the striatum in the pathophysiology of OCD.

*This work has been supported by the National Institute for Psychobiology in Israel, Grant no 2004513.*

# Abstract: DORSAL STRIATUM ACTIVITY DISTINGUISHES LEARNERS FROM NONLEARNERS IN AN INSTRUMENTAL CONDITIONING TASK

**Authors:** T. Schonberg, N. Daw, D. Joel, J. P. O'Doherty

**Affiliations:** ^1^Department of Psychology, Tel Aviv University, Tel Aviv 69978, Israel; ^2^Division of Humanities and Social Sciences and Computation and Neural Systems Program, Caltech, Pasadena, CA, USA; ^3^Gatsby Computational Neuroscience Unit, University College London, London, UK

A recent neuroimaging study found evidence for neural activity in dorsal and ventral striatum that may relate to the actor and critic components of instrumental conditioning respectively (O'Doherty et al, 2004). The aim of the present study was to determine whether instrumental conditioning critically depends on activation of the striatum. We used a task in which approx 50% of normal subjects fail to learn. We hypothesized that regions playing a critical role in instrumental conditioning should be more engaged in those subjects who succeed in learning the task (learners) than in those who fail to learn the task (nonlearners). 30 subjects (1 discarded) were scanned with fMRI while undergoing the task. Subjects' decisions were modeled using a temporal-difference (TD) algorithm to generate prediction error (PE) signals for each subject and then regressed against each subjects' fMRI data. Comparisons were conducted to determine areas showing enhanced correlations with PE signals in learners than nonlearners. 17 subjects met the learning criterion and were categorized as learners; 12 categorized as nonlearners. Consistent with our hypothesis, significant correlations with PE signals were found in both the dorsal and ventral striatum in learners at P < .001. In contrast, only ventral striatum activity was observed in nonlearners, at a lower significance level (P < .05). Furthermore, a direct comparison between the two groups revealed that the dorsal striatum was significantly better correlated with PE signals in learners than in nonlearners. We found that neural activity in dorsal striatum is significantly better correlated with prediction error signals in learners compared to nonlearners. These results are suggestive of a causal relationship between prediction error signals in dorsal striatum activity and learning efficacy. These signals may be generated by afferent dopaminergic inputs into this region.

# Abstract: EFFECT OF EARLY FOOD RESTRICTION ON YOUNG CCK1 SPONTANEOUS KNOCKOUT RATS

**Authors:** M. Schroeder, O. Zagoory-Sharon, V. Gelbar, T. H. Moran, S. Bi, A. Weller

**Affiliations:** ^1^Bar-Ilan University, Ramat Gan, Israel; ^2^Johns Hopkins University School of Medicine, Baltimore, MD 21205, USA

The OLETF rat has been extensively studied as a model of hyperphagia, obesity, and diabetes mellitus. Male and female OLETF rats are about 30% heavier than their LETO controls by 20weeks of age. Previous studies in our lab demonstrated that OLETF pups are heavier already from the first postnatal day (PND1) and were found suckling more frequently than controls during the whole lactating period. The purpose of the present study was to better understand early dietary influences on the later development of obesity. We examined the influence of early, short term and chronic food restriction, starting from the day of weaning, on obesity, fat pads and leptin levels, in 22–90 day-old male and female OLETF rats. Pups were separated from the dam on PND22 and weighed every fifth day. The first group of males and females was fed from weaning to PND90 according to the amount of food consumed by LETO controls. Tissues were collected on PND38, 65, and 90. In the second group, pups were pair fed from weaning until their weight was normalized (approx day 30) and for two further weeks (until around PND45) and were then returned to ad-libitum food access. Tissues were collected on PND90. OLETF males and females under chronic food-restriction showed normalized (to LETO levels) weight, hormonal and fat levels. Permitting free feeding after restriction allowed OLETF females to regain almost all the weight and fat. In contrast, OLETF males showed a slight increase in weight gain, fat mass, and hormone levels, but these remained significantly lower than in freely fed OLETF rats.

*Supported by US-Israel BSF.*

# Abstract: INHIBITION OF NMDA RECEPTOR TYROSINE PHOSPHORYLATION IMPROVES FUNCTIONAL RECOVERY FOLLOWING CLOSED HEAD INJURY IN MICE

**Authors:** J. Schumann, A. Alexandrovich, E. Shohami, R. Yaka

**Affiliations:** Departmant of Pharmacology, School of Pharmacy, Faculty of Medicine, The Hebrew University, Jerusalem 91120, Israel

Traumatic brain injury triggers a massive glutamate efflux, hyperactivation of NMDA receptors (NMDARs) and neuronal cell death. Previously we reported that immediately (15 min) following experimentally induced closed head injury (CHI), the number of activated NMDARs increases in the hippocampus, and decreases in the cortex at the impact site. Here we show that CHI-induced alterations in the number of activated NMDARs correlate with changes in the expression levels of the major NMDARs subunits. In the hippocampus, the expressions of NR1, NR2A, and NR2B subunits were increased while in the cortex at the impact site, we found a decrease in the expressions of NR1 and NR2B but not in NR2A. We demonstrate that the CHI-induced increase in the expression of NMDAR subunits in the hippocampus but not in the cortex is associated with an increase in NR2B tyrosine phosphorylation, and inhibition of NR2B-phosphorylation restored the expressions of these subunits to their normal levels. Finally, a single injection of tyrosine kinase inhibitor, prior to the induction of CHI, resulted in a significant improvement in long-term recovery of motor functions observed in CHI mice. These results provide a novel approach for designing new drug targets to treat human brain injury.

# Abstract: VOLTAGE-GATED ION CHANNELS IN THE SUBSTANTIA-NIGRA DOPMINERGIC NEURONS

**Authors:** D. Segev, A. Korngreen

**Affiliations:** ^1^The Mina and Everard Goodman Faculty of Life Sciences, Bar-Ilan University, Israel; ^2^The Leslie and Susan Gonda (Goldschmied) Multidisiplinary Brain Research Center, Bar-Ilan University, Israel

The Substantia Nigra is part of the Basal Ganglia area of the brain—the motion circuit in the brain. The Substantia Nigra contains two major kinds of neurons—dopaminergic, which are damaged by Parkinson's disease, (∼85%) and GABAergic (∼15%). One of the characteristics of dopaminergic neurons is that, in most of them, the axon's origin is on the dendrite (and not on the soma); sometimes it is located tens of microns from the soma. It has been shown that the action potential is created on the axon's origin, and transferred to the soma through the dendrite. Through electrophysiological measurements, we are interested in finding the basic electrophysiological characteristics of the voltage-gated potassium currents of those cells, and whether there is a relation between the concentrations of different kinds of voltagegated ion channels on the dendrite and their distance from the axon's origin on the dendrite. That kind of information could be valuable in acquiring knowledge on the dopaminergic neurons in the Substantia Nigra, and may help understanding the variety of diseases (like Parkinson's disease and Huntington disease) and psychiatric disorders (schizophrenia, etc) that are related to this area. In addition, our data can help in understanding the process of creating the action potential in the axon's origin and the way that information flows from the axon's origin to the soma through the dendrite.

# Abstract: AFMK, AN ACTIVEMETABOLITE OF MELATONIN, HAS A BENEFICIAL EFFECT ON CLINICAL RECOVERY AND APOPTOTIC SIGNALING PATHWAYS AFTER CLOSED HEAD INJURY

**Authors:** D. Shabashov, A. Alexandrovich, R. Kohen, R. J. Reiter, D. X. Tan, E. Shohami

**Affiliations:** ^1^Department of Pharmacology, The Hebrew University, Jerusalem 91010, Israel; ^2^School of Pharmacy, The Hebrew University, Jerusalem 91010, Israel; ^3^Department of Cellular and Structural Biology, The University of Texas, San Antonio, TX, USA

Closed head injury (CHI) initiates a cascade of massive reactive oxygen species (ROS) production. Disruption of cell oxidative balance leads to activation of inflammatory and apoptotic pathways. The brain tissue is highly susceptible to oxidative stress due to its high metabolic rate, high contents of PUFA and lack of repair and defense mechanisms. Melatonin and its active metabolite N1-acetyl-N2-formyl-5- methoxykynuramine (AFMK) are potent antioxidants, and previous studies have shown their effectiveness in various inflammatory and apoptotic models. Nuclear Factor-kappa-B is a redox-sensitive transcription factor involved in inflammatory processes (TNF-alpha, IL-1beta). Its activation involves phosphorylation of the Inhibitory-kappa-B molecule. Akt-1 phosphorylation has been shown to play a role in neuroprotection via deactivation of pro-apoptotoc molecules (Bad, Bax) and augmentation of antiapoptotic ones (BclXL). This study examined the effect of AFMK on neurological outcome, gene expression, and protein activation of ROSrelated pathways.Three groups of male Sabramicewere used in this study—vehicle, melatonin, and AFMK injected. Mice were subjected to CHI induced by a weight drop device or sham surgery. Motor function was measured 1 hour after injury and on given timepoints. Akt-1 and I-kappa-B levels were measured by Western Blotting. Bad, Bax, BclXL, IL- 1beta. IL-10 and TNF-alpha levels were measured by realtime RT-PCR. AFMK improved motor function after injury and increased Akt-1 phosphorylation. Its effect on I-kappa- B phosphorylation was less pronounced, yet it kept its activation on the basal level (similar to sham group). AFMK also enhanced the expression of BclXL and IL-10 while reducing the expressions of Bad, Bax, IL-10, and TNF-alpha. These finding suggest that AFMK exerts a neuroprotective effect by acting on keymolecules in apoptotic and inflammatory pathways affected by ROS, shifting the balance towards more protective cellular pathways.

# Abstract: NICOTINIC ACETYLCHOLINE RECEPTOR (nAChR) DESENSITIZATION AND ITS RELEVANCE TO PHYSIOLOGICAL AND BEHAVIORAL FUNCTIONS

**Authors:** M. Shachar, H. Cohen, E. Heldman

**Affiliations:** ^1^Department of Clinical Biochemistry, Ben Gurion University of the Negev, Beer Sheva 84105, Israel; ^2^Anxiety and Stress Research Unit, Beer Sheva MHC, Ben Gurion University of the Negev, Beer Sheva 84105, Israel

The nicotinic acetylcholine receptor (nAChR), a member of the ligand-gated ion channel super family, mediates thermoregulation and cognitive functions. Treatment with nicotine may provide beneficial effects for cognitive dysfunctions; however rapid desensitization of the nAChR makes this therapeutically ineffective. Drugs that retard receptor desensitization may prolong the effect of repeated administration. This study assessed the effect of repeated administration of nicotine, with and without nAChR modulators, on emotional (elevated plus-maze (EPM)), cognitive (Morris water maze (MWM)), biochemical (BDNF mRNA expression) and physiological parameters (hypothermia). Rats were injected with nicotine three times a day for three days, with and without nAChR modulators and body temperature was measured continuously at 30 minutes intervals. Behavioral responses were assessed in the MWM and EPM 30 minutes after the first and third injections. Frontal cortex (FC) and hippocampal subregions were isolated. When nicotine was repeatedly injected for three consecutive days, the decrease in body temperature was progressively significantly smaller, indicating desensitization of the nAChR. When nAChR modulators were injected together with nicotine on the second and third days, the progressive attenuation was significantly less marked, and the drop in body temperature was significantly greater after the second and the third injections than in the nicotine-treated control group. Concomitantly, animals receiving the nAChR modulator with nicotine demonstrated significantly more rapid learning in the MWM, lower anxiety response on the EPM, and significantly increased levels of BDNF mRNA in the FC, than in the nicotine only and untreated controls. Modulators of the nAChR may circumvent the problem of nicotine-induced desensitization, delaying the development of tolerance to nicotine and significantly extend the period of efficacy of long-term administration of nicotine.

# Abstract: HIGH-RESOLUTION REPRESENTATION OF LEFT AND RIGHT HEMISPACE IN AUDITORY CORTEX: A COMPREHENSIVE ELECTROPHYSIOLOGICAL AND BEHAVIORAL STUDY

**Authors:** M. Shachar, L. Y. Deouell

**Affiliations:** Department of Psychology, The Hebrew University, Jerusalem 91010, Israel

The representation of auditory space in the human cortex is unclear. While behavioral evidence suggests that the human auditory system is sensitive to azimuthal differences of less than 10 deg, neurons in the auditory cortex of cats and monkeys show broad receptive fields. We measured the neural correlate ofhuman spatial changedetection in 17 subjects, using the mismatch negativity (MMN) event related potential, a marker of preattentive auditory processing with major sources at the auditory cortex. We assessed both the electrophysiological and the behavioral change detection measures for 10, 20, 30, 40, 50, and 60 deg of deviation of sound location from the standard location, comparing the left and right hemispaces, as well as the direction of deviation (towards or away from the midline). The MMN was measured in an unattended session, while during the attended session we asked the subjects to detect auditory spatial changes. The MMN response displayed a double peak, more evident in the case where the standard was closer to the midline. Both peaks showed a significant effect of deviance, with a near linear relationship between the magnitude of spatial change and the MMN amplitude throughout the range. In contrast, speed and accuracy of performance improved up to 30 deg and then reached a plateau. There was a significant interaction between the direction of deviation and deviation magnitude for the early peak, with a steeper slope for the case in which the standard was lateral and the deviation was towards the midline. No difference was found between deviations on the left and on the right for either MMN peak. We conclude that the positions of auditory events are accurately tracked at the level of the auditory cortex, with no indication of hemispace differences.

*This work was supported by grant 477/05 from the Israel Science Foundation to LYD.*

# Abstract: EFFECTS OF PREGESTATIONAL STRESS ON OFFSPRING BEHAVIOR

**Authors:** A. Shahar-Dadon, R. Triester, N. Fredman, G. Gonen, M. Leshem, K. Braun

**Affiliations:** ^1^Department of Psychology, University of Haifa, Haifa 31905, Israel; ^2^Department of Zoology/Developmental Neurobiology, Otto von Guericke University, Magdeburg, Germany

Abuse during pregnancy is damaging to mother and infant, and has been extensively studied in rodents to reveal its neuroendocrine basis and how it impairs offspring development. However, similar assumptions about women abused before pregnancy, for example, in childhood, adulthood, or by rape, have not been investigated experimentally. Here, in rats, we investigated the effects of 7 days of unpredictable variable stress 2 weeks before mating (modeling adult stress), and immediately before mating (modeling stress before conception) on offspring behavior of 18 female SD rats. 6 females were mated 2 weeks after stress (group A), 7 females were mated immediately after the stress (group B), and 5 control females were left undisturbed until mating. All females were introduced to the males on the same day. Conception rates did not differ, group A 3 from 6, group B 5 from 7, and controls 3 from 5. When tested 2 months old in the open field, group A female offspring were most anxious and spent less time in the central space than group B or controls (*n* equals 11, 15, 13, resp). In the shuttle box, 70 days old group B male offspring avoided shock more, and escaped shock less than controls (*n* equals 6 and 8 resp). In the raised plus maze there were only gender differences. In the startle box, 60–65 days old male group A offspring responded significantly less than control males (*n* equals 6 and 6 resp). These results confirm and extend our previous findings, suggesting that pregestational stress can influence offspring behavior, and that there are significant gender differences.

*Supported by the German-Israel Fund, Grant no 902/00 to the fifth and sixth authors.*

# Abstract: ASSOCIATION BETWEEN THE REY-OSTERRIETH COMPLEX FIGURE TASK SCORESWITH CATECHOL-O-METHYLTRANSFERASE (COMT), DOPAMINE D4 RECEPTOR (DRD4), AND SYNAPTOSOMAL-ASSOCIATED PROTEIN (SNAP25)

**Authors:** I. Shalev, R. Levin, E. Heled, E. Lerer, L. Cohen, Y. Raz, D. Hoffine, R. P. Ebstein

**Affiliations:** ^1^Department of Psychology, The Hebrew University, Jerusalem 91010, Israel; ^2^Department of Brain and Behavior Science, The Hebrew University, Jerusalem 91010, Israel; ^3^Department of Human Genetics, The Hebrew University, Jerusalem 91010, Israel; ^4^Department of Neurobiology, The Hebrew University, Jerusalem 91010, Israel; ^5^S. Herzog Memorial Hospital, Jerusalem, Israel

The rey-osterrieth complex figure (ROCF) task assesses visuoconstructional ability and visual memory performance. It is used to investigate the relationship between executive function, especially in the prefrontal cortex (PFC), and visual memory and provides qualitative and quantitative information regarding the respondent's strategies and organizational approach. The ROCF generally requires the test-taker to draw a complex geometric design with multiple embedded details, first copying with the stimulus present, and then drawing from memory. Its demands aremultiple and include planning/organization; visual perception and construction; and memory encoding, storage, and retrieval processes. In the present study we tested association between several candidate genes and performance on the ROCF which was evaluated with the Boston Qualitative Scoring System (BQSS). 118 nonclinical subjects for whom genotype information was available were administered the ROCF and association between candidate genes was tested using a robust familybased association test (PBAT). Highly significant association was observed between COMT and long-term memory (*P* = .0007) as well as working memory (*P* = .001) and planning (*P* = .04). SNAP25 had negative correlation with planning (*P* = .03) and organization (*P* = .03). DRD4 (5 polymorphisms were examined: 4 in the promoter region and the exon3 repeat) was associated with planning (*P* = .007), organization (*P* = .01), short-term memory (*P* = .02) and longterm memory (*P* = .04). This is the first molecular genetic study demonstrating association between common polymorphisms and a complex neuropsychological task that robustly assesses executive function and working memory and is suitable for testing cognitively intact individuals.

# Abstract: THE SCALING OF “WINNER TAKES ALL” ACCURACY WITH THE POPULATION SIZE

**Authors:** M. Shamir

**Affiliations:** Center for BioDynamics and the Hearing Research Center, Boston University, USA

Empirical studies appear to support conflicting hypotheses in regard to the nature of the neural code. While some studies highlight the role of a distributed population code, others emphasize the possibility of a “single best cell” readout. One particularly interesting example of “single best cell” readout is provided by the “Winner Takes All” (WTA) approach. According to the WTA, every cell is characterized by one particular preferred stimulus, to which it responds maximally. The WTA estimate for the stimulus is defined as the preferred stimulus of the cell with the strongest response. From a theoretical point of view, not much is known about efficiency of “single best cell” readout mechanisms, in contrast to the considerable existing theoretical knowledge on the efficiency of distributed population codes. In this work, we provide a basic theoretical framework for investigating single best cell readout mechanisms. We study the accuracy of the WTA readout. In particular, we are interested in how the WTA accuracy scales with the number of cells in the population. Using this framework, we show that, for large neuronal populations, the WTA accuracy is dominated by the tail of the single-cell response distribution. Furthermore, we find that although the WTA accuracy does improve when larger populations are considered, this improvement is extremely weak compared to other types of population codes. More precisely, we show that while the accuracy of a linear readout scales linearly with the population size, the accuracy of the WTA readout scales logarithmically with the number of cells in the population.

*The author is supported by a fellowship from the Burroughs Wellcome fund.*

# Abstract: GLYCOGEN SYNTHASE KINASE-3: A NOVEL TARGET IN TRAUMATIC BRAIN INJURY

**Authors:** M. Shapira, A. Licht, A. Milman, C. G. Pick, E. Shohami, H. Eldar-Finkelman

**Affiliations:** ^1^Department of Human Molecular Genetics and Biochemistry, Tel Aviv University, Israel; ^2^Department of Anatomy, Tel Aviv University, Tel Aviv 69978, Israel; ^3^Laboratory of Biological Psychiatry, The Hebrew University, Jerusalem 91010, Israel

Glycogen synthase kinase-3 (GSK-3) has recently emerged as a potent in vivo regulator of neurodegenerative disorders and depression. We developed a selective competitive cell-permeable peptide inhibitor of GSK-3, L803-mts, and used it as a novel tool in evaluating its biological activity in various cell systems. In previous work we demonstrated that L803-mts provoked antidepressive-like activity in the animal model of depression, the mouse forced swimming test (FST). In the current study we investigated how GSK-3 is regulated by mild traumatic brain injury (mTBI), and whether or not its inhibition affects depressive behavior induced by this insult. Experiments performed in the closed brain-injury weight-drop model showed that hippocampus GSK-3bta was rapidly inhibited by mTBI. This was associated with activation of PKB, and accumulation of GSK-3-downstream target beta-catenin that was mainly detected in the dentate gyrus and CA3. mTBI produced a rapid depressive-like behavior 24 h post injury, however, treatment with L803-mts (i.c.v. injection), significantly reduced immobility, in fact, L803-mts prevented depression in mTBI-animals. We conclude that mTBI elicits an early “prosurvival” cascade of PKB/GSK-3beta/beta-catenin that likely supports neuroprotection. We suggest that GSK-3 inhibitors may present a new tool in the treatment of the adverse effects associated with TBI including cell apoptosis and depression.

# Abstract: GENETIC AND PHARMACOLOGICAL BLOCKADE OF INTERLEUKIN-1 SIGNALING ATTENUATES NEUROPATHIC PAIN AND SPONTANEOUS DISCHARGE FOLLOWING NERVE INJURY

**Authors:** Y. Shavit

**Affiliations:** Department of Psychology, The Hebrew University, Jerusalem 91010, Israel

Neuropathic pain, induced by peripheral nerve injury, is characterized by hyperalgesia, allodynia, and spontaneous ectopic discharge. Interleukin-1 (IL-1) plays a pain facilitatory role in baseline pain sensitivity, as well as in various inflammatory conditions. We tested the hypothesis that impaired IL-1-signaling influences neuropathic pain, using two mousemodels: mice with targeted deletion of the IL-1 receptor type I (IL-1rKO), and mice with transgenic overexpression of the IL-1 receptor antagonist (IL-1raTG), and their wild-type (WT) controls. Neuropathy was induced by cutting the L5 spinal nerve on one side (Chung's procedure), and mechanosensitivity was assessed for 7 successive days. WT mice developed neuropathic pain, as reflected by significant allodynia in the hindpaw ipsilateral to the injury. The mutant strains, however, did not display increased pain sensitivity in either hindpaw. Spontaneous ectopic neuronal activity of these strains was recorded in the dorsal root ganglion (DRG), 1, 3, and 7 days following nerve injury. In WT mice a significant proportion of the axons exhibited spontaneous ectopic neuronal activity, whereas in mutant mice only a minimal number of axons exhibited such activity. We also tested the effect of pharmacological blockade of IL-1 signaling, by chronic infusion of interleukin- 1 receptor antagonist (IL-1ra) in control adult mice, using subcutaneously osmotic micropumps, implanted two days prior to Chung's procedure. Saline-treated mice developed neuropathic pain, whereas the IL-1ra-treated mice did not display increased pain sensitivity. Correspondingly, in saline-treated mice a significant proportion of the axons exhibited spontaneous discharge, whereas in IL-1ra-treated mice only a minimal number of axons exhibited such activity. Taken together, these results indicate that IL-1 signaling plays an important role in the post-injury altered neuronal activity that underlies the development of neuropathic pain.

# Abstract: DIFFERENTIAL NEUROPROTECTIVE PROPERTIES OF ENDOGENOUS AND EXOGENOUS ERYTHROPOIETIN IN A MOUSEMODEL OF TRAUMATIC BRAIN INJURY

**Authors:** N. A. Shein, N. Grigoriadis, I. Yatsiv, A. Alexandrovich, J. Tsenter, M. Horowitz, E. Shohami

**Affiliations:** ^1^Department of Pharmacology, The Hebrew University, Jerusalem 91010, Israel; ^2^Department of Physiology, The Hebrew University, Jerusalem 91010, Israel; ^3^Rehabilitation Center, Hadassah Medical Center, Jerusalem, Israel; ^4^Pediatric Critical Care Unit, Hadassah Medical Center, Jerusalem, Israel; ^5^Department of Neurology, AHEPA University Hospital, Thessaloniki, Greece

Heat acclimation (HA) as well as post-injury treatment with erythropoietin (rhEpo) are established neuroprotective paradigms in a mouse model of closed head injury (CHI). HA-induced neuroprotection includes a reduction in cerebral edema and involves increased erythropoietin receptor (EpoR) expression, suggesting that endogenous Epo may be involved in reducing post-CHI tissue water accumulation and that the beneficial effects of HA possibly will be augmented by post-CHI rhEpo (exogenous Epo). Here we examined the effects of endogenous and exogenous Epo on cerebral edema and assessed the effects of supplemental exogenous Epo treatment on neuroprotective outcomes in HA animals. CHI was induced using a weight drop device. Tissue water content was measured at 24 hours post-CHI in brain segments taken from mice treated with either rhEpo or an anti-Epo antibody and appropriate controls. The efficacy of combining exogenous Epo treatment and HA was examined by subjecting HA and control (NT) mice to an object recognition test (ORT) as well as comparing the extent of neuronal degeneration by Fluoro-Jade B staining. Treatment with anti-Epo led to increased edema formation while exogenous Epo induced no beneficial effect on this outcome measure. ORT analysis and immunohistochemical findings reinforced HA and rhEpo as individual protective interventions but showed no advantage to combining the two strategies. In conclusion, the reduction of edema formation requires endogenous Epo but is unaffected by exogenous administration of this agent. Similarly, while increased expression of EpoR has been shown to be involved in HA-induced neuroprotection, additional supplementation of Epo does not lead to a further enhancement of the effect. HA-induced neuroprotection may therefore be comparable to certain features of CHI pathophysiology which are affected by preexisting neuroprotective mediators but cannot be further augmented by treatment aimed at increasing the levels of these agents.

# Abstract: INVOLVEMENT OF NOVEL GROWTH FACTORS IN ANIMALMODELS OF ALZHEIMER'S DISEASE AND OTHER TAUOPATHIES

**Authors:** N. Shiryaev, Y. Jouroukhin, A. Pinhasov, H. Rosenmann, I. Gozes

**Affiliations:** ^1^Human Molecular Genetics and Biochemistry, Sackler Medical School, Tel Aviv University, Tel Aviv 69978, Israel; ^2^Hadassah University Hospital, Jerusalem, Israel

Tauopathy is a neurodegenerative disease resulting from the abnormal phosphorylation and aggregation of tau protein. This pathology is generally accompanied by microtubule (MT) instability that finally results in degeneration of neurons. Inhibition of tau abnormality and recovery of MT structure are among the most promising therapeutic approaches to Alzheimer's disease and other tauopathies. NAP is a short neuroprotective peptide derived from activitydependent neuroprotective protein (Bassan et al [1]; Gozes et al [2]). Earlier studies in our laboratory showed that NAP can directly interact with tubulin, a subunit of MT, causing MT reorganization and an increase in nonphosphorylated tau (Divinski et al [3]; Gozes and Divinski [4]). The current research analyzed whether NAP could provide neuroprotection in cases of an in vivo pathology. We used a tau-transgenic (Tg) mouse model that expresses a human double mutant tau protein in the central nervous system. Daily treatment (5 days a week) of both wild type (wt) and Tg mice with intranasal NAP for a period of approximately 5 months significantly reduced anxiety-like behavior in the Tg male mice, with only a slight, nonsignificant decrease in the wt group. The same specific NAP effect on the Tg mice was demonstrated by the probe test during a Morris Water Maze (MWM) task, which assesses spatial short-term learning and memory. Eleven-month-old Tg animals exhibited learning and memory impairments in the MWM, as compared to the controls. This pathology was significantly improved by chronic NAP administration for 9 months. Furthermore, daily treatment with NAP resulted in improved short-termmemory in wtmice. Our results suggest that NAP is a promising drug candidate in ameliorating cognitive dysfunctions, associated with tau pathology and aging processes.

*Supported by BSF, ISF, Lily and Avraham Gildor Chair, Dr Diana and Zelman Elton Lab and Allon Therapeutics.*

## References

[1] Bassan M, Zamostiano R, Davidson A (1999). Complete sequence of a novel protein containing a femtomolar-activity-dependent neuroprotective peptide. *Journal of Neurochemistry*.

[2] Gozes I, Morimoto BH, Tiong J (2005). NAP: research and development of a peptide derived from activity-dependent neuroprotective protein (ADNP). *CNS Drug Reviews*.

[3] Divinski I., Holtser-Cochav M, Vulih-Schultzman I, Steingart RA, Gozes I (2006). Peptide neuroprotection through specific interaction with brain tubulin. *Journal of Neurochemistry*.

[4] Gozes I, Divinski I (2004). The femtomolar-acting NAP interacts with microtubules: novel aspects of astrocyte protection. *Journal of Alzheimer's Disease*.

# Abstract: SPONTANEOUS SEIZURES AND REDUCED HIPPOCAMPAL NEUROPEPTIDE Y INNERVATION UNDER TRANSGENIC EXCESS OF THE STRESS-ASSOCIATED READTHROUGH ACETYLCHOLINESTERASE VARIANT

**Authors:** S. Shoham, E. Kovalev, H. Soreq

**Affiliations:** ^1^Research Department, Herzog Hospital, Israel; ^2^Department of Biological Chemistry, Hebrew University, Jerusalem 91010, Israel

Psychogenic seizures present a yet unknown etiology, with no evidence of neuronal loss, frequent histories of stressful experiences and increased risk to develop post-traumatic stress disorder (PTSD). This suggested that chronic, stressinduced alterations in hippocampal gene expression may be causally involved. We have recently shown that chronic over-expression of the readthrough splice variant of acetylcholinesterase, AChE-R, accentuates long-term potentiation in murine hippocampus. Therefore, we explored the appearance of seizures in TgR transgenic mice over-expressing human AChE-R. Up to 8 months of age, TgR mice kept under regular laboratory conditions presented no seizures. However, 30% of > 14 months old female, but not male TgR mice not subjected to any stress paradigms began to display seizures. This was accompanied by variably declined density of neuropeptide Y (NPY)-positive axonal profiles in the hippocampal stratum lacunosum moleculare (SLM) and outer molecular layer of the dentate gyrus. NPY is known to moderate stress reactions and seizures. Intriguingly, NPY reductions are associated inversely with the severity of stress-associated symptoms, ranging from no behavioral alterations, to stereotypic behavior and to spontaneous seizures for mice presenting 45%, 70%, and 90% reduction in SLM NPY, respectively. In mice with seizures, NPY innervation of the mossy fiber terminal region was increased, supporting reports of others that this is a reaction rather than a cause of seizures. Our findings demonstrate genderassociated interactions of chronically over-expressed AChER with NPY neurotransmission, and suggest that such interactions cause a range of stress-related effects culminating with genesis of seizures.

*Supported by Israel Science Foundation.*

# Abstract: EXPLORING THE ORGANIZATION OF A LEARNING AND MEMORY SYSTEM BY LONG-TERM POTENTIATING VERSUS SEVERING OF A SPECIFIC LEARNING PATHWAY IN THE OCTOPUS BRAIN

**Authors:** T. Shomrat, G. Fiorito, B. Hochner

**Affiliations:** ^1^Department of Neurobiology, Interdisciplinary Center for Neural Computation, The Hebrew University, Jerusalem, Israel; ^2^Neurobiology Laboratory, Stazione Zoologica, Napoli, Italy

The median superior frontal (MSF) and the vertical (VL) lobes play an important role in the learning neural circuitry of cephalopods. In Octopus vulgaris we discovered LTP at the MSF to VL connection. Here we “short-circuit” this connection by inducing a global LTP, or “disconnect” it by severing the MSF to VL tract. Octopuses were pretrained to attack a white ball. Animals were anesthetized in seawater with 55mM MgCl2 and 1% ethanol (that does not block LTP induction). Five sites in the exposed MSF-VL area were tetanized (4 × 33 Hz 1 s). This reduced the level of LTP that could be induced in brains isolated afterwards. “Disconnection” was achieved by severing the tract between the MSF to VL. After 90 min of recovery, the animals were trained to avoid attacking a red ball by negative reinforcement (12Vac). Four consecutive “no-touch” was set as a learning criterion. Although there was no difference in the number of trials to reach criterion (tetanized: 4.2 ± 0.7, *n* = 14, sham: 5.6 ± 1, *n* = 15; *P* = .2, *t*-test), the tetanized animals learned faster, performing less touches by the 4th trail (*P* = .02, chi square). “Disconnected” animals required more trials than the sham to reach criterion (10.1 ± 1, *n* = 12, versus 4.8 ± 0.8, *n* = 9, *P* < .001, *t*-test). Memory recall was tested the following day. While there was no difference between groups in the number of “no-touch” in the first testing trial (*P* = .65 chi square), it was significantly higher in sham than in tetanized animals at the third trial (10 of 15 versus 4 of 14; *P* = .04, chi square). “Disconnected” animals did not show significant memory recall as 8 out of the 12 octopuses made errors in all 5 test trials in contrast to only 1 out of the 9 sham (*P* = .015, chi square). These results suggest that while the VL modulates the rate of short-term avoidance learning that takes place outside of the VL, the VL is a crucial site for the establishment of long-term memory traces.

*Supported by US-Israel BSF, Smith Laboratory, and Istituto Banco di Napoli-Fondazione.*

# Abstract: ANTAGONISTIC ROLES OF FULL-LENGTH CADHERIN AND ITS SOLUBLE BMP CLEAVAGE PRODUCT IN NEURAL CREST DELAMINATION

**Authors:** I. Shoval, C. Kalcheim

**Affiliations:** The Hebrew University, Jerusalem 91010, Israel

During neural crest ontogeny, an epithelial to mesenchymal transition is necessary for cell emigration from the dorsal neural tube. This process is likely to involve a network of gene activites which remain largely unexplored. We demonstrate that N-cadherin inhibits the onset of crest delamination both by a cell adhesion-dependent mechanism as well as by repressing canonical Wnt signaling previously found to be necessary for crest delamination by acting downstream of BMP4. Furthermore, N-cadherin protein, but not mRNA, is normally downregulated along the dorsal tube in association with the onset of crest delamination, and we find that this process is triggered by BMP4. BMP4 stimulates cleavage of N-cadherin into a soluble cytoplasmic fragment via an ADAM10-dependent mechanism. Intriguingly, when overexpressed, the cytoplasmic N-cadherin fragment translocates into the nucleus, stimulates cyclinD1 transcription and crest delamination, while enhancing transcription of *f* ò *f* {catenin. Hence, by promoting its cleavage, BMP4 converts N-cadherin inhibition into an activity likely to participate, along with canonical Wnt signaling, in the stimulation of neural crest emigration.

*Supported by SFB488 fromthe DFG.*

# Abstract: A NOVEL SYSTEM TO STUDY STEM-NICHE CELL INTERACTIONS

**Authors:** Z. Silverman, T. Kaisman, C. Brodski, D. Castellanos, J. Huang, J. Sagen

**Affiliations:** ^1^The Zlotowski Center for Neuroscience, Ben Gurion University of the Negev, Beer Sheva 84105, Israel; ^2^The Miami Project to Cure Paralysis, Miami, FL, USA

Stem cells are regulated by specialized cells in the so-called stem cell “niche.” In mammals, the identity of these specialized cells is largely unknown. The Sertoli cells of the testis represent a known example of an identified niche cell. Though poorly understood, Sertoli cells exert control over proliferation and differentiation of the stem cell population of the testis, the spermatagonia. To test if Sertoli cells can act as surrogate niche cells for other stem cell populations, cocultures of postnatal Sertoli cells and embryonic neural progenitor cells (NPCs) were produced and monitored for reciprocal communication. The neural progenitor cells were transfected with GFP prior to establishing the cultures. Immunofluorescence was performed to assess differentiation of the progenitor cells. The Sertoli and NPCs in a parallel set of cultures were separated by FACS and seeded in 96 well plates and subjected to an ELISA for the trophic factor GDNF. This factor originally discovered in glial cells in the CNS is also produced by Sertoli cells, and promotes proliferation in spermatagonia. NPCs exposed to Sertoli cells over 4 days exhibited increased differentiation into neurons and glia, compared with NPC-derived neurospheres maintained in culture alone. A potential mechanism for this observation was obtained in the Elisa assay carried out on the cultures. The concentration of GDNF in the NPCs alone was determined to be more than 700 pg/mg protein, which dropped to 150 pg/mg in the NPCs cocultured for 4 days with Sertoli cells. This indicates that the Sertoli cells inhibited GDNF production in the NPCs. Since GDNF promotes proliferation of progenitor cells in the testis, this effect could induce differentiation in the NPCs. Current efforts are underway to identify the Sertoli signals mediating the reduction in GDNF produced in the neurospheres and NPC-derived signals to Sertoli cells.

*Supported by ISF Grant no 572/04 to WSF.*

# Abstract: MIND YOUR LEFT: SUPERIORITY OF LEFT VISUAL FIELD PRESENTATION IN AMYGDALA RESPONSE TO FEARFUL FACES

**Authors:** T. Siman-Tov, A. Mendelssohn, T. Schonberg, D. Perry, L. Pessoa, L. G. Ungerleider, T. Hendler

**Affiliations:** ^1^Functional Brain Imaging Unit, Tel Aviv Sourasky Medical Center, Israel; ^2^Department of Psychology, Brown University, Providence, RI, USA; ^3^Laboratory of Brain and Cognition, National Institute of Mental Health, Bethesda, MD, USA

Is emotion processing spatially biased? Right hemisphere specialization in brain emotion processing has long been suggested by hemifield neuropsychological studies. However, functional brain imaging using full field presentations have failed to unequivocally prove such lateralization in emotional processing. In this fMRI study, fearful and neutral faces were presented to hemifields so that spatial and emotion processing could be studied independently. We assumed that an interaction between stimulus valence and visual field in the amygdala may (1) indicate emotional lateralization (2) help uncovering neural pathways mediating the interhemispheric processing of valence. Thirteen healthy females were scanned by MRI. Subjects viewed black and white faces presented to left or right visual field (LVF, RVF). Mixed design was composed of epochs for visual field and events for fearful or neutral facial expressions. To achieve visual field segregation as well as implicit emotional processing participants had to fixate and report on its color. Whole brain analysis showed increased activation of left amygdala to fearful versus neutral expressions (random effect, *P* < .004). This emotional selectivity was demonstrated for LVF but not for RVF epochs (random effect, *P* < .008). Region of interest analysis in the left amygdala showed greater emotional effect (fear > neutral) for LVF than RVF presentation (interaction, *F*(1, 12) = 6.71,*P* < .0237). A similar trend was found in right amygdala activations (*F*(1, 12) = 4.65, *P* < .0521). Our study shows that differential activation of the amygdala to fearful faces is significantly greater when faces are presented to LVF. These results support a study reporting right-lateralized brain activations for fearful faces in right visual areas and right amygdala (Noesselt et al, 2005). However, while both studies claim for superiority of LVF presentation in processing emotional facial expressions, our findings also point to dominance of left amygdala.

# Abstract: AMATHEMATICALMODEL THAT PREDICTS RAT WHISKER TRAJECTORIES DURING ELECTRICAL STIMULI OF THE FACIALMOTOR NERVE: A REVERSE ENGINEERING APPROACH

**Authors:** E. Simony, K. Bagdasarian, E. Ahissar

**Affiliations:** The Weizmann Institute of Science, Rehovot 76100, Israel

Sensation involves active repositioning of the underlying sensors. In order to investigate how rats explore their environment using their whiskers, an artificial whisking can be used in which the facial motor nerve is electrically stimulated. The stimulation that induces whisker protraction consists of a bipolar pulse train of specific frequency and amplitude, which propagates through the motor nerve fibers to activate the intrinsic and extrinsic muscles which control whisker position. The aim of this study is to model the whisker trajectory as a function of the principal stimulus parameters: instantaneous frequency and pulse amplitude. Thus, nerve conduction, muscle activation, muscle-follicle interaction, and follicle-whisker interaction are included in the “muscle-follicle system” whose transfer function is to be deciphered. First, we studied the impulse response of this system by measuring whisker trajectory due to single electrical bipolar pulse. If the system is linear, a convolution of the impulse response with the actual stimulus train should generate the response to the train. We tested this prediction and found that the system is not linear—a facilitatory nonlinear component, which stands for a nonlinear interaction between successive pulses, must be added. The nonlinear interaction can be accounted by several processes, among which are neuro-muscular facilitation and muscle-follicle dynamics. We are currently assessing the plausibility of each of these mechanisms. The transfer function between the motor nerve and whisker trajectory is the first step in reverse engineering vibrissal loops. It will be used to generate behaviorallymeasured trajectories in anesthetized animals, and to generate “white noise” stimuli which will facilitate characterization of neuronal transfer functions along the various loops.

*Supported by The United States-Israel Binational Science Foundation and the Minerva Foundation.*

# Abstract: BETA AND GAMMA OSCILLATIONS IN THE OLFACTORY BULB OF MICE

**Authors:** E. Sivan, C. Martin, J. Beshel, B. Hedinsson, L. M. Kay, N. Kopell

**Affiliations:** ^1^Center for Biodynamic, Boston University, 111 Cummington Street, MA 02215, USA; ^2^Department of Psychology, The University of Chicago, Chicago, IL 60637, USA

Various types of oscillations where recorded in the olfactory bulb (OB) of mammals. These oscillations were shown to be related to the behavioral state of the animal. Both beta oscillations (20–30 Hz) and gamma oscillations (60–100 Hz) were observed in mice performing odor discrimination tasks. In one task the oscillations evolved, as the animal learned the task, from low to high powered gamma oscillations. In another task the oscillations evolved to beta oscillations. Here, we investigate the underlying mechanisms that produce these oscillations and the changes in the network that enables the transition from one oscillation type to another. High power gamma oscillations were shown to be generated by the local circuitry of the dendro-dendritic synapses between the mitral cells (MC) and the granule cells (MC) in the OB. These oscillations are maintained when the GC dendrites are separated from their cell body. We have built a detailed model of both the MCs and the dendrites of the GCs and were able to produce these high power gamma oscillations. We hypothesize that the low power gamma oscillations are generated when nonsynchronized input from the pyriform cortex (PC) arrives to the OB. We show that the source of this nonsynchronized input may be the OB itself: the MCs in the OB excite the pyramidal cells in the PC that, in turn, excite the GCs back in the OB. We show that the wide range of delays seen in this excitation loop (15 ms–35ms) is enough to generate such nonsynchronized input and consequently, the low powered gamma oscillations. Beta oscillations were shown to require the existence of the excitation loop between the OB and the PC; that is, both input from the MCs onto the pyramidal cells in the PC and input from the PC onto the GCs are required. We hypothesize that during the learning of the task, in which beta appears, a subset of the synapses, with a lower range of delays, is strengthene to produce the beta oscillations.

*Partial support was provided to the fifth and sixth authors from the NIH/NSF program in CRCN.*

# Abstract: THE INVOLVEMENT OF THE RAPHE NUCLEI IN PARKINSON'S DISEASE

**Authors:** D. Sourani, G. Goelman

**Affiliations:** MRI/MRS Laboratory, Human Biology Research Center, Department of Medical Biophysics and Nuclear Medicine, Hadassah-Hebrew University Medical Center, Jerusalem, Israel

Parkinson's disease (PD) is primarily considered to be a movement disorder and is commonly related to the lack of dopamine. Yet, patients with PD frequently have mental disorders, such as cognitive decline and depression. These findings support the involvement of the serotonergic system. Our previous research suggests that there are changes in connectivity between the Raphe nuclei, which is the brain main 5-HT source, and the BG in PD rats. These findings might explain the mental disorders. In order to separate the neurological from the behavioral changes, unilateral 6-OHDA rats were used, since they are known to have no behavioral dysfunctions. The unique characteristics of manganese enhanced MRI (MEMRI) were utilized to compare dynamic connectivity changes between 6-OHDA and Sham rats. MnCl2 was injected to the Raphe nuclei (RIP) and was followed up to 96 hours post injection. Preliminary results: signal enhancement was observed in the injection site as well as in the middle habenular nucleus, subthalamic nucleus, and in field CA3 of hippocampus (CA3), a site which is known to be related to the limbic system. In addition, differences between PD and Sham rats were observed.

# Abstract: DIFFUSION TENSORMAGNETIC RESONANCE IMAGING OF PERIPHERAL NERVES

**Authors:** D. Stein, A. Neufeld, M. Graif, Y. Assaf

**Affiliations:** ^1^Department of Neurobiochemistry, Faculty of Life Sciences, Tel Aviv University, Tel Aviv 69978, Israel; ^2^fMRI unit, the Wohl Institute for Advanced Imaging, Tel Aviv Sourasky Medical Center, Israel

While MRI has been widely used for the study and assessment of the central nerve system (CNS), the peripheral nerve system (PNS) has received relatively little attention, at least partly, due to susceptibility induced artifacts and the small volume of the nerves. The main problem facing PNS imaging is rooted in the narrow structure of the limbs, resulting in a large boundary layer. Therefore the problem of susceptibility induced artifacts, appearing due to the passage of the magnetic field through the different mediums and distorting almost all the relevant data, was the first to overcome. In this study we have used diffusion tensor imaging (DTI) to obtain images of the median and ulnar forearm's nerves in 18 healthy subjects and 8 patients suffering from the carpal tunnel syndrome (CTS). We used DTI to extract the fractional anisotropy (FA), and the apparent diffusion coefficient (ADC) values of the median nerve proximally to, and within, the wrist's carpal tunnel (CT). We found that, for healthy subjects, at the CT FA values are elevated while the ADC values are significantly reduced. The CTS subjects showed the opposite trend with significantly lower FA at the CT indicating progressive edema of the nerve. With the use of a high-field magnet, a stronger gradient system and technical devices to reduce susceptibility artifacts, imaging of peripheral nerves using DTI is now feasible. Using the protocol described above the median and ulnar nerves can be well quantified and characterized using DTI for healthy and CTS subjects giving clear cut differentiation between the two groups. This could have tremendous effects for studying many related forearm nerve pathologies, as the carpal tunnel syndrome, as well as being the beginning of a vast study and assessment of the rest of the PNS.

# Abstract: COMPLEX NEURAL PROCESSING IN BACKWARD MASKING AS REVEALED BY VISUAL EVOKED POTENTIALS

**Authors:** A. Sterkin, O. Yehezkel, Y. Bonneh, A. Norcia, U. Polat

**Affiliations:** ^1^Goldschleger Eye Research Institute, Tel Aviv University, Tel Hashomer, Israel; ^2^Department of Neurobiology, Brain Research, TheWeizmann Institute of Science, Rehovot 76100, Israel; ^3^Smith Kettelwell Eye Research Institute, San Francisco, CA, USA

Masking is a tool that is widely used to study sensory information processing. When a ý mask is presented, 100 ms after the target (backward masking), the target's visibility is ý reduced. We developed a new visual evoked potentials (VEP) paradigm for exploring the ý neural processing involved in backward masking (BM) in the human visual cortex. The ý mask (M) was composed of two collinear high-contrast Gabor patches (GPs), spatially ý separated from the target location (T, low-contrast GP). The mask was presented at ý different ISIs after (1) T alone (BM-on-T) or (2) T + 2 flanking GPs (LM, BM-on-LM). The waveforms of the elicited responses for the various BM combinations and their amplitudes were compared within time-windows defined by the waveforms of the control responses of T, LM, and M stimuli at different ISIs. The amount of correlation between the waveforms and/or their amplitude modulation was regarded as BM effect. In both conditions, at the ISI = 50 ms the T response was not suppressed due to the integration (fusion) in the time domain of the responses elicited by T and M. In BM-on-T, a negative sign of the LM was suppressed up to ISI = 200 ms. However, while the M response in the BMon- T condition remained unchanged, it was strongly suppressed in BM-on-LM at the ISI = 100 ms and recovered at the ISI = 200 ms. Thus, the results suggest that BM effect reflects complex neural processing of temporal order and saliency of each of the components. Remarkably, it was also relevant in which modality the recalled objects were originally recognized in the memory tasks. Recall of the list of haptically recognized objects elicited greater activation than its auditory parallel in the occipital lobe of the blind. More evidence for the importance of encoding modality was found in “memory traces” in the relevant sensory (somatosensory or auditory) cortex. These results demonstrate the relevance of the original sensory encoding source to the cortical patterns of activation during recall in the congenitally blind.

*Supported by ISF “Center of Excellence” and US-Israel BSF.*

# Abstract: COCAINE SELF-ADMINISTRATION DECREASES NEUROGENESIS IN THE DENTATE GYRUS OF THE HIPPOCAMPUS OF ADULT RATS

**Authors:** E. Sudai, I. Gispan, I. Roth-Deri, N. Kinor, G. Yadid

**Affiliations:** Faculty of Life Sciences, Bar-Ilan University, Israel

Drug addiction is a chronic brain disorder, characterized by loss of control over drug consumption. The neurobiology of addiction is traditionally thought to involve the reward system of the brain. This system comprises DA neurons with cell bodies in the ventral tegmental area (VTA) of the midbrain and projection areas of these neurons in the limbic forebrain and in particular the nucleus accumbens (NAC). However, the hippocampus has received renewed interest for its potential role in addiction. Part of this attention is due to the fact that drugs of abuse are potent negative regulators of the neurogenesis in the adult hippocampus. We investigated the effect of chronic cocaine self-administration on neurogenesis in the dentate gyrus of the hippocampus. Rats (300–320 gr) were trained to self-administer cocaine (1.5mg/kg) using the FR1 paradigm. After reaching maintenance, rats were injected with 5-bromodeoxyuridine (BrdU). 28 days later they were euthanized and their brains stained with antibodies to BrdU and the mature neuronal marker NeuN. Significantly less newly formed neurons (double positive for BrdU and NeuN) were found in the dentate gyrus of rats that self administrated cocaine. These results suggest that cocaine-induced alterations in the hippocampus, a region central to learning and memory, in accordance with the intense memories associated with drug use and the propensity to drug relapse.

# Abstract: THE PARADOX OF NEUROPATHIC PAIN

**Authors:** M. Tal, J. N. Campbell, M. Devor, E. Eisenberg, Y. Shavit

**Affiliations:** ^1^Department of Anatomy and Cell Biology, School of Medicine, Research on Pain Center, The Hebrew University, Jerusalem 91120, Israel; ^2^Department of Neurology, Johns Hopkins Medical School, Baltimore, MD 21205, USA; ^3^Institute of Life Sciences, The Hebrew University, Jerusalem 91010, Israel; ^4^Department of Neurology, Rambam Medical Center, Haifa, Israel; ^5^Department of Psychology, The Hebrew University, Jerusalem 91010, Israel

Chronic neuropathic pain can result from nerve injury or disease and it is often refractory to analgesic therapy. It is characterized in humans by spontaneous pain and abnormal evoked pain such as allodynia and hyperalgesia. Neuropathic pain is thought to result from nerve injury-triggered pathophysiological changes in both the peripheral and the central nervous systems. The details, however, are uncertain. In addition to its clinical significance, neuropathic pain is important from a basic scientific point of view because of the fundamental paradox that it poses. Blockade of nerve conduction due to injury ought to reduce sensation, causing hypoesthesia. Why, then, is there frequently amplification of sensation, with hyperesthesia and ongoing pain? Both the clinical imperative and the scientific mystery are subjects of this symposium. Elon Eisenberg will address the prevalence, clinical manifestations, and treatments of chronic pain in general, and some key neuropathic pain states in particular. James Campbell will address the issue of pain-control in the spinal cord. Adjustment of excitatory mechanisms, “central sensitization,” can cause normally innocuous stimuli to be felt as painful. Specific types of peripheral neuronal input are associated with nerve injury trigger andmaintain this “central sensitization.” Marshall Devor will consider the regulation of neuronal excitability in the pain system, and how genetic polymorphisms can lead to individual differences in pain susceptibility. Finally, Yehuda (Udi) Shavit will discuss the role of cytokines in neuropathic pain. Data from animals with genetically impaired IL-1 signaling indicate that this cytokine plays an intimate role in the altered neuronal activity that underlies the development of neuropathic pain following nerve injury. The symposium will shed light on the neural mechanisms that underlie neuropathic pain and that cause so much suffering in the clinical setting.

# Abstract: STATE-DEPENDENT SYNAPTIC PLASTICITY IN PURKINJE CELLS

**Authors:** Z. Tal, E. Chorev, Y. Yarom

**Affiliations:** ^1^Department of Neurobiology, Life Science Institute, The Hebrew University, Jerusalem, Israel; ^2^Interdiscplinary Center for Neural Computation, The Hebrew University, Jerusalem, Israel

One of the popular theories of cerebellar function assumes that the cerebellum stores memory traces at the parallel fibers (pf) synapse. According to this theory, the climbing fibers (cf) control the learning process by inducing long-term depression (LTD) of the simultaneously activated pf synapses. In a recent study we showed that Purkinje cells (PCs), under in vivo conditions, display bistability of their membrane potential. The bistability is an intrinsic property of the neurons, such that the membrane potential can remain either in a hyperpolarizing quiescent state (“down” state) or in a depolarizing active state (“up” state). In the current study we examined to what extent the bistability of PCs in a slice preparation affects plastic processes. To that end, we paired local application of glutamate with the state of the PCs. At the end of this pairing procedure, we measured the voltage response induced by a pulse of glutamate and compared it to the control measurement. We found that 20 pairs at repetition rate of 1Hz was sufficient to induce a robust long term depression (LTD) in the response to glutamate applications. We concluded that the up state of the PCs membrane potential could produce LTD. Furthermore, the fact that LTD can be induced by the up state can account for the loose temporal relationship between the pf and the cf that has been reported in the literature.

# Abstract: THE ROLE OF BRAIN-DERIVED NEUROTROPHIC FACTOR (BDNF)WITHIN HIPPOCAMPAL SUBREGIONS IN DEPRESSIVE BEHAVIOR: EVALUATION BY LOCALIZED BDNF SILENCING

**Authors:** D. Taliaz, D. E. Dalit, R. Gersner, M. Lebow, A. Zangen

**Affiliations:** Department of Neurobiology, Weizmann Institute of Science, Rehovot 76100, Israel

Previous studies demonstrated that BDNF is down-regulated in human suicide victims and in animal models of depression, and that long-term antidepressant treatment upregulate BDNF levels, especially in the hippocampus. However, it is still not clear whether reduction of BDNF levels in a specific brain region actually causes depressive behavior or whether BDNF reduction in depressed subjects is just a side effect. In order to test this, localized RNA interference (RNAi), was utilized to induce reduction of BDNF expression in hippocampal subregions by localized injections of doublestrand RNA (dsRNA) that contains a homologous sequence to the selected gene. For that purpose, a number of dsRNAs specific to rat's BDNF mRNA (dsBDNF) were cloned into lentiviral (LV) vectors to allow long-term interference. Vectors containing dsBDNF or mock controls were injected bilaterally via implanted cannulea into the CA3, dentate gyrus (DG), and subiculum (Sub) of the hippocampus in rats. A battery of behavioral tests was used to assess aspects of depressive behavior including anhedonia, motivation, exploration, and fatigue in the different experimental groups. In vitro, our dsBDNF vectors reduced BDNF levels by 70%. In vivo, injections of vectors containing the dsBDNF into both the CA3 and the subinduced reductions in sucrose preference, mobility in the forced swim test and home cage locomotion as compared to the controls. However, exploration of a novel environment test was not affected. Injection of vectors containing dsBDNF into the CA3 alone did not affect any of these behavioral tests. Injection of the vectors into the DG tended to induce depressive symptom as observed in the CA3-subgroup, however additional groups still need to be analyzed. These results demonstrate the correlation between reduced levels of BDNF at subregions of the hippocampus and depressive symptoms including anhedonia, reduced motivation, and fatigue.

# Abstract: FUNCTIONAL STATE OF VISUAL SYSTEM IN AMBLYOPIA

**Authors:** E. Tsitsiashvili

**Affiliations:** Georgian National Center of Ophthalmology and Neurology, Tbilisi, Georgia

At the present time, the pathophysiological mechanisms of amblyopia remain a puzzle. The objective of our investigation was to estimate functional state of the visual system at various kinds of a high degree amblyopia. Clinical researches were based on the analysis of results of 167 patients (207 eyes) with anisometropic (52 patients, 52 eyes), disbinocular (75 patients, 75 eyes), and refractive (40 patients, 80 eyes) amblyopias of high degree at the age from 5 to 17 with visual acuity from 0.02 up to 0.2 on the amblyopic eye, and also 20 healthy children, 40 eyes. In addition to routine ophthalmologic methods of research, we also used the special computer programs: “ZEBRA” (A. M. Shamshinova, A. E. Belezerov) psychophysical method of visiocontrastometry for determination of the topography of spatial contrast sensitivity, “OFF-ON” (A. M. Shamshinova, A. S. Petrov) psychophysical method of static campimetry for determination of the topography of color sensitivity and activity of on-off channels of the retinal cone system. The symptoms of functional disturbance of visual system at various kinds of a high degree of amblyopia are characterized by a decrease in achtomatic and color spatial contrast sensitivity in the domain of high and middle frequency, in contrast sensitivity-activity of onoff channels of the retinal cone system, in color sensitivity to unsaturated red and green colors, as well as by normal sensitivity to saturated colors and dramatic changes in color sensitivity in the paracentral zone (5°–10°) of the retina. So, changes in various channels of visual system and topography of these changes help to understand the mechanisms of disturbances of visual functions in amblyopia, can shed light on the localization of functional changes at this pathology, and make possible to seek the ways for adequate strategy of treatment.

# Abstract: ALTERATIONS IN THE NEURAL CELL ADHESION MOLECULE L1 EXPRESSION IN THE LIMBIC SYSTEM FOLLOWING JUVENILE STRESS-POTENTIAL RELEVANCE FOR MOOD AND ANXIETY DISORDERS

**Authors:** M. M. Tsoory, A. Guterman, G. Richter-levin

**Affiliations:** Psychology Department and The Brain and Behavior Research Center, University of Haifa, Haifa 31905, Israel

Epidemiological studies indicate that childhood trauma is predominantly associated with higher rates of both mood and anxiety disorders, which were associated with altered limbic system functioning. Exposing rats to stress during juvenility has comparable effects and was suggested as a model of induced predisposition for these disorders (Avital and Richter-Levin, 2005; Tsoory and Richter-Levin, 2005; Tsoory et al, 2006). The neural cell adhesion molecule L1 is critically involved in development, activity-dependent synaptic plasticity, and learning processes. Since chronic stress protocols affect L1 functioning, and induce heightened anxiety and impaired cognitive and neural function in adult rats, the current study utilized the juvenile stress model and examined the effects of juvenile stress on L1 expression both soon after the exposure and in adulthood with or without additional exposure to acute stress in adulthood. Juvenile stress increased L1 expression levels in the limbic system, BLA, CA1, DG, and EC, both soon after the juvenile stress exposure as well as in adulthood. Furthermore, exposure to juvenile stress has shifted the set point of the normative developmental decrease in the expression of L1. Though acute adulthood stress alone increased L1 expression throughout the limbic system, adulthood acute stress exposure that followed a juvenile exposure did not add to the juvenile stress induce increase in the BLA and DG, but attenuated it in the CA1 and EC. Our data suggest that exposure to juvenile stress may hinder the limbic system.

# Abstract: INITIATION OF ACTION POTENTIALS IN NEOCORTICAL NEURONS: DOES THE HODGKIN-HUXLEY THEORY DESCRIBE IT?

**Authors:** M. Volgushev, F. Wolf

**Affiliations:** ^1^Department of Neurophysiology, Ruhr-University Bochum, Bochum 44780, Germany; ^2^Institute of Higher Nervous Activity and Neurophysiology Russian Academy of Sciences, Moscow, Russia; ^3^MPI for Dynamics and Self-Organization, Gottingen, Germany; ^4^Bernstein Center for Computational Neuroscience, Gottingen, Germany

A ubiquitous step in operation of neurons and neural networks is encoding of the incoming information into sequences of action potentials (APs). Recent evidence from theoretical and experimental studies has questioned the direct applicability of the reigning theory of cellular electrogenesis—the Hodgkin-Huxley theory—to the AP initiation in central mammalian neurons. We have shown the two salient features of AP initiation in neocortical neurons in vivo: their sharp, step-like initiation dynamics and large variability of the onset potentials are virtually impossible to capture by Hodgkin-Huxley type model with realistic parameter settings. Our quantitative analysis of AP waveforms and initiation dynamics in a large population of mammalian neocortical neurons and invertebrate (snail) neurons showed that the Hodgkin-Huxley formalism could explain AP initiation in most of snail neurons, but not in vertebrate neocortical neurons. To describe the AP initiation dynamics, we used the ratio of errors of exponential over the piecewise linear fits of the initial portion of AP in the phase plot representation. This quantitative measure segregates the AP initiation dynamics in two fundamentally different classes: a gradual Hodgkin-Huxley-type AP initiation usual in the snail neurons, and the fast AP initiation typical for the neocortical neurons. Segregation of neurons by the ratio of fit errors corresponded well to the segregation by other AP parameters. Under the conditions which diminish functioning of voltagegated sodium channels (TTX or reduced extracellular Na+), not only the amplitude of APs in neocortical neurons was decreased, as the canonical Hodgkin-Huxley theory predicts, but also their initiation dynamics was altered, becoming slow and gradual. These results support our hypothesis that sharp, step-like onset dynamics of neocortical APs is due to cooperative activation of voltage-gated sodium channels.

*Supported by the DFG, HFSP, and the Max-Planck Society.*

# Abstract: GLUCOCORTICOID RECEPTORS AND BETAADRENOCEPTORS IN BASOLATERAL AMYGDALA ARE REQUIRED FOR MODULATION OF SYNAPTIC PLASTICITY IN HIPPOCAMPAL DENTATE GYRUS, BUT NOT IN AREA CA1

**Authors:** R. Vouimba, G. Richter-Levin

**Affiliations:** Brain and Behavior Research Center, University of Haifa, Haifa 31905, Israel

The basolateral amygdala (BLA) is a key structure in a memory-modulatory system that regulates stress and stress hormones (glucocorticoid and noradrenaline) effects on hippocampal functioning. We have shown previously that priming the amygdala differentially affected plasticity in the hippocampal dentate gyrus (DG) and CA1, and mimicked acute stress effects on plasticity in these two subregions. In the present study, we investigated the mechanisms that mobilize the BLA to differentially alter plasticity in DG and CA1. Glucocorticoid receptors antagonist RU 38486 or betaadrenoceptors antagonist propranolol were micro-infused in the BLA, 10 minutes prior to BLA activation-induced modulation of long-term potentiation (LTP) in DG and CA1. Results showed that blockade of glucocorticoid or noradrenergic transmission in BLA suppressed the enhancing effect of BLA activation on DG LTP. In contrast, neither glucocorticoid nor noradrenergic transmission in the BLA are necessary for LTP induction and for the impairing effect of amygdala activation on CA1 LTP. These findings provide further evidence for a differential amygdala control of hippocampal subregions as well as for differential memory processes involving CA1 and DG. They also provide insight into how stress hormones exert their actions on the circuits involved in these processes.

*This research was supported by an EU project Grant no 512012 to the second author.*

# Abstract: VISUAL SEARCH IN VIRTUAL DEPTH

**Authors:** M. Wagner, W. Ehrenstein

**Affiliations:** ^1^Smith Psychobiology Laboratory, The Hebrew University, and College of Judea and Samaria, Ariel, Israel; ^2^Leibniz Research Center for Working Environment and Human Factors, University of Dortmund, Germany

Binocular vergence eye-movements were studied under conditions that require shifting of search between fronto-parallel and virtual depth layers. Six normal-sighted participants were given a feature search task in two superimposed displays, a 2D (18 or 30 items) and a 2(1/2)D (24 or 42 items) layer of otherwise identical, gray-shaded features on a dark surface. The respective target layer location was pre-cued by varying the form of the fixation sign, while attention shifts were controlled for by valid or invalid pre-cues. An EyeLink- II system served to record binocular eye-movements with unrestrained head posture. In addition, we took manual response times (RTs) for target detection. The eyes converged or diverged depending on the direction in virtual depth search: shifts from 2D to 2(1/2)D search layer elicited divergence, while convergence was caused by shifts from 2(1/2)D to 2D search. Interestingly, even single surface search required two steps in eye movements and RTs did not depend on surface type or set size. The present study demonstrates that vergence eye movements can be controlled by virtual depth (top-down mechanisms) and that surface selection precedes visual search of targets, even in a preattentive (“pop-out”) situation.

*Supported by the National Institute for Psychobiology.*

# Abstract: PHASIC AND TONIC RESPONSES IN THE MAIN AND ACCESSORY OLFACTORY BULBS: DIFFERENT MODES OF INFORMATION PROCESSING

**Authors:** S. Wagner, Y. Yarom

**Affiliations:** ^1^Neurobiology and Ethology Department, Haifa University, Haifa 31905, Israel; ^2^Neurobiology Department, Life Sciences Institute, The Hebrew University, Jerusalem, Israel

To ensure survival, animals must engage in social and reproductive interactions with other individuals of the same species. In many mammalian species, communication within the animal group relies on the emission and detection of specific chemical cues, the pheromones. It has been traditionally assumed that pheromones detection is solely mediated by the accessory olfactory system(AOS, also called vomeronasal system). However, a growing number of recent studies have implicated the main olfactory system (MOS) in pheromoneevoked responses. These findings have raised a general question: what is the division of labor between these two systems in processing pheromonal information? We have previously showed that the two systems significantly differ in their wiring pattern (Wagner et al [1]). Using acute brain slices, we now show that these two systems differ also in their physiological properties. Mitral cells in the main olfactory bulb (MOB) react with a strong bursting response to a brief electrical shock given to the sensory fibers. In contrast, mitral cells of the accessory olfactory bulb (AOB) respond to the same stimulus with only weak graded responses. Given a strong enough stimulus, however, AOB mitral cells maintained an elevated firing rate for 10–30 seconds, a reaction which was never observed in the MOB. We further show that distinct intrinsic properties of these cells underlie the difference in their responses. We propose that the MOS serves as an analytic tool to dissect the blend of pheromones into its individual components. In contrast, the AOS process the mixture of cues as a whole. Hence, both sensory systems may cooperatively analyze pheromonal cues, each extracting a different aspect of the sensory information.

*Supported by the National Institute for Psychobiology, Interdisciplinary Center for Neural Computation.*

## References

[1] Wagner S, Gresser AL, Torello AT, Dulac C (2006). A multireceptor genetic approach uncovers an ordered integration of VNO sensory inputs in the accessory olfactory bulb. *Neuron*.

# Abstract: CRH-INDUCED REGULATION OF SPINE MORPHOLOGY IN CULTURED HIPPOCAMPAL NEURONS

**Authors:** S. Westerholz, H. Herzog, C. Helmeke, M. Segal, K. Braun

**Affiliations:** ^1^Department of Zoology/Developmental Neurobiology, Otto-von-Guericke University, Magdeburg, Germany; ^2^Institute for Electronics, Signal Processing, and Communications, Otto-von-Guericke University Magdeburg, Germany; ^3^Department of Neurobiology, The Weizman Institute, Rehovot 76100, Israel

Stress releases endogenous CRH, and CRH as well as CRH1 receptors are expressed in neonatal hippocampal neurons. Furthermore, it has been shown that central CRH application results in endocrine, autonomical, and behavioral reactions comparable to the effects of stress, for example, in an activation of HPA axis and sympathic nervous system. Stress produces structural alterations of neuronal morphology, including dendritic atrophy and loss of excitatory synapses in the hippocampal formation in the intact animal. Here we tested the effects of elevated CRH-mediated neurotransmission on the morphology of dendritic spines and the density of their associated presynaptic terminals. Cultured eGFP transfected primary hippocampal neurons (DIV 7) were incubated with (1) 500 nM synthetic CRH, (2) 560 nM astressin (CRH1,2-antagonist), (3) CRH + astressin for 24 hours. Subsequently, pharmacologically stimulated and control cultures were fixed and dendritic segments of spine bearing neurons were visualized in optical stacks using laser scanning confocal microscopy (LSM 510 Meta, Zeiss). Distinct shape parameters of presumed excitatory spine synapses and spine density were analyzed using a newly developed image analysis software, which allows 3D-measurements of dendritic spines (length, size of spine heads and necks, volume, diameter, shape factors). We found that elevation of CRH neurotransmission does not affect spine density, but induces a shift of certain morphologically defined spine classes. The density and the length of thin spines decreased after CRH treatment, whereas the density of stubby spines increased. These morphological changes might indicate a CRH-mediated transition from immature spines (thin) towards more mature spines (stubby), which most likely changes the functional properties of this in vitro synaptic network.

*Supported by a grant of the State of Saxony-Anhalt and Morris Belkin Visiting Professorship at WIS.*

# Abstract: INTERLEUKIN-1 SIGNALING MODULATES STRESS-INDUCED ANALGESIA

**Authors:** G. Wolf, R. Yirmiya, T. Kreisel, I. Goshen, J. Weidenfeld, Y. Shavit

**Affiliations:** ^1^Department of Psychology, The Hebrew University, Jerusalem 91010, Israel; ^2^Department of Neurology, Hadassah University Hospital, Jerusalem, Israel

Exposure to stressful stimuli is often accompanied by reduced pain sensitivity, termed “stress-induced analgesia” (SIA). In the present study, the hypothesis that interleukin- 1 (IL-1) may play a modulatory role in SIA was examined. Two genetic mouse models impaired in IL-1-signaling and their wild type (WT) controls were employed: targeted deletion of the IL-1 receptor type I (IL-1rKO) or transgenic overexpression of the IL-1 receptor antagonist (IL-1raTG). Another group of C57 mice was acutely administered with IL-1 receptor antagonist (IL-1ra). Mice were exposed to 2 minutes swim stress at one of three water temperatures: 32°C (mild stress), 20–23°C (moderate stress), or 15°C (severe stress); and then tested for pain sensitivity using the hot-plate test. Corticosterone levels were assessed in separate groups of WT and IL-1rKO mice following exposure to the three types of stress. Mild stress induced significant analgesia in the two WT strains and saline-treated mice, but not in the mutant strains or the IL-1ra-treated mice. Similarly mild stress induced significantly elevated corticosterone levels in WT mice, and blunted corticosterone response in IL-1Rko mice. In contrast, bothWT and mutant strains, as well as IL- 1ra-treated mice, displayed analgesic and corticosterone responses following moderate and severe stress. Interestingly, the analgesic response to moderate stress was markedly potentiated in the IL-1rKO and IL-1raTG mice as well as in IL-1ra treated mice, compared with their WT controls and saline-treated mice. The present results support our previous findings that in the absence of IL-1, stress response to mild stress is noticeably diminished. However, the analgesic response to moderate stress is markedly potentiated in mice with impaired IL-1 signaling, corroborating the antianalgesic role of IL-1 in several pain modulatory conditions, including SIA.

# Abstract: SEEING THE IMPOSSIBLE

**Authors:** M. Wolf, S. Hochstein

**Affiliations:** ^1^Neurobiology Department, The Hebrew University, Jerusalem Israel; ^2^ICNC, The Hebrew University, Israel

In binocular rivalry, two stimuli compete for consciousness. The brain does not fuse different images seen by the two eyes, instead, perception alternates between two views. Sometimes one eye's view is dominant for longer periods than that of the other eye, and sometimes perception mixes the two. High-contrast figures win more often than lowcontrast figures, brighter than dimmer, moving than stationary, and high spatial frequency than low (Levelt 1965). Both high-level and low-level mechanisms may be responsible for the rivalry pattern, but it is assumed that complex images, scenes, or other pictures are represented at higher cortical levels, and therefore affected more by highlevel mechanisms. We found (ISfN, 2005) that when competing words versus nonwords, subjects spent a longer time perceiving nonwords. We explained this advantage of nonwords over words as saliency of interesting harder stimuli over familiar easier ones. We have now extended our study to the relative dominance of possible versus impossible figures in binocular rivalry. Two red/green pictures were superimposed, an impossible one and a quite similar possible one. Participants viewed the pictures through red/green glasses and tracked the changes in perception by pressing one of two keys corresponding to the two pictures. We found that with simple pictures (as with words) impossible figures were generally dominant for longer periods. On the other hand, with complex pictures, possible figures had longer dominance periods. This result is consistent with our hypothesis that for higher cortical level mechanisms, more interesting stimuli are more salient and more dominant in binocular rivalry.

*Supported by Center of Excellence Grant from the ISF and the US-Israel Binational Science Foundation.*

# Abstract: THE SPIKE AFTERDEPOLARIZATION IN HIPPOCAMPAL PYRAMIDAL NEURONS: A TAIL OF TWO CURRENTS

**Authors:** Y. Yaari

**Affiliations:** Department of Physiology, Hadassah School of Medicine, The Hebrew University, Jerusalem, Israel

In principal neocortical and hippocampal neurons, the spike is followed by a conspicuous afterdepolarization (ADP). The waveform of this afterpotential determines whether the neuron will generate a solitary spike or a high-frequency burst of spikes. The spike ADP waveform reflects an interplay between two opposing afterspike (tail) currents, namely, persistent Na+ current and muscarinic-sensitive K+ current (M-current). Both electrophysiological measurements and computer simulations indicate that Ca2+ currents flowing during or after the spike are unlikely to contribute to the generation of the spike ADP. Surprisingly, however, pharmacological blockage of N- and P-/Q-type Ca2+ currents markedly suppresses the spike ADP, indicating that these Ca2+ currents do participate in ADP electrogenesis. How can this discrepancy be resolved? Come and see.

# Abstract: NOVEL ASYMMETRIC FLUOROGENIC ORGANOPHOSPHATES FOR THE DEVELOPMENT OF ORGANOPHOSPHATE HYDROLASES WITH REVERSED STEREO-SELECTIVITY

**Authors:** G. Yacov, S. Teitlboim, S. Yishay, L. Tveria, O. Limanovich, R. Adani, M. Kushnir, H. Meshulam, G. Amitai

**Affiliations:** Division of Medicinal Chemistry, Israel Institute for Biological Research, Ness-Ziona, Israel

Organophosphates (OP) nerve agents such as cyclosarin (GF) and insecticides such as paraoxon exert their toxicity by irreversible inhibition of acetylcholinesterase (AChE) in the cholinergic nervous system. OP hydrolases (OPH) may serve as catalytic scavengers and noncorrosive decontamination of toxic OP's on sensitive surfaces including skin. We have developed novel asymmetric fluorogenic organophosphates (Flu-OP) as new molecular probes for screening and selection of new variants of bacterial OPH or mammalian paraoxonase (PON1) by directed evolution. It was previously demonstrated that these OPHs degrade the less toxic P(+) stereoisomer more rapidly than the toxic P(−) isomer. Our major goal is to use this stereoselective degradation in order to separate the more toxic stereoisomer out of its racemic mixture. Subsequently, this isomer will be used for the selection of new PON1 variants with reversed stereoselectivity. Eight nerve agents analogous Flu-OPs were synthesized containing ethyl (E), cyclohexyl (C), pinacolyl (P), and isopropyl (I) groups attached to methyl phosphonyl (MP) moiety. The fluorescent leaving groups are OH-MeCyC or OHDDAO. The inhibition kinetics of human AChE (HuAChE) and horse serum BChE (Hs-BChE) by Flu-OPs and the time course of hydrolysis by nine PON1 variants were determined. Degradation kinetics of Flu-OPs was found to be biphasic indicating faster hydrolysis of the less toxic P(+) optical isomer. Interestingly, wt PON1 caused only 50% degradation of racemic EMP-MeCyC and CMP-MeCyC. We used this phenomenon for the enzymatic separation of the P(−) isomer of CMP-MeCyC. The bimolecular rate constant (ki) for HuAChE inhibition by the isolated P(−) isomer of CMPMeCyC is 5 fold greater than that of the P(+) isomer. The separated P(−) isomer of CMP-MeCyC will be used for the selection of new PON1 variants with reversed stereoselectivity by directed evolution employing fluorescence activated cell sorting (FACS) in emulsion compartments.

*Supported by Contract M/SAB1/3/A007 to the ninth author from MOD, Germany.*

# Abstract: FAMILIARITY DETECTION MEMORY: MONKEYS RECOGNIZING ONCE-SEEN IMAGES

**Authors:** V. Yakovlev, S. Romani, D. Amit, S. Hochstein

**Affiliations:** ^1^Institute of Life Sciences and International Center for Neural Computation, The Hebrew University, Jerusalem, Israel; ^2^Racah Institute of Physics and International Center for Neural Computation, The Hebrew University, Jerusalem, Israel; ^3^Doctoral Programin Neurophysiology, University of Rome, La Sapienza, Italy

We studied multiple-stimulus working memory in two macaques, comparing abilities for recognizing stimulus repetition when using well-trained sets of images versus novel images that the monkey had never seen before. The task was to differentiate between stimuli that had not been previously presented in the trial, and a repeated stimulus. Since it is assumed that working-memory delay-activity is required for recognizing a repetition, and that it takes many presentations to establish delay activity, it should be difficult for monkeys to detect repetition of a one-shot learned stimulus. In fact, the opposite was the case. In each trial the monkeys were shown a sequence of 2–7 stimuli, with the last always being a repetition of a previous image. This paradigm tested simultaneous working memory for all trial stimuli. In Experiment 1, we used a fixed set of 16 images, presenting a random selection from among them in each trial. Each stimulus was shown hundreds of times during the experiment, so these stimuli were very familiar to the monkeys. Performance was very good, with 82% hits and only 4% false positives (FP). In Experiment 2 we challenged working memory by introducing a set of 12 000 novel images of different objects, scenes, and symbols, neither of which the monkey had seen before. Surprisingly, the monkeys demonstrated even better performance, 92% hits and 2% FPs. We introduced “catch” trials, where an image from a preceding trial was presented as a sample on a subsequent trial. Here, too, FPs were only 15%. The fact that not all catch trials led to FPs suggests that monkeys activate a “reset” mechanism between trials. We conclude that monkeys use a different strategy for novel stimuli, not based on delay activity but on the dynamics of the visual response when a stimulus is repeated versus when it has never been seen before. Both single cell recording in the inter-trial interval and model simulations support this presumed mechanism (Romani et al ISfN 2006).

*Supported by the National Institute for Psychobiology in Israel and a Center of Excellence grant from the ISF.*

# Abstract: PUFAS INDUCE ALPHA SYNUCLEIN-RELATED PATHOGENESIS: STUDIES ON BRAINS OF MOUSEMODELS OF PEROXISOMAL BIOGENESIS DISORDERS

**Authors:** E. Yakunin, D. J. Selkoe, R. Sharon

**Affiliations:** ^1^Department of Cellular Biochemistry and Human Genetics, The Hebrew University, Ein Kerem, Jerusalem 91010, Israel; ^2^Center for Neurologic Diseases, Harvard Institutes of Medicine, Boston, MA 02115, USA

The neuronal cytoplasmic protein, alpha-Synuclein (aS), has been implicated in the pathogenesis of Parkinson's disease (PD) at both the genetic and cytopathological levels. aS is a small, highly soluble synaptic protein; however, under pathological conditions it forms insoluble aggregates that are deposited in Lewy bodies, which are the pathological hallmark of PD. Previously we found that certain PUFA levels were elevated in tg-mice, overexpressing aS, and in PD patients' post-mortem brains. Interestingly, we identified polyunsaturated fatty acids (PUFAs) as a factor that induces aS oligomerization and aggregation in mesencephalic dopaminergic cell line. We now tested whether PUFAs also induce aS oligomerization and aggregation in vivo. For this aim, we used mouse models of Zellweger syndrome. A major metabolic consequence of this peroxisomal biogenesis disorder is accumulation of very long-chain fatty acids and specifically PUFAs. Indeed we found enhanced levels of soluble oligomers in Pex13-/- and Pex5-/- mice compared with normal mice. Further, we found that the increase in total aS protein was due to increased protein stability. We concluded that PUFAs induce aS oligomerization and aggregation in vivo and, therefore, act to induce PD-related pathogenesis.

# Abstract: PAIN RELIEF LEARNING IN FRUIT FLIES

**Authors:** A. Yarali, T. Niewalda, Y. C. Chen, S. Duerrnagel, H. Tanimoto, B. Gerber

**Affiliations:** University of Wurzburg

Painful stimuli may have appetitive after-effects: relief. Whether such relief is neuronally processed similar to natural rewards is, however, unclear. We address this question using the fruit fly. Electric shock can trigger two opposing predictions depending on its timing relative to an odour (Tanimoto et al 2004Nature): after odour-shock training, the odour predicts “danger” and therefore flies avoid the odour; in contrast, after shock-odour training, the odour may predict “relief ” and consequently flies approach the odour. We provide a parametric analysis of relief-learning focussing on effects of gender, training amount, shock intensity, odour identity, and odour intensity. Having established the optimal conditions, we asked whether conditioned approach after relief— learning shares neuronal requirements with sugar reward learning. In flies, octopamine is required for sugar reward learning, whereas it is dispensible for punishment learning (Schwaerzel et al, 2003 J Neurosci). We therefore tested for the role of octopamine in relief: learning, using the TßHM18 mutant that is impaired in octopamine biosynthesis. Relief: learning does not seem to require octopamine, whereas sugar reward learning does. This suggests distinct processing for the establishment relief versus reward memories. Current work now focuses on a possible role of dopamine and serotonine for relief learning.

# Abstract: BISTABILITY IN RELATION TO BEHAVIOR IN CAT CEREBELLAR PURKINJE CELLS

**Authors:** M. M. Yartsev, R. Givon, B. Lasry, O. Donchin

**Affiliations:** Department of Biomedical Engineering, Ben Gurion University, Beer Sheva 84105, Israel

Recent reports of bistability in vivo and in vitro in Purkinje neurons of the cerebellum have led to reevaluation of basic ideas regarding cerebellar function. Specifically, these neurons have been demonstrated to transit between two possible stable membrane potentials, which was also reflected in their bimodal firing pattern. However, these reports have been challenged by recent findings claiming that bistability does not exist in behaving animals, and that it is merely an artifact of the anesthetics, thus there is as yet no evidence of a functional relationship between bistability and behavior. By recording the extracellular activity of identified Purkinje cells we sought to examine this discrepancy and test whether bistability was increased, decreased, or unchanged during behavior that activated the region of the cerebellum from which we recorded. Specifically, we tested for changes during feeding in vermal lobule VI, a region of the cerebellum known for orofacial responses and increased activity during feeding. Indeed, in our neurons we found increased activity when the cat was actively feeding. In addition, we confirmed results in vitro and in anaesthetized animals that showed long pauses in the activity of Purkinje cells probably reflecting bistability in their membrane potential. We find this bistable activity in a substantial fraction of our cells, but many cells did not exhibit bistable behavior. Preliminary analysis of the relationship between behavior and bistability indicates that a fraction of the bistable cells modulate bistability in relation to behavior. We will present quantitative analysis of the fraction of the cells so modulated, and discuss the possible functional implications of these findings.

# Abstract: TOMOSYN INHIBITS EXOCYTOSIS INDEPENDENTLY OF SYNTAXIN BY INTERFERINGWITH VESICLE IMMOBILIZATION

**Authors:** O. Yizhar, N. Lipstein, S. E. Gladycheva, U. Matti, S. A. Ernst, J. Rettig, E. L. Stuenkel, U. Ashery

**Affiliations:** ^1^Department of Neurobiochemistry, Life Sciences Institute, Tel Aviv University, Tel Aviv 69978, Israel; ^2^Department of Molecular and Integrative Physiology, University of Michigan, Ann Arbor, Michigan, USA; ^3^Physiologisches Institut, Gebaude 59, University of Saarlandes, Homburg, Saar, Germany; ^4^Department of Cell and Developmental Biology, University of Michigan, Ann Arbor, Michigan, USA

Tomosyn is a cytoplasmic protein that binds to Syntaxin1 and SNAP-25 through an R-SNARE domain, forming a complex that is almost identical in structure to the neuronal SNARE complex. However, since tomosyn does not contain a vesicle-attachment transmembrane domain, this complex cannot support membrane fusion. Tomosyn was shown to inhibit exocytosis in a variety of cell types and these effects were attributed to direct competition between tomosyn's SNARE domain and the R-SNARE Synaptobrevin/VAMP. To characterize the effect of tomosyn overexpression on the attachment of vesicles to the plasma membrane, we used TIRF microscopy and examined the mobility of prefusion vesicles in the region directly adjacent to the membrane. Tomosyn caused a reduction in the pool of immobile vesicles, the same pool from which exocytosis preferencially takes place. To understand the contribution of the different domains of tomosyn to its inhibitory function, we used a tomosyn mutant that lacks the entire SNARE domain and does not bind Syntaxin. Using capacitance measurements we show that this mutant is a potent inhibitor of exocytosis, similar to the fulllength tomosyn. Overexpression of the SNARE domain of tomosyn by itself failed to inhibit exocytosis, indicating that this domain is not sufficient to perform the regulatory roles of tomosyn. However, overexpression of a mutant lacking 5 of the 9 WD40 repeats did not lead to inhibition of exocytosis although this mutant is still bound to Syntaxin. Our results indicate that tomosyn inhibits exocytosis by interfering with the immobilization of vesicles at the membrane. This effect is independent of Syntaxin and the integrity of the WD40 domain is crucial for tomosyn's inhibitory function.

# Abstract: NEUROPROTECTIVE ACTIVITY OF THE MULTIFUNCTIONAL ANTI-ALZHEIMER'SANTI- PARKINSON'S DRUG, LADOSTIGIL

**Authors:** M. B. H. Youdim, M. Yogev-Falach, O. Bar Am, T. Amit, O. Weinreb

**Affiliations:** Eve Top Centre, Technion–Israel Institute of Technology, Haifa 32000, Israel

The recent therapeutic approach in which drug candidates are designed to possess diverse pharmacological properties acting on multiple targets has stimulated the development of the bifunctional drug, Ladostigil, that combines the neuroprotective effects of the anti-Parkinson's drug, rasagiline, a selective monoamine oxidase (MAO)-B inhibitor, with the cholinesterase inhibitory activity of rivastigmine in a single molecule, as a potential treatment for Alzheimer and Lewy body diseases. Here, we assessed the dual effects of Ladostigil in terms of molecular mechanism of neuroprotection and amyloid precursor protein (APP) regulation/processing, using an apoptotic model of neuroblastoma SK-N-SH cells. Ladostigil dose-dependently decreased cell death via inhibition of the cleavage and activation of caspase-3 through a mechanism related to regulation of the Bcl-2 family proteins, resulting in reduced levels of Bad and Bax and induced levels of Bcl-2 gene and protein expression. Additionally, Ladostigil elevated brain-derived neurotrophic factor and the glial cell line-derived neurotrophic factor gene expressions. We have also followed APP regulation/processing and found that Ladostigil markedly decreased apoptoticinduced levels of holo-APP protein without altering APP mRNA levels, suggesting a post-transcriptional mechanism. In addition, this drug elevated phosphorylated protein kinase C levels and stimulated the release of the nonamyloidogenic alpha-secretase proteolytic pathway. Similar to Ladostigil, its S-isomer, TV3279, is a cholinesterase inhibitor but lacks MAO inhibitory activity, exerts neuroprotective properties, indicated that these effects are reside in the propargyl moiety rather than in the MAO inhibition moiety of the drugs. These findings indicate that the antiapoptotic-neuroprotective activity, accompanied by the ability to modulate APP processing, could make Ladostigil a potentially valuable drug for the treatment of Alzheimer's disease.

*Supported by Teva Pharmaceutical Co, Israel, and Technion Research and Development.*

# Abstract: VIRTUAL DEFINITION OF TISSUE BY CLUSTER ANALYSIS OF MULTIPARAMETRIC MR IMAGING (VIRTUAL-DOT-COMIMAGING)

**Authors:** Y. Yovel, Y. Assaf

**Affiliations:** Department of Neurobiochemistry, Faculty of Life Sciences, Tel Aviv University, Tel Aviv 69978, Israel

Resolution and contrast limits of MRI are the main factors that restrict our ability to segment and define certain tissues. While typical brain image resolution lies in the order of 1-2 mm, some CNS structures are smaller than that. In this work we devised an algorithm that detects and defines small subcortical regions based on contrast enhancement and cluster analysis. In order to test the algorithm we applied it on the thalamus. Histologically, the thalamus is composed of at least 9 different nuclei. These nuclei have different cytoarchitectonics and different functions. It is expected that the different cyto-architecture will be differentially weighted in a multicontrast MRI protocol. These contrast differences combined with clustering algorithm might allow us to define the subnuclei. 9 healthy male subjects aged 25–30 underwent MRI in a 3T scanner. Each volunteer was subject to 10 different image contrasts with resolution of 1.5mm3. The algorithm included the following steps: (1) selection of region of interest (ROI), (2) stretching of the contrast dynamic range, (3) transformation of the data into its principal component (PC) space, and (4) clustering. The clustering algorithm detected as many as 7 significantly different clusters in the thalamus. In addition, high symmetry between the two thalami of the same subject was observed. These clusters were assigned to the different thalamus nuclei-based histologic atlases that were digitized and coregistered to our template. The position of the clusters found by our algorithm and the nuclei positions according to the atlas were very similar in all subjects. We found that using advanced image processing routines one can extend the use of MRI to study and define sub-structures for individual subjects. The use of the virtual-dot-com imaging algorithm enables not only to detect the subthalamic nuclei but also to characterize them in terms of contrast fingerprint.

# Abstract: EFFECT OF GLOBAL VERSUS LOCAL ATTENTION ON THE HUMAN EVOKED GAMMA-BAND RESPONSE

**Authors:** S. Yuval-Greenberg, C. S. Herrmann, L. Y. Deouell

**Affiliations:** ^1^Department of Psychology, The Hebrew University, Jerusalem 91010, Israel; ^2^Department of Biological Psychology, Otto-von-Guericke University of Magdeburg, Germany; ^3^Interdisciplinary Center for Neural Computation, The Hebrew University, Jerusalem, Israel

Gamma-band responses (GBR) in the human EEG were shown to correlate with application of Gestalt laws and object perception. A few of these studies focused on the perception of illusory contours as a marker for binding object parts. In such studies (eg, Tallon-Baudry et al, 1996) gammaband response was found to be larger in response to coherent Kanizsa objects (composed of “pacman” figures facing the center to create an illusory shape) than in response to incoherent Kanizsa objects (when the “pacmans” are facing outwards and thus not creating a shape). Since this type of binding is thought to be an early automatic process, task-related effects would challenge the hypothesis relating GBR to the binding mechanism. However, all previous studies have investigated the binding process in tasks explicitly requiring subjects to recognize illusory contours, that is, to bind object parts. The present experiment was designed to explore the effect of the task on the gamma effect. Two tasks were used with the same Kanizsa objects: a global task (requiring the recognition of illusory contours) and a local task (responding to the appearance of a dark gray pacman). Analysis compared the GBR to nontarget coherent Kanizsa objects with the GBR to incoherent Kanizsa objects. In the global task, coherent objects evoked larger GBR than incoherent objects at central and posterior sites around 100 ms post stimulus. However, the local task eliminated this effect and there was no difference in the GBR of coherent and incoherent objects. Since binding is thought to be an early automatic process, this finding suggests that GBR does not reflect binding per se but rather as a higher mechanism such as memory matching or the creation of semantic object representations.

# Abstract: WHAT YOU SEE IS NOT (ALWAYS) WHAT YOU HEAR: INDUCED GAMMA BAND RESPONSES REFLECT CROSS-MODAL INTERACTIONS IN OBJECT RECOGNITION

**Authors:** S. Yuval-Greenberg, L. Y. Deouell

**Affiliations:** ^1^Department of Psychology, The Hebrew University, Jerusalem 91010, Israel; ^2^Interdisciplinary Center for Neural Computation, The Hebrew University, Jerusalem, Israel

Gamma-band responses (GBR) are hypothesized to reflect neuronal synchronous activity at several levels of object representations, including low-level Gestalt perception, featurebinding, and memory. However, it is not known whether synchrony in the gamma range is also related to the activation of multisensory object processing. We investigated the effect of semantic congruity between auditory and visual information on the GBR. The paradigm consisted of a simultaneous presentation of pictures and vocalizations of animals, which were either congruent or incongruent. EEG was measured in seventeen students while they attended either the auditory or the visual stimulus and performed a recognition task. Behavioral results showed a congruity effect, indicating information from the unattended modality affected behavior. Visual information affected auditory recognition more than auditory information affected visual recognition, suggesting a bias towards reliance on visual information in object recognition. While the evoked (phase-locked) GBR was unaffected by congruity, the induced (non-phase-locked) GBR was increased for congruent compared to incongruent stimuli. This effect was independent of the attended modality. The results show that integration of information across modalities, based on semantic congruity, is associated with increased synchronization at the gamma band.

# Abstract: OVERLAPPING REPRESENTATION OF INTERNAL MODELS IN MOTOR AND PREMOTOR CORTEX

**Authors:** N. Zach, D. Inbar, Y. Grinvald, E. Vaadia

**Affiliations:** ^1^Physiology Department, Hadassah Medical School, Israel; ^2^ICNC Center for Neural Computation, The Hebrew University, Jerusalem, Israel

At is known that newly learned sensorimotor tasks are represented in the activity of motor cortical neurons. However, an open question regarding the function of cortical neurons is their capacity for multiple representations for several tasks. To address this question, we compared the response properties of M1 and premotor cortical neurons before and after learning two sensorimotor transformation tasks. One task (rotational transformation) involved angular deviation between cursor movement and hand movement and induced a specific enhancement in representation of the relevant direction. The second task (arbitrary association) involved learning association between target color and movement direction, and induced enhancement in representation of target color. We compared the neurons' activity during performance of center-out task before and after learning the transformations. We found that not only were both tasks represented in the motor cortices, but single neurons in both M1 and premotor cortex represented the two tasks simultaneously. These results demonstrate that the motor cortices can represent two tasks simultaneously in an overlapping manner.

# Abstract: MULTIMODAL ENHANCEMENT IN THE OPTIC TECTUM OF THE BARN OWL

**Authors:** Y. Zahar, Y. Gutfreund

**Affiliations:** Department of Physiology and Biophysics, Technion–Israel Institute of Technology, Haifa 32000, Israel

Many events in the everyday life are registered by the sense organs of more than one modality. However, our perception of the external world is unitary and coherent. A challenging question is how these physically distributed feature representations are combined in the brain to form one homogeneous percept. We investigated the role of temporal cues in the binding of visual and auditory features, using the barn owl's brain as a model system. We explored the ability of neurons in the optic tectum (OT), a midbrain structure concerned with orientation and attentive behaviors, to code the temporal features of the stimulus. Multiunit recordings were taken from neurons being exposed to 8 seconds of amplitude-modulated unimodal stimuli (auditory or visual), and bimodal stimuli consisting of auditory and visual stimuli presented together congruently or with unmatched fundamental frequencies. We used vector strength analysis (VS) to quantify the neurons ability to phase lock to the fundamental frequency of the stimulus. Our results demonstrate that, at the low fundamental frequency range, in many neurons, the VS of the spike train at the stimulus fundamental frequency was larger for congruent bimodal stimuli than for unimodal or unmatched bimodal stimuli. Interestingly, bimodal stimulation resulted with an increase in the response (number of spikes) above the unimodal response at the onset of the stimulus, but not at subsequent periods (probably due to adaptation). Thus, visual-auditory integration was manifested in better phase locking to the stimulus and not in a change in the overall spike count. The observation that VS enhancement was significantly larger at congruent bimodal stimuli demonstrates that this multimodal enhancement is based on temporal cues. Our findings provide a better understanding of the way in which multisensory neurons synthesize cross-modal information, transforming it into an integrated product, which no longer resembles the individual unimodal inputs.

# Abstract: DIFFERENTIAL EFFECTS OF NEUROTRANSMITTERS ON TOPO I ACTIVITY IN MOUSE CEREBELLAR SECTIONS

**Authors:** E. Zehorai, M. Hershfinkel, I. Sekler, E. Priel

**Affiliations:** ^1^Departments of Microbiology and Immunology, Ben Gurion University of the Negev, Beer Sheva 84105, Israel; ^2^Departments of Morphology, Ben Gurion University of the Negev, Beer Sheva 84105, Israel; ^3^Departments of Physiology, Ben Gurion University of the Negev, Beer Sheva 84105, Israel

Topoisomerase I (topo I) is a nuclear enzyme participating in most DNA transactions by controlling the topological state of the DNA. We showed that the various mouse brain regions, although exhibited a relatively high topo I activity, differed in the level of the enzyme activity and protein. This topo I activity was age and sex dependent and a specific distribution pattern of this enzyme was observed. Inhibitory neurons contained the highest topo I activity and protein level not only in the nucleus but also in the cytoplasm. These results suggest that topo I might possess a specific, yet unknown, role in the brain. To further investigate this possibility we examined the effect of neurotransmitters on the activity of topo I in mouse cerebellar sections. Coronary cerebellar sections of 400 μm were prepared from 3 months old male mice. Following a 1 hour recovery period, the slices were treated with various concentrations of neurotransmitters for different intervals. Topo I activity, protein level, and posttranslational modifications of the enzyme protein were examined. Exposure of the cerebellar slices to glutamate for 1 or 5 minutes revealed an increased inhibition of the enzyme activity with time, while GABA treatment revealed an immediate inhibition of topo I which was diminished with time. No changes in the level of topo I protein were observed, suggesting post-translational modifications of these enzymes that occur following the neurotransmitters treatments. Pretreatment of the cerebellar slices with 3AB, a poly-ADP ribose polymerase inhibitor, prevented the glutamate but not the GABA induced inhibition of topo I. The present results together with our previous published data suggest that the brain topo I activity is modified by neurotransmitters probably due to activation of PARP. All together, these data suggest that topo I possesses a specific role in the brain which differs fromits known function.

# Abstract: TWO FUNCTIONALLY OPPOSING FRAGMENTS OF F-SPONDIN CONSTRICT THE COMMISSURAL AXON BETWEEN THE FLOOR PLATE AND THE PIA

**Authors:** S. Zisman, K. Marom, L. Rinsky-Halivni, A. Klar

**Affiliations:** Department of Anatomy and Cell Biology, Hadassah Medical School, The Hebrew University, Jerusalem, Israel

The formation of neuronal networks is governed by a limited number of guidance molecules, yet is immensely complex. The complexity of guidance cues is attained by a combinatorial code of guidance molecules and their receptors, intracellular signaling at the growth cone, and ligand and receptor modifications. We report here that the cleavage of the floor plate guidance molecule F-spondin generates two functionally opposing fragments: a short-range repellent protein deposited in the membrane of floor plate cells, and an adhesive protein that accumulates at the basement membrane. The coordinate activity of both constricts commissural axons to the basement membrane beneath the floor plate cells. We further demonstrate that the repulsive activity of the inhibitory fragment of F-spondin requires its presentation by the receptor ApoER2, which is expressed in the floor plate. Thus, two novel modifications elicit F-spondin activity, namely, the orchestrated generation of two functionally opposing polypeptides from a single protein by proteolysis, and immobilization by a membranal receptor.

*Supported by ISF, BSF, and GIF.*

# Abstract: IMMUNE CELLS CONTRIBUTE TO NEUROGENESIS UNDER BOTH NORMAL AND PATHOLOGICAL CONDITIONS

**Authors:** Y. Ziv, H. Avidan, A. Finkelstein, N. Ron, E. Yoles, J. Kipnis, M. Schwartz

**Affiliations:** ^1^Department of Neurobiology, The Weizmann Institute of Science, Israel; ^2^Proneuron Biotechnologies, Weizmann Science Park, Ness-Ziona 74101, Rehovot 76100, Israel; ^3^University of Nebraska Medical Center, Omaha, NE 68198-5800, USA

Neurogenesis is known to continuously take place in certain “neurogenic” areas of the adult central nervous system (CNS) and can be induced in “nonneurogenic” areas (eg, cortex and spinal cord) under traumatic or degenerative conditions. Recently we have introduced T cells and CNSresident microglia as important players in the regulation of adult neurogenesis. Under normal conditions, immunedeficient mice (SCID and nude) and transgenic mice that most of their T-cell pool is specific for an irrelevant antigen (ovalbumin) exhibited impaired hippocampal neurogenesis. In contrast, mice in which the majority of T cells specifically recognize the CNS-abundant antigen myelin basic protein showed normal neurogenesis. Using a rat model of cerebral ischemic insult and a mouse model of spinal cord injury (SCI) we tested whether CNS-specific T cells can facilitate neurogenesis in nonneurogenic brain areas. We demonstrated that T-cell-based immune activation following stroke induces a robust elevation of neurogenesis in the hippocampus as well as in the cerebral cortex. Following SCI, a two-fold increase in neurogenesis from endogenous progenitor cells was observed in mice that received a dual treatment of immunization with a weak agonist of myelin-derived peptide and injection of adult neural progenitor cells into the CSF. These neurogenesis-promoting effects were correlated with microglial production of BDNF and noggin. Our results suggest that T cells, acting via resident antigen presenting cells, are important regulators of adult neurogenesis under both physiological and pathological conditions.

# Abstract: NEURAL PROCESSING OF SUBJECTIVE PAIN EXPERIENCE: THE EFFECT OF STATE AND TRAIT

**Authors:** M. Ziv, R. Tomer, R. Defrin, T. Hendler

**Affiliations:** ^1^Department of Psychology, University of Haifa, Haifa 31905, Israel; ^2^Department of Physical Therapy, Sackler Faculty of Medicine, Tel Aviv University, Tel Aviv 69978, Israel; ^3^Functional Brain Imaging Unit, Tel Aviv Sourasky Medical Center, Tel Aviv University, Israel

Individuals differ in their subjective experience of pain. This diversity is partially mediated by personality traits which affect the way one attends to negative cues in the environment. Another important factor is preceding anticipation processes, which can evoke an emotional state of fear or anxiety. Thus, the association between expectancy processes (state) and the tendency to avoid harm (trait) might predetermine the subjective experience of pain. Using fMRI we investigated the neural mechanism that underlies such possible association. Twelve subjects were scanned while receiving a sequence of twelve subjectively defined painful warm stimuli to their wrist intermixed with events of nonpainful warm stimuli. Six of the painful stimuli were preceded by a warning signal and six were not. Following each stimulus participants rated the pain intensity on a ten point scale. The results showed that, on average, the rating of the expected painful stimuli was higher than the rating of the unexpected ones (*P* < .001). However, this difference was significant only for half of the subjects. Brain signals during the anticipation interval differentiated between the two behaviorally distinctive subgroups. Whole brain analysis revealed that subjects who reported expected pain asmore painful then unexpected pain exhibited greater activity in the midbrain, the cingulate cortex, the dorsolateral prefrontal cortex, and the hippocampus. Correlating whole brain activity during the anticipation interval with harm-avoidance trait scores demonstrated distributed increased brain activation in the putamen, the anterior cingulate cortex, and the amygdala. The results of this study elucidate two possible neural mechanisms that mediate the subjective experience of pain; one includes the hippocampus and is involved in the processing of preceding cues (state) while the other, which includes the amygdala, relates to personality differences (trait).
